# Highly congested spiro-compounds via photoredox-mediated dearomative annulation cascade

**DOI:** 10.1038/s42004-022-00706-3

**Published:** 2022-08-05

**Authors:** Chao Zhou, Andrey Shatskiy, Azamat Z. Temerdashev, Markus D. Kärkäs, Peter Dinér

**Affiliations:** 1 Department of Chemistry, Division of Organic Chemistry, KTH Royal Institute of Technology, Teknikringen 30, 10044 Stockholm, Sweden; 2 Department of Analytical Chemistry, Kuban State University, Stavropolskaya St. 149, 350040 Krasnodar, Russia

**Keywords:** Photocatalysis, Synthetic chemistry methodology

## Abstract

Photo-mediated radical dearomatization involving 5-*exo*-trig cyclizations has proven to be an important route to accessing spirocyclic compounds, whereas 6-*exo*-trig spirocyclization has been much less explored. In this work, a dearomative annulation cascade is realized through photoredox-mediated C–O bond activation of aromatic carboxylic acids to produce two kinds of spirocyclic frameworks. Mechanistically, the acyl radical is formed through oxidation of triphenylphosphine and subsequent C–O bond cleavage, followed by a 6-*exo*-trig cyclization/SET/protonation sequence to generate the spiro-chromanone products in an intramolecular manner. Furthermore, the protocol was extended to more challenging intermolecular tandem sequences consisting of C–O bond cleavage, radical addition to an alkene substrate, and 5-*exo*-trig cyclization to yield complex spirocyclic lactams.

## Abstract (web summary)

Photo-mediated radical dearomatization involving 5-*exo*-trig cyclizations has proven to be an important route to accessing spirocyclic compounds, whereas 6-*exo*-trig spirocyclization has been much less explored. Here, a dearomative annulation cascade is realized through a photoredox-mediated C–O bond activation of aromatic carboxylic acids to produce two kinds of spirocyclic frameworks, whereby the spirocyclizations are triggered by acyl radical formation from benzoic acids leading to spiro-chromanones via a direct intramolecular 6-*exo*-trig cyclization or spirocyclic lactams via an intermolecular addition/5-*exo*-trig cyclization cascade.

## Introduction

**Fig. 1:**
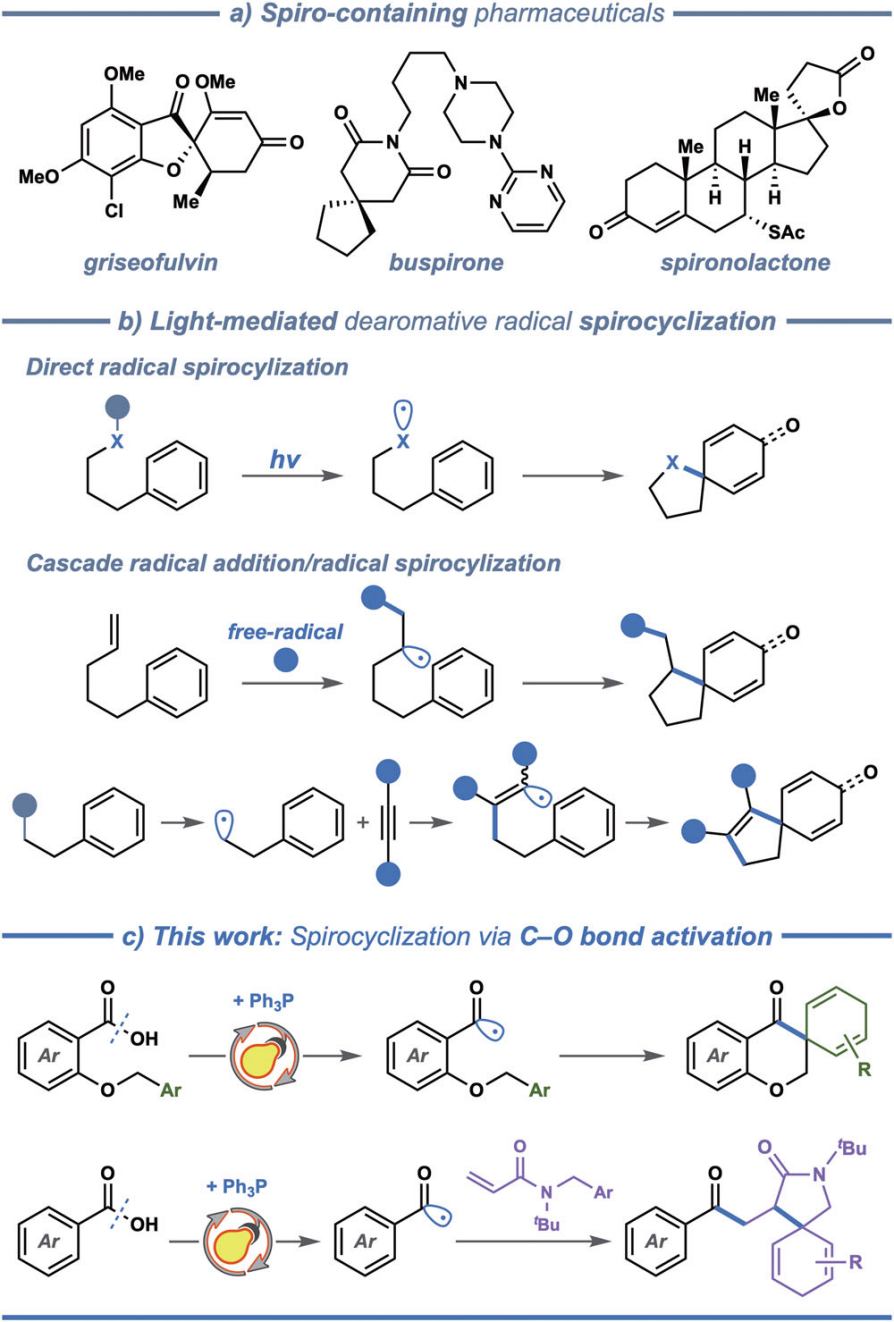
Photocatalytic construction of spiro-compounds via radical dearomatization. **a** Examples of spiro-containing pharmaceuticals. **b** Different strategies for light-mediated dearomative radical spirocyclization. **c** This work: spirocyclization via C–O bond activation of carboxylic acids.

Over the past decades, diversity-oriented synthesis (DOS) has evolved to yield a number of strategies for increasing the diversity and complexity of pharmaceutically relevant molecules^1–5^. Among these, increasing the content of quaternary carbon centers is regarded as a prime tool for increasing the three-dimensionality of potential drug candidates. An attractive strategy for creating three-dimensionality is through introduction of spiro centers, which are ring systems that have two rings linked together by one common atom^6–8^. Several commercially available drugs, including grisefulvin, buspirone, and spironolactone, contain such spirocyclic motifs (Fig. 1a)^9–11^. A number of synthetic protocols have been established for efficient construction of spirocyclic compounds^12,13^, among which dearomative spirocyclization offers a particularly straightforward route to spiro-compounds with a minimal number of synthetic steps^14,15^. Although *ipso*-annulation towards spirocycles has mainly focused on transition metal-catalyzed, electrophilic or nucleophilic dearomatization reactions^15^, radical pathways have gained increased attention^16^, promoted by the recent developments in radical chemistry^17^.

Visible light-promoted photocatalysis is a sustainable and versatile synthetic tool^18–20^, which has recently revitalized the radical-mediated dearomatization manifolds aiming at accessing spirocyclic compounds^16,21,22^. The related synthetic strategies presented to date can be divided into direct strategies proceeding through radical *ipso*-cyclization^23–33^ and cascade strategies, where radical *ipso*-cyclization is preceded by radical addition reactions (Fig. 1b)^34–49^. Examples of the first category include photocatalytic manifolds developed by Wang and co-workers^25–27^, which allow accessing spiro γ-lactam indoline derivates through sequential radical dearomatization/hydroxylation, followed by either oxidation or nucleophilic substitution. In several related systems disclosed by Samec^29^, Cho^30^, and Cariou groups^31^, photooxygenation provides access to spirolactone, spiroazalactam and spirolactam cyclohexadienone products with molecular oxygen as the terminal oxidant. These reactions proceed via 5-*exo*-trig cyclization of carboxylate, iminyl, and *N*-amide radicals. Additionally, Jui and coworkers reported hydroarylation^32^ and hydroalkylation^33^ of arenes to construct spirocyclohexadiene skeletons through a reductive radical-polar crossover mechanism, in which aryl or alkyl radicals derived from aryl- and alkyl halides trigger regioselective 5-*exo*-trig radical spirocyclization. The second class of spirocyclization reactions involves visible light-promoted cascade radical addition and sequential radical *ipso*-cyclization reactions, which provide access to a range of spirocyclohexadienones^34–49^. In these sequential radical additions between diverse radicals, including alkyl^34–43^, acyl^44^, aryl radicals^45^, selenium-^46,47^, and sulfur-centered radicals^36,48,49^, as well as carbon-carbon double or triple bonds, the spirocyclohexadienones are formed via 5-*exo*-trig cyclization and hydroxylation/oxidation. Most of these photo-mediated radical dearomatization manifolds involve 5-*exo*-trig cyclizations, while 6-*exo*-trig cyclization manifolds are less explored, and the target products are mostly spirocyclohexadienones rather than spirocyclohexadienes. These limitations are mainly due to the challenges in controlling the generation of radicals and the subsequent selective cyclization^16^, and therefore, it is essential to develop new visible light-promoted routes to spirocyclic compounds, especially via 6-*exo*-trig spirocyclization.

### Design plan

**Fig. 2:**
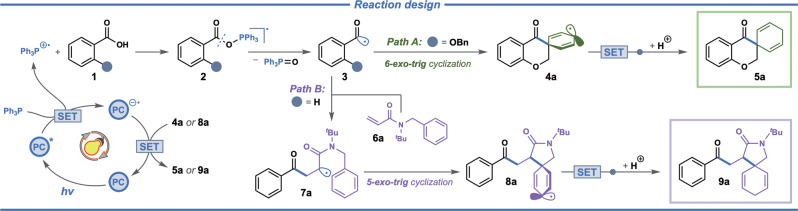
Reaction design. Proposed photocatalytic cycle for intramolecular (Path A) and intermolecular (Path B) construction of spiro-compounds.

Acyl radicals generated from the corresponding aldehydes, acyl chlorides, and other precursors are important building blocks for the construction of carbonyl compounds^50^ and have been applied widely in the visible-light photocatalysis^51,52^. However, the direct generation of acyl radicals from readily available benzoic acids is largely unexploited due to the limitation of convenient catalytic systems^53–59^. In our reaction design, we aimed at utilizing the acyl radical as the key radical intermediate, generated from aromatic carboxylic acids, to form two different spirocyclohexadienes via both direct and indirect routes (Fig. 1c). As shown in the reaction design (Fig. 2), triphenylphosphine is oxidized by the excited iridium photocatalyst to the triphenylphosphine cation-radical via single-electron transfer. The triphenylphosphine cation-radical reacts with the deprotonated aromatic acid **1** to form the adduct **2**, which undergoes β-selective C(acyl)–O bond cleavage, forming the acyl radical **3** concomitant with the loss of triphenylphosphine oxide. The acyl radical undergoes 6-*exo*-trig spirocyclization via dearomatization of the aromatic ring, leading to the cyclohexadienyl radical **4a**. Finally, one-electron reduction yields the cyclohexadienyl anion, which is further protonated to give the chromanone-based spiro compound **5a** (Path A, Fig. 2). An alternative approach to access spiro compounds via the acyl radical is to trap the acyl radical by a somophile leading to a new α-carbonyl radical that can undergo dearomative spirocyclization. In our alternative reaction design, the acyl radical **3**, derived from benzoic acid **1**, participates in radical addition to *N*-benzyl-*N*-(*tert*-butyl)acrylamide **6a** to afford α-carbonyl radical intermediate **7a**. This radical undergoes 5-*exo*-trig cyclization to yield a new cyclohexadienyl radical **8a** that upon one-electron reduction and protonation provides the desired spirolactam **9a** (Path B, Fig. 2).

## Results and discussion

### Optimization of the reaction conditions

In order to investigate the feasibility of the envisioned transformation, the spirocyclization reaction was first attempted with 2-(benzyloxy)benzoic acid **1a** and triphenylphosphine. After an extensive screening of the reaction conditions (Table 1 and Supplementary Tables 1–4), we were delighted to find that the desired spirocyclic product **5a** was furnished in 66% yield under visible light irradiation (440 nm LEDs) in the presence of an iridium-based photocatalyst (Table 1, entry 1). The use of aqueous acetonitrile (CH_3_CN/H_2_O, 85:15 vol%) as the solvent was found to be pivotal for achieving the desired transformation (Supplementary Tables 1 and 2). In the absence of water or with an increased content of water (up to 50 vol%) a significant decrease in yield was observed (Table 1, entries 2 and 3, respectively). The use of alternative Ir-based or organic photocatalysts, including 4DPAIPN and 3DPAFIPN (Table 1, entries 4 and 5, respectively, and Supplementary Table 1), led to deteriorated yields of the desired product.

**Table 1:** Optimization of the reaction conditions^[a]^.


Entry	Variations	Yield[b]
1	None	66%
2	CH_3_CN as the solvent	9%
3	CH_3_CN/water 1:1 as the solvent	13%
4	4DPAIPN as the photocatalyst	6%
5	3DPAFIPN as the photocatalyst	13%
6	No K_3_PO_4_	trace
7	Na_2_CO_3_ instead of K_3_PO_4_	47%
8	DMAP instead of K_3_PO_4_	6%
9	1 equiv. of PPh_3_	58%
10	No light or no photocatalyst	–

^[a]^Standard reaction conditions: **1a** (0.3 mmol), photocatalyst (1 mol%), PPh_3_ (1.5 equiv.), K_3_PO_4_ (1 equiv.), CH_3_CN/H_2_O (85:15 vol%, 6 mL), N_2_, blue LEDs (440 nm), 36 h reaction time, fan cooling (ca. 35–40 °C). ^[b]^Yields were determined by ^1^H NMR analysis of the crude reaction mixtures using 4-nitrobenzonitrile as the internal standard.

The screening of various inorganic and organic bases identified tribasic potassium phosphate as the optimal base for the reaction (Table 1, entries 6–8 and Supplementary Table 2). Furthermore, the use of an excess of triphenylphosphine (1.5 equiv.) was shown to increase the yield of the desired product (Table 1, entry 9 and Supplementary Table 3). In the absence of either light irradiation or photocatalyst, no conversion of the starting material was observed (Table 1, entry 10).

### Investigation of the substrate scope

**Fig. 3:**
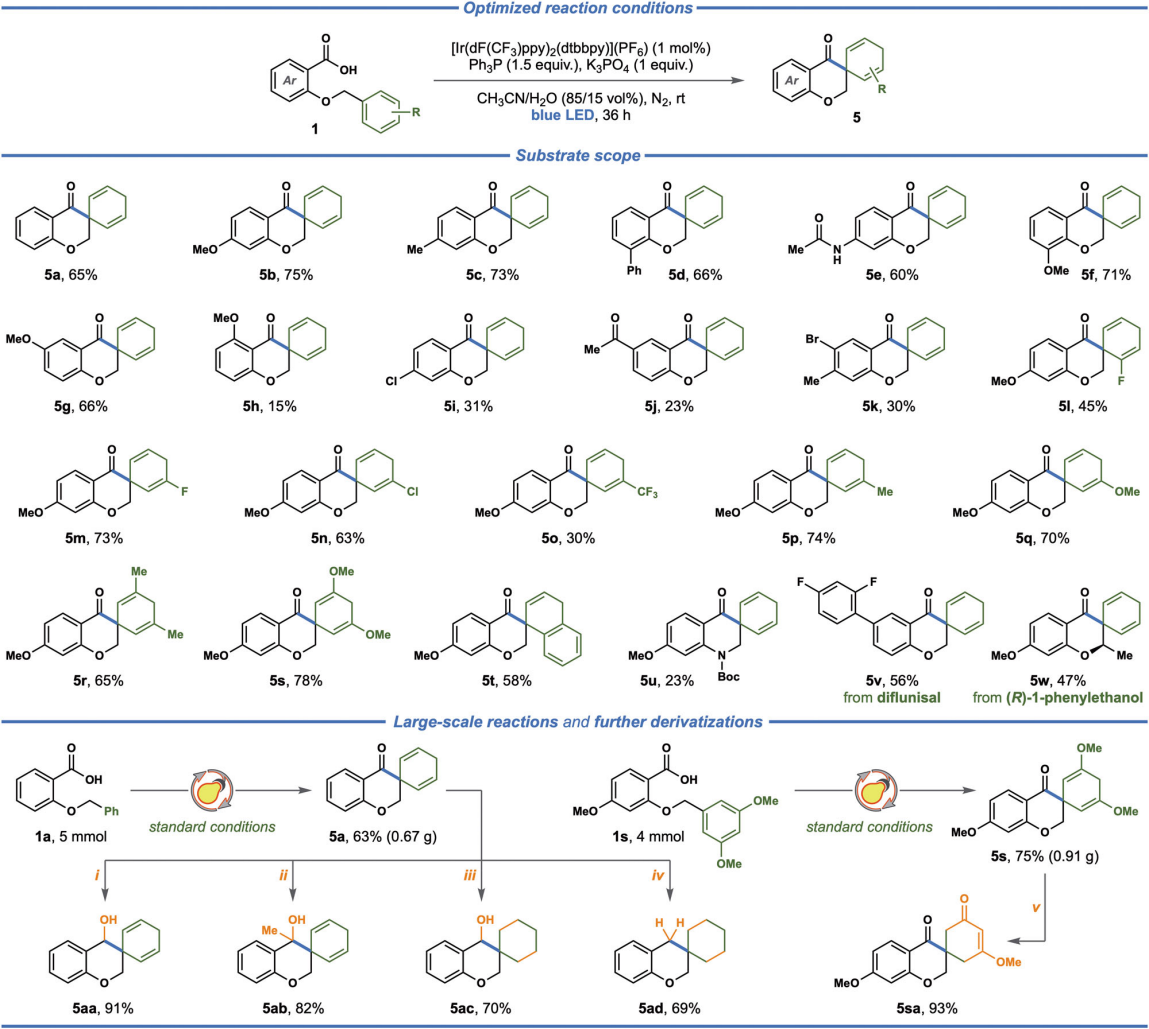
Scope for the radical-based intramolecular dearomative spirocyclization. Reaction conditions: carboxylic acid (0.3 mmol, 1 equiv.), triphenylphosphine (1.5 equiv.), K_3_PO_4_ (1 equiv.), [Ir(dF(CF_3_)ppy)_2_(dtbbpy)](PF_6_), (1 mol%) CH_3_CN/H_2_O (85:15 vol%, 6 mL), N_2_, blue LEDs (440 nm), 36 h, fan cooling (35–40 °C). Further derivatization of products **5a** and **5s** were performed under the following conditions: (i) NaBH_4_, MeOH, 0 °C, 2 h; (ii) MeMgBr, THF, 0 °C, 16 h; (iii) H_2_, Pd/C, MeOH, 12 h; (iv) H_2_, Pd/C, MeOH, 60 h; (v) TFA, CH_2_Cl_2_, 16 h.

Using the optimized reaction conditions (Table 1), we explored the generality of the developed photochemical transformation. As illustrated in Fig. 3., a range of substituted 2-(aryloxy)benzoic acids were shown to engage in the radical-mediated dearomative spirocyclization to provide the corresponding spiro-chromanones. The reaction proved efficient for 2-(benzyloxy)benzoic acids that bear electron-neutral or electron-rich substituents at the carboxylate-functionalized aromatic ring. Accordingly, the substrates equipped with methoxy (**5b**, **5f**, **5g**), methyl (**5c**), phenyl (**5d**) and acetamide (**5e**) groups provided the expected products in generally good yields (**5b**–**5g**, 65–75%), except for the substrate with a methoxy-group at the *ortho*-position (**5h**, 15%). The substrates featuring electron-withdrawing substituents at the carboxylate-functionalized aromatic ring, including chloro (**5i**), bromo (**5k**) and acyl groups (**5j**), provided the corresponding products in consistently lower yields (**5i**–**5k**, 23–31%). Next, substrates with various substituents at the benzyloxy functionality were investigated. Halogenated substates featuring fluoro (**5l**, **5m**) and chloro groups (**5n**) were well-tolerated and provided the expected spirocyclic products for both *ortho*- (**5l**, 45%) and *meta*-substituted (**5m**, **5n**, 63–73%) substrates. Here, competitive dehalogenation was not observed, providing products with handles for further functionalization of vinylic motifs. Introducing methyl (**5p**) or methoxy groups (**5q**) at the *meta*-position of the benzyloxy ring was also well-tolerated (**5p, 5q**, 70–74%), while a CF_3_-functionalized substrate provided the desired product in lower yield (**5o**, 30%). The related disubstituted substrates bearing methyl (**5r**) and methoxy groups (**5s**), as well as a naphthalene functionality (**5t**) furnished the desired products in good yields (**5r**–**5t**, 58–78%). Unfortunately, *para*-substituted substrates, including 4-fluoro, 4-methyl, and 4-pyridinyl substrates (see Supplementary Table 6) did not provide the desired products. Furthermore, a Boc-protected aniline-derived substrate provided the corresponding *N*-heterocyclic spiro product, albeit in low yield (**5u**, 23%), while other linkers (aliphatic, thioether, amide, amino, see Supplementary Table 6) showed poor or no reactivity. In reactions with lower yields, e.g., with benzoic acids **1j** and **1i**, the reaction mixture after 36 h consisted of unreacted starting material (30% and 27%, respectively) together with trace amount of the aldehyde side product as determined by ^1^H NMR spectroscopy. The radical-mediated dearomative spirocyclization protocol was also successfully applied to pharmaceutically relevant molecules. The substrate derived from diflunisal^60^—a salicylic acid derivative with analgesic and anti-inflammatory properties—engaged in the spirocyclization reaction to provide the corresponding product in 56% yield (**5v**).

Similarly, the substrate functionalized with (*R*)-(+)-1-phenylethanol was converted to the corresponding spiro-chromanone in 47% yield with the stereogenic center being intact (**5w**, see Supplementary Figs. 93, 94). The synthetic utility of the developed protocol was demonstrated by upscaling two of the model reactions and subsequent conversion of the resulting products into a range of spirocyclic derivatives (Fig. 3, *bottom*). To our delight, increasing the scale of the reaction (from 0.3 mmol to 4 or 5 mmol) provided the expected products **5a** and **5s** in 63% and 75% yields, respectively, that is with nearly identical efficiency compared to the smaller-scale reactions. The carbonyl group in spiro-chromanone **5a** was efficiently reduced to the corresponding secondary alcohol with NaBH_4_ (**5aa**, 91%) or converted to the corresponding methyl alcohol with MeMgBr (**5ab**, 82%), while preserving the 1,4-diene functionality. In contrast, hydrogenation of compound **5a** with hydrogen over Pd/C resulted in facile reduction of the 1,4-diene moiety. In this reaction, concomitant selective hydrogenation of the carbonyl group to the secondary alcohol was observed at shorter reaction times (**5ac**, 70%), while at longer reaction times the initially formed alcohol functionality undergoes reductive deoxygenation to yield a completely saturated product (**5ad**, 69%). In addition, the trimethoxy-substituted spiro-chromanone (**5s**) was readily converted to cyclohexenone derivative **5sa** in 93% yield upon treatment with trifluoroacetic acid.

**Fig. 4:**
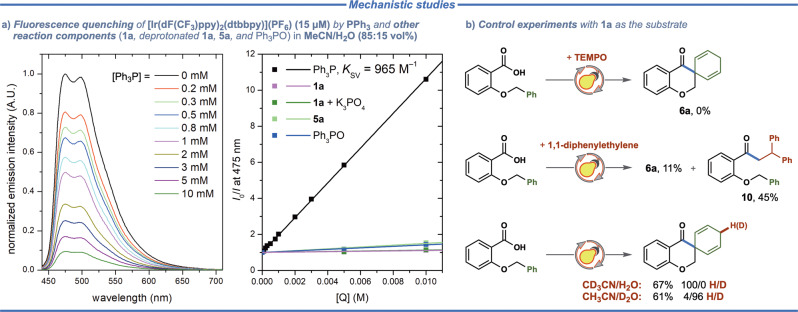
Mechanistic studies. **a** Fluorescence quenching of [Ir(dF(CF_3_)ppy)_2_(dtbbpy)](PF_6_) (15 µM) by PPh_3_ and other reaction components (**1a**, deprotonated **1a**, **5a**, and Ph_3_PO) in MeCN/H_2_O (85:15 vol%). **b** Control experiments with substrate **1a** with radical traps (TEMPO and 1,1-diphenylethylene) and different deuterated solvents.

The key mechanistic aspects of the developed transformation were elucidated by a series of experiments (Fig. 4). First, efficient quenching of Ir-based photocatalyst by triphenylphosphine was confirmed through fluorescence quenching studies (Fig. 4a, *K*_SV_ = 965 M^–1^), while no appreciable quenching was observed for the starting material (**1a** and deprotonated **1a**, *K*_SV_ = 11 and 13 M^–1^, respectively). A slightly increased quenching was observed for the product **5a** and the triphenylphosphine oxide (*K*_SV_ = 50 and 42 M^–1^, respectively).

Addition of an excess of 2,2,6,6-tetramethyl-1-piperidinyloxy (TEMPO) to the reaction mixture under standard conditions completely inhibited formation of the spirocyclic product (Fig. 4b), supporting the free-radical nature of the developed transformation. Furthermore, formation of the proposed acyl radical intermediate was confirmed by a trapping experiment with an excess of 1,1-diphenylethylene. As evident from the distribution of cyclic and linear products in this reaction (4:1 cyclic/linear), intermolecular trapping of the acyl radical by 1,1-diphenylethylene easily outcompetes the intramolecular spirocyclization step under the employed reaction conditions. Finally, deuterium labeling experiments with CD_3_CN/H_2_O or CH_3_CN/D_2_O as the solvents firmly support that the terminal step of the developed reaction proceeds through one-electron reduction of the spirocyclic C-radical intermediate, followed by protonation of the formed carbanion.

**Fig. 5:**
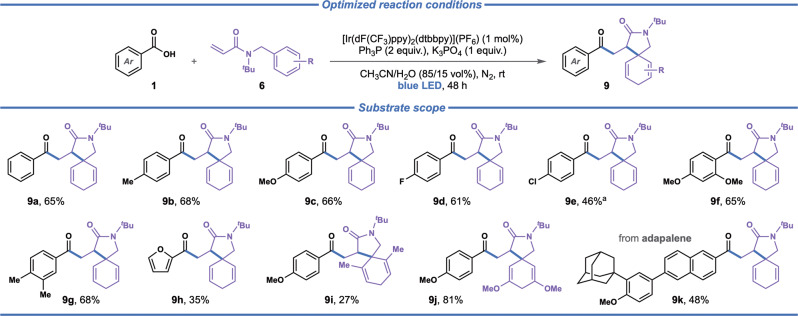
The scope for intermolecular radical dearomative spirocyclization. Reaction conditions: carboxylic acid (0.2 mmol, 1 equiv.), acrylamide acceptor (2 equiv.), triphenylphosphine (2 equiv.) K_3_PO_4_ (1 equiv.), [Ir(dF(CF_3_)ppy)_2_(dtbbpy)](PF_6_) (1 mol%), CH_3_CN/H_2_O (85:15 vol%, 4 mL), N_2_, blue LEDs (440 nm), 48 h, fan cooling (35–40 °C). ^a^carboxylic acid (2 equiv.), acrylamide acceptor (0.2 mmol, 1 equiv.).

Subsequently, we investigated the feasibility of the developed protocol for synthesis of spiro-lactams through a related intermolecular mechanism. This reaction was envisioned to proceed through intermolecular addition of the photochemically-generated acyl radical to a *N*-benzyl-*N*-(*tert*-butyl)acrylamide somophile, followed by intramolecular spirocyclization of the formed C-radical intermediate. It has previously been shown that the *tert*-butyl group is beneficial to achieve spirocyclizations on the benzyl ring^33^, but the *tert*-butyl group can easily be removed under mild conditions using copper(II) triflate^61^. This transformation was expected to pose a significant challenge, as the high-energy acyl radical must undergo addition to a somophile in an intermolecular fashion, while outcompeting deleterious HAT and SET processes, that are less pronounced in the complementary intramolecular reaction. To our delight, the optimized conditions were readily applicable for the proposed intermolecular spirocyclization reaction (Tables S4 and S5), and its scope was evaluated on a range of benzoic acid and acrylamide derivatives (Fig. 5). Benzoic acids featuring substituents at the *para*-position, including methyl (**9b**), methoxy (**9c**) and fluoro substituents (**9d**), readily engaged in the reaction to deliver the corresponding spirocyclic lactams in reasonable yields (**9a**–**9d**, 61–68%). For benzoic acids having a more electron-withdrawing substituent, such as 4-chlorobenzoic acid, the target product was obtained in lower yield (**9e**, 46%). Trisubstituted benzoic acids equipped with methoxy (**9f**) and methyl groups (**9g**) were efficiently converted to the corresponding spirocyclic products (**9f**, **9g**, 65–68%). The heterocycle-containing 2-furoic acid was also tolerated, albeit providing the desired product in relatively low yield (**9h**, 35%). With respect to acrylamide acceptors with 4-methoxybenzoic acid as the acyl radical donor, the more sterically encumbered 2,6-dimethyl substituted somophile displayed poor reactivity (**9i**, 27%), while the electron-rich 3,5-dimethoxy substituted somophile provided the desired product in high yield (**9j**, 81%). Adapalene (**9k**), a third-generation topical retinoid primarily used in the treatment of acne, psoriasis, and photoaging^62^, delivered the expected spirocyclic product in reasonable yield (**9k**, 48%), highlighting the applicability of the disclosed protocol for late-stage modification of complex biologically relevant compounds.

**Fig. 6:**
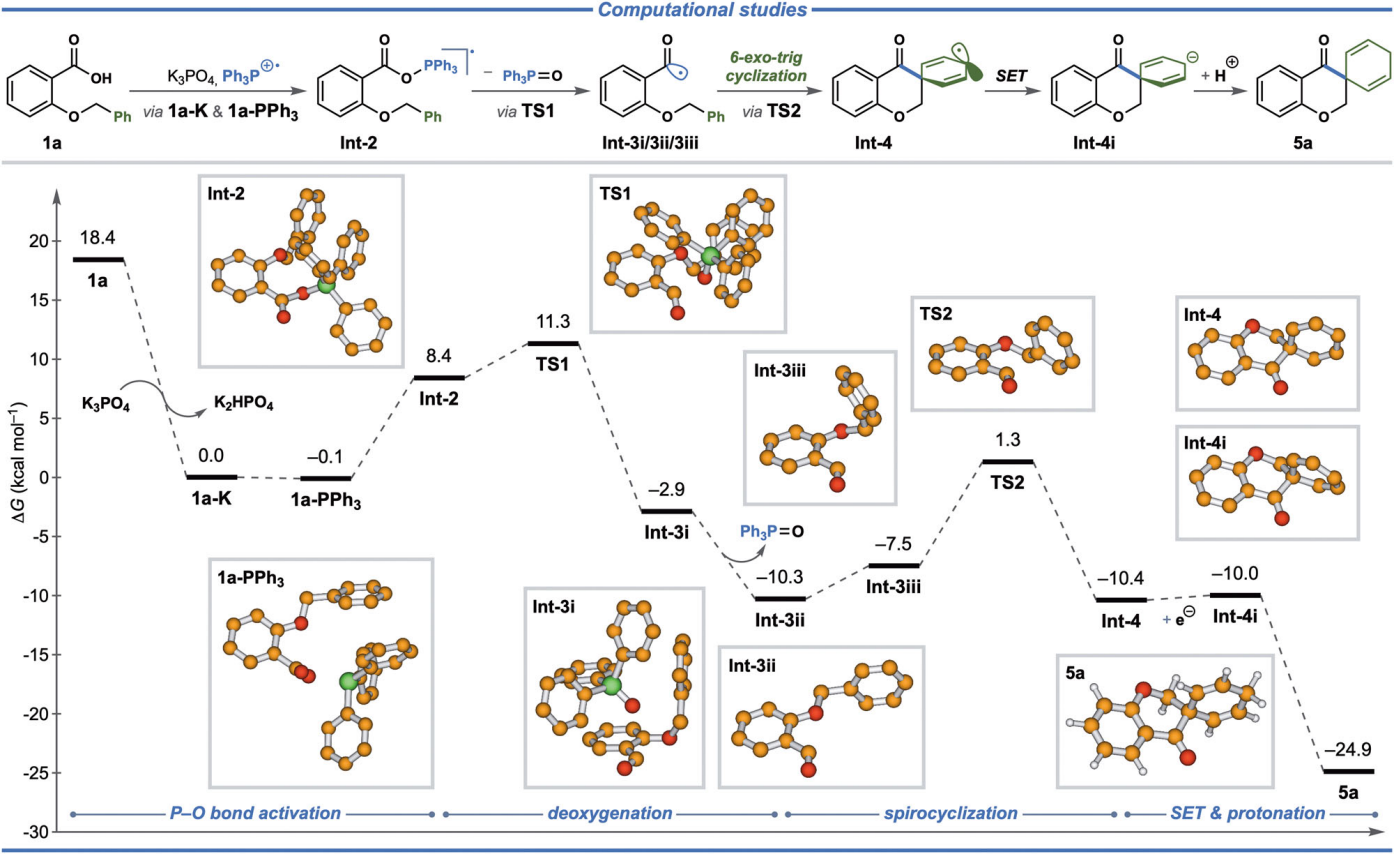
Mechanistic studies. DFT-investigation of the dearomatization mechanism at the B3LYP/6-311+G(d,p)–D3–CPCM(acetonitrile, UFF) level of theory.

Furthermore, the mechanism of the developed transformations was investigated using density functional theory (DFT) calculations at the B3LYP/6-311+G(d,p)-D3^63–66^ level of theory in combination with the Polarizable Continuum Model (CPCM, UFF, acetonitrile)^67,68^ as implemented in Gaussian 16 (Revision B.01)^69^. The photocatalytic generation of triphenylphosphinyl cation-radical was previously investigated by Xie, Zhu, and coworkers^70^. In their calculations, photoexcitation of [Ir(dF(CF_3_)ppy)_2_(dtbbpy)](PF_6_) was found endergonic by 58.0 kcal mol^–1^, while the following SET between the excited-state photocatalyst and Ph_3_P is exergonic by 0.8 kcal mol^–1^ with nearly null energy barrier (0.01 kcal mol^–1^). According to our calculations, deprotonation of carboxylic acid **1a** by K_3_PO_4_ to yield the potassium carboxylate **1a-K** was found highly exergonic, while the following replacement of the potassium cation by the triphenylphosphinyl cation-radical leads to the ionic complex **1a-PPh**_**3**_ of similar free energy (lowered by 0.1 kcal mol^–1^, Fig. 6). For this complex, the spin density of the cation-radical is localized almost exclusively on the phosphorus atom. In the following step of the reaction, formation of a covalent P–O bond between the phosphinyl radical cation and the carboxylate is accompanied by a spin density shift from the phosphorous center to the aromatic ring of the formed radical intermediate **Int-2**, which is less stable compared to the ionic intermediate **1a-PPh**_**3**_ by 8.5 kcal mol^–1^. Subsequently, intermediate **Int-2** undergoes homolytic C–O bond cleavage via **TS1** with an overall Gibbs free energy of activation of approximately 11 kcal mol^–1^ to form acyl radical complex **Int-3i** (Fig. 6)

The latter liberates triphenylphosphine oxide to provide the free acyl radical species **Int-3ii** in an exergonic process. The key spiro-cyclization step takes place from acyl radical **Int-3iii** via **TS2** to form the chromanone radical species in a slightly exergonic process. In the final stage of the reaction, SET from the reduced Ir(II) photocatalyst to the delocalized C-centered radical **Int-4** concludes the photocatalytic cycle in a slightly endergonic process (0.4 kcal mol^–1^). Finally, the thereby formed carbanion **Int-4i** is protonated by K_2_HPO_4_ to yield the final product **5a** in an exergonic process. The potential energy surface was also investigated for the tandem intermolecular radical addition/spirocyclization reaction (see Supplementary Table 8 and Supplementary Fig. 6). The reaction follows the same mechanistic pathway as the intramolecular spirocyclization through initial C–O bond cleavage (**TS3’**), and the activation barrier for the formation of the acyl radical from benzaldehyde is slightly lower (7.2 kcal mol^–1^) than for **1a** (see Supplementary Table 8 and Supplementary Fig. 6). In the presence of the acrylamide-based somophile, the acyl radical first undergoes radical addition to the somophile via **TS4’** (2.1 kcal mol^–1^), generating a stable α-carbonyl radical (–19.8 kcal mol^–1^). The α-carbonyl radical attacks at the *ipso*-carbon of the benzyl moiety in a 5-*exo*-trig fashion via **TS5’**, which leads to the delocalized radical to ultimately deliver the final product upon an overall exergonic SET/protonation sequence.

In summary, we have developed an effective, modular, and regioselective methodology to access two kinds of spirocyclic frameworks through visible light-mediated radical dearomatization. Originating from readily available benzoic acids in the presence of triphenylphosphine by photocatalysis, acyl radicals can either engage in the direct intramolecular 6-*exo*-trig cyclization to provide complex spiro-chromanones or be trapped by alkene-based somophiles, followed by a 5-*exo*-trig cyclization to furnish valuable spirocyclic lactams in an intermolecular tandem manner. The protocols show great substrate scopes and functional group compatibilities, emphasizing the synthetic viability and applicability in late-stage modification. This provides an efficient and versatile radical-based framework for constructing spiro-compounds, exhibiting promising practical applications in both academic and industrial settings.

## Methods

### Materials and procedures for synthesis of substrates and catalysts

See Supplementary Methods.

### Optimization of reaction conditions and general procedures, fluorescence quenching studies, and computational studies

See Supplementary Results and discussion, Supplementary Figs. 1–6 and Supplementary Tables 1–8.

### Characterization data

See Supplementary Note 1.

### NMR spectra and HPLC chromatograms of substrates and products

See Supplementary Note 2, Supplementary Figs. 7–129.

## Supplementary information

Supplementary Information

## Data Availability

Supplementary data are available in the online version of the paper. The supplementary data contains experimental procedures, ^1^H, ^13^C, ^19^F NMR spectra, XYZ coordinates for all optimized structures, figures of fluorescence quenching. Correspondence and reasonable requests for materials should be addressed to P.D.
