# Supplementary material for: Highly congested spiro-compounds via photoredox-mediated dearomative annulation cascade
Source: Commun Chem. 2022 Aug 5;5:92. doi: 10.1038/s42004-022-00706-3 (PMC9814605; doi:10.1038/s42004-022-00706-3)
Supplement: Supplementary file 1 — Supplementary Information [file 42004_2022_706_MOESM1_ESM.docx]

Supporting Information

**Highly Congested Spiro-Compounds via Photoredox-Mediated Dearomative Annulation Cascade**

Chao Zhou,^a^ Andrey Shatskiy,^a^ Azamat Z. Temerdashev,^b^ Markus D. Kärkäs^a^ and Peter Dinér*^a^

^a^ Division of Organic Chemistry, Department of Chemistry, KTH Royal Institute of Technology, Teknikringen 30, 100 44 Stockholm, Sweden

^b^ Department of Analytical Chemistry, Kuban State University, Stavropolskaya St. 149, 350040 Krasnodar, Russia

*Corresponding author e-mail: diner@kth.se

Table of Contents

[1. Supplementary methods 1](#_Toc108689433)

[1.1 Materials and methods 1](#_Toc108689434)

[1.2 General procedure for preparation of photocatalysts and substrates 2](#_Toc108689435)

[2. Supplementary Result and discussion 7](#_Toc108689436)

[2.1 Optimization of reaction conditions and general procedures 7](#_Toc108689437)

[2.2 Fluorescence quenching studies 13](#_Toc108689438)

[2.3 Computational studies 16](#_Toc108689439)

[3. Supplementary Note 1 54](#_Toc108689440)

[3.1 Characterization data for substrates and products 54](#_Toc108689441)

[2-(Benzyloxy)-6-methoxybenzoic acid (1h) 54](#_Toc108689442)

[2-(Benzyloxy)-5-bromo-4-methylbenzoic acid (1k) 54](#_Toc108689443)

[2-((2-Fluorobenzyl)oxy)-4-methoxybenzoic acid (1l) 54](#_Toc108689444)

[2-((3-Fluorobenzyl)oxy)-4-methoxybenzoic acid (1m) 54](#_Toc108689445)

[2-((3-Chlorobenzyl)oxy)-4-methoxybenzoic acid (1n) 55](#_Toc108689446)

[4-Methoxy-2-((3-(trifluoromethyl)benzyl)oxy)benzoic acid (1o) 55](#_Toc108689447)

[4-Methoxy-2-((3-methylbenzyl)oxy)benzoic acid (1p) 55](#_Toc108689448)

[4-Methoxy-2-((3-methoxybenzyl)oxy)benzoic acid (1q) 55](#_Toc108689449)

[2-((3,5-Dimethylbenzyl)oxy)-4-methoxybenzoic acid (1r) 55](#_Toc108689450)

[2-((3,5-Dimethoxybenzyl)oxy)-4-methoxybenzoic acid (1s) 56](#_Toc108689451)

[4-Methoxy-2-(naphthalen-1-ylmethoxy)benzoic acid (1t) 56](#_Toc108689452)

[2-(Benzyl(*tert*-butoxycarbonyl)amino)benzoic acid (1u) 56](#_Toc108689453)

[4-(Benzyloxy)-2',4'-difluoro-[1,1'-biphenyl]-3-carboxylic acid (1v) 56](#_Toc108689454)

[(*R*)-4-Methoxy-2-(1-phenylethoxy)benzoic acid (1w) 57](#_Toc108689455)

[*N*-(*tert*-Butyl)-*N*-(2,6-dimethylbenzyl)acrylamide (6b) 57](#_Toc108689456)

[*N*-(*tert*-Butyl)-*N*-(3,5-dimethoxybenzyl)acrylamide (6c) 57](#_Toc108689457)

[Spiro[chromane-3,1'-cyclohexane]-2',5'-dien-4-one (5a) 57](#_Toc108689458)

[7-Methoxyspiro[chromane-3,1'-cyclohexane]-2',5'-dien-4-one (5b) 57](#_Toc108689459)

[7-Methylspiro[chromane-3,1'-cyclohexane]-2',5'-dien-4-one (5c) 58](#_Toc108689460)

[8-Phenylspiro[chromane-3,1'-cyclohexane]-2',5'-dien-4-one (5d) 58](#_Toc108689461)

[*N*-(4-Oxospiro[chromane-3,1'-cyclohexane]-2',5'-dien-7-yl)acetamide (5e) 58](#_Toc108689462)

[8-Methoxyspiro[chromane-3,1'-cyclohexane]-2',5'-dien-4-one (5f) 58](#_Toc108689463)

[6-Methoxyspiro[chromane-3,1'-cyclohexane]-2',5'-dien-4-one (5g) 59](#_Toc108689464)

[5-Methoxyspiro[chromane-3,1'-cyclohexane]-2',5'-dien-4-one (5h) 59](#_Toc108689465)

[7-Chlorospiro[chromane-3,1'-cyclohexane]-2',5'-dien-4-one (5i) 59](#_Toc108689466)

[6-Acetylspiro[chromane-3,1'-cyclohexane]-2',5'-dien-4-one (5j) 59](#_Toc108689467)

[6-Bromo-7-methylspiro[chromane-3,1'-cyclohexane]-2',5'-dien-4-one (5k) 60](#_Toc108689468)

[2'-Fluoro-7-methoxyspiro[chromane-3,1'-cyclohexane]-2',5'-dien-4-one (5l) 60](#_Toc108689469)

[3'-Fluoro-7-methoxyspiro[chromane-3,1'-cyclohexane]-2',5'-dien-4-one (5m) 60](#_Toc108689470)

[3'-Chloro-7-methoxyspiro[chromane-3,1'-cyclohexane]-2',5'-dien-4-one (5n) 60](#_Toc108689471)

[7-Methoxy-3'-(trifluoromethyl)spiro[chromane-3,1'-cyclohexane]-2',5'-dien-4-one (5o) 61](#_Toc108689472)

[7-Methoxy-3'-methylspiro[chromane-3,1'-cyclohexane]-2',5'-dien-4-one (5p) 61](#_Toc108689473)

[3',7-Dimethoxyspiro[chromane-3,1'-cyclohexane]-2',5'-dien-4-one (5q) 61](#_Toc108689474)

[7-Methoxy-3',5'-dimethylspiro[chromane-3,1'-cyclohexane]-2',5'-dien-4-one (5r) 61](#_Toc108689475)

[3',5',7-Trimethoxyspiro[chromane-3,1'-cyclohexane]-2',5'-dien-4-one (5s) 62](#_Toc108689476)

[7-Methoxy-4'H-spiro[chromane-3,1'-naphthalen]-4-one (5t) 62](#_Toc108689477)

[*tert*-Butyl 4'-oxo-2'H-spiro[cyclohexane-1,3'-quinoline]-2,5-diene-1'(4'H)-carboxylate (5u) 62](#_Toc108689478)

[7-(2,4-Difluorophenyl)spiro[chromane-3,1'-cyclohexane]-2',5'-dien-4-one (5v) 62](#_Toc108689479)

[(*R*)-7-Methoxy-2-methylspiro[chromane-3,1'-cyclohexane]-2',5'-dien-4-one (5w) 63](#_Toc108689480)

[Spiro[chromane-3,1'-cyclohexane]-2',5'-dien-4-ol (5aa) 64](#_Toc108689481)

[4-Methylspiro[chromane-3,1'-cyclohexane]-2',5'-dien-4-ol (5ab) 65](#_Toc108689482)

[Spiro[chromane-3,1'-cyclohexan]-4-ol (5ac) 65](#_Toc108689483)

[Spiro[chromane-3,1'-cyclohexane] (5ad) 65](#_Toc108689484)

[3',7-Dimethoxyspiro[chromane-3,1'-cyclohexan]-3'-ene-4,5'-dione (5sa) 65](#_Toc108689485)

[1-(2-(Benzyloxy)phenyl)-3,3-diphenylpropan-1-one (10) 66](#_Toc108689486)

[2-(*tert*-Butyl)-4-(2-oxo-2-phenylethyl)-2-azaspiro[4.5]deca-6,9-dien-3-one (9a) 66](#_Toc108689487)

[2-(*tert*-Butyl)-4-(2-oxo-2-(p-tolyl)ethyl)-2-azaspiro[4.5]deca-6,9-dien-3-one (9b) 66](#_Toc108689488)

[2-(*tert*-Butyl)-4-(2-(4-methoxyphenyl)-2-oxoethyl)-2-azaspiro[4.5]deca-6,9-dien-3-one (9c) 66](#_Toc108689489)

[2-(*tert*-Butyl)-4-(2-(4-fluorophenyl)-2-oxoethyl)-2-azaspiro[4.5]deca-6,9-dien-3-one (9d) 67](#_Toc108689490)

[2-(*tert*-Butyl)-4-(2-(4-chlorophenyl)-2-oxoethyl)-2-azaspiro[4.5]deca-6,9-dien-3-one (9e) 67](#_Toc108689491)

[2-(*tert*-Butyl)-4-(2-(2,4-dimethoxyphenyl)-2-oxoethyl)-2-azaspiro[4.5]deca-6,9-dien-3-one (9f) 67](#_Toc108689492)

[2-(*tert*-Butyl)-4-(2-(3,4-dimethylphenyl)-2-oxoethyl)-2-azaspiro[4.5]deca-6,9-dien-3-one (9g) 68](#_Toc108689493)

[2-(*tert*-Butyl)-4-(2-(furan-2-yl)-2-oxoethyl)-2-azaspiro[4.5]deca-6,9-dien-3-one (9h) 68](#_Toc108689494)

[2-(*tert*-Butyl)-4-(2-(4-methoxyphenyl)-2-oxoethyl)-6,10-dimethyl-2-azaspiro[4.5]deca-6,9-dien-3-one (9i) 68](#_Toc108689495)

[2-(*tert*-Butyl)-7,9-dimethoxy-4-(2-(4-methoxyphenyl)-2-oxoethyl)-2-azaspiro[4.5]deca-6,9-dien-3-one (9j) 69](#_Toc108689496)

[4-(2-(6-(3-((3r,5r,7r)-Adamantan-1-yl)-4-methoxyphenyl)naphthalen-2-yl)-2-oxoethyl)-2-(*tert*-butyl)-2-azaspiro[4.5]deca-6,9-dien-3-one (9k) 69](#_Toc108689497)

[4. Supplementary Note 2 70](#_Toc108689498)

[4.1 NMR spectra of substrates and products 70](#_Toc108689499)

[5. Supplementary references 135](#_Toc108689500)

# 1. Supplementary methods

## 1.1 Materials and methods

All reagents were obtained from commercial sources and used without further purification. All solvents were purified and dried according to standard methods prior to use, unless stated otherwise. Other benzoic acid derivatives were prepared by using the reported procedure and purified through column chromatography respectively. Thin-layer chromatography (TLC) was performed using 60 mesh silica gel plates visualized with short-wavelength UV light (254 nm). Silica gel 60 (200-300 mesh) was used for column chromatography. A Bruker Ascend 400 spectrometer (400 MHz) or Bruker Avance DMX 500 (500 MHz) spectrometer was used for the recording of ^1^H NMR spectra, ^13^C{1H} NMR spectra and ^19^F NMR spectra. Proton chemical shifts are reported as δ values (ppm) relative to tetramethylsilane with residual undeuterated CHCl_3_ (δ 7.26), DMSO-*d_6_* (δ 2.50), MeCN-*d_3_* (δ 1.94) and methanol-*d_4_* (δ 3.31) as internal standards. ^13^C chemical shifts are reported as δ values (ppm) relative to tetramethylsilane with CDCl_3_ (δ 77.16 ppm), DMSO-*d_6_* (δ 39.52 ppm), MeCN-*d_3_* (δ 1.32 and 118.26 ppm) or methanol-*d_4_* (δ 49.0 ppm) as internal standards. Data for ^1^H NMR are reported as follows: chemical shift (δ, ppm), multiplicity (s = singlet, d = doublet, t = triplet, q = quartet, m = multiplet or unresolved, br = broad singlet, *J=* coupling constants in Hz, integration). HRMS measurements were performed on methanolic solutions of the compounds with Bruker maXis impact II micrOTOF spectrometer (direct injection, electrospray ionization, ESI). The photoreactions were carried out in 8 mL vials equipped with a stirring bar unless otherwise noted, and then were illuminated with 440 nm LED (40 W, Kessil PR160, set to maximum intensity) with continuous stirring. The fluorescence quenching studies were performed using a FS5 steady-state spectrofluorometer (Edinburgh Instruments).

## 1.2 General procedure for preparation of photocatalysts and substrates

1.2.1 Preparation of photocatalysts

Method A^1^

To a flame dried flask equipped with a magnetic stir bar, NaH (60% in oil, 0.32 g, 8.0 mmol) was added slowly a solution of diphenylamine (1.01 g, 6.0 mmol) tetrafluoroisophthalonitrile (0.20 g, 1.0 mmol) in dry DMF (10 mL) under an argon atmosphere at room temperature. The deprotonation was first performed at 50 °C for 1 h. After stirring at the same temperature for 4 h, water was added to the reaction to quench the excess of NaH. The resulting mixture was then concentrated and washed with water and EtOH to yield the crude product that was further purified by flash chromatography using silica gel to give the product (4DPAIPN). The characterization data of 4DPAIPN was in accordance with previous reports.^1^

Method B^2^

To a flame dried flask equipped with a magnetic stir bar, NaH (60% in oil, 0.60 g, 15.0 mmol) was added slowly a solution of diphenylamine (1.69 g, 10.0 mmol) in dry THF (40 mL) under argon atmosphere at room temperature. After 30 min, tetrafluoroisophthalonitrile (0.40 g, 2.0 mmol) was added. After stirring for 12 h at room temperature, water (2 mL) was added to the reaction mixture to quench the excess NaH. The resulting mixture was concentrated and washed by water and EtOH to yield the crude product and further purified by flash chromatography using silica gel to give the product (3DPAFIPN). The characterization data of 3DPAFIPN was in accordance with previous reports.^2,3^

1.2.2 Preparation of substrates

Method C^4^

To a solution of methyl salicylate derivative (5.0 mmol) and K_2_CO_3_ (10 mmol, 2 equiv.) in DMF (10 mL) benzyl bromide derivative (10 mmol, 2 equiv.) was slowly added at room temperature. Upon completion of addition, the reaction was stirred at room temperature for 12 h. Water was added and the reaction mixture was extracted 3 times with EtOAc. The combined organic layers were washed with brine and dried over sodium sulfate and concentrated under reduced pressure. The crude product was dissolved in MeOH/H_2_O (3:1, 20 mL), KOH (15 mmol, 3 equiv.) solution was added at room temperature and the reaction was then refluxed for 1 h. The mixture was adjusted to pH 2 with HCl (1 M) and extracted with EtOAc. The combined organic layers were dried over anhydrous Na_2_SO_4_, filtered and the organic solvent was evaporated in vacuo and the crude product was purified by flash chromatography using silica gel to give the corresponding product.

Method D^5^

To a solution of methyl salicylate derivative (5.0 mmol) and K_2_CO_3_ (12.5 mmol, 2.5 equiv.) in DMF (20 mL) benzyl bromide derivative (12.5 mmol, 2.5 equiv.) was added slowly at room temperature. Upon completion of addition, the reaction was stirred at room temperature for 12 h. Water was added and extracted 3 times with EtOAc. The combined organic layers were washed with brine and dried over sodium sulfate and concentrated under reduced pressure. The crude product was dissolved in MeOH/H_2_O (4:1, 20 mL), KOH (20 mmol, 4 equiv.) solution was added at room temperature and the reaction was refluxed for 1 h. The mixture was adjusted to pH 2 with HCl (1 M) and extracted with EtOAc. The combined organic layers were dried over anhydrous Na_2_SO_4,_ filtered and the organic solvent was evaporated in vacuo and was purified by flash chromatography using silica gel to give the corresponding product.

Method E^6^

To a stirred solution of sodium carbonate (5 mmol) in water (5.0 mL) was added 2-(benzylamino)benzoic acid (5 mmol) in small portions with continuous stirring at room temperature. Boc anhydride (5 mmol) was added, and the reaction mixture was stirred at room temperature for another 30 min. The reaction mixture was then acidified with aqueous HCl, resulting in the formation of a precipitate that was collected and dried to yield product **1u**.

Method F^7^

Methyl 2-hydroxy-4-methoxybenzoate (6 mmol, 1 equiv.), triphenylphosphine (8.4 mmol, 1.4 equiv.), and (*R*)-1-phenylethanol (8.4 mmol, 1.36 equiv.) were dissolved in dry THF (50 mL) under N_2_. The reaction mixture was stirred for 5 min and then placed in an ice bath and cooled to 0 °C. DIAD (8.4 mmol, 14 equiv.) was added dropwise over 30 min. After 15 min, the ice bath was removed, and the mixture was stirred overnight at room temperature. THF was removed under reduced pressure, and the residue was dissolved in a mixture of diethyl ether and hexanes. After a few minutes, a white precipitate was formed and was removed by filtration. The filtrate was evaporated to dryness and the crude product was purified by flash chromatography using silica gel to yield the intermediate product. The intermediate product was dissolved in MeOH/H_2_O (4:1, 24 mL), KOH (24 mmol, 4 equiv.) solution was added at room temperature and the reaction mixture was refluxed for 1 h. The mixture was adjusted to pH 2 with HCl (1 M) and extracted with EtOAc. The combined organic layers were dried over anhydrous Na_2_SO_4_, filtered and the organic solvent was evaporated in vacuo and purified by flash chromatography using silica gel to give the corresponding product **1w**.

Method G^8,9^

2,6-dimethylbenzaldehyde or 3,5-dimethoxybenzaldehyde (5.0 mmol, 1.0 equiv.) and *tert*-butyl amine (6 mmol, 1.2 equiv.) were stirred in MeOH (25 mL) for 2 hours. NaBH_4_ (7.5 mmol, 1.5 equiv) was added and the reaction mixture was stirred for an additional 1 hour. The organic solvent was evaporated in vacuo, and the residue was purified via flash chromatography using silica to give the corresponding product **S1** and **S2**.

To a flame dried flask equipped with a magnetic stir bar, were added *tert*-butylamine derivatives (2.2 equiv.), triethylamine (2 equiv.) and DMAP (0.1 equiv.) in CH_2_Cl_2_ under argon atmosphere at room temperature. After stirring at 0 °C under a nitrogen atmosphere for 15 min, a solution of acryloyl chloride (1 equiv.) in CH_2_Cl_2_ was slowly added dropwise at 0 °C. After stirring for 14 h at room temperature, HCl (2 M) was added, and the layers were separated. The organic layer was washed with HCl (2M) and water, and the organic phase was dried with MgSO_4_ and concentrated under reduced pressure to afford the target product **6a**–**6c**.

#

# 2. Supplementary Result and discussion

## 2.1 Optimization of reaction conditions and general procedures

**Supplementary Table 1.** The optimization of solvent, photocatalyst and base for the intramolecular spirocyclization.*^a^*

| **Entry** | **PC (1 mol%)** | **Base (1 equiv)** | **Solvent** | **Yield** |
| --- | --- | --- | --- | --- |
| 1*^b^* | 4DPAIPN | Na_2_CO_3_ | CH_3_CN | trace |
| 2*^b^* | 4DPAIPN | Na_2_CO_3_ | CH_3_COCH_3_ | 0 |
| 3*^b^* | 4DPAIPN | Na_2_CO_3_ | MeOH | 0 |
| 4*^b^* | 4DPAIPN | Na_2_CO_3_ | EtOAc | 0 |
| 5*^b^* | 4DPAIPN | Na_2_CO_3_ | ClCH_2_CH_2_Cl | 0 |
| 6*^b^* | 4DPAIPN | Na_2_CO_3_ | DMSO | 0 |
| 7*^b^* | 4DPAIPN | Na_2_CO_3_ | CH_3_CN/H_2_O (75:25 vol%) | 3 |
| 8*^b^* | 3DPAFIPN | Na_2_CO_3_ | CH_3_CN/H_2_O (75:25 vol%) | 11 |
| 9*^b^* | [Ir(dFCF_3_ppy)_2_(dtbbpy)]PF_6_ | Na_2_CO_3_ | CH_3_CN/H_2_O (75:25 vol%) | 40 |
| 10*^b^* | [Ir(dtbbpy)(ppy)_2_]PF_6_ | Na_2_CO_3_ | CH_3_CN/H_2_O (75:25 vol%) | 11 |
| 11*^c^* | [Ir(dF(Me)ppy)_2_(dtbbpy)]PF_6_ | Na_2_CO_3_ | CH_3_CN/H_2_O (75:25 vol%) | 37 |
| 12*^c^* | [Ir(dFCF_3_ppy)_2_(dtbbpy)]PF_6_ | K_3_PO_4_ | CH_3_CN/H_2_O (75:25 vol%) | 58 |
| 13*^c^* | [Ir(dFCF_3_ppy)_2_(dtbbpy)]PF_6_ | Na_2_CO_3_ | CH_3_CN/H_2_O (75:25 vol%) | 40 |
| 14*^c^* | [Ir(dFCF_3_ppy)_2_(dtbbpy)]PF_6_ | DMAP | CH_3_CN/H_2_O (75:25 vol%) | 8 |
| 15*^c^* | [Ir(dFCF_3_ppy)_2_(dtbbpy)]PF_6_ | Cs_2_CO_3_ | CH_3_CN/H_2_O (75:25 vol%) | 36 |
| 16*^c^* | [Ir(dFCF_3_ppy)_2_(dtbbpy)]PF_6_ | NaH_2_PO_4_ | CH_3_CN/H_2_O (75:25 vol%) | 12 |
| 17*^c^* | [Ir(dFCF_3_ppy)_2_(dtbbpy)]PF_6_ | NaHCO_3_ | CH_3_CN/H_2_O (75:25 vol%) | 38 |

*^a^* Reaction conditions: **1a** (0.2 mmol, 1 equiv.), triphenylphosphine (0.2 mmol, 1 equiv.), base (0.2 mmol, 1 equiv.), photocatalyst (l mol%), solvent (4 mL), N_2_, blue LEDs (440 nm), 16 h, room temperature. *^b^* Yields were determined by ^1^H NMR using 4-cyanopyridine as the internal standard. *^c^* Yields were determined by ^1^H NMR using 4-nitrobenzonitrile as the internal standard.

**Supplementary Table 2.** The optimization of the amount of water and base for the intramolecular spirocyclization.*^a^*

| **Entry** | **1a (mmol)** | **K_3_PO_4_ (mmol)** | **Solvent** | **Yield*^b^*** |
| --- | --- | --- | --- | --- |
| 1 | 0.3 | 0.3 | CH_3_CN/H_2_O (90:10 vol%) | 63 |
| 2 | 0.3 | 0.3 | CH_3_CN/H_2_O (85:15 vol%) | 66 |
| 3 | 0.3 | 0.3 | CH_3_CN/H_2_O (75:25 vol%) | 61 |
| 4 | 0.3 | 0.3 | CH_3_CN/H_2_O (65:35 vol%) | 52 |
| 5 | 0.3 | 0.3 | CH_3_CN/H_2_O (50:50 vol%) | 12 |
| 6 | 0.3 | 0.15 | CH_3_CN/H_2_O (85:15 vol%) | 40 |
| 7 | 0.3 | 0.45 | CH_3_CN/H_2_O (85:15 vol%) | 43 |

*^a^* Reaction conditions: **1a** (0.3 mmol, 1 equiv.), triphenylphosphine (0.6 mmol, 2 equiv.), K_3_PO_4_, [Ir(dFCF_3_ppy)_2_(dtbbpy)]PF_6_, (1 mol%), solvent (6 mL), N_2_, blue LEDs (440nm), 16 h, room temperature. *^b^* Yields were determined by ^1^H NMR using 4-nitrobenzonitrile as the internal standard.

**Supplementary Table 3.** The optimization of the amount of water and base for the intramolecular spirocyclization.*^a^*

| **Entry** | **1a (mmol)** | **PPh_3_ (mmol)** | **Time (hours)** | **Yield*^b^*** |
| --- | --- | --- | --- | --- |
| 1 | 0.3 | 0.3 | 16 | 56 |
| 2 | 0.3 | 0.36 | 16 | 56 |
| 3 | 0.3 | 0.45 | 16 | 61 |
| 4 | 0.3 | 0.6 | 16 | 66 |
| 5 | 0.3 | 0.45 | 24 | 62 |
| 6 | 0.3 | 0.45 | 36 | 66 |

*^a^* Reaction conditions: **1a** (0.3 mmol, 1 equiv.), triphenylphosphine, K_3_PO_4_ (0.3 mmol, 1 equiv.) [Ir(dFCF_3_ppy)_2_(dtbbpy)]PF_6_ (1 mol%), CH_3_CN/H_2_O (85:15 vol%, 6 mL), blue LEDs (440 nm), N_2_, temperature. *^b^* Yields were determined by ^1^H NMR using 4-nitrobenzonitrile as the internal standard.

**Supplementary Table 4.** The optimization of base and solvent for the intramolecular spirocyclization**.***^a^*

| **Entry** | **Base (1 equiv.)** | **Solvent** | **Yield** |
| --- | --- | --- | --- |
| 1*^b^* | K_2_CO_3_ | CH_3_CN/H_2_O (85:15 vol%) | 31 |
| 2*^b^* | K_3_PO_4_ | CH_3_CN/H_2_O (85:15 vol%) | 33 |
| 3*^b^* | Cs_2_CO_3_ | CH_3_CN/H_2_O (85:15 vol%) | 32 |
| 4*^b^* | K_2_HPO4 | CH_3_CN/H_2_O (85:15 vol%) | 12 |
| 5*^b^* | DMAP | CH_3_CN/H_2_O (85:15 vol%) | 10 |
| 6 | K_2_CO_3_ | CH_3_CN/H_2_O (85:15 vol%) | 30 |
| 7 | K_3_PO_4_ | CH_3_CN/H_2_O (85:15 vol%) | 54 |
| 8 | Cs_2_CO_3_ | CH_3_CN/H_2_O (85:15 vol%) | 40 |
| 9 | K_3_PO_4_ | CH_3_CN/H_2_O (75:25 vol%) | 46 |
| 10 | K_3_PO_4_ | CH_3_CN/H_2_O (50:50 vol%) | 9 |
| 11 | K_3_PO_4_ | CH_3_CN/H_2_O (95:5 vol%) | 27 |
| 12 | K_3_PO_4_ | DMSO/H_2_O (85:15 vol%) | trace |
| 13 | K_3_PO_4_ | THF/H_2_O (85:15 vol%) | trace |
| 14 | K_3_PO_4_ | DMF/H_2_O (85:15 vol%) | trace |

*^a^* Reaction conditions: Benzoic acid (0.4 mmol, 2 equiv.), *N*-benzyl-*N*-(*tert*-butyl)acrylamide **6a** (0.2 mmol, 1 equiv.), triphenylphosphine (0.4 mmol, 1 equiv.), base (0.4 mmol, 2 equiv.), photocatalyst (1 mol%), solvent (4 mL), N_2_, blue LEDs (440 nm), 36 h, room temperature. Yields were determined by ^1^H NMR using 4-nitrobenzonitrile as the internal standard. *^b^* Base (0.2 mmol, 1 equiv.).

**Supplementary Table 5.** Additional optimizations for the intermolecular spirocyclization.*^a^*

| **Entry** | **benzoic acid (mmol)** | **6a (mmol)** | **PPh_3_ (mmol)** | **K_3_PO_4_ (mmol)** | **Yield** |
| --- | --- | --- | --- | --- | --- |
| 1 | 0.4 | 0.2 | 0.4 | 0.4 | 63 |
| 2 | 0.6 | 0.3 | 0.6 | 0.4 | 49 |
| 3*^b^* | 0.4 | 0.2 | 0.4 | 0.4 | 45 |
| 4 | 0.3 | 0.2 | 0.4 | 0.3 | 41 |
| 5 | 0.6 | 0.2 | 0.6 | 0.6 | 53 |
| 6*^c^* | 0.4 | 0.2 | 0.4 | 0.4 | 50 |
| 7*^c^* | 0.6 | 0.2 | 0.6 | 0.6 | 52 |
| 8 | 0.2 | 0.4 | 0.4 | 0.2 | 69 |
| 9 | 0.2 | 0.3 | 0.4 | 0.2 | 51 |

*^a^* Reaction conditions: benzoic acid, *N*-benzyl-*N*-(*tert*-butyl)acrylamide **6a**, triphenylphosphine, K_3_PO_4_, photocatalyst (1 mol%), solvent (4 mL), N_2_, blue LEDs (440 nm), 48 h, room temperature. The yields were determined by ^1^H NMR using 4-nitrobenzonitrile as the internal standard. *^b^* CH_3_CN/H_2_O (85:15 vol%, 2 mL). *^c^* CH_3_CN/H_2_O (85:15 vol%, 6 mL).

General Procedure A for the intramolecular radical dearomative spirocyclization

A 10 mL Pyrex tube equipped with a magnetic stir bar was charged with 2-(benzyloxy)benzoic acid **1a** (68.4 mg, 0.3 mmol), triphenylphosphine (117.9 mg, 0.45 mmol), and [Ir(dFCF_3_ppy)_2_(dtbbpy)]PF_6_ (3.4 mg, 0.003 mmol) in CH_3_CN/H_2_O (85:15 vol%, 6 mL). The mixture was strictly deaerated with N_2_, and then further irradiated by blue LEDs (*λ* = 440 nm) for 36 hours at room temperature. When the reaction was finished, the residue was purified by chromatography on silica gel to yield product **5a**.

General Procedure B for the gram-scale reaction

A 100 mL round-bottom flask equipped with a magnetic stir bar was charged with 2-(benzyloxy)benzoic acid **1a** (1.14 g, 5 mmol), triphenylphosphine (1.97 g, 7.5 mmol), and [Ir(dFCF_3_ppy)_2_(dtbbpy)]PF_6_ (56 mg, 0.005 mmol) in CH_3_CN/H_2_O (85:15 vol%, 100 mL). The mixture was carefully deaerated with N_2_, and then the mixture was irradiated under stirring by blue LEDs (*λ* = 440 nm) for 60 hours at room temperature. The solvent was evaporated, and the residue was purified by flash chromatography using silica gel to yield product **5a**.

General Procedure C for the intermolecular radical dearomative spirocyclization

A 10 mL Pyrex tube equipped with a magnetic stir bar was charged with benzoic acid (30.4 mg, 0.2 mmol), *N*-benzyl-*N*-(*tert*-butyl)acrylamide **6a** (86.8 mg, 0.4 mmol), triphenylphosphine (104.8 mg, 0.4 mmol), and [Ir(dFCF_3_ppy)_2_(dtbbpy)]PF_6_ (2.2 mg, 0.002 mmol) in CH_3_CN/H_2_O (85:15 vol%, 4 mL). The mixture was carefully deaerated with N_2_, and then the mixture was irradiated by blue LEDs (*λ* = 440 nm) for 48 hours at room temperature. The solvent was evaporated, and the residue was purified by flash chromatography using silica gel to yield product **9a**.

**Supplementary Table 6.** Unsuccessful substrates for the intra- and intermolecular spirocyclization

## 2.2 Fluorescence quenching studies

Fluorescence quenching studies were performed on FS5 steady-state spectrofluorometer (Edinburgh Instruments) using 10 × 10 mm quartz cuvettes. All measurements were performed under Ar atmosphere at room temperature (ca. 20 °C), using CH_3_CN/H_2_O (85:15 vol%) as the solvent (HPLC-grade MeCN, MilliQ-grade water). The emission spectra were collected at 440–750 nm with excitation at 420 nm.

For the quenching experiments with PPh_3_ as the quencher (Figure 3), a series of solutions (10 solutions, 3 mL each) of the Ir-photocatalyst ([Ir(dF(CF_3_)ppy)_2_(dtbbpy)](PF_6_), 15 μM) and PPh_3_ (0–10 mM) were prepared by mixing appropriate volumes of CH_3_CN/H_2_O (85:15 vol%) (0–2.67 mL) with stock solutions of Ir-photocatalyst (0.3 mL, 0.15 mM) and PPh_3_ (0.027–2.7 mL, 11.1 mM) in CH_3_CN/H_2_O (85:15 vol%). The Stern-Volmer quenching constant (*K*_SV_ = 965 M^−1^) was calculated as the slope of linear regression in the plot of normalized emission intensity (*I*_0_/*I*) at 475 nm vs. concentration of the quencher.

The quenching experiments with starting material **1a** (Figure S1), starting material **1a** deprotonated by K_3_PO_4_ (Figure S2), product **5a** (Figure S3), and Ph_3_PO (Figure S4) as quenchers were performed in analogous manner to the quenching experiments with PPh_3_. The emission spectra were recorded for three concentrations of each quencher (0 mM, 5 mM, 10 mM) and fixed concentration of the Ir-photocatalyst (15 μM). For the quenching experiments with deprotonated **1a**, **1a** and K_3_PO_4_ were mixed as stock solutions in MeCN and water, respectively, due to the low solubility of K_3_PO_4_ in CH_3_CN/H_2_O (85:15 vol%). The molar ratio between **1a** and K_3_PO_4_ was maintained at 1:0.9 to prevent apparent degradation of the photocatalyst by K_3_PO_4_. The following Stern-Volmer quenching constant were calculated as: *K*_SV_ = 11 M^−1^ for starting material **1a**, *K*_SV_ = 13 M^−1^ for starting material **1a** deprotonated by K_3_PO_4_, *K*_SV_ = 50 M^−1^ for product **5a**, *K*_SV_ = 42 M^−1^ for Ph_3_PO.

|  |  |
| --- | --- |

**Supplementary Figure 1.** Fluorescence quenching study of 15 µM Ir-photocatalyst by starting material **1a** (0–10 mM).

|  |  |
| --- | --- |

**Supplementary Figure 2.** Fluorescence quenching study of 15 µM Ir-photocatalyst by starting material **1a** deprotonated by K_3_PO_4_ (0–10 mM).

|  |  |
| --- | --- |

**Supplementary Figure 3.** Fluorescence quenching study of 15 µM Ir-photocatalyst by product **5a** (0–10 mM).

|  |  |
| --- | --- |

**Supplementary Figure 4.** Fluorescence quenching study of 15 µM Ir-photocatalyst by Ph_3_PO (0–10 mM).

## 2.3 Computational studies

2.3.1 Computational details

All stationary points were optimized, first at the B3LYP/6-311+G(d,p) level of theory and further re-optimization at the B3LYP/6-311+G(d,p) level of theory, as implemented in Gaussian 16 Rev D.01. In the optimizations, the Grimme correction for dispersion (D3) was used in combination with the Conductor-like Polarizable Continuum Model (CPCM) using the parameters for acetonitrile and the default Unified Force Field radii (UFF) as implemented in Gaussian 16 Rev D.01. All geometries were characterized as minima or saddle points on the potential-energy surface (PES) by using the sign of the eigenvalues of the force-constant matrix obtained from a frequency calculation. Transition states with one imaginary frequency were confirmed to describe the correct movement on the PES by mode analysis and by intrinsic reaction coordinate (IRC) calculations connecting the correct reactants and products.

2.3.2 Complete Gaussian 16 reference

Gaussian 16, Revision B.01, Frisch, M. J.; Trucks, G. W.; Schlegel, H. B.; Scuseria, G. E.; Robb, M. A.; Cheeseman, J. R.; Scalmani, G.; Barone, V.; Petersson, G. A.; Nakatsuji, H.; Li, X.; Caricato, M.; Marenich, A. V.; Bloino, J.; Janesko, B. G.; Gomperts, R.; Mennucci, B.; Hratchian, H. P.; Ortiz, J. V.; Izmaylov, A. F.; Sonnenberg, J. L.; Williams-Young, D.; Ding, F.; Lipparini, F.; Egidi, F.; Goings, J.; Peng, B.; Petrone, A.; Henderson, T.; Ranasinghe, D.; Zakrzewski, V. G.; Gao, J.; Rega, N.; Zheng, G.; Liang, W.; Hada, M.; Ehara, M.; Toyota, K.; Fukuda, R.; Hasegawa, J.; Ishida, M.; Nakajima, T.; Honda, Y.; Kitao, O.; Nakai, H.; Vreven, T.; Throssell, K.; Montgomery, J. A., Jr.; Peralta, J. E.; Ogliaro, F.; Bearpark, M. J.; Heyd, J. J.; Brothers, E. N.; Kudin, K. N.; Staroverov, V. N.; Keith, T. A.; Kobayashi, R.; Normand, J.; Raghavachari, K.; Rendell, A. P.; Burant, J. C.; Iyengar, S. S.; Tomasi, J.; Cossi, M.; Millam, J. M.; Klene, M.; Adamo, C.; Cammi, R.; Ochterski, J. W.; Martin, R. L.; Morokuma, K.; Farkas, O.; Foresman, J. B.; Fox, D. J. Gaussian, Inc., Wallingford CT, **2016**.

**Supplementary Table 7.** Energies, enthalpies, and Gibbs free energies for intramolecular spirocyclization.

| **Species** | **Energy** | **Enthalpy** | **Gibbs free energy** | **∆E** | **∆H** | **∆G** |
| --- | --- | --- | --- | --- | --- | --- |
|  | (Hartree) | | | (kcal mol^–1^) | | |
| **PPh_3_^+•^** | -1036.331036 | -1036.040916 | -1036.104093 |  |  |  |
| **PF_6_^-^** | -940.9832706 | -940.95813 | -940.995775 |  |  |  |
| **Ir(II)** | -2943.823575 | -2943.095289 | -2943.239056 |  |  |  |
| **K_3_PO_4_** | -2442.600688 | -2442.572959 | -2442.624978 |  |  |  |
| **1a** | -766.6493378 | -766.405858 | -766.465203 | 0.00 | 0.00 | 0.00 |
| **1a-K** | -1366.085884 | -1365.852699 | -1365.918431 | -15.98 | -16.74 | -18.44 |
| **K_2_HPO_4_** | -1843.189601 | -1843.152802 | -1843.201142 |  |  |  |
| **K^+^** | -599.8839348 | -599.881574 | -599.899111 |  |  |  |
| **1a-PPh_3_** | -1802.545034 | -1802.022845 | -1802.123646 | -39.52 | -40.26 | -0.15 |
| **Int-2** | -1802.532587 | -1802.011868 | -1802.109462 | -31.71 | -33.38 | 8.75 |
| **TS1** | -1802.526604 | -1802.007084 | -1802.105374 | -27.95 | -30.37 | 11.32 |
| **Int-3i** | -1802.550132 | -1802.028292 | -1802.128041 | -42.72 | -43.68 | -2.90 |
| **O=PPh_3_** | -1111.823604 | -1111.528578 | -1111.595865 |  |  |  |
| **Int-3ii** | -690.7106938 | -690.485933 | -690.544039 | -32.78 | -35.03 | -10.35 |
| **Int-3iii** | -690.7071127 | -690.482669 | -690.539559 | -30.53 | -32.99 | -7.54 |
| **TS2** | -690.6956918 | -690.472988 | -690.525421 | -23.37 | -26.91 | 1.33 |
| **Int-4** | -690.7157602 | -690.491632 | -690.544089 | -35.96 | -38.61 | -10.38 |
| **Ir(III)^+^•PF_6_^-^** | -3884.709609 | -3883.950053 | -3884.113719 | -47.10 | -70.09 | -7.09 |
| **Ir-III^+^** | -2943.711827 | -2942.979993 | -2943.122543 | -37.99 | -39.71 | -9.98 |
| **Int-4i** | -690.8307519 | -690.608677 | -690.659958 |  |  |  |
| **5a** | -691.3438315 | -691.106586 | -691.158946 | -63.24 | -62.61 | -24.86 |

**Supplementary Figure 5.** Gibbs free energy diagram for intramolecular spirocyclization.

**Supplementary Table 8.** Energies, enthalpies, and Gibbs free energies for intermolecular spirocyclization.

| **Species** | **Energy** | **Enthalpy** | **Gibbs free energy** | **∆E** | **∆H** | **∆G** |
| --- | --- | --- | --- | --- | --- | --- |
|  | (Hartree) | | | (kcal mol^–1^) | | |
| **PPh_3_^+•^** | -1036.331036 | -1036.040916 | -1036.104093 |  |  |  |
| **PF_6_^-^** | -940.9832706 | -940.95813 | -940.995775 |  |  |  |
| **Ir(II)** | -2943.823575 | -2943.095289 | -2943.239056 |  |  |  |
| **K_3_PO_4_** | -2442.600688 | -2442.572959 | -2442.624978 |  |  |  |
| **6a** | -675.1250574 | -674.807308 | -674.868766 |  |  |  |
| **PhCOOH** | -420.9653934 | -420.842325 | -420.882644 | 17.40 | 18.10 | 20.53 |
| **PhCOO^-^K^+^** | -1020.404201 | -1020.291329 | -1020.33919 | 0.00 | 0.00 | 0.00 |
| **K_2_HPO_4_** | -1843.189601 | -1843.152802 | -1843.201142 |  |  |  |
| **K^+^** | -599.8839348 | -599.881574 | -599.899111 |  |  |  |
| **PhCOO^–^PPh_3_^+•^** | -1456.860377 | -1456.458522 | -1456.546778 | -5.69 | -4.93 | -1.63 |
| **Int-2’** | -1456.852036 | -1456.451501 | -1456.534577 | -0.46 | -0.52 | 6.03 |
| **TS3** | -1456.847625 | -1456.448328 | -1456.532772 | 2.31 | 1.47 | 7.16 |
| **Int-3´i** | -1456.864537 | -1456.462867 | -1456.54995 | -8.30 | -7.65 | -3.62 |
| **O=PPh_3_** | -1111.823604 | -1111.528578 | -1111.595865 |  |  |  |
| **Int-3´ii** | -345.0287725 | -344.924362 | -344.962952 | -0.67 | -1.43 | -9.19 |
| **Int-3’iii** | -1020.163677 | -1019.739424 | -1019.823403 | -6.85 | -6.29 | -3.97 |
| **TS4’** | -1020.157405 | -1019.734456 | -1019.81378 | -2.91 | -3.17 | 2.07 |
| **7a** | -1020.197104 | -1019.771312 | -1019.848548 | -27.8 | -26.3 | -19.8 |
| **TS5’** | -1020.176718 | -1019.752724 | -1019.828524 | -15.03 | -14.64 | -7.18 |
| **8ai** | -1020.199100 | -1019.773413 | -1019.850329 | -29.08 | -27.62 | -20.86 |
| **Ir-III^+^** | -2943.711827 | -2942.979993 | -2943.122543 | -35.93 | -32.10 | -13.67 |
| **8aii** | -1020.307265 | -1019.883921 | -1019.959969 | -26.83 | -24.62 | -16.55 |
| **9a** | -1020.826873 | -1020.388092 | -1020.464671 | -56.17 | -51.44 | -35.02 |

**Supplementary Figure 6.** Gibbs free energy diagram for intermolecular spirocyclization.

2.3.3 XYZ-coordinates for optimized molecules

**1a**

0 1

C 1.957924 -1.860805 0.002813

C 1.424734 -0.570128 0.001838

C 2.281039 0.556110 0.000628

C 3.665198 0.339019 0.000502

C 4.199604 -0.942404 0.001478

C 3.338939 -2.039530 0.002621

H 1.308382 -2.724325 0.003758

H 4.312876 1.206278 -0.000415

H 5.272849 -1.085792 0.001340

H 3.739193 -3.046708 0.003392

C 1.813405 1.980212 -0.000612

O 0.491138 2.219432 -0.000862

O 0.082056 -0.322408 0.002093

C -0.883090 -1.411556 0.001919

H -0.721628 -2.020052 0.894585

H -0.720820 -2.020473 -0.890312

C -4.748650 0.468054 -0.000228

C -4.123909 0.154782 -1.208193

C -2.876898 -0.468270 -1.206466

C -2.246203 -0.783265 0.001172

C -2.877564 -0.466894 1.208107

C -4.124564 0.156175 1.208439

H -5.719481 0.950446 -0.000769

H -4.607643 0.393001 -2.148502

H -2.391011 -0.712185 -2.145271

H -2.392200 -0.709759 2.147456

H -4.608809 0.395476 2.148209

O 2.584833 2.919552 -0.001497

H -0.004168 1.373032 -0.000020

**K_3_PO_4_**

0 1

P 0.003099 1.059540 0.616361

O -0.003406 -0.523278 0.537268

K 2.379841 -0.793549 -0.579791

O 1.273199 1.576609 -0.177675

O -1.286325 1.585503 -0.140622

K -2.378388 -0.788924 -0.586784

O 0.026567 1.548347 2.093900

K -0.014226 3.097214 -1.909672

**K_2_HPO_4_**

0 1

P -0.000221 0.805221 -0.059486

O -0.000239 -0.549497 0.690253

K 2.486507 -1.291039 -0.094880

O 1.299646 1.041668 -0.849502

O -1.300569 1.042443 -0.848461

K -2.485727 -1.292459 -0.097257

O 0.000663 1.926625 1.207422

H -0.000189 2.824262 0.856261

**1a–K**

0 1

C 2.335876 -2.014977 0.000000

C 1.692648 -0.775741 0.000000

C 2.441827 0.413833 0.000000

C 3.831295 0.339196 0.000000

C 4.485387 -0.896861 0.000000

C 3.733799 -2.067513 0.000000

H 1.771338 -2.937418 0.000000

H 4.409179 1.257527 0.000000

H 5.568358 -0.939417 0.000000

H 4.225580 -3.033658 0.000000

C 1.703579 1.741235 0.000000

O 1.397072 2.224290 1.122095

O 0.327915 -0.612761 0.000000

C -0.511547 -1.786807 0.000000

H -0.299023 -2.386624 0.890401

H -0.299023 -2.386624 -0.890401

C -4.547465 -0.289710 0.000000

C -3.897296 -0.546570 -1.208277

C -2.598270 -1.054885 -1.207028

C -1.937387 -1.309588 0.000000

C -2.598270 -1.054885 1.207028

C -3.897296 -0.546570 1.208277

H -5.556833 0.105591 0.000000

H -4.400241 -0.352010 -2.148570

H -2.091532 -1.250769 -2.146069

H -2.091532 -1.250769 2.146069

H -4.400241 -0.352010 2.148570

O 1.397072 2.224290 -1.122095

K -1.216931 2.207704 0.000000

**PPh_3_+•**

1 2

P -1.578285 -0.176817 0.074073

C -2.294915 2.917931 -3.214759

C -2.322859 3.323086 -1.878654

C -2.106515 2.400498 -0.862295

C -1.870660 1.053104 -1.191016

C -1.827662 0.648696 -2.539736

C -2.049098 1.582489 -3.543202

H -2.462673 3.643974 -4.001329

H -2.520209 4.358099 -1.627548

H -2.142765 2.717540 0.171809

H -1.623772 -0.383553 -2.798623

H -2.022742 1.271919 -4.580390

C -4.447979 -3.734564 -0.046878

C -4.700779 -2.626176 -0.857872

C -3.842280 -1.533651 -0.838637

C -2.722755 -1.549710 0.013218

C -2.460900 -2.674603 0.819802

C -3.330495 -3.756659 0.791225

H -5.121259 -4.583199 -0.069325

H -5.571895 -2.609856 -1.501401

H -4.050895 -0.671828 -1.459023

H -1.587681 -2.701158 1.460820

H -3.133866 -4.619538 1.415467

C -1.003413 1.622110 4.238200

C -2.014374 0.680298 4.035516

C -2.207244 0.119078 2.778969

C -1.382453 0.516615 1.710891

C -0.353477 1.455586 1.920194

C -0.176172 2.009286 3.181104

H -0.858322 2.053474 5.221483

H -2.657994 0.387326 4.855988

H -3.000826 -0.600350 2.624680

H 0.298880 1.747849 1.105896

H 0.610536 2.735968 3.342087

**1-PPh_3_**

0 2

C 4.776633 -1.675804 0.030288

C 3.413782 -1.396272 -0.093701

C 2.504887 -2.432792 -0.377436

C 2.974850 -3.733362 -0.532659

C 4.334905 -4.021874 -0.394774

C 5.227793 -2.990387 -0.116009

H 5.484917 -0.885743 0.241975

 H 2.270478 -4.524707 -0.764829

H 4.689770 -5.039060 -0.510414

H 6.286502 -3.199542 -0.012440

C 1.039605 -2.095386 -0.553995

O 0.522767 -2.156352 -1.676517

O 2.866258 -0.153811 0.025427

C 3.710459 1.009259 0.006230

H 4.384355 0.951129 -0.856504

H 4.313755 1.057913 0.917818

C 1.010344 4.350412 -0.293895

C 1.895423 4.270314 0.779785

C 2.780496 3.194143 0.878028

C 2.793907 2.199520 -0.101106

C 1.908659 2.292406 -1.182148

C 1.018361 3.357373 -1.276458

H 0.309830 5.174987 -0.361740

H 1.887820 5.033533 1.549694

H 3.451682 3.122378 1.727302

H 1.901358 1.511527 -1.934237

H 0.320054 3.404261 -2.103095

O 0.450410 -1.740799 0.534332

P -1.319127 -0.352447 0.039546

C -2.280112 2.807821 -3.183657

C -2.615012 3.075875 -1.855827

C -2.338004 2.142220 -0.860115

C -1.728776 0.924954 -1.196850

C -1.380024 0.662869 -2.530494

C -1.662249 1.601668 -3.519122

H -2.492245 3.540450 -3.953776

H -3.090864 4.013640 -1.593166

H -2.599056 2.357780 0.168354

H -0.881094 -0.267144 -2.779887

H -1.390909 1.397199 -4.548296

C -4.661815 -3.527421 0.000812

C -4.853497 -2.313216 -0.658357

C -3.849026 -1.347592 -0.655964

C -2.639331 -1.597016 0.008665

C -2.444558 -2.824728 0.659691

C -3.457424 -3.779834 0.659675

H -5.445748 -4.275989 -0.002463

H -5.786123 -2.113732 -1.173354

H -4.009457 -0.405728 -1.165787

H -1.501646 -3.027796 1.150653

H -3.302796 -4.724796 1.167506

C -1.095755 1.717873 4.146255

C -1.985067 0.658216 3.967734

C -2.083626 0.024521 2.729855

C -1.288589 0.455794 1.660049

C -0.387076 1.516394 1.845748

C -0.298726 2.146214 3.082452

H -1.023017 2.208124 5.110245

H -2.607385 0.325314 4.790368

H -2.782093 -0.792787 2.599121

H 0.238736 1.852627 1.028482

H 0.395963 2.967580 3.213131

**Int-2**

0 2

 C 2.386394 3.506635 -0.573281

C 1.396937 2.540828 -0.641508

C 0.050418 2.879883 -1.005389

C -0.196679 4.275274 -1.247349

C 0.805562 5.224013 -1.177365

C 2.120667 4.856865 -0.849081

H 3.380683 3.187649 -0.277853

H -1.207247 4.568141 -1.503353

H 0.567477 6.263683 -1.378186

H 2.910432 5.595804 -0.788520

C -1.058273 2.005700 -1.142772

O -2.259568 2.286021 -1.300918

O 1.728952 1.252310 -0.249633

C 2.392161 0.445129 -1.227910

H 2.915939 1.096810 -1.937002

H 1.642958 -0.125500 -1.787428

C 5.277091 -2.219303 0.562073

C 4.892486 -2.374962 -0.771042

C 3.948498 -1.517607 -1.330103

C 3.382747 -0.487056 -0.571656

C 3.772374 -0.334588 0.759712

C 4.712425 -1.198154 1.324231

H 6.004579 -2.892269 1.002042

H 5.317728 -3.172460 -1.370349

H 3.639137 -1.656167 -2.361345

H 3.327614 0.453869 1.353146

H 4.998904 -1.074246 2.362983

O -0.724660 0.574056 -1.115180

P -1.327422 -0.463315 -0.042434

C -2.169295 1.807324 3.841223

C -0.924561 1.910783 3.219744

C -0.668138 1.208544 2.045002

C -1.673541 0.396308 1.495349

C -2.925939 0.296745 2.114596

C -3.167345 1.000440 3.291029

H -2.363248 2.356337 4.755440

H -0.151940 2.538189 3.648033

H 0.297999 1.281628 1.562208

H -3.704422 -0.320603 1.683395

H -4.133664 0.922283 3.774595

C -5.162726 -2.432475 -1.606598

C -4.310258 -3.143565 -0.760677

C -3.134379 -2.556143 -0.302537

C -2.817533 -1.245933 -0.690490

C -3.672634 -0.529350 -1.537726

C -4.843574 -1.130862 -1.993913

H -6.075070 -2.894980 -1.965310

H -4.556940 -4.155331 -0.461912

H -2.470990 -3.114066 0.347418

H -3.418516 0.485603 -1.819754

H -5.506366 -0.581761 -2.652389

C 1.850775 -3.726915 0.422642

C 1.517214 -2.941983 1.523321

C 0.552999 -1.944011 1.406030

C -0.079360 -1.735423 0.175575

C 0.253864 -2.529848 -0.934018

C 1.219358 -3.520007 -0.805040

H 2.611632 -4.492119 0.515511

H 2.014098 -3.096380 2.473101

H 0.299025 -1.338343 2.266206

H -0.237769 -2.377207 -1.887786

H 1.485097 -4.125649 -1.662977

**TS1**

0 2

C 0.497545 3.883705 0.469754

C 0.318488 2.741275 -0.291089

C -0.802270 2.633704 -1.185478

C -1.641966 3.803162 -1.302979

C -1.436301 4.924285 -0.526899

 C -0.375258 4.975645 0.392291

H 1.358896 3.908300 1.128023

H -2.473018 3.767128 -1.997153

H -2.105385 5.771154 -0.635564

H -0.214273 5.850928 1.009375

C -1.289629 1.535753 -1.896842

O -1.916097 1.242846 -2.869806

O 1.257938 1.744141 -0.143562

C 2.120800 1.548095 -1.273521

H 2.465115 2.529841 -1.625669

H 1.567927 1.064556 -2.082413

C 5.558905 -0.817268 -0.185327

C 5.091636 -0.807694 -1.499984

C 3.967296 -0.055618 -1.836260

C 3.302791 0.704343 -0.868559

C 3.776748 0.692353 0.445657

C 4.895381 -0.067598 0.785640

H 6.425159 -1.412239 0.081335

H 5.593025 -1.396655 -2.260038

H 3.597204 -0.067250 -2.856676

H 3.255954 1.266443 1.201734

H 5.245670 -0.078377 1.811943

O -0.382616 -0.081359 -1.358969

P -0.831550 -0.861381 -0.107396

C -2.740490 1.852193 3.088922

C -1.352378 1.747728 3.002288

C -0.771555 0.915143 2.049952

C -1.584209 0.173155 1.181684

C -2.978963 0.277135 1.273625

C -3.552986 1.118228 2.223448

H -3.189207 2.504143 3.829707

H -0.720077 2.321489 3.669525

H 0.305666 0.852738 1.970535

H -3.615652 -0.295709 0.610312

H -4.631747 1.197496 2.289460

C -4.046445 -3.919843 -1.348141

C -3.374858 -4.086729 -0.135068

C -2.391120 -3.179166 0.248097

C -2.077463 -2.098230 -0.585470

C -2.747424 -1.932907 -1.802567

C -3.732359 -2.845553 -2.179660

H -4.811133 -4.628834 -1.644246

H -3.615156 -4.924318 0.509322

H -1.870898 -3.319026 1.188499

H -2.499762 -1.094185 -2.441989

H -4.252078 -2.716629 -3.122063

C 2.708027 -3.244886 1.604328

C 1.675409 -2.859844 2.459405

C 0.604436 -2.114019 1.970277

C 0.569507 -1.752758 0.617819

C 1.608441 -2.138752 -0.237450

C 2.674604 -2.882636 0.257844

H 3.543271 -3.818456 1.989615

H 1.704335 -3.133681 3.507581

H -0.188080 -1.810108 2.643305

H 1.590472 -1.838689 -1.277233

H 3.487182 -3.158766 -0.402012

**Int-3i**

0 2

 C -3.188322 -2.081922 0.611809

C -2.682597 -1.964315 -0.682078

C -1.483406 -2.624373 -1.016527

C -0.797037 -3.357038 -0.032434

C -1.298062 -3.453794 1.255694

C -2.502693 -2.820674 1.570593

H -4.112147 -1.574883 0.856562

H 0.130370 -3.846659 -0.305218

H -0.757083 -4.007921 2.012516

H -2.900540 -2.885661 2.576243

C -0.966349 -2.609557 -2.387634

O 0.014001 -3.124083 -2.832317

O -3.425559 -1.264555 -1.597973

C -2.846053 -0.071879 -2.181920

H -3.447815 0.105471 -3.075894

H -1.815037 -0.261039 -2.482710

C -2.911527 3.369743 0.423577

C -1.910424 3.223297 -0.537604

C -1.900711 2.104838 -1.369056

C -2.891968 1.124829 -1.258573

C -3.900632 1.284924 -0.305069

C -3.906700 2.397426 0.536687

H -2.910960 4.228315 1.085668

H -1.116854 3.957740 -0.618821

H -1.092607 1.963383 -2.074735

H -4.670880 0.529329 -0.211926

H -4.685658 2.502353 1.283775

O 0.342571 -0.161073 -1.468863

P 1.189588 0.205493 -0.272881

C -0.703347 -0.698456 3.848586

C -1.356520 0.119871 2.926359

C -0.781897 0.370806 1.684712

C 0.456946 -0.195785 1.353889

C 1.104577 -1.022478 2.279180

C 0.524464 -1.271383 3.522398

H -1.153912 -0.893992 4.815092

H -2.317382 0.558698 3.169398

H -1.296692 1.007441 0.977738

H 2.059364 -1.472112 2.036720

H 1.031401 -1.914895 4.232264

C 5.207892 -2.060077 -0.412681

C 5.138707 -0.849510 0.279131

C 3.940327 -0.139631 0.325478

C 2.805119 -0.641280 -0.321747

C 2.878244 -1.851975 -1.021014

C 4.079025 -2.559141 -1.063433

H 6.141348 -2.610278 -0.447892

H 6.017034 -0.457883 0.779258

H 3.893672 0.801725 0.861262

H 2.000706 -2.229478 -1.533679

H 4.133591 -3.496330 -1.605606

C 2.005351 4.753806 -0.238986

C 1.919818 4.074167 0.975742

C 1.681709 2.700159 0.993047

C 1.530762 1.999783 -0.208583

C 1.623431 2.685425 -1.426061

C 1.858315 4.057850 -1.440421

H 2.181697 5.823348 -0.250417

H 2.029313 4.613056 1.909813

H 1.598652 2.182321 1.941229

H 1.490839 2.146339 -2.356974

H 1.919019 4.584977 -2.385618

**Int-3ii**

0 2

C 2.903190 3.883109 -0.041253

C 1.992014 2.819919 0.020347

C 0.604801 3.103103 0.002825

C 0.161704 4.435321 -0.075451

C 1.068826 5.480965 -0.136030

C 2.436825 5.192505 -0.118209

H 3.967983 3.701166 -0.029871

H -0.905566 4.622915 -0.087160

H 0.724428 6.505814 -0.196248

H 3.157494 6.000926 -0.165029

C -0.368399 2.013889 0.065915

O -1.561914 2.063700 0.062170

O 2.347314 1.519737 0.096695

C 3.754332 1.168200 0.118206

H 4.227305 1.640750 0.983478

H 4.228489 1.536632 -0.795651

C 3.926000 -3.123015 0.369442

C 3.905394 -2.496208 -0.877246

C 3.861707 -1.104982 -0.957153

C 3.838531 -0.329126 0.205888

C 3.860390 -0.964019 1.451509

C 3.904077 -2.355072 1.534476

H 3.962956 -4.204602 0.432755

H 3.926119 -3.089266 -1.784383

H 3.847299 -0.618189 -1.926629

H 3.844955 -0.367470 2.357572

H 3.923775 -2.838259 2.504624

**Int-3iii**

0 2

C -2.501666 -0.975616 -0.668194

 C -1.505403 -0.739195 0.292735

C -1.413468 0.545572 0.875539

C -2.309268 1.557900 0.455780

C -3.279135 1.312422 -0.494988

C -3.373950 0.028345 -1.052549

H -2.558805 -1.964725 -1.105341

H -2.216229 2.536152 0.912521

H -3.958019 2.097713 -0.803882

H -4.130267 -0.183252 -1.799698

C -0.463091 0.871274 1.936955

O -0.304562 1.899000 2.524216

O -0.747976 -1.827678 0.578141

C 0.594247 -1.739510 1.109869

H 0.908642 -2.784142 1.146064

H 0.573564 -1.348056 2.128120

C 3.294904 0.515904 -1.383267

C 3.354317 0.639799 0.004219

C 2.466936 -0.076696 0.808533

C 1.523849 -0.932332 0.234156

C 1.471528 -1.055656 -1.158477

C 2.348999 -0.332445 -1.963270

H 3.977772 1.078293 -2.009892

H 4.081731 1.301118 0.461190

H 2.503842 0.035678 1.886634

H 0.735905 -1.712003 -1.609493

H 2.297279 -0.431603 -3.041661

**TS2**

0 2

 C -2.924775 -1.162388 -0.349581

C -1.620741 -0.827321 0.039539

C -1.287226 0.525890 0.255890

C -2.286670 1.513223 0.111774

C -3.574937 1.173097 -0.253871

C -3.886487 -0.174870 -0.491780

H -3.158592 -2.205713 -0.521628

H -2.011024 2.546262 0.287677

H -4.334644 1.937427 -0.362238

H -4.892789 -0.453096 -0.783107

C 0.067365 0.926368 0.623040

O 0.511474 2.022188 0.799367

O -0.757115 -1.865132 0.146479

C 0.478924 -1.704560 0.861609

H 0.939428 -2.692985 0.814876

H 0.262574 -1.467489 1.908287

C 3.582843 0.655738 -0.965343

C 3.596577 0.394394 0.417286

C 2.555052 -0.282923 1.016573

C 1.400838 -0.658711 0.261062

C 1.476748 -0.519905 -1.160573

C 2.526156 0.173172 -1.745210

H 4.402664 1.193369 -1.426588

H 4.441163 0.717366 1.016256

H 2.573242 -0.480182 2.083278

H 0.673705 -0.914940 -1.771296

H 2.536384 0.320969 -2.819600

**Int-4**

0 2

 C -2.929200 -1.228502 -0.336178

C -1.617493 -0.867106 -0.011243

C -1.294718 0.477366 0.254555

C -2.318675 1.441888 0.216089

C -3.619138 1.086002 -0.094056

C -3.917512 -0.256073 -0.376942

H -3.149525 -2.269189 -0.539424

H -2.054818 2.472334 0.421060

H -4.400077 1.835690 -0.125516

H -4.933433 -0.542389 -0.623915

C 0.102508 0.883872 0.500958

O 0.421791 2.017299 0.813282

O -0.691056 -1.859025 0.017180

C 0.527020 -1.562423 0.716857

H 1.194832 -2.397592 0.515282

H 0.312281 -1.517820 1.791207

C 3.784846 0.295785 -0.911654

C 3.619257 0.275648 0.496937

C 2.406983 0.032205 1.068536

C 1.169557 -0.229171 0.255913

C 1.439081 -0.241525 -1.230297

C 2.674067 0.026965 -1.746533

H 4.756046 0.500138 -1.344170

H 4.479020 0.460301 1.132479

H 2.295098 0.033079 2.147296

H 0.604026 -0.466717 -1.883311

H 2.810487 0.017166 -2.822901

**Ir(II)**

0 2

Ir -0.826401 -0.000631 0.000041

C 2.103859 -0.218531 0.678685

C 0.816580 -0.805645 2.562243

C 3.281695 -0.483071 1.435589

C 1.933131 -1.061468 3.324861

H -0.177564 -0.926846 2.976238

C 3.230071 -0.902909 2.744766

H 4.236104 -0.348477 0.949860

H 1.803949 -1.382228 4.349279

C 0.815662 0.806790 -2.562007

C 2.103606 0.221334 -0.678387

C 1.931918 1.064225 -3.324516

H -0.178619 0.926637 -2.976064

C 3.281135 0.487531 -1.435188

C 3.229031 0.907413 -2.744332

H 1.802373 1.384904 -4.348914

H 4.235693 0.354214 -0.949398

C -2.265217 -0.279541 1.400370

C -2.963234 -1.464267 1.663803

C -2.542677 0.875296 2.192564

C -3.894289 -1.492280 2.685683

H -2.788692 -2.364150 1.088057

C -3.499209 0.770271 3.210327

C -4.190041 -0.394211 3.482984

H -4.921900 -0.441132 4.276889

C -2.265479 0.276250 -1.400421

C -2.965442 1.459875 -1.663648

C -2.540918 -0.878854 -2.192928

C -3.896418 1.486586 -2.685633

H -2.792494 2.359902 -1.087649

C -3.497494 -0.775171 -3.210789

C -4.190234 0.388224 -3.483245

H -4.922078 0.434119 -4.277225

C -0.131969 -2.910020 -0.394301

C -1.776785 -2.076214 -1.856672

C -0.179683 -4.160971 -0.982683

H 0.533161 -2.693094 0.428128

C -1.856669 -3.332240 -2.479012

C -1.060482 -4.376059 -2.045235

H -2.538607 -3.481199 -3.299645

H -1.119265 -5.343078 -2.528814

C -0.136311 2.909794 0.394249

C -1.780415 2.073810 1.856181

C -0.186349 4.160871 0.982145

H 0.529531 2.693660 -0.427806

C -1.862670 3.329946 2.478007

C -1.068038 4.374922 2.044186

H -2.545250 3.478080 3.298256

H -1.128695 5.342040 2.527347

F -4.567452 2.642678 -2.933962

F -3.788547 -1.850017 -3.993738

F -3.792129 1.844817 3.992991

F -4.563406 -2.649435 2.934225

C 0.683959 -5.274340 -0.471799

C 0.675925 5.275426 0.471534

F -0.050501 6.216803 -0.187227

F 1.626581 4.845746 -0.385970

F 1.308832 5.924417 1.479697

F -0.041104 -6.215656 0.188590

F 1.316466 -5.923793 -1.479868

F 1.635017 -4.843032 0.384468

C 4.475478 1.209292 -3.581484

C 4.476867 -1.202962 3.582055

N 0.864238 -0.405759 1.277102

N 0.863775 0.406877 -1.276892

N -0.904987 -1.898592 -0.815140

N -0.907931 1.897270 0.815051

C 4.488280 -0.281096 4.823986

H 5.369433 -0.489933 5.437997

H 4.521385 0.770369 4.524581

H 3.603443 -0.428531 5.447138

C 5.781639 -0.976397 2.799265

H 5.880051 0.061357 2.468876

H 6.636633 -1.203011 3.441577

H 5.844592 -1.625422 1.921457

C 4.434797 -2.679468 4.041123

H 3.548697 -2.891663 4.643471

H 4.428252 -3.352247 3.178798

H 5.315990 -2.908913 4.647689

C 5.780504 0.984382 -2.798638

H 5.880333 -0.053299 -2.468444

H 6.635231 1.212306 -3.440842

H 5.842492 1.633323 -1.920699

C 4.488233 0.287632 -4.823559

H 3.603224 0.433939 -5.446731

H 5.369130 0.497781 -5.437488

H 4.522776 -0.763830 -4.524307

C 4.431437 2.685811 -4.040316

H 3.545105 2.896902 -4.642709

H 4.423900 3.358441 -3.177882

H 5.312372 2.916557 -4.646765

**Ir(III)**

1 1

Ir 0.813398 -0.008021 0.002787

C -2.129597 -0.141042 -0.719637

C -0.847673 -0.594895 -2.601435

C -3.293075 -0.304375 -1.461867

C -1.977377 -0.769808 -3.387281

H 0.145289 -0.704178 -3.017470

C -3.247610 -0.629519 -2.822975

H -4.249261 -0.178848 -0.976659

H -1.839618 -1.015847 -4.429774

C -0.833699 0.632205 2.603295

C -2.125644 0.197581 0.723869

C -1.959180 0.830607 3.389655

H 0.161576 0.723888 3.018043

C -3.285158 0.383878 1.466876

C -3.232367 0.711212 2.827181

H -1.815860 1.077524 4.431194

H -4.243942 0.274895 0.982821

C 2.257266 -0.243821 -1.394097

C 2.911854 -1.435102 -1.719429

C 2.581516 0.946226 -2.109009

C 3.854948 -1.434259 -2.732420

H 2.700844 -2.361640 -1.202356

C 3.547031 0.869179 -3.121201

C 4.199241 -0.301238 -3.456117

H 4.938952 -0.326070 -4.243723

C 2.261393 0.182115 1.402649

C 2.953809 1.351769 1.728337

C 2.545178 -1.017127 2.119453

C 3.894000 1.321278 2.743592

H 2.774703 2.284112 1.209641

C 3.510032 -0.970384 3.134041

C 4.199201 0.178534 3.469469

H 4.937334 0.180082 4.258941

C 0.109093 -2.943237 0.244225

C 1.779737 -2.194457 1.721790

C 0.175663 -4.229257 0.749054

H -0.574562 -2.688672 -0.551377

C 1.880466 -3.486661 2.258438

C 1.081190 -4.506376 1.774033

H 2.581559 -3.684181 3.052082

H 1.156614 -5.502766 2.190823

C 0.202223 2.948347 -0.239207

C 1.852809 2.146890 -1.712374

C 0.315120 4.232254 -0.739390

H -0.489464 2.715194 0.555712

C 1.999184 3.436628 -2.245739

C 1.233270 4.480877 -1.761673

H 2.711947 3.612749 -3.034004

H 1.349506 5.475927 -2.172884

F 4.558930 2.462542 3.053494

F 3.810011 -2.087185 3.846930

F 3.884949 1.976236 -3.832264

F 4.482832 -2.596303 -3.042178

C -0.688280 -5.311399 0.171442

C -0.540533 5.340778 -0.200831

F 0.207379 6.383938 0.235328

F -1.313492 4.944754 0.832191

F -1.371638 5.839031 -1.150848

F 0.002762 -6.104122 -0.688114

F -1.180118 -6.126897 1.132604

F -1.741049 -4.817283 -0.515916

C -4.527108 0.918974 3.616620

C -4.546650 -0.813917 -3.611125

N -0.910276 -0.294983 -1.297494

N -0.903131 0.329081 1.300462

N 0.884846 -1.955379 0.712406

N 0.947487 1.936009 -0.706833

C -5.355262 0.503712 -3.560302

H -6.284564 0.385115 -4.123573

H -5.618245 0.783426 -2.537777

H -4.787851 1.325839 -4.004368

C -5.367065 -1.954060 -2.962954

H -5.632811 -1.729918 -1.927525

H -6.295173 -2.100437 -3.521638

H -4.807053 -2.892813 -2.976425

C -4.283150 -1.173382 -5.083287

H -3.726189 -0.386934 -5.599490

H -3.730555 -2.111838 -5.178971

H -5.237382 -1.297011 -5.600313

C -5.332247 2.067858 2.964800

H -5.604069 1.842698 1.931181

H -6.256682 2.230589 3.525037

H -4.758507 2.998356 2.972279

C -5.354790 -0.387096 3.573279

H -4.798605 -1.215200 4.020445

H -6.281453 -0.252450 4.137296

H -5.623139 -0.667775 2.552396

C -4.255623 1.281414 5.086590

H -3.710183 0.488895 5.605784

H -3.688243 2.211567 5.176706

H -5.206955 1.422434 5.604512

**PF_6_^–^**

-1 1

P -6.803485 0.003051 -0.002200

F -8.280725 0.699630 -0.191764

F -7.526021 -1.426120 0.371389

F -5.325480 -0.695622 0.187889

F -6.080854 1.432422 -0.375934

F -6.808563 0.421146 1.587750

F -6.795622 -0.416553 -1.591726

**Ir(III)•PF_6_**

0 1

Ir -1.784500 -0.032774 -0.006056

C 1.153492 -0.104553 0.754300

C -0.151552 -0.633655 2.602593

C 2.310650 -0.290151 1.503511

C 0.968242 -0.826714 3.396471

H -1.149234 -0.764702 3.000682

C 2.244799 -0.666006 2.852541

H 3.273860 -0.142412 1.037677

H 0.818408 -1.106809 4.428610

C -0.132743 0.769396 -2.547518

C 1.158340 0.295401 -0.674800

C 0.991387 1.044438 -3.310678

H -1.126938 0.844003 -2.968169

C 2.319253 0.559335 -1.393768

C 2.262498 0.947320 -2.739782

H 0.848746 1.334354 -4.341129

H 3.278203 0.472067 -0.904799

C -3.244366 -0.366075 1.354375

C -3.853880 -1.592132 1.635939

C -3.634873 0.790345 2.091170

C -4.818263 -1.657034 2.626377

H -3.591132 -2.495411 1.101631

C -4.618517 0.646800 3.078290

C -5.227268 -0.558280 3.369219

H -5.982526 -0.634547 4.138562

C -3.214061 0.136829 -1.428178

C -3.958237 1.281924 -1.726844

C -3.421831 -1.047041 -2.195032

C -4.873965 1.242959 -2.763768

H -3.837593 2.201887 -1.170452

C -4.365505 -1.009917 -3.229729

C -5.104892 0.114946 -3.538588

H -5.825079 0.109600 -4.344439

C -0.952098 -2.928072 -0.317898

C -2.609416 -2.200270 -1.820548

C -0.941439 -4.194873 -0.872236

H -0.307738 -2.673511 0.509633

C -2.629305 -3.473111 -2.410588

C -1.796930 -4.472104 -1.939654

H -3.294006 -3.671506 -3.234742

H -1.809934 -5.452875 -2.398256

C -1.294138 2.935156 0.334459

C -2.947411 2.029850 1.743143

C -1.470951 4.199584 0.864951

H -0.573668 2.750292 -0.447483

C -3.158786 3.297929 2.305828

C -2.423751 4.384306 1.868735

H -3.897464 3.424393 3.079633

H -2.590220 5.363064 2.301059

F -5.589916 2.360431 -3.046605

F -4.593245 -2.112506 -3.990305

F -5.019231 1.720284 3.808447

F -5.402451 -2.852317 2.893399

C -0.046185 -5.260132 -0.311440

C -0.641828 5.353079 0.382885

F -1.413589 6.404937 0.015124

F 0.127657 5.031276 -0.678113

F 0.190577 5.812288 1.351761

F -0.742972 -6.172766 0.414127

F 0.583312 -5.951643 -1.290434

F 0.906444 -4.758116 0.503227

C 3.550493 1.239022 -3.511311

C 3.528533 -0.898619 3.650988

N -0.072481 -0.292663 1.310049

N -0.063117 0.409937 -1.259599

N -1.757272 -1.958717 -0.775756

N -2.010802 1.882400 0.755019

C 4.395376 0.381226 3.604198

H 5.311364 0.221268 4.178910

H 4.686628 0.640456 2.586821

H 3.857910 1.226077 4.044122

C 4.292410 -2.079571 3.005958

H 4.578787 -1.863608 1.976637

H 5.209677 -2.272308 3.568419

H 3.682156 -2.986976 3.020197

C 3.240833 -1.243457 5.122230

H 2.701936 -0.438633 5.629835

H 2.661888 -2.165817 5.219959

H 4.187237 -1.391675 5.647335

C 4.327085 2.360557 -2.781982

H 4.618489 2.065516 -1.774319

H 5.242238 2.587870 -3.334627

H 3.724421 3.271454 -2.724926

C 4.401747 -0.051735 -3.555510

H 3.858498 -0.853086 -4.064468

H 5.326294 0.139929 -4.106272

H 4.678847 -0.395116 -2.559051

C 3.270319 1.693654 -4.953835

H 2.748301 0.924669 -5.529785

H 2.677974 2.612408 -4.982605

H 4.218970 1.895006 -5.456757

P 6.785237 0.044997 -0.021557

F 8.290861 -0.574456 -0.244994

F 7.428467 1.393670 0.662909

F 5.265308 0.664577 0.201059

F 6.121233 -1.296611 -0.702676

F 6.707765 -0.664327 1.454009

F 6.856312 0.760088 -1.494564

**Int-4i**

-1 1

 C -2.939062 -1.194908 -0.288559

C -1.617064 -0.896396 0.056154

C -1.196637 0.442793 0.190081

C -2.149967 1.460549 0.017939

C -3.466320 1.169518 -0.311706

C -3.856652 -0.166667 -0.473959

H -3.228305 -2.234118 -0.393139

H -1.820168 2.486082 0.138032

H -4.186289 1.968093 -0.448196

H -4.881517 -0.406692 -0.734126

C 0.212178 0.757048 0.552698

O 0.562763 1.902175 0.849035

O -0.774722 -1.949043 0.231758

C 0.474304 -1.678881 0.902605

H 1.089139 -2.564163 0.732783

H 0.268933 -1.583026 1.977237

C 3.524353 0.736991 -0.941377

C 3.562466 0.381151 0.438875

C 2.523277 -0.240422 1.071489

C 1.185777 -0.422755 0.389356

C 1.378084 -0.425383 -1.124828

C 2.450159 0.234337 -1.696386

H 4.375535 1.207910 -1.418862

H 4.479018 0.558097 0.999865

H 2.616767 -0.568562 2.103601

H 0.599546 -0.866016 -1.736509

H 2.485658 0.310495 -2.782366

***6a, N,N*-*tert*-butylbenzylacrylamide**

0 1

C -0.144606 -0.091684 0.732499

C -0.305194 1.229413 0.310534

C 0.799077 1.990290 -0.076519

C 2.070386 1.406396 -0.035466

C 2.233863 0.087670 0.383162

C 1.124298 -0.667399 0.769814

H -1.012327 -0.668060 1.033638

H -1.293432 1.673005 0.295014

H 2.937871 1.990176 -0.327633

H 3.225865 -0.349161 0.413221

H 1.250075 -1.692279 1.099976

C 0.662433 3.420692 -0.571459

H 0.803435 3.441401 -1.652915

H 1.482189 4.021563 -0.179629

N -0.605476 4.087610 -0.247914

C -0.731832 4.848267 1.055403

C -0.599018 6.356655 0.780633

H -0.645536 6.910017 1.722610

H 0.363504 6.571411 0.307548

H -1.397675 6.705439 0.127935

C 0.375914 4.426270 2.039843

H 0.376510 3.349655 2.216316

H 1.372107 4.730978 1.715931

H 0.182980 4.923164 2.992413

C -2.079892 4.513029 1.722888

H -2.146685 3.439773 1.919211

H -2.144293 5.037404 2.679538

H -2.923852 4.812558 1.107294

C -1.576664 4.189849 -1.208984

O -2.527439 4.974480 -1.131632

C -2.101637 3.526980 -3.529270

C -1.473871 3.258502 -2.384920

H -2.057268 2.843631 -4.369139

H -0.912372 2.337791 -2.279904

H -2.674740 4.439713 -3.649235

**PhCO_2_H**

0 1

C 1.926567 -1.846214 0.000176

C 1.444304 -0.540119 0.000493

C 2.342328 0.535769 0.000239

C 3.721914 0.291983 -0.000363

C 4.199014 -1.014409 -0.000679

C 3.301808 -2.084655 -0.000411

H 1.230959 -2.677071 0.000384

H 4.406076 1.131295 -0.000561

H 5.266617 -1.199804 -0.001135

H 3.674163 -3.102708 -0.000661

C 1.877766 1.947135 0.000597

O 0.532115 2.064839 0.000458

O 2.610713 2.916033 -0.000167

H 0.378659 -0.353196 0.000950

H 0.312240 3.009093 0.000183

**PhCOO^–^K^+^**

0 1

 C -2.964367 1.190885 0.227979

C -1.569985 1.186103 0.226320

C -0.859924 0.000971 0.000496

C -1.574674 -1.181580 -0.224079

C -2.969063 -1.181302 -0.223287

C -3.668026 0.006064 0.002963

H -3.502417 2.116008 0.404260

H -1.023142 -2.097060 -0.398518

H -3.510775 -2.104467 -0.398616

H -4.752499 0.008028 0.003919

C 0.664065 -0.001790 -0.000824

O 1.246677 -1.103290 -0.210503

O 1.251015 1.097582 0.207903

K 3.763051 -0.007798 -0.002092

H -1.014831 2.099577 0.399791

**PhCOO^–^PPh_3_^+•^**

0 2

 C 4.225441 -3.383537 1.000971

C 3.080307 -2.603057 0.853430

C 2.512804 -2.414360 -0.412448

C 3.107210 -3.016068 -1.527506

C 4.253761 -3.793503 -1.380550

C 4.814537 -3.979581 -0.115411

H 4.658105 -3.527067 1.984678

H 2.661064 -2.866310 -2.502718

H 4.709257 -4.254667 -2.249660

H 5.706025 -4.585910 -0.000364

C 1.263933 -1.578472 -0.596079

O 0.738360 -1.486005 -1.715264

O 0.825257 -1.013357 0.479733

P -1.158468 -0.003882 -0.000539

C -2.900261 2.771537 -3.250196

C -2.986661 3.157219 -1.911997

C -2.482993 2.332282 -0.908211

C -1.892620 1.104441 -1.244620

C -1.784127 0.730732 -2.595350

C -2.298639 1.557321 -3.589176

H -3.294263 3.416892 -4.026771

H -3.451734 4.099724 -1.646415

H -2.561777 2.635834 0.128409

H -1.291510 -0.199300 -2.855467

H -2.221357 1.260041 -4.628646

C -3.714976 -3.837269 0.260709

C -4.119964 -2.796906 -0.574550

C -3.359003 -1.632909 -0.666783

C -2.178315 -1.504670 0.078613

C -1.772674 -2.556141 0.916540

C -2.541928 -3.712034 1.007390

H -4.309640 -4.740783 0.330683

H -5.032034 -2.885903 -1.153356

H -3.691026 -0.829312 -1.311419

H -0.857252 -2.467968 1.487044

H -2.222672 -4.517566 1.658555

C -1.382628 2.209495 4.036776

C -2.332037 1.231048 3.742550

C -2.276839 0.542360 2.531794

C -1.265403 0.833869 1.607839

C -0.307594 1.811835 1.910629

C -0.371150 2.499646 3.119584

H -1.428489 2.742999 4.979209

H -3.118281 1.003875 4.453191

H -3.019381 -0.213900 2.308268

H 0.485379 2.033731 1.205371

H 0.370766 3.255954 3.347864

H 2.617758 -2.137218 1.714162

**Int-2’**

0 2

 C 1.599011 3.626687 -0.327888

C 0.800603 2.514508 -0.534195

C -0.551856 2.659354 -0.979503

C -1.030056 3.989807 -1.210704

C -0.211234 5.084548 -1.000259

C 1.113847 4.926680 -0.554548

H 2.619724 3.486981 0.014565

H -2.050609 4.123554 -1.549344

H -0.601901 6.081306 -1.180762

H 1.748462 5.789633 -0.391241

C -1.434723 1.575644 -1.191928

O -2.631795 1.533785 -1.508990

O -0.762967 0.264299 -0.958271

P -1.227555 -0.799893 0.144511

C -3.471744 1.369877 3.489120

C -2.230004 1.834049 3.053324

C -1.549670 1.162573 2.041593

C -2.126323 0.019865 1.463839

C -3.375365 -0.442893 1.895507

C -4.042184 0.232755 2.913848

H -3.996523 1.895494 4.278462

H -1.790905 2.718205 3.499762

H -0.585805 1.525562 1.705240

H -3.821949 -1.319815 1.443013

H -5.005973 -0.125627 3.254814

C -3.889409 -3.997858 -1.765172

C -2.925732 -4.374693 -0.829332

C -2.096580 -3.413040 -0.257110

C -2.242007 -2.069742 -0.626732

C -3.206680 -1.687319 -1.569786

C -4.028193 -2.658840 -2.134957

H -4.531208 -4.749529 -2.210032

H -2.816196 -5.414974 -0.547379

H -1.345939 -3.707321 0.466566

H -3.301884 -0.643594 -1.848768

H -4.774919 -2.371439 -2.865689

C 2.640892 -2.691614 1.707658

C 1.619813 -2.378296 2.604723

C 0.441876 -1.793038 2.146271

C 0.290916 -1.523680 0.780345

C 1.315656 -1.840907 -0.123847

C 2.488956 -2.421853 0.345910

H 3.556448 -3.145386 2.068674

H 1.739276 -2.586036 3.661249

H -0.348056 -1.546769 2.845229

H 1.199826 -1.627104 -1.179532

H 3.284283 -2.663029 -0.349124

H 1.199930 1.523954 -0.358764

**TS3´**

0 2

 C 1.542161 3.537300 -0.293831

C 0.689780 2.506463 -0.639421

C -0.652813 2.789095 -1.058044

C -1.057877 4.174488 -1.119011

C -0.176906 5.175542 -0.763339

C 1.133343 4.882351 -0.341496

H 2.552728 3.295537 0.019641

H -2.064528 4.417054 -1.438123

H -0.508670 6.207713 -0.813314

H 1.814531 5.677376 -0.063896

C -1.632772 1.865545 -1.372245

O -2.734781 1.670004 -1.780346

O -0.785364 0.142721 -1.147749

P -1.178385 -0.812206 -0.002899

C -3.415050 1.431009 3.336167

C -2.129885 1.821478 2.952923

C -1.461129 1.127177 1.950171

C -2.081460 0.036172 1.323096

C -3.369434 -0.352016 1.706412

C -4.032618 0.346719 2.715003

H -3.933257 1.973088 4.118850

H -1.650392 2.665792 3.434106

H -0.465707 1.437420 1.653839

H -3.854660 -1.192299 1.225208

H -5.029726 0.043655 3.011873

C -3.951825 -4.100091 -1.621814

C -3.068724 -4.409475 -0.586729

C -2.206544 -3.434303 -0.089474

C -2.231389 -2.144932 -0.633228

C -3.114488 -1.834652 -1.675705

C -3.974274 -2.814863 -2.165685

H -4.620089 -4.861637 -2.007031

H -3.048334 -5.409083 -0.168851

H -1.520164 -3.680331 0.712057

H -3.124354 -0.834314 -2.093699

H -4.658321 -2.577003 -2.971883

C 2.622054 -2.683101 1.787948

C 1.486769 -2.547973 2.587567

C 0.330265 -1.971500 2.066334

C 0.310819 -1.534674 0.736187

C 1.451449 -1.668240 -0.065197

C 2.605224 -2.241030 0.464188

H 3.521497 -3.127986 2.197724

H 1.502570 -2.884487 3.617470

H -0.544604 -1.855342 2.695474

H 1.435567 -1.316046 -1.089760

H 3.489766 -2.339606 -0.154014

H 1.023646 1.480752 -0.605587

**Int-3’i**

0 2

 C 0.208148 3.516229 -0.621050

C -1.003898 2.979608 -0.196980

C -1.988551 2.661272 -1.138545

C -1.766611 2.892943 -2.507420

C -0.557110 3.432938 -2.922511

C 0.430665 3.740450 -1.979607

H 0.979239 3.747096 0.103458

H -2.538229 2.638203 -3.223965

H -0.374572 3.608684 -3.976159

H 1.377195 4.152954 -2.309568

C -3.248189 2.056145 -0.680916

O -4.173452 1.654001 -1.314355

O 2.490579 0.315148 -0.167909

P 1.364646 -0.527697 0.388098

C -1.066369 1.815924 3.537775

C 0.270891 2.128382 3.287635

C 0.989362 1.407383 2.336127

C 0.370414 0.371307 1.627428

C -0.972722 0.065299 1.875087

C -1.686625 0.784601 2.831776

H -1.626074 2.380776 4.274352

H 0.750981 2.936311 3.827504

H 2.023129 1.656653 2.127606

H -1.469017 -0.718436 1.314928

H -2.728228 0.548902 3.015464

C -1.658531 -1.770748 -2.873689

C -1.371894 -2.662970 -1.840498

C -0.449547 -2.316278 -0.854006

C 0.186197 -1.069536 -0.896292

C -0.103530 -0.177046 -1.936020

C -1.022067 -0.529719 -2.921272

H -2.374876 -2.042017 -3.640785

H -1.861901 -3.629059 -1.803060

H -0.226567 -3.017600 -0.058475

H 0.384598 0.789649 -1.972484

H -1.244411 0.166247 -3.721334

C 2.934036 -4.425979 2.308321

C 1.876265 -3.752653 2.918173

C 1.389505 -2.567645 2.366396

C 1.961065 -2.052771 1.197222

C 3.027128 -2.730701 0.590098

C 3.510392 -3.913140 1.144651

H 3.311167 -5.346311 2.739431

H 1.430692 -4.146214 3.824470

H 0.570196 -2.049750 2.850227

H 3.478767 -2.331024 -0.310856

H 4.335504 -4.432837 0.671379

H -1.188028 2.789864 0.852544

**Int-3’ii**

0 2

 C 2.019394 -1.677195 -0.000065

C 1.500042 -0.385607 -0.000001

C 2.368714 0.712765 0.000043

C 3.761629 0.517982 0.000027

C 4.272060 -0.773478 -0.000035

C 3.401781 -1.868975 -0.000081

H 1.351947 -2.530464 -0.000102

H 4.421042 1.377749 0.000063

H 5.344016 -0.932674 -0.000048

H 3.805534 -2.874907 -0.000130

C 1.806441 2.071899 0.000109

O 2.359291 3.126452 0.000150

H 0.429372 -0.217353 0.000013

**Int-3’iii**

0 2

 C -1.351176 2.994593 -0.113994

C -1.241678 1.608338 -0.231472

C -2.230590 0.873214 -0.887145

C -3.331829 1.548427 -1.425305

C -3.442655 2.932684 -1.310729

C -2.450308 3.661949 -0.652332

H -0.576932 3.551498 0.400324

H -0.390086 1.097773 0.201121

H -4.110820 0.986289 -1.931257

H -4.304333 3.441034 -1.729009

H -2.536143 4.738496 -0.557633

C -2.128307 -0.631162 -1.066977

H -1.816555 -0.847156 -2.089913

H -3.118798 -1.079209 -0.984620

N -1.220513 -1.324075 -0.142986

C -1.762667 -1.822954 1.177843

C -0.720868 -1.591871 2.288533

H -1.144273 -1.908892 3.244859

H 0.195254 -2.148663 2.112355

H -0.477655 -0.529465 2.358018

C -2.128980 -3.311741 1.048421

H -2.559141 -3.672157 1.986896

H -2.872163 -3.452635 0.258192

H -1.248249 -3.907735 0.813380

C -3.022931 -1.030575 1.576870

H -3.866912 -1.203377 0.907713

H -3.327710 -1.364476 2.570485

H -2.827903 0.041609 1.625786

C -0.006205 -1.760060 -0.608284

O 0.655476 -2.644169 -0.057525

C 1.442674 -1.661940 -2.605577

C 0.531743 -1.074162 -1.831617

H 1.795365 -2.663536 -2.387523

H 0.199979 -0.066506 -2.053472

H 1.860640 -1.156130 -3.467716

C 2.034982 0.936561 2.152601

C 1.823211 2.228047 1.661135

C 2.277281 2.586640 0.391171

C 2.950115 1.653561 -0.391524

C 3.174803 0.363769 0.104271

C 2.711046 0.001377 1.379574

H 1.670590 0.664276 3.135799

H 1.294472 2.952929 2.269453

H 2.102962 3.586528 0.012811

H 3.305109 1.912204 -1.382213

H 2.879218 -1.004750 1.742993

C 3.913463 -0.603598 -0.723095

O 4.288755 -1.702281 -0.460108

**TS4’**

0 2

C -1.974910 2.564583 0.319516

C -1.565553 1.263838 0.022847

C -2.400856 0.403606 -0.691595

C -3.651984 0.869662 -1.111495

C -4.061353 2.169482 -0.820910

C -3.222858 3.022583 -0.100088

H -1.317062 3.217842 0.880745

H -0.596449 0.915691 0.357882

H -4.313319 0.207756 -1.662351

H -5.035580 2.514197 -1.149536

H -3.541669 4.032053 0.133740

C -1.983392 -1.013664 -1.047582

H -1.696363 -1.049531 -2.099717

H -2.847737 -1.674586 -0.977111

N -0.891170 -1.577421 -0.248362

C -1.204438 -2.275135 1.054662

C -0.201098 -1.822545 2.134340

H -0.459828 -2.295284 3.085236

H 0.819639 -2.094066 1.878455

H -0.254042 -0.738774 2.266677

C -1.160952 -3.800358 0.849436

H -1.431420 -4.308097 1.779597

H -1.878345 -4.098045 0.079062

H -0.166121 -4.122991 0.547563

C -2.609795 -1.888516 1.554966

H -3.407141 -2.254906 0.906528

H -2.754600 -2.349413 2.533928

H -2.719064 -0.809561 1.671412

C 0.371959 -1.648654 -0.789400

O 1.251437 -2.398465 -0.340730

C 1.880088 -0.837234 -2.563857

C 0.692023 -0.727337 -1.907677

H 2.460416 -1.747429 -2.489641

H 0.063290 0.135393 -2.085711

H 2.126197 -0.158096 -3.371052

C 2.501670 1.659394 2.168713

C 1.778579 2.747762 1.673670

C 1.598992 2.910349 0.298955

C 2.122859 1.973189 -0.585043

C 2.868473 0.897558 -0.091495

C 3.061120 0.739005 1.289160

H 2.631057 1.533542 3.237280

H 1.348317 3.466115 2.362045

H 1.035286 3.753526 -0.080528

H 1.963121 2.070080 -1.651785

H 3.629963 -0.106656 1.656588

C 3.433435 -0.091212 -1.023929

O 4.367012 -0.813405 -0.893011

**7a**

0 2

 C -1.431494 1.889573 -1.525289

C -1.318666 0.891896 -0.555049

C -2.310146 0.726134 0.413140

C -3.423775 1.574764 0.390991

C -3.539604 2.571057 -0.575654

C -2.540825 2.732518 -1.539339

H -0.646138 2.003961 -2.263632

H -0.451579 0.245717 -0.559720

H -4.205523 1.452306 1.134449

H -4.409156 3.218924 -0.580025

H -2.630288 3.506751 -2.292938

C -2.211320 -0.321556 1.512494

H -3.183847 -0.788435 1.648886

H -2.013348 0.168383 2.466908

N -1.212350 -1.374057 1.301728

C -1.594565 -2.643598 0.568284

C -1.533443 -3.836781 1.539837

H -1.868469 -4.742921 1.027403

H -0.521368 -3.994010 1.906744

H -2.196335 -3.661908 2.391735

C -0.654845 -2.851383 -0.637357

H -0.936426 -3.771461 -1.155715

H -0.752281 -2.021462 -1.341075

H 0.384883 -2.933760 -0.330763

C -3.028441 -2.543981 0.016447

H -3.163701 -1.671529 -0.625792

H -3.214731 -3.433831 -0.587632

H -3.782899 -2.527138 0.805136

C 0.075934 -1.207556 1.758894

O 0.917188 -2.125341 1.737025

C 1.909582 0.422234 2.516360

C 0.480819 0.119450 2.228271

H 2.032346 0.796414 3.537079

H -0.214719 0.943966 2.284385

H 2.515554 -0.478549 2.398082

C 2.420587 2.233657 -2.121344

C 2.332882 0.947460 -2.659941

C 2.293901 -0.161317 -1.814618

C 2.329644 0.010668 -0.432952

C 2.411665 1.299219 0.114983

C 2.466476 2.407823 -0.743299

H 2.444894 3.096355 -2.776946

H 2.291716 0.811706 -3.734676

H 2.226980 -1.160280 -2.229218

H 2.285791 -0.860245 0.207796

H 2.524997 3.400506 -0.314314

C 2.419333 1.538222 1.590208

O 2.772670 2.609597 2.055317

**TS5´**

0 2

 C -0.842784 -1.535928 -4.157074

C -1.137415 -1.868347 -2.849708

C -1.353031 -0.853437 -1.858465

C -1.457723 0.492239 -2.333516

C -1.158289 0.811358 -3.646587

C -0.832619 -0.193010 -4.569738

H -0.637520 -2.321326 -4.876205

H -1.155497 -2.907726 -2.540427

H -1.717723 1.275476 -1.629423

H -1.191780 1.847230 -3.965984

H -0.599637 0.062126 -5.596443

C -2.065904 -1.245507 -0.564558

H -3.024877 -1.693928 -0.813368

H -2.265805 -0.348941 0.033141

N -1.246088 -2.211488 0.182129

C -1.830091 -3.247988 1.098816

C -1.244287 -3.058532 2.510725

H -1.675493 -3.799131 3.189060

H -0.162306 -3.179333 2.509565

H -1.488118 -2.062470 2.890265

C -1.509934 -4.649037 0.547845

H -1.955481 -5.411037 1.193231

H -1.926725 -4.764210 -0.456471

H -0.434210 -4.812475 0.502205

C -3.356212 -3.086851 1.186941

H -3.851346 -3.301808 0.237829

H -3.732108 -3.802873 1.920531

H -3.643305 -2.086446 1.519042

C 0.096885 -2.057994 0.014208

O 0.965425 -2.834315 0.428934

C 1.642532 -0.839929 -1.660828

C 0.428682 -0.836576 -0.772505

H 1.921099 -1.859409 -1.923452

H 0.223825 0.091033 -0.244138

H 1.408031 -0.303773 -2.587796

C 3.777403 3.190236 0.432063

C 2.818973 4.026489 -0.146520

C 1.858359 3.494650 -1.006551

C 1.849297 2.129785 -1.285022

C 2.809664 1.282983 -0.711849

C 3.774883 1.829633 0.147782

H 4.522793 3.601669 1.102821

H 2.821788 5.088037 0.073316

H 1.115575 4.140717 -1.459782

H 1.094995 1.737223 -1.954619

H 4.514121 1.172227 0.588580

C 2.850349 -0.186590 -0.994555

O 3.823349 -0.857272 -0.684274

**8ai**

0 2

C -1.548376 -1.157153 -3.571947

C -1.338772 -1.359438 -2.239913

C -1.486908 -0.262426 -1.216988

C -1.975376 1.018813 -1.825712

C -2.160342 1.176803 -3.168617

 C -1.943985 0.108120 -4.073839

H -1.418740 -1.983634 -4.263117

H -1.047647 -2.337929 -1.872200

H -2.150938 1.846936 -1.146195

H -2.486864 2.137900 -3.552194

H -2.097441 0.251758 -5.136042

C -2.395784 -0.759008 -0.049065

H -3.200503 -1.386570 -0.424221

H -2.835458 0.092768 0.483111

N -1.483832 -1.510119 0.823907

C -1.946822 -2.378873 1.951980

C -1.692625 -1.647097 3.282086

H -2.034564 -2.263384 4.117525

H -0.630011 -1.441913 3.413821

H -2.239909 -0.700811 3.307633

C -1.186638 -3.715691 1.901600

H -1.546926 -4.369646 2.699922

H -1.360819 -4.213052 0.943782

H -0.116129 -3.564996 2.028764

C -3.449049 -2.661817 1.805247

H -3.670609 -3.193069 0.876761

H -3.763713 -3.295968 2.636485

H -4.045277 -1.747449 1.840513

C -0.199356 -1.093558 0.679010

O 0.777659 -1.449219 1.335248

C 1.125237 -0.050946 -1.268104

C -0.151945 -0.041076 -0.423173

H 1.290934 -1.035137 -1.705551

H -0.242203 0.918705 0.098183

H 0.988457 0.653467 -2.096977

C 3.452176 3.007872 1.925357

C 2.666218 4.093116 1.530414

C 1.779172 3.959296 0.462250

C 1.666306 2.740238 -0.202616

C 2.456478 1.648104 0.183066

C 3.354426 1.796453 1.249454

H 4.139173 3.109360 2.757513

H 2.745461 5.039186 2.053609

H 1.174219 4.801763 0.148120

H 0.975507 2.654705 -1.032538

H 3.963007 0.949688 1.542375

C 2.395172 0.330279 -0.524649

O 3.364293 -0.413847 -0.528204

**8aii**

-1 1

C 1.641708 -3.202488 -0.951279

C 1.896653 -1.874609 -0.709655

C 1.190929 -1.091610 0.384401

C 0.558238 -2.056400 1.360583

C 0.330327 -3.378992 1.056162

C 0.805638 -3.996599 -0.125902

H 2.138678 -3.670310 -1.801387

H 2.559997 -1.318043 -1.367951

H 0.166357 -1.634696 2.284039

H -0.218254 -3.980889 1.780768

H 0.650818 -5.051605 -0.318791

C 2.164963 -0.084866 1.058890

H 3.167341 -0.504214 1.122579

H 1.826691 0.166041 2.073975

N 2.138940 1.108339 0.193867

C 3.118520 2.231768 0.286799

C 2.409477 3.471833 0.863036

H 3.115883 4.302060 0.948712

H 1.586117 3.780276 0.218728

H 2.014252 3.253617 1.859209

C 3.693169 2.528018 -1.110533

H 4.439769 3.324142 -1.040967

H 4.178898 1.635629 -1.514833

H 2.907998 2.840370 -1.796843

C 4.276248 1.840728 1.218148

H 4.833706 0.984373 0.832314

H 4.965574 2.685122 1.288073

H 3.928482 1.607153 2.226713

C 0.996043 1.167035 -0.540315

O 0.682307 2.060553 -1.333805

C -0.716552 -0.596497 -1.336473

C 0.154774 -0.050478 -0.196549

H -0.187315 -0.531741 -2.286278

H -0.478392 0.260453 0.644928

H -0.886248 -1.657495 -1.125984

C -5.143525 0.791683 0.569873

C -5.013042 -0.129336 1.612231

C -3.910062 -0.982875 1.651591

C -2.934643 -0.914814 0.658819

C -3.061294 0.002381 -0.395499

C -4.177304 0.851943 -0.428730

H -5.997501 1.458736 0.538048

H -5.766806 -0.179372 2.390000

H -3.806091 -1.699739 2.458187

H -2.076704 -1.574855 0.716857

H -4.271867 1.557434 -1.245129

C -2.053287 0.090824 -1.505790

O -2.331397 0.694942 -2.535435

**9a**

0 1

 C 2.058610 -3.225002 -1.041446

C 1.968005 -1.911362 -0.840015

C 1.264586 -1.283407 0.342854

C 0.780127 -2.322153 1.318926

C 0.853817 -3.637013 1.111843

C 1.471560 -4.248472 -0.112608

H 2.584681 -3.598060 -1.915640

H 2.417636 -1.221867 -1.549624

H 0.321285 -1.941644 2.228279

H 0.456383 -4.320215 1.856979

C 2.204063 -0.238706 1.004432

H 3.239847 -0.569844 0.974235

H 1.923426 -0.069756 2.051097

N 2.002616 0.981154 0.208101

C 2.864761 2.198320 0.328895

C 2.103501 3.273021 1.125806

H 2.723620 4.166680 1.233864

H 1.180236 3.550128 0.616379

H 1.857234 2.903842 2.125229

C 3.222905 2.704392 -1.079479

H 3.884523 3.570747 -0.997963

H 3.744774 1.923873 -1.639575

H 2.331156 2.995246 -1.631739

C 4.163588 1.841803 1.066889

H 4.740428 1.087977 0.526072

H 4.776849 2.742121 1.141740

H 3.975381 1.482429 2.080903

C 0.789975 0.980118 -0.404909

O 0.283366 1.895080 -1.052327

C -0.761941 -0.887735 -1.260732

C 0.104223 -0.351095 -0.116561

H -0.191632 -0.936464 -2.187886

H -0.525239 -0.156790 0.759929

H -1.056086 -1.913366 -1.007934

C -4.827952 1.448900 0.467291

C -4.922936 0.574402 1.552510

C -4.067595 -0.524111 1.639488

C -3.109810 -0.742857 0.651709

C -3.012453 0.126126 -0.444532

C -3.884859 1.220426 -0.528994

H -5.660980 0.749158 2.327030

H -4.144032 -1.208694 2.476140

H -2.455485 -1.602178 0.733527

C -2.022256 -0.089133 -1.547407

O -2.241606 0.348757 -2.666994

H -5.489389 2.304949 0.400317

H -3.806003 1.887937 -1.378390

H 0.723590 -4.852622 -0.646063

H 2.245219 -4.970399 0.183434

# 3. Supplementary Note 1

## 3.1 Characterization data for substrates and products

2-(Benzyloxy)-6-methoxybenzoic acid (1h)**.** ^1^H NMR (400 MHz, CDCl_3_): δ 7.51–7.25 (m, 6H), 6.63 (dd, *J* = 8.3, 6.4 Hz, 2H), 5.23 (d, *J* = 28.5 Hz, 2H), 3.88 (d, *J* = 5.8 Hz, 3H); ^13^C NMR (101 MHz, CDCl_3_): δ 170.3, 157.9, 156.9, 136.5, 131.8, 128.6, 127.9, 126.9, 112.3, 105.9, 104.4, 70.7, 56.2; HRMS (ESI-TOF, *m/z*): calcd for C_15_H_14_O_4_ [M + H]^+^, 259.0965; found, 259.0963.

2-(Benzyloxy)-5-bromo-4-methylbenzoic acid (1k)**.** ^1^H NMR (400 MHz, CDCl_3_): δ 8.34 (s, 1H), 7.52–7.39 (m, 5H), 7.04 (s, 1H), 5.28 (s, 3H), 2.47 (s, 3H); ^13^C NMR (101 MHz, CDCl_3_): δ 164.1, 156.3, 145.7, 136.9, 134.0, 129.4, 129.2, 128.0, 117.5, 117.3, 115.5, 72.6, 23.8; HRMS (ESI-TOF, *m/z*): calcd for C_15_H_13_BrO_3_ [M + H]^+^, 321.0121; found, 321.0120.

2-((2-Fluorobenzyl)oxy)-4-methoxybenzoic acid (1l)**.** ^1^H NMR (400 MHz, CDCl_3_): δ 10.52 (s, 1H), 8.17 (d, *J* = 8.6 Hz, 1H), 7.56–7.38 (m, 2H), 7.20 (dt, *J* = 18.4, 8.1 Hz, 2H), 6.75–6.61 (m, 2H), 5.36 (s, 2H), 3.90 (s, 3H); ^13^C NMR (101 MHz, CDCl_3_) δ 165.2, 165.0, 160.8 (d, *J**_C–F_* = 248.6 Hz), 158.5, 135.7, 131.4 (d, *J_C–F_* = 8.3 Hz), 130.4 (d, *J_C–F_* = 3.3 Hz), 124.9 (d, *J_C–F_* = 3.7 Hz), 121.5 (d, *J_C–F_* = 14.2 Hz), 116.0 (d, *J_C–F_* = 21.1 Hz), 110.9, 107.2, 99.8, 65.9 (d, *J_C–F_* = 4.1 Hz), 55.8; ^19^F NMR (377 MHz, CDCl_3_) δ -117.62 – -117.95 (m); HRMS (ESI-TOF, *m/z*): calcd for C_15_H_13_FO_4_ [M + H]^+^, 277.0871; found, 277.0873.

2-((3-Fluorobenzyl)oxy)-4-methoxybenzoic acid (1m)**.** ^1^H NMR (400 MHz, CDCl_3_): δ 8.23–8.13 (m, 1H), 7.42 (dd, *J* = 13.9, 7.7 Hz, 1H), 7.23 (d, *J* = 7.6 Hz, 1H), 7.20–7.07 (m, 2H), 6.67 (d, *J* = 8.8 Hz, 1H), 6.57 (d, *J* = 2.2 Hz, 1H), 5.25 (s, 2H), 3.86 (s, 3H); ^13^C NMR (101 MHz, CDCl_3_): δ 166.0, 165.0, 163.0 (d, *J_C–F_* = 247.5 Hz), 158.9, 137.1 (d, *J_C–F_* = 7.3 Hz), 135.6, 130.8 (d, *J_C–F_* = 8.2 Hz), 123.2 (d, *J_C–F_* = 3.0 Hz), 115.9 (d, *J_C–F_* = 21.1 Hz), 114.6 (d, *J_C–F_* = 22.3 Hz), 110.9, 106.8, 100.1, 71.0 (d, *J_C–F_* = 1.5 Hz), 55.8 (s); ^19^F NMR (377 MHz, CDCl_3_) δ –111.67 (dd, *J* = 14.4, 8.5 Hz); HRMS (ESI-TOF, *m/z*): calcd for C_15_H_13_FO_4_ [M + H]^+^, 277.0871; found, 277.0870.

2-((3-Chlorobenzyl)oxy)-4-methoxybenzoic acid (1n)**.** ^1^H NMR (400 MHz, CDCl_3_): δ 8.19 (d, *J* = 8.8 Hz, 1H), 7.48–7.32 (m, 4H), 6.70 (dd, *J* = 8.8, 2.3 Hz, 1H), 6.58 (d, *J* = 2.2 Hz, 1H), 5.25 (s, 2H), 3.89 (s, 3H); ^13^C NMR (101 MHz, CDCl_3_): δ 165.0, 165.95, 158.5, 136.3, 135.8, 135.1, 130.6, 129.4, 128.0, 125.9, 110.9, 107.0, 100.1, 71.3, 55.8; HRMS (ESI-TOF, *m/z*): calcd for C_15_H_13_ClO_4_ [M + H]^+^, 293.0575; found, 293.0576.

4-Methoxy-2-((3-(trifluoromethyl)benzyl)oxy)benzoic acid (1o)**.** ^1^H NMR (400 MHz, CDCl_3_): δ 8.19 (d, *J* = 8.8 Hz, 1H), 7.78–7.58 (m, 4H), 6.71 (dd, *J* = 8.8, 2.2 Hz, 1H), 6.60 (d, *J* = 2.2 Hz, 1H), 5.33 (s, 2H), 3.89 (s, 3H); ^13^C NMR (101 MHz, CDCl_3_): δ 165.9, 165.1, 158.8, 135.8, 135.7, 131.5 (q, *J_C–F_* = 32.7 Hz), 130.9, 129.7, 125.8 (q, *J_C–F_* = 3.7 Hz), 124.5 (q, *J_C–F_* = 3.8 Hz), 123.8 (q, *J_C–F_* = 270.7 Hz), 111.0, 106.9, 100.1, 71.1, 55.8; ^19^F NMR (377 MHz, CDCl_3_): δ –62.72; HRMS (ESI-TOF, *m/z*): calcd for C_16_H_13_F_3_O_4_ [M + H]^+^, 327.0839; found, 327.0843.

4-Methoxy-2-((3-methylbenzyl)oxy)benzoic acid (1p)**.** ^1^H NMR (400 MHz, CDCl_3_): δ 8.17 (t, *J* = 7.0 Hz, 1H), 7.32 (t, *J* = 7.5 Hz, 1H), 7.26–7.17 (m, 3H), 6.70–6.58 (m, 2H), 5.21 (s, 2H), 3.86 (s, 3H), 2.38 (s, 3H); ^13^C NMR (101 MHz, CDCl_3_): δ 165.1, 165.0, 158.8, 139.0, 135.7, 134.1, 130.0, 129.1, 128.7, 125.1, 110.8, 106.9, 100.0, 72.3, 55.8, 21.4; HRMS (ESI-TOF, *m/z*): calcd for C_16_H_16_O_4_ [M + H]^+^, 273.1122; found, 273.1124.

4-Methoxy-2-((3-methoxybenzyl)oxy)benzoic acid (1q)**.** ^1^H NMR (400 MHz, CDCl_3_): δ 10.62 (s, 1H), 8.18 (d, *J* = 8.8 Hz, 1H), 7.36 (t, *J* = 7.9 Hz, 1H), 7.09–6.88 (m, 3H), 6.73–6.58 (m, 2H), 5.25 (s, 2H), 3.88 (s, 3H), 3.85 (s, 3H); ^13^C NMR (101 MHz, CDCl_3_): δ 165.2, 165.0, 160.2, 158.7, 135.7, 135.7, 130.3, 120.0, 114.7, 113.3, 110.8, 107.0, 100.0, 72.1, 55.8, 55.4; HRMS (ESI-TOF, *m/z*): calcd for C_16_H_16_O_5_ [M + H]^+^, 289.1071; found, 289.1068.

2-((3,5-Dimethylbenzyl)oxy)-4-methoxybenzoic acid (1r)**.** ^1^H NMR (400 MHz, CDCl_3_): δ 8.17 (t, *J* = 7.5 Hz, 1H), 7.04 (s, 3H), 6.72–6.57 (m, 2H), 5.17 (s, 2H), 3.87 (s, 3H), 2.34 (s, 6H); ^13^C NMR (101 MHz, CDCl_3_): δ 165.1, 165.0, 158.8, 138.9, 135.6, 134.0, 130.9, 125.8, 110.8, 106.9, 99.9, 72.4, 55.8, 21.3; HRMS (ESI-TOF, *m/z*): calcd for C_17_H_18_O_4_ [M + H]^+^, 287.1278; found, 287.1279.

2-((3,5-Dimethoxybenzyl)oxy)-4-methoxybenzoic acid (1s)**.** ^1^H NMR (400 MHz, CDCl_3_): δ 8.13 (d, *J* = 8.8 Hz, 1H), 6.64 (dd, *J* = 8.8, 2.2 Hz, 1H), 6.57 (t, *J* = 2.2 Hz, 3H), 6.45 (t, *J* = 2.1 Hz, 1H), 5.17 (s, 2H), 3.83 (d, *J* = 8.8 Hz, 3H), 3.79 (s, 6H); ^13^C NMR (101 MHz, CDCl_3_): δ 165.5, 165.0, 161.4, 158.9, 136.7, 135.6, 110.8, 106.9, 105.5, 100.8, 100.0, 72.0, 55.8, 55.5; HRMS (ESI-TOF, *m/z*): calcd for C_17_H_18_O_6_ [M + H]^+^, 319.1176; found, 319.1187.

4-Methoxy-2-(naphthalen-1-ylmethoxy)benzoic acid (1t)**.** ^1^H NMR (400 MHz, CDCl_3_): δ 10.41 (s, 1H), 8.21 (d, *J* = 8.8 Hz, 1H), 8.04–7.92 (m, 3H), 7.66–7.48 (m, 4H), 6.80 (d, *J* = 2.2 Hz, 1H), 6.72 (dd, *J* = 8.8, 2.2 Hz, 1H), 5.70 (s, 2H), 3.92 (s, 3H); ^13^C NMR (101 MHz, CDCl_3_): δ 165.1, 165.0, 158.8, 135.8, 134.0, 131.3, 130.5, 129.5, 129.1, 127.9, 127.4, 126.5, 125.3, 122.8, 110.8, 106.9, 99.9, 70.7, 55.8; HRMS (ESI-TOF, *m/z*): calcd for C_19_H_16_O_4_ [M + H]^+^, 309.1122; found, 309.1121.

2-(Benzyl(*tert*-butoxycarbonyl)amino)benzoic acid (1u)**.** ^1^H NMR (400 MHz, CDCl_3_): δ 8.10–7.99 (m, 1H), 7.44 (t, *J* = 7.1 Hz, 1H), 7.39–7.22 (m, 7H), 7.00 (d, *J* = 7.7 Hz, 1H), 5.27 (s, 1H), 4.34 (s, 1H), 1.43 (s, 9H); ^13^C NMR (101 MHz, CDCl_3_): δ 170.5, 155.0, 142.9, 138.3, 133.4, 131.8, 129.5, 128.4, 127.7, 127.3, 127.0, 80.8, 54.3, 28.2; HRMS (ESI-TOF, *m/z*): calcd for C_19_H_21_NO_4_ [M + H]^+^, 328.1544; found, 328.1541.

4-(Benzyloxy)-2',4'-difluoro-[1,1'-biphenyl]-3-carboxylic acid (1v)**.** ^1^H NMR (400 MHz, CDCl_3_): δ 10.80 (s, 1H), 8.37 (d, *J* = 1.9 Hz, 1H), 7.75 (dt, *J* = 8.6, 2.1 Hz, 1H), 7.53–7.40 (m, 6H), 7.24 (d, *J* = 8.7 Hz, 1H), 7.04–6.90 (m, 2H), 5.38 (s, 2H), 1.58 (s, 2H); ^13^C NMR (101 MHz, CDCl_3_): δ 165.1, 162.5 (dd, *J_C–F_* = 249.8, 11.8 Hz), 159.7 (dd, *J_C–F_* = 250.6, 11.9 Hz), 156.9, 135.4 (d, *J_C–F_* = 4.1 Hz), 134.2, 134.1 (d, *J_C–F_* = 1.7 Hz), 131.3 (dd, *J_C–F_* = 9.5, 4.7 Hz), 129.4, 129.3, 129.2, 128.0, 123.3 (dd, *J_C–F_* = 13.5, 3.9 Hz), 118.3, 113.3, 111.8 (dd, *J_C–F_* = 21.2, 3.8 Hz), 104.5 (dd, *J_C–F_* = 26.4, 25.5 Hz), 72.5; ^19^F NMR (377 MHz, CDCl_3_): δ -110.40 – -110.54 (m), -113.63 (dd, *J* = 17.9, 8.9 Hz); HRMS (ESI-TOF, *m/z*): calcd for C_20_H_14_F_2_O_3_ [M + H]^+^, 341.0984; found, 341.0981.

(*R*)-4-Methoxy-2-(1-phenylethoxy)benzoic acid (1w)**.** ^1^H NMR (400 MHz, CDCl_3_): δ 10.98 (s, 1H), 8.14 (d, *J* = 8.8 Hz, 1H), 7.48–7.31 (m, 5H), 6.60 (dd, *J* = 8.8, 2.3 Hz, 1H), 6.41 (d, *J* = 2.3 Hz, 1H), 5.56 (q, *J* = 6.4 Hz, 1H), 3.75 (s, 3H), 1.82 (d, *J* = 6.5 Hz, 3H); ^13^C NMR (101 MHz, CDCl_3_): δ 165.3, 164.7, 157.9, 140.4, 135.4, 129.2, 128.6, 125.4, 111.1, 107.1, 101.2, 79.4, 55.6, 24.1; HRMS (ESI-TOF, *m/z*): calcd for C_16_H_16_O_4_ [M + H]^+^, 273.1122; found, 273.1125.

*N*-(*tert*-Butyl)-*N*-(2,6-dimethylbenzyl)acrylamide (6b)**.** ^1^H NMR (400 MHz, CDCl_3_): δ 7.06 (dd, *J* = 8.4, 6.5 Hz, 1H), 6.98 (d, *J* = 7.5 Hz, 2H), 6.58 (dd, *J* = 16.7, 10.4 Hz, 1H), 6.23 (dd, *J* = 16.7, 2.0 Hz, 1H), 5.56 (dd, *J* = 10.4, 2.0 Hz, 1H), 4.70 (s, 2H), 2.35 (s, 6H), 1.39 (s, 9H); ^13^C NMR (101 MHz, CDCl_3_): δ 169.3, 136.2, 135.3, 132.8, 129.7, 126.8, 126.2, 58.0, 46.8, 28.4, 20.8; HRMS (ESI-TOF, *m/z*): calcd for C_16_H_23_NO [M + H]^+^, 246.1853; found, 246.1852.

*N*-(*tert*-Butyl)-*N*-(3,5-dimethoxybenzyl)acrylamide (6c)**.** ^1^H NMR (400 MHz, CDCl_3_): δ 6.46–6.25 (m, 5H), 5.57–5.50 (m, 1H), 4.56 (s, 2H), 3.78 (s, 6H), 1.47 (s, 9H); ^13^C NMR (101 MHz, CDCl_3_): δ 168.6, 161.3, 142.2, 131.6, 127.2, 103.7, 98.8, 57.7, 55.3, 49.0, 28.5; HRMS (ESI-TOF, *m/z*): calcd for C_16_H_23_NO_3_ [M + H]^+^, 278.1751; found, 278,1749.

The characterization data of the other starting materials were the same as reported elsewhere (**S1**^10^, **1c**^11^, **1e**^12^**_,_ 1f**^13^**_,_ 1g**^14^ **1i**^5^**_,_ 1j**^15^**_,_ 1d**^16^, **S1**,^8^ **S2**,^8^ **6a**^9^).

Spiro[chromane-3,1'-cyclohexane]-2',5'-dien-4-one (5a)**.** The product was obtained as a white solid (isolated yield: 65%). ^1^H NMR (400 MHz, CDCl_3_): δ 7.99–7.86 (m, 1H), 7.56–7.44 (m, 1H), 7.11–6.93 (m, 2H), 6.16–6.01 (m, 2H), 5.69 (dt, *J* = 10.4, 1.9 Hz, 2H), 4.27 (s, 2H), 2.94–2.69 (m, 2H); ^13^C NMR (101 MHz, CDCl_3_): δ 193.1, 161.1, 135.9, 128.7, 128.2, 122.2, 121.7, 119.7, 117.7, 75.2, 48.3, 26.8; HRMS (ESI-TOF, *m/z*): calcd for C_14_H_12_O_2_ [M + H]^+^, 213.0910; found, 213.0914.

7-Methoxyspiro[chromane-3,1'-cyclohexane]-2',5'-dien-4-one (5b)**.** The product was obtained as a white solid (isolated yield: 75%). ^1^H NMR (400 MHz, CDCl_3_): δ 7.85 (dd, *J* = 8.7, 6.0 Hz, 1H), 6.60 (dt, *J* = 15.9, 7.9 Hz, 1H), 6.43 (d, *J* = 2.4 Hz, 1H), 6.13–6.00 (m, 2H), 5.68 (dt, *J* = 10.5, 2.0 Hz, 2H), 4.26 (s, 2H), 3.85 (s, 3H), 2.91–2.67 (m, 2H); ^13^C NMR (101 MHz, CDCl_3_): δ 192.0, 166.0, 163.1, 129.9, 128.5, 122.6, 113.5, 110.3, 100.5, 75.6, 55.7, 48.0, 26.8; HRMS (ESI-TOF, *m/z*): calcd for C_15_H_14_O_3_ [M + H]^+^, 243.1016; found, 243.1018.

7-Methylspiro[chromane-3,1'-cyclohexane]-2',5'-dien-4-one (5c)**.** The product was obtained as a white solid (isolated yield: 73%). ^1^H NMR (400 MHz, CD_3_CN): δ 7.73 (d, *J* = 8.0 Hz, 1H), 6.97–6.87 (m, 2H), 6.08 (dtd, *J* = 10.6, 3.4, 1.8 Hz, 2H), 5.67 (dp, *J* = 10.5, 1.9 Hz, 2H), 4.28 (s, 2H), 2.77 (ttd, *J* = 3.1, 2.0, 0.8 Hz, 2H), 2.39 (s, 3H); ^13^C NMR (126 MHz, CD_3_CN): δ 191.9, 160.7, 147.3, 127.8, 126.9, 122.5, 122.0, 117.1, 74.5, 47.6, 25.9, 20.4; HRMS (ESI-TOF, *m/z*): calcd for C_15_H_14_O_2_ [M + H]^+^, 227.1067; found, 227.1064.

8-Phenylspiro[chromane-3,1'-cyclohexane]-2',5'-dien-4-one (5d)**.** The product was obtained as a white solid (isolated yield: 66%). ^1^H NMR (500 MHz, CDCl_3_): δ 7.96 (dd, *J* = 7.9, 1.8 Hz, 1H), 7.60–7.51 (m, 3H), 7.49–7.42 (m, 2H), 7.42–7.35 (m, 1H), 7.12 (t, *J* = 7.6 Hz, 1H), 6.09 (dt, *J* = 10.2, 3.4 Hz, 2H), 5.74 (dt, *J* = 10.4, 2.0 Hz, 2H), 4.28 (s, 2H), 2.92–2.71 (m, 2H); ^13^C NMR (126 MHz, CDCl_3_): δ 193.5, 158.2, 136.9, 131.2, 129.6, 128.9, 128.4, 127.83, 127.75, 122.3, 121.7, 120.3, 75.3, 48.2, 26.9; HRMS (ESI-TOF, *m/z*): calcd for C_20_H_16_O_2_ [M + H]^+^, 289.1223; found, 289.1222.

*N*-(4-Oxospiro[chromane-3,1'-cyclohexane]-2',5'-dien-7-yl)acetamide (5e)**.** The product was obtained as a white solid (isolated yield: 60%). ^1^H NMR (400 MHz, CDCl_3_): δ 7.89–7.78 (m, 2H), 7.51 (s, 1H), 6.95 (dd, *J* = 8.6, 2.0 Hz, 1H), 6.11–6.01 (m, 2H), 5.67 (dt, *J* = 10.4, 1.9 Hz, 2H), 4.25 (s, 2H), 2.89–2.67 (m, 21H), 2.21 (s, 3H); ^13^C NMR (101 MHz, CDCl_3_): δ 192.4, 168.8, 162.4, 144.8, 129.2, 128.7, 122.3, 115.7, 113.0, 107.2, 75.4, 48.1, 26.8, 24.8; HRMS (ESI-TOF, *m/z*): calcd for C_16_H_15_NO_3_ [M + H]^+^, 270.1125; found, 270.1128.

8-Methoxyspiro[chromane-3,1'-cyclohexane]-2',5'-dien-4-one (5f)**.** The product was obtained as a white solid (isolated yield: 71%). ^1^H NMR (400 MHz, CDCl_3_): δ 7.50 (d, *J* = 7.7 Hz, 1H), 7.09–6.90 (m, 2H), 6.06 (d, *J* = 10.1 Hz, 2H), 5.68 (d, *J* = 10.1 Hz, 2H), 4.34 (s, 2H), 3.91 (s, 3H), 2.91–2.64 (m, 2H); ^13^C NMR (101 MHz, CDCl_3_): δ 193.0, 151.0, 148.6, 128.8, 122.1, 121.1, 120.3, 119.3, 116.5, 75.7, 56.2, 48.1, 26.8; HRMS (ESI-TOF, *m/z*): calcd for C_15_H_14_O_3_ [M + H]^+^, 243.1016; found, 243.1018.

6-Methoxyspiro[chromane-3,1'-cyclohexane]-2',5'-dien-4-one (5g)**.** The product was obtained as a white solid (isolated yield: 66%). ^1^H NMR (400 MHz, CD_3_CN): δ 7.27 (d, *J* = 3.2 Hz, 1H), 7.17 (dd, *J* = 9.1, 3.2 Hz, 1H), 6.99 (d, *J* = 9.0 Hz, 1H), 6.12–6.03 (m, 2H), 5.66 (dp, *J* = 10.6, 2.0 Hz, 2H), 4.24 (s, 2H), 3.78 (s, 3H), 2.78–2.71 (m, 2H); ^13^C NMR (126 MHz, CD_3_CN): δ 193.4, 156.4, 155.0, 129.1, 125.3, 123.1, 120.2, 119.7, 108.9, 75.8, 56.1, 48.8, 27.0; HRMS (ESI-TOF, *m/z*): calcd for C_15_H_14_O_3_ [M + H]^+^, 243.1016; found, 243.1019.

5-Methoxyspiro[chromane-3,1'-cyclohexane]-2',5'-dien-4-one (5h)**.** The product was obtained as a white solid (isolated yield: 15%). ^1^H NMR (400 MHz, CDCl_3_): δ 7.32 (t, *J* = 8.4 Hz, 1H), 6.51 (dd, *J* = 8.4, 0.8 Hz, 1H), 6.45 (d, *J* = 8.3 Hz, 1H), 6.02–5.94 (m, 2H), 5.62 (dt, *J* = 10.4, 1.9 Hz, 2H), 4.16 (s, 2H), 3.81 (s, 3H), 2.80–2.61 (m, 2H); ^13^C NMR (101 MHz, CDCl_3_): δ 191.8, 162.6, 161.5, 135.8, 128.3, 122.8, 115.0, 109.7, 103.9, 74.7, 56.1, 48.8, 26.8; HRMS (ESI-TOF, *m/z*): calcd for C_15_H_14_O_3_ [M + H]^+^, 243.1016; found, 243.1017.

7-Chlorospiro[chromane-3,1'-cyclohexane]-2',5'-dien-4-one (5i)**.** The product was obtained as a white solid (isolated yield: 31%). ^1^H NMR (400 MHz, CD_3_CN): δ 7.83 (d, *J* = 8.4 Hz, 1H), 7.19–7.06 (m, 2H), 6.10 (dtd, *J* = 10.6, 3.4, 1.8 Hz, 2H), 5.68 (dp, *J* = 10.5, 1.9 Hz, 2H), 4.34 (s, 2H), 2.78 (ttd, *J* = 3.3, 2.1, 1.1 Hz, 2H); ^13^C NMR (126 MHz, CD_3_CN): δ 191.5, 161.3, 140.8, 128.8, 128.4, 121.9, 121.6, 118.2, 117.4, 75.0, 47.7, 26.0; HRMS (ESI-TOF, *m/z*): calcd for C_14_H_11_ClO_2_ [M + H]^+^, 247.0521; found, 247.0525.

6-Acetylspiro[chromane-3,1'-cyclohexane]-2',5'-dien-4-one (5j)**.** The product was obtained as a white solid (isolated yield: 23%). ^1^H NMR (400 MHz, CDCl_3_): δ 8.48 (d, *J* = 2.2 Hz, 1H), 8.20–8.11 (m, 1H), 7.07 (d, *J* = 8.8 Hz, 1H), 6.17–6.02 (m, 2H), 5.67 (dd, *J* = 8.6, 1.7 Hz, 2H), 4.34 (s, 2H), 2.91–2.72 (m, 2H), 2.60 (s, 3H); ^13^C NMR (101 MHz, CDCl_3_): δ 196.3, 192.3, 164.4, 135.2, 131.2, 129.7, 129.2, 121.6, 118.9, 118.5, 75.4, 48.1, 26.8, 26.4; HRMS (ESI-TOF, *m/z*): calcd for C_16_H_14_O_3_ [M + H]^+^, 255.1016; found, 255.1020.

6-Bromo-7-methylspiro[chromane-3,1'-cyclohexane]-2',5'-dien-4-one (5k)**.** The product was obtained as a white solid (isolated yield: 30%). ^1^H NMR (500 MHz, CD_3_CN): δ 7.92 (s, 1H), 7.02 (s, 1H), 6.10–6.03 (m, 2H), 5.63 (dp, *J* = 10.6, 2.0 Hz, 2H), 4.27 (s, 2H), 2.74 (qd, *J* = 3.4, 1.7 Hz, 2H), 2.40 (s, 3H); ^13^C NMR (126 MHz, CD_3_CN): δ 192.1, 160.9, 147.4, 131.1, 129.3, 122.7, 120.6, 119.9, 117.4, 75.8, 48.6, 27.0, 23.3; HRMS (ESI-TOF, *m/z*): calcd for C_15_H_13_BrO_2_ [M + Na]^+^, 326.9991; found, 326.9973.

2'-Fluoro-7-methoxyspiro[chromane-3,1'-cyclohexane]-2',5'-dien-4-one (5l)**.** The product was obtained as a white solid (isolated yield: 45%). ^1^H NMR (400 MHz, CDCl_3_): δ 7.91–7.87 (m, 1H), 6.62 (dd, *J* = 8.9, 2.4 Hz, 1H), 6.44 (d, *J* = 2.4 Hz, 1H), 5.96–5.89 (m, 1H), 5.82–5.74 (m, 1H), 5.64 (dtd, *J* = 17.7, 3.6, 1.0 Hz, 1H), 4.70 (d, *J* = 11.0 Hz, 1H), 4.39 (d, *J* = 11.0 Hz, 1H), 3.85 (s, 3H), 3.04–2.81 (m, 2H); ^13^C NMR (101 MHz, CDCl_3_): δ 188.4 (d, *J_C–F_* = 2.2 Hz), 166.3, 163.1, 154.7 (d, *J_C–F_* = 254.1 Hz), 130.1, 127.3 (d, *J_C–F_* = 2.3 Hz), 122.4 (d, *J_C–F_* = 5.3 Hz), 113.7, 110.6, 104.9 (d, *J_C–F_* = 15.8 Hz), 100.6, 73.5, 55.7, 50.8 (d, *J_C–F_* = 23.3 Hz), 26.4 (d, *J_C–F_* = 6.8 Hz); ^19^F NMR (377 MHz, CD_3_CN): δ -117.91 (ddt, J = 23.7, 8.9, 4.2 Hz).; HRMS (ESI-TOF, *m/z*): calcd for C_15_H_13_FO_3_ [M + H]^+^, 261.0922; found, 261.0923.

3'-Fluoro-7-methoxyspiro[chromane-3,1'-cyclohexane]-2',5'-dien-4-one (5m)**.** The product was obtained as a white solid (isolated yield: 73%). ^1^H NMR (400 MHz, CDCl_3_): δ 7.76 (d, *J* = 8.8 Hz, 1H), 6.52 (dt, *J* = 12.5, 6.3 Hz, 1H), 6.38 (dd, *J* = 20.9, 2.1 Hz, 1H), 5.90 (ddt, *J* = 10.6, 7.1, 3.4 Hz, 1H), 5.57 (d, *J* = 9.9 Hz, 1H), 5.15 (d, *J* = 16.5 Hz, 1H), 4.25–4.11 (m, 2H), 3.77 (s, 3H), 2.98–2.77 (m, 2H); ^13^C NMR (101 MHz, CDCl_3_): δ 190.9 (d, *J_C–F_* = 2.1 Hz), 166.2, 163.0, 160.4 (d, *J_C–F_* = 259.6 Hz), 129.9, 126.3 (d, *J_C–F_* = 10.6 Hz), 122.9 (d, *J_C–F_* = 2.6 Hz), 113.2, 110.6, 100.5, 99.4 (t, *J_C–F_* = 14.8 Hz), 75.4 (d, *J_C–F_* = 2.5 Hz), 55.7, 50.4 (d, *J_C–F_* = 7.0 Hz), 27.4 (d, *J_C–F_* = 26.5 Hz); ^19^F NMR (377 MHz, CDCl_3_): δ –100.28 (dd, *J* = 16.1, 6.6 Hz); HRMS (ESI-TOF, *m/z*): calcd for C_15_H_13_FO_3_ [M + H]^+^, 261.0922; found, 261.0920.

3'-Chloro-7-methoxyspiro[chromane-3,1'-cyclohexane]-2',5'-dien-4-one (5n)**.** The product was obtained as a white solid (isolated yield: 63%). ^1^H NMR (400 MHz, CDCl_3_): δ 7.74 (t, *J* = 11.8 Hz, 1H), 6.54 (dd, *J* = 8.8, 2.3 Hz, 1H), 6.36 (d, *J* = 2.2 Hz, 1H), 5.93 (dt, *J* = 10.0, 3.4 Hz, 1H), 5.74 (d, *J* = 1.3 Hz, 1H), 5.57 (dd, *J* = 10.1, 1.8 Hz, 1H), 4.19 (s, 2H), 3.77 (s, 3H), 3.09–2.85 (m, 2H); ^13^C NMR (101 MHz, CDCl_3_): δ 190.3, 166.2, 163.0, 133.9, 129.9, 127.6, 122.0, 120.0, 113.2, 110.7, 100.6, 75.0, 55.7, 51.2, 34.1; HRMS (ESI-TOF, *m/z*): calcd for C_15_H_13_ClO_3_ [M + H]^+^, 277.0626; found, 277.0629.

7-Methoxy-3'-(trifluoromethyl)spiro[chromane-3,1'-cyclohexane]-2',5'-dien-4-one (5o)**.** The product was obtained as a white solid (isolated yield: 30%). ^1^H NMR (400 MHz, CDCl_3_): δ 7.80–7.74 (m, 1H), 6.59–6.53 (m, 1H), 6.39 (t, *J* = 6.5 Hz, 1H), 6.22 (d, *J* = 1.4 Hz, 1H), 6.05 (dt, *J* = 10.1, 3.5 Hz, 1H), 5.60 (ddd, *J* = 10.1, 4.0, 1.9 Hz, 1H), 4.22 (s, 2H), 3.78 (s, 3H), 2.98–2.73 (m, 2H); ^13^C NMR (101 MHz, CDCl_3_): δ 188.7, 165.4, 162.0, 128.9, 128.7 (q, *J_C–F_* = 30.7 Hz), 125.8, 124.4 (q, *J_C–F_* = 5.7 Hz), 122.2 (q, *J_C–F_* = 270.8 Hz), 120.8, 112.1, 109.8, 99.6, 73.6, 54.7, 47.6, 23.1; ^19^F NMR (377 MHz, CDCl_3_): δ –69.78; HRMS (ESI-TOF, *m/z*): calcd for C_16_H_13_F_3_O_3_ [M + H]^+^, 311.089; found, 311.089.

7-Methoxy-3'-methylspiro[chromane-3,1'-cyclohexane]-2',5'-dien-4-one (5p)**.** The product was obtained as a white solid (isolated yield: 74%). ^1^H NMR (500 MHz, CDCl_3_): δ 7.85 (d, *J* = 8.8 Hz, 1H), 6.60 (dd, *J* = 8.8, 2.4 Hz, 1H), 6.43 (d, *J* = 2.5 Hz, 1H), 6.07 (dt, *J* = 10.1, 3.4 Hz, 1H), 5.64 (dq, *J* = 10.1, 2.1 Hz, 1H), 5.40 (p, *J* = 1.6 Hz, 1H), 4.27–4.18 (m, 2H), 3.85 (s, 3H), 2.79–2.59 (m, 2H), 1.77 (s, 4H); ^13^C NMR (101 MHz, CDCl_3_): δ 192.4, 165.9, 163.1, 136.2, 129.9, 128.7, 122.3, 117.0, 113.7, 110.2, 100.4, 75.8, 55.6, 49.2, 31.6, 23.4; HRMS (ESI-TOF, *m/z*): calcd for C_16_H_16_O_3_ [M + H]^+^, 257.1172; found, 257.1172.

3',7-Dimethoxyspiro[chromane-3,1'-cyclohexane]-2',5'-dien-4-one (5q)**.** The product was obtained as a white solid (isolated yield: 70%). ^1^H NMR (400 MHz, CD_3_CN): δ 7.78 (d, *J* = 8.8 Hz, 1H), 6.67 (dd, *J* = 8.8, 2.4 Hz, 1H), 6.55 (d, *J* = 2.4 Hz, 1H), 6.00 (dt, *J* = 10.0, 3.5 Hz, 1H), 5.65 (dq, *J* = 10.0, 2.0 Hz, 1H), 4.65 (q, *J* = 1.4 Hz, 1H), 4.35–4.21 (m, 2H), 3.87 (s, 3H), 3.55 (s, 3H), 2.79 (ddd, *J* = 3.5, 2.2, 1.1 Hz, 2H); ^13^C NMR (101 MHz, CD_3_CN): δ 192.5, 166.6, 163.7, 157.0, 129.9, 127.6, 123.6, 114.0, 110.7, 101.2, 90.9, 76.6, 56.2, 54.4, 50.3, 29.5; HRMS (ESI-TOF, *m/z*): calcd for C_16_H_16_O_4_ [M + H]^+^, 273.1122; found, 273.1126.

7-Methoxy-3',5'-dimethylspiro[chromane-3,1'-cyclohexane]-2',5'-dien-4-one (5r)**.** The product was obtained as a white solid (isolated yield: 65%). ^1^H NMR (500 MHz, CDCl_3_): δ 7.85 (d, *J* = 8.8 Hz, 1H), 6.60 (dd, *J* = 8.8, 2.4 Hz, 1H), 6.43 (d, *J* = 2.4 Hz, 1H), 5.39–5.33 (m, 2H), 4.19 (s, 2H), 3.84 (s, 3H), 2.70–2.48 (m, 2H), 1.78 (s, 6H); ^13^C NMR (101 MHz, CDCl_3_): δ 193.0, 166.0, 163.2, 136.5, 130.0, 116.9, 113.9, 110.3, 100.6, 76.2, 55.8, 50.5, 36.7, 23.2; HRMS (ESI-TOF, *m/z*): calcd for C_17_H_18_O_3_ [M + H]^+^, 271.1329; found, 271.1330.

3',5',7-Trimethoxyspiro[chromane-3,1'-cyclohexane]-2',5'-dien-4-one (5s)**.** The product was obtained as a white solid (isolated yield: 78%). ^1^H NMR (400 MHz, CD_3_CN): δ 7.78 (dd, *J* = 8.8, 4.3 Hz, 1H), 6.67 (dd, *J* = 8.8, 2.4 Hz, 1H), 6.54 (dd, *J* = 7.0, 2.4 Hz, 1H), 4.66 (s, 2H), 4.25 (s, 2H), 3.87 (d, *J* = 3.2 Hz, 3H), 3.55 (s, 6H), 2.93–2.73 (m, 2H); ^13^C NMR (101 MHz, CD_3_CN): δ 193.1, 166.5, 163.8, 155.8, 130.0, 114.1, 110.6, 101.2, 91.1, 77.2, 56.2, 54.8, 50.0, 31.9; HRMS (ESI-TOF, *m/z*): calcd for C_17_H_18_O_5_ [M + H]^+^, 303.1227; found, 303.1232.

7-Methoxy-4'H-spiro[chromane-3,1'-naphthalen]-4-one (5t)**.** The product was obtained as a white solid (isolated yield: 58%). ^1^H NMR (400 MHz, CDCl_3_): δ 7.84 (d, *J* = 8.8 Hz, 1H), 7.22–6.98 (m, 4H), 6.55 (dd, *J* = 8.8, 2.1 Hz, 1H), 6.40 (d, *J* = 2.1 Hz, 1H), 6.14 (dt, *J* = 9.9, 3.5 Hz, 1H), 5.86 (d, *J* = 10.1 Hz, 1H), 4.53 (d, *J* = 11.1 Hz, 1H), 4.26 (d, *J* = 11.1 Hz, 1H), 3.77 (s, 3H), 3.43 (q, *J* = 21.8 Hz, 2H); ^13^C NMR (101 MHz, CDCl_3_): δ 192.2, 166.1, 163.4, 134.9, 133.0, 130.1, 128.8, 128.6, 127.5, 127.4, 126.5, 123.8, 114.7, 110.5, 100.7, 76.2, 55.7, 51.0, 30.2; HRMS (ESI-TOF, *m/z*): calcd for C_19_H_16_O_3_ [M + H]^+^, 293.1172; found, 293.1172.

*tert*-Butyl 4'-oxo-2'H-spiro[cyclohexane-1,3'-quinoline]-2,5-diene-1'(4'H)-carboxylate (5u)**.** The product was obtained as a white solid (isolated yield: 23%). ^1^H NMR (400 MHz, CDCl_3_): δ 7.95 (dd, *J* = 7.9, 1.6 Hz, 1H), 7.76 (d, *J* = 8.4 Hz, 1H), 7.44 (ddd, *J* = 8.7, 7.2, 1.7 Hz, 1H), 7.11–7.05 (m, 1H), 6.03–5.95 (m, 2H), 5.57 (dt, *J* = 10.4, 1.9 Hz, 2H), 3.94 (s, 2H), 2.85–2.64 (m, 2H), 1.48 (s, 9H); ^13^C NMR (101 MHz, CDCl_3_): δ 195.2, 153.5, 143.8, 134.0, 128.5, 128.2, 123.9, 123.4, 123.1, 123.0, 82.2, 54.6, 49.3, 28.2, 26.8; HRMS (ESI-TOF, *m/z*): calcd for C_19_H_21_NO_3_ [M + H]^+^, 312.1594; found, 312.1605.

7-(2,4-Difluorophenyl)spiro[chromane-3,1'-cyclohexane]-2',5'-dien-4-one (5v)**.** The product was obtained as a white solid (isolated yield: 56%). ^1^H NMR (400 MHz, CD_3_CN): δ 7.98 (dd, *J* = 2.4, 1.5 Hz, 1H), 7.75 (ddd, *J* = 8.7, 2.4, 1.7 Hz, 1H), 7.59–7.51 (m, 1H), 7.17 (d, *J* = 8.7 Hz, 1H), 7.14–7.04 (m, 2H), 6.17–6.08 (m, 2H), 5.72 (dp, *J* = 10.5, 1.9 Hz, 2H), 4.37 (s, 2H), 2.79 (ttd, *J* = 3.3, 2.0, 1.1 Hz, 2H); ^13^C NMR (101 MHz, CD_3_CN): δ 193.1, 162.9 (dd, *J_C–F_* = 247.1, 12.3 Hz), 161.3, 160.2 (dd, *J_C–F_* = 247.1, 12.3 Hz), 137.0 (d, *J_C–F_* = 3.0 Hz), 132.2 (dd, *J_C–F_* = 9.8, 4.8 Hz), 129.2, 128.8, 128.2 (d, *J_C–F_* = 2.9 Hz), 124.5 (dd, *J_C–F_* = 13.5, 3.9 Hz), 122.7, 120.2, 118.7, 112.4 (dd, *J_C–F_* = 21.3, 3.9 Hz), 104.8 (dd, *J_C–F_* = 27.2, 25.7 Hz), 75.7, 48.7, 27.0; ^19^F NMR (377 MHz, CD_3_CN): δ –112.93 (p, *J* = 8.1 Hz), –115.39 (q, *J* = 10.1 Hz); HRMS (ESI-TOF, *m/z*): calcd for C_20_H_14_F_2_O_2_ [M + H]^+^, 325.1035; found, 325.1027.

(*R*)-7-Methoxy-2-methylspiro[chromane-3,1'-cyclohexane]-2',5'-dien-4-one (5w)**.** The product was obtained as a white solid (isolated yield: 47%). ^1^H NMR (400 MHz, CDCl_3_): δ 7.83 (d, *J* = 8.8 Hz, 1H), 6.59 (dd, *J* = 8.8, 2.4 Hz, 1H), 6.43 (d, *J* = 2.3 Hz, 1H), 6.19–6.11 (m, 1H), 6.06 (d, *J* = 10.2 Hz, 1H), 5.64 (dd, *J* = 10.2, 2.1 Hz, 1H), 5.46 (dd, *J* = 10.2, 2.1 Hz, 1H), 4.40 (q, *J* = 6.4 Hz, 1H), 3.84 (s, 3H), 2.92–2.60 (m, 2H), 1.36 (d, *J* = 6.4 Hz, 3H); ^13^C NMR (101 MHz, CDCl_3_): δ 192.5, 166.0, 162.8, 129.9, 129.6, 128.6, 123.3, 120.4, 113.1, 110.3, 100.3, 80.4, 55.6, 52.2, 26.9, 16.6; HRMS (ESI-TOF, *m/z*): calcd for C_16_H_16_O_3_ [M + H]^+^, 257.1172; found, 257.1176. HPLC: the ee value was determined by HPLC analysis (Chiralcel IC, i-PrOH/Hexane = 5/95, 1.0 mL/min, 215 nm), retention time: t_major_ = 9.197 min, t_minor_ = 5.367 min, ee = 93%.

Spiro[chromane-3,1'-cyclohexane]-2',5'-dien-4-ol (5aa)**.** The product was obtained as a white solid (isolated yield: 91%). ^1^H NMR (500 MHz, CD_3_CN): δ 7.34 (dd, *J* = 7.7, 1.7 Hz, 1H), 7.19 (ddd, *J* = 8.6, 7.3, 1.7 Hz, 1H), 6.93 (td, *J* = 7.5, 1.2 Hz, 1H), 6.80 (dd, *J* = 8.2, 1.2 Hz, 1H), 5.97 (ddtd, *J* = 22.1, 10.2, 3.4, 1.6 Hz, 2H), 5.65 (dq, *J* = 10.3, 2.1 Hz, 1H), 5.39 (dq, *J* = 10.2, 2.1 Hz, 1H), 4.38 (d, *J* = 4.6 Hz, 1H), 4.02 (d, *J* = 10.6 Hz, 1H), 3.87 (dd, *J* = 10.6, 0.8 Hz, 1H), 3.22 (d, *J* = 6.0 Hz, 1H), 2.72 (ddd, *J* = 5.5, 3.5, 2.1 Hz, 2H); ^13^C NMR (101 MHz, CD_3_CN): δ 154.5, 130.6, 129.9, 128.9, 128.6, 127.8, 125.9, 125.3, 121.6, 116.7, 71.6, 71.2, 40.0, 27.8; HRMS (ESI-TOF, *m/z*): calcd for C_14_H_14_O_2_ [M + H]^+^, 215.1067; found, 215.1069.

4-Methylspiro[chromane-3,1'-cyclohexane]-2',5'-dien-4-ol (5ab)**.** The product was obtained as a white solid (isolated yield: 82%). ^1^H NMR (400 MHz, CD_3_CN): δ 7.49 (dd, *J* = 7.8, 1.7 Hz, 1H), 7.19 (ddd, *J* = 8.2, 7.2, 1.7 Hz, 1H), 6.93 (ddd, *J* = 7.8, 7.2, 1.2 Hz, 1H), 6.80 (dd, *J* = 8.2, 1.3 Hz, 1H), 6.07 (dtd, *J* = 10.4, 3.4, 1.6 Hz, 1H), 5.88 (dtd, *J* = 10.4, 3.4, 1.6 Hz, 1H), 5.71 (dq, *J* = 10.4, 2.2 Hz, 1H), 5.45 (dq, *J* = 10.3, 2.1 Hz, 1H), 4.22 (d, *J* = 10.5 Hz, 1H), 3.81 (d, *J* = 10.4 Hz, 1H), 3.08 (s, 1H), 2.71 (tt, *J* = 3.3, 1.7 Hz, 2H), 1.45 (s, 3H); ^13^C NMR (101 MHz, CD_3_CN): δ 154.1, 129.9, 129.6, 128.8, 128.3, 127.9, 127.5, 126.0, 121.5, 117.2, 71.3, 71.0, 43.2, 27.8, 24.6; HRMS (ESI-TOF, *m/z*): calcd for C_15_H_16_O_2_ [M + H]^+^, 229.1223; found, 229.1225.

Spiro[chromane-3,1'-cyclohexan]-4-ol (5ac)**.** The product was obtained as a white solid (isolated yield: 70%). ^1^H NMR (400 MHz, CDCl_3_): δ 7.27 (dt, *J* = 29.7, 7.7 Hz, 2H), 7.00–6.81 (m, 2H), 4.36 (s, 1H), 4.19–3.81 (m, 2H), 1.77–1.28 (m, 10H); ^13^C NMR (126 MHz, CDCl_3_): δ 153.8, 130.6, 129.6, 123.8, 120.7, 116.5, 70.6, 68.5, 35.7, 30.3, 28.2, 26.3, 21.5, 21.4; HRMS (ESI-TOF, *m/z*): calcd for C_14_H_18_O_2_ [M + Na]^+^, 241.1199; found, 241.1190.

Spiro[chromane-3,1'-cyclohexane] (5ad)**.** The product was obtained as a white solid (isolated yield: 69%). ^1^H NMR (400 MHz, CDCl_3_): δ 7.14–7.01 (m, 2H), 6.89–6.78 (m, 2H), 3.87 (s, 2H), 2.63 (s, 2H), 1.60–1.34 (m, 10H); ^13^C NMR (101 MHz, CDCl_3_): δ 153.1, 129.2, 126.0, 120.3, 119.3, 115.1, 73.2, 35.5, 32.2, 30.2, 25.5, 20.5; HRMS (ESI-TOF, *m/z*): calcd for C_14_H_18_O [M + H]^+^, 203.1430; found, 203.1427.

3',7-Dimethoxyspiro[chromane-3,1'-cyclohexan]-3'-ene-4,5'-dione (5sa)**.** The product was obtained as a white solid (isolated yield: 93%). ^1^H NMR (400 MHz, CDCl_3_): δ 7.81 (d, *J* = 8.9 Hz, 1H), 6.60 (dd, *J* = 8.9, 2.4 Hz, 1H), 6.39 (d, *J* = 2.4 Hz, 1H), 5.43 (d, *J* = 1.5 Hz, 1H), 4.34–4.21 (m, 2H), 3.82 (s, 3H), 3.72 (s, 3H), 2.96 (dd, *J* = 17.9, 1.4 Hz, 1H), 2.79 (d, *J* = 16.8 Hz, 1H), 2.44–2.31 (m, 2H); ^13^C NMR (101 MHz, CDCl_3_): δ 195.7, 191.7, 174.4, 166.4, 162.9, 129.8, 112.8, 110.9, 101.5, 100.6, 72.7, 56.1, 55.7, 45.4, 39.1, 31.5; HRMS (ESI-TOF, *m/z*): calcd for C_16_H_16_O_5_ [M + H]^+^, 289.1071; found, 289.1073.

1-(2-(Benzyloxy)phenyl)-3,3-diphenylpropan-1-one (10)**.** The product was obtained as a white solid (isolated yield: 45%). ^1^H NMR (400 MHz, CDCl_3_): δ 7.56 (dd, *J* = 7.7, 1.8 Hz, 1H), 7.48–7.33 (m, 6H), 7.23–7.08 (m, 10H), 7.04 (dd, *J* = 8.4, 1.0 Hz, 1H), 6.97 (td, *J* = 7.5, 1.0 Hz, 1H), 5.15 (s, 2H), 4.71 (t, *J* = 7.4 Hz, 1H), 3.76 (d, *J* = 7.5 Hz, 2H); ^13^C NMR (101 MHz, CDCl_3_): δ 200.3, 157.8, 144.6, 136.1, 133.5, 130.8, 128.9, 128.8, 128.5, 128.5, 128.2, 128.0, 126.2, 121.2, 112.6, 71.0, 50.3, 46.1; HRMS (ESI-TOF, *m/z*): calcd for C_28_H_24_O_2_ [M + H]^+^, 393.1849; found, 393.1846.

2-(*tert*-Butyl)-4-(2-oxo-2-phenylethyl)-2-azaspiro[4.5]deca-6,9-dien-3-one (9a)**.** The product was obtained as a white solid (isolated yield: 65%). ^1^H NMR (400 MHz, CDCl_3_): δ 7.97–7.88 (m, 2H), 7.55–7.50 (m, 1H), 7.47–7.38 (m, 2H), 5.89 (dtd, *J* = 10.2, 3.3, 1.6 Hz, 1H), 5.77 (dtd, *J* = 10.0, 3.3, 1.6 Hz, 1H), 5.59 (ddq, *J* = 24.2, 10.1, 2.1 Hz, 2H), 3.42–3.13 (m, 4H), 2.88 (dd, *J* = 16.7, 6.3 Hz, 1H), 2.65–2.39 (m, 2H), 1.40 (s, 9H); ^13^C NMR (101 MHz, CDCl_3_): δ 198.8, 174.3, 137.4, 133.0, 129.6, 128.6, 128.3, 127.0, 126.9, 126.9, 56.7, 54.2, 50.0, 41.9, 34.6, 27.9, 26.7; HRMS (ESI-TOF, *m/z*): calcd for C_21_H_25_NO_2_ [M + H]^+^, 324.1958; found, 324.1957.

2-(*tert*-Butyl)-4-(2-oxo-2-(p-tolyl)ethyl)-2-azaspiro[4.5]deca-6,9-dien-3-one (9b)**.** The product was obtained as a white solid (isolated yield: 68%). ^1^H NMR (400 MHz, CDCl_3_): δ 7.84 (d, *J* = 8.2 Hz, 2H), 7.22 (d, *J* = 8.0 Hz, 2H), 5.88 (dtd, *J* = 10.2, 3.3, 1.6 Hz, 1H), 5.76 (dtd, *J* = 10.0, 3.3, 1.6 Hz, 1H), 5.59 (ddq, *J* = 24.3, 10.0, 2.1 Hz, 2H), 3.38–3.19 (m, 4H), 2.93–2.82 (m, 1H), 2.64–2.34 (m, 5H), 1.39 (s, 9H); ^13^C NMR (101 MHz, CDCl_3_): δ 198.2, 174.2, 143.5, 134.7, 129.5, 129.1, 128.3, 126.9, 126.7, 126.7, 56.6, 54.0, 49.9, 41.8, 34.3, 27.8, 26.5, 21.6; HRMS (ESI-TOF, *m/z*): calcd for C_22_H_27_NO_2_ [M + H]^+^, 338.2115; found, 338.2122.

2-(*tert*-Butyl)-4-(2-(4-methoxyphenyl)-2-oxoethyl)-2-azaspiro[4.5]deca-6,9-dien-3-one (9c)**.** The product was obtained as a white solid (isolated yield: 66%). ^1^H NMR (400 MHz, CDCl_3_): δ 7.93 (d, *J* = 8.9 Hz, 2H), 6.89 (d, *J* = 8.9 Hz, 2H), 5.88 (dtd, *J* = 10.3, 3.3, 1.6 Hz, 1H), 5.76 (dtd, *J* = 10.1, 3.3, 1.6 Hz, 1H), 5.58 (ddq, *J* = 25.9, 10.1, 2.1 Hz, 2H), 3.85 (s, 3H), 3.34 (d, *J* = 9.7 Hz, 1H), 3.30–3.13 (m, 3H), 2.84 (dd, *J* = 18.4, 8.1 Hz, 1H), 2.63–2.37 (m, 2H), 1.39 (s, 9H); ^13^C NMR (101 MHz, CDCl_3_): δ 197.1, 174.3, 163.3, 130.5, 130.3, 129.5, 126.9, 126.6, 113.5, 56.6, 55.4, 54.0, 49.9, 41.8, 34.0, 27.8, 26.5; HRMS (ESI-TOF, *m/z*): calcd for C_22_H_27_NO_3_ [M + H]^+^, 354.2064; found, 354.2074.

2-(*tert*-Butyl)-4-(2-(4-fluorophenyl)-2-oxoethyl)-2-azaspiro[4.5]deca-6,9-dien-3-one (9d)**.** The product was obtained as a white solid (isolated yield: 61%). ^1^H NMR (400 MHz, CDCl_3_): δ 8.02–7.93 (m, 2H), 7.14–7.04 (m, 2H), 5.89 (dtd, *J* = 10.2, 3.3, 1.6 Hz, 1H), 5.79 (dtd, *J* = 10.1, 3.3, 1.6 Hz, 1H), 5.59 (ddq, *J* = 25.1, 10.0, 2.1 Hz, 2H), 3.40–3.17 (m, 4H), 2.86–2.74 (m, 1H), 2.66–2.40 (m, 2H), 1.39 (s, 9H); ^13^C NMR (101 MHz, CDCl_3_): δ 197.2, 174.2, δ 165.8 (d, *J_C–F_* = 254.3 Hz), 133.8 (d, *J_C–F_* = 3.0 Hz), 131.0 (d, *J_C–F_* = 9.2 Hz), 129.5, 127.0, 126.9, 126.9, 115.6 (d, *J_C–F_* = 21.8 Hz), 56.7, 54.2, 50.1, 41.9, 34.5, 27.9, 26.7; ^19^F NMR (377 MHz, CDCl_3_): δ –105.87 (tq, *J* = 9.0, 4.5, 3.3 Hz); HRMS (ESI-TOF, *m/z*): calcd for C_21_H_24_FNO_2_ [M + H]^+^, 342.1864; found, 342.1872.

2-(*tert*-Butyl)-4-(2-(4-chlorophenyl)-2-oxoethyl)-2-azaspiro[4.5]deca-6,9-dien-3-one (9e)**.** The product was obtained as a white solid (isolated yield: 46%). ^1^H NMR (400 MHz, CD_3_CN): δ 7.95–7.89 (m, 2H), 7.56–7.48 (m, 2H), 5.92 (dtd, *J* = 10.3, 3.3, 1.6 Hz, 1H), 5.82 (dtd, *J* = 10.1, 3.4, 1.6 Hz, 1H), 5.67 (dq, *J* = 10.3, 2.1 Hz, 1H), 5.57 (dq, *J* = 10.1, 2.1 Hz, 1H), 3.43 (d, *J* = 9.8 Hz, 1H), 3.23 (d, *J* = 9.8 Hz, 1H), 3.18–3.11 (m, 2H), 2.79–2.70 (m, 1H), 2.67–2.40 (m, 2H), 1.37 (s, 9H); ^13^C NMR (126 MHz, CD_3_CN): δ 198.8, 174.2, 139.2, 136.8, 130.4, 130.0, 129.4, 127.5, 127.5, 127.0, 56.9, 54.1, 51.2, 42.1, 34.9, 27.5, 27.5, 26.9; HRMS (ESI-TOF, *m/z*): calcd for C_21_H_24_ClNO_2_ [M + H]^+^, 358.1569; found, 358.1575.

2-(*tert*-Butyl)-4-(2-(2,4-dimethoxyphenyl)-2-oxoethyl)-2-azaspiro[4.5]deca-6,9-dien-3-one (9f)**.** The product was obtained as a white solid (isolated yield: 65%). ^1^H NMR (400 MHz, CD_3_CN): δ 7.57 (dt, *J* = 8.5, 1.0 Hz, 1H), 6.58–6.52 (m, 2H), 5.83 (ddtd, *J* = 17.0, 11.6, 3.3, 1.6 Hz, 2H), 5.56 (ddq, *J* = 28.5, 10.0, 2.1 Hz, 2H), 3.85 (s, 3H), 3.84 (s, 3H), 3.36 (d, *J* = 9.7 Hz, 1H), 3.17 (d, *J* = 9.7 Hz, 1H), 3.09–2.96 (m, 2H), 2.78 (dd, *J* = 16.4, 5.3 Hz, 1H), 2.65–2.42 (m, 2H), 1.33 (s, 9H); ^13^C NMR (101 MHz, CD_3_CN): δ 199.3, 174.5, 164.8, 161.1, 132.7, 130.2, 127.8, 127.1, 126.3, 122.0, 106.2, 98.8, 56.7, 56.0, 55.9, 53.9, 50.8, 42.0, 40.1, 27.4, 26.8; HRMS (ESI-TOF, *m/z*): calcd for C_23_H_29_NO_4_ [M + H]^+^, 384.2170; found, 384.2185.

2-(*tert*-Butyl)-4-(2-(3,4-dimethylphenyl)-2-oxoethyl)-2-azaspiro[4.5]deca-6,9-dien-3-one (9g)**.** The product was obtained as a white solid (isolated yield: 68%). ^1^H NMR (400 MHz, CD_3_CN): δ 7.72–7.61 (m, 2H), 7.23 (d, *J* = 7.8 Hz, 1H), 5.89 (dtd, *J* = 10.2, 3.3, 1.6 Hz, 1H), 5.78 (dtd, *J* = 10.1, 3.4, 1.7 Hz, 1H), 5.65 (dq, *J* = 10.3, 2.1 Hz, 1H), 5.54 (dq, *J* = 10.1, 2.1 Hz, 1H), 3.40 (d, *J* = 9.8 Hz, 1H), 3.20 (d, *J* = 9.8 Hz, 1H), 3.16–3.05 (m, 2H), 2.79–2.68 (m, 1H), 2.64–2.39 (m, 2H), 2.31 (s, 6H), 1.34 (s, 9H); ^13^C NMR (101 MHz, CD_3_CN): δ 199.7, 174.7, 143.5, 138.0, 136.4, 130.6, 130.4, 130.0, 128.0, 127.7, 127.2, 126.7, 57.2, 54.4, 51.4, 42.4, 35.0, 27.8, 27.2, 20.0, 19.8; HRMS (ESI-TOF, *m/z*): calcd for C_23_H_29_NO_2_ [M + H]^+^, 352.2271; found, 352.2281.

2-(*tert*-Butyl)-4-(2-(furan-2-yl)-2-oxoethyl)-2-azaspiro[4.5]deca-6,9-dien-3-one (9h)**.** The product was obtained as a white solid (isolated yield: 35%). ^1^H NMR (400 MHz, CD_3_CN): δ 7.69 (d, *J* = 1.4 Hz, 1H), 7.23 (dd, *J* = 3.6, 0.8 Hz, 1H), 6.59 (dd, *J* = 3.6, 1.7 Hz, 1H), 5.90 (dtd, *J* = 10.3, 3.3, 1.6 Hz, 1H), 5.80 (dtd, *J* = 10.1, 3.4, 1.6 Hz, 1H), 5.64 (dq, *J* = 10.2, 2.1 Hz, 1H), 5.53 (dq, *J* = 10.1, 2.1 Hz, 1H), 3.40 (d, *J* = 9.8 Hz, 1H), 3.20 (d, *J* = 9.8 Hz, 1H), 3.07 (t, *J* = 6.5 Hz, 1H), 2.97 (dd, *J* = 16.3, 7.0 Hz, 1H), 2.64–2.40 (m, 3H), 1.34 (s, 9H); ^13^C NMR (101 MHz, CD_3_CN): δ 188.5, 174.4, 153.8, 148.0, 130.2, 127.9, 127.8, 127.3, 118.3, 113.2, 57.2, 54.4, 51.3, 42.3, 35.1, 27.8, 27.2; HRMS (ESI-TOF, *m/z*): calcd for C_19_H_23_NO_3_ [M + H]^+^, 314.1760; found, 314.1755.

2-(*tert*-Butyl)-4-(2-(4-methoxyphenyl)-2-oxoethyl)-6,10-dimethyl-2-azaspiro[4.5]deca-6,9-dien-3-one (9i)**.** The product was obtained as a white solid (isolated yield: 27%). ^1^H NMR (400 MHz, CD_3_CN): δ 7.99–7.85 (m, 2H), 7.05–6.95 (m, 2H), 5.64–5.59 (m, 1H), 5.53 (tt, *J* = 3.2, 1.5 Hz, 1H), 3.88 (s, 3H), 3.76 (d, *J* = 10.6 Hz, 1H), 3.56 (dd, *J* = 6.7, 5.2 Hz, 1H), 3.28 (d, *J* = 10.6 Hz, 1H), 3.15 (dd, *J* = 16.9, 5.2 Hz, 1H), 2.78 (dd, *J* = 16.9, 6.7 Hz, 1H), 2.55–2.44 (m, 1H), 2.37–2.26 (m, 1H), 1.83 (q, *J* = 1.7 Hz, 3H), 1.74 (q, *J* = 1.7 Hz, 3H), 1.42 (s, 9H); ^13^C NMR (101 MHz, CD_3_CN): δ 197.9, 174.9, 164.0, 134.5, 132.9, 130.9, 130.8, 124.9, 124.6, 114.2, 55.9, 54.4, 52.2, 46.2, 45.6, 34.5, 27.5, 27.4, 21.2, 19.4; HRMS (ESI-TOF, *m/z*): calcd for C_24_H_31_NO_3_ [M + Na]^+^, 404.2196; found, 404.2190.

2-(*tert*-Butyl)-7,9-dimethoxy-4-(2-(4-methoxyphenyl)-2-oxoethyl)-2-azaspiro[4.5]deca-6,9-dien-3-one (9j)**.** The product was obtained as a white solid (isolated yield: 81%). ^1^H NMR (500 MHz, CD_3_CN): δ 7.88 (d, *J* = 9.0 Hz, 2H), 6.97 (d, *J* = 9.0 Hz, 2H), 4.64 (d, *J* = 1.4 Hz, 1H), 4.49 (d, *J* = 1.4 Hz, 1H), 3.85 (s, 3H), 3.58 (s, 3H), 3.45 (s, 3H), 3.21 – 3.11 (m, 2H), 3.02 (dd, *J* = 16.3, 6.1 Hz, 1H), 2.71 (dd, *J* = 16.3, 6.8 Hz, 1H), 2.62–2.40 (m, 2H), 1.36 (s, 9H); ^13^C NMR (126 MHz, CD_3_CN): δ 197.8, 173.9, 163.2, 153.3, 153.1, 130.4, 130.1, 113.5, 97.3, 94.2, 57.4, 55.1, 53.8, 53.2, 51.9, 44.2, 33.7, 30.8, 26.8; HRMS (ESI-TOF, *m/z*): calcd for C_24_H_31_NO_5_ [M + H]^+^, 414.2275; found, 414.2287.

4-(2-(6-(3-((3r,5r,7r)-Adamantan-1-yl)-4-methoxyphenyl)naphthalen-2-yl)-2-oxoethyl)-2-(*tert*-butyl)-2-azaspiro[4.5]deca-6,9-dien-3-one (9k)**.** The product was obtained as a white solid (isolated yield: 48%). ^1^H NMR (400 MHz, CD_3_CN): δ 8.54 (d, *J* = 1.4 Hz, 1H), 8.16–8.05 (m, 2H), 7.97 (d, *J* = 1.2 Hz, 2H), 7.87 (dd, *J* = 8.6, 1.9 Hz, 1H), 7.67–7.58 (m, 2H), 7.09 (d, *J* = 8.2 Hz, 1H), 5.92 (dtd, *J* = 9.9, 3.4, 1.6 Hz, 1H), 5.78 (dtd, *J* = 10.0, 3.3, 1.6 Hz, 1H), 5.71 (dq, *J* = 10.3, 2.1 Hz, 1H), 5.57 (dq, *J* = 10.1, 2.1 Hz, 1H), 3.89 (s, 3H), 3.43 (d, *J* = 9.7 Hz, 1H), 3.33–3.17 (m, 3H), 2.93 (dd, *J* = 16.3, 5.9 Hz, 1H), 2.62–2.34 (m, 2H), 2.20 (m, 6H), 2.08 (m, 3H), 1.82 (m, 6H), 1.36 (s, 9H); ^13^C NMR (101 MHz, CD_3_CN): δ 199.4, 174.3, 159.7, 141.7, 139.4, 136.5, 135.0, 132.7, 131.9, 130.6, 130.1, 130.0, 128.9, 127.5, 127.4, 126.9, 126.8, 126.4, 126.2, 125.0, 124.8, 113.2, 56.8, 55.5, 54.0, 51.2, 42.1, 40.9, 37.6, 37.3, 34.8, 29.7, 27.4, 26.8; HRMS (ESI-TOF, *m/z*): calcd for C_42_H_47_NO_3_ [M + H]^+^, 614.3629; found, 614.3658.

# 4. Supplementary Note 2

## 4.1 NMR spectra and HPLC chromatograms of substrates and products

^1^H NMR spectra of 2-(Benzyloxy)-6-methoxybenzoic acid (1h)

^1^H NMR (400 MHz, CDCl_3_)


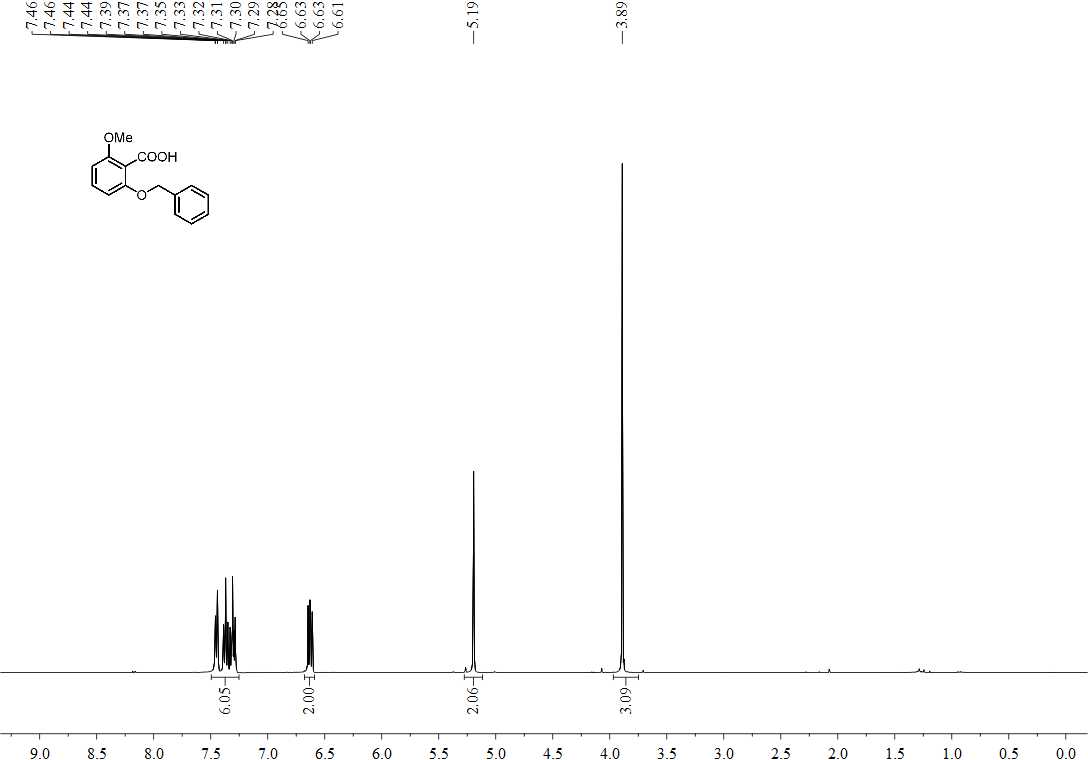


**Supplementary Figure 7.** ^1^H NMR spectra of 2-(benzyloxy)-6-methoxybenzoic acid (**1h**).

^13^C NMR (101 MHz, CDCl_3_)

**、**
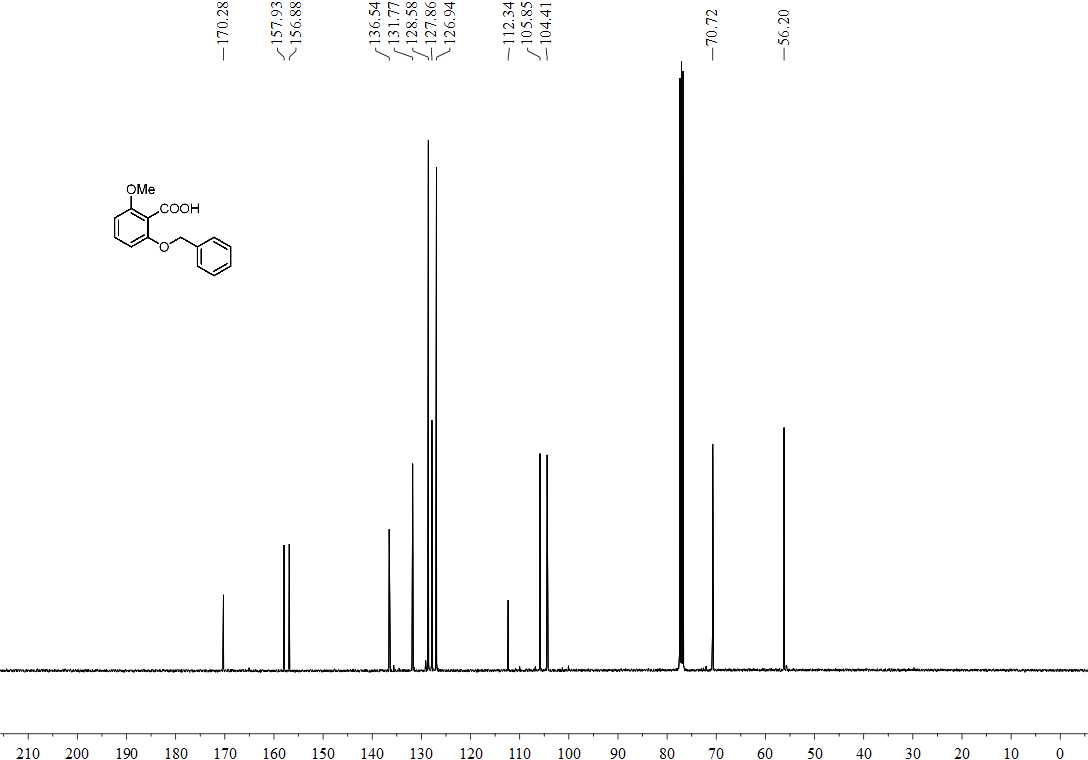


**Supplementary Figure 8.** ^13^C NMR NMR spectra of 2-(benzyloxy)-6-methoxybenzoic acid (**1h**).

2-(Benzyloxy)-5-bromo-4-methylbenzoic acid (1k)

^1^H NMR (400 MHz, CDCl_3_)


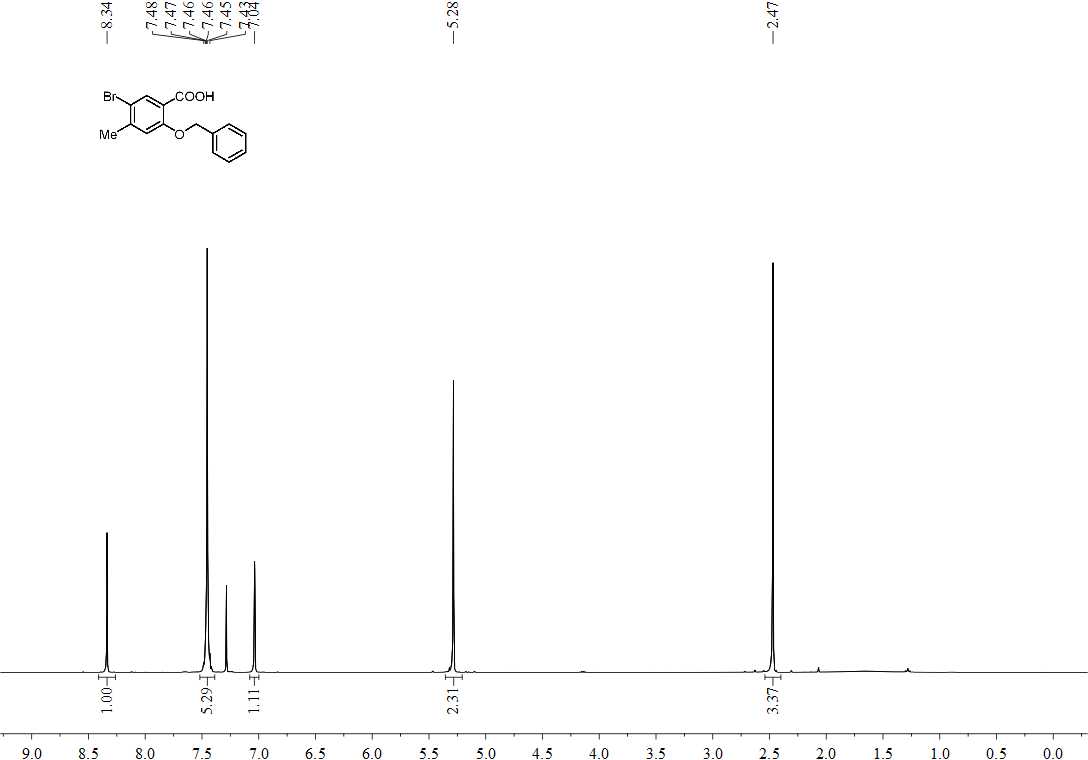


**Supplementary Figure 9.** ^1^H NMR spectra of 2-(benzyloxy)-5-bromo-4-methylbenzoic acid (**1k**)

^13^C NMR (101 MHz, CDCl_3_)


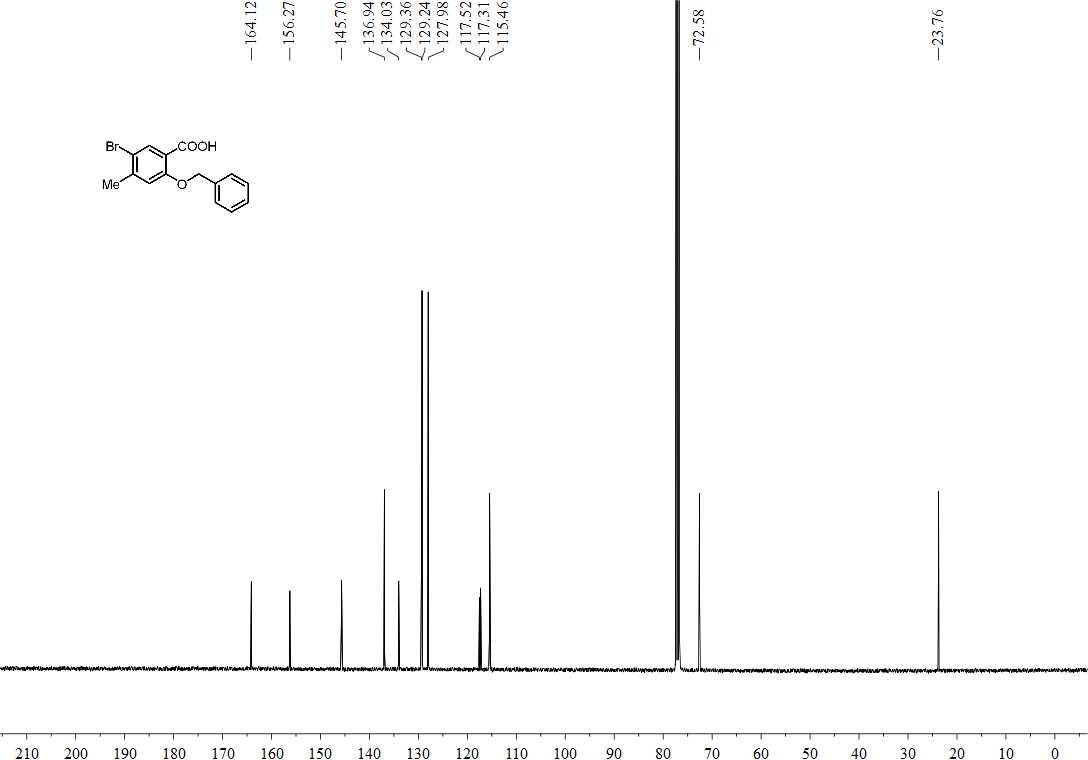


**Supplementary Figure 10.** ^13^C NMR spectra of 2-(benzyloxy)-5-bromo-4-methylbenzoic acid (**1k**).

2-((2-Fluorobenzyl)oxy)-4-methoxybenzoic acid (1l)

^1^H NMR (400 MHz, CDCl_3_)


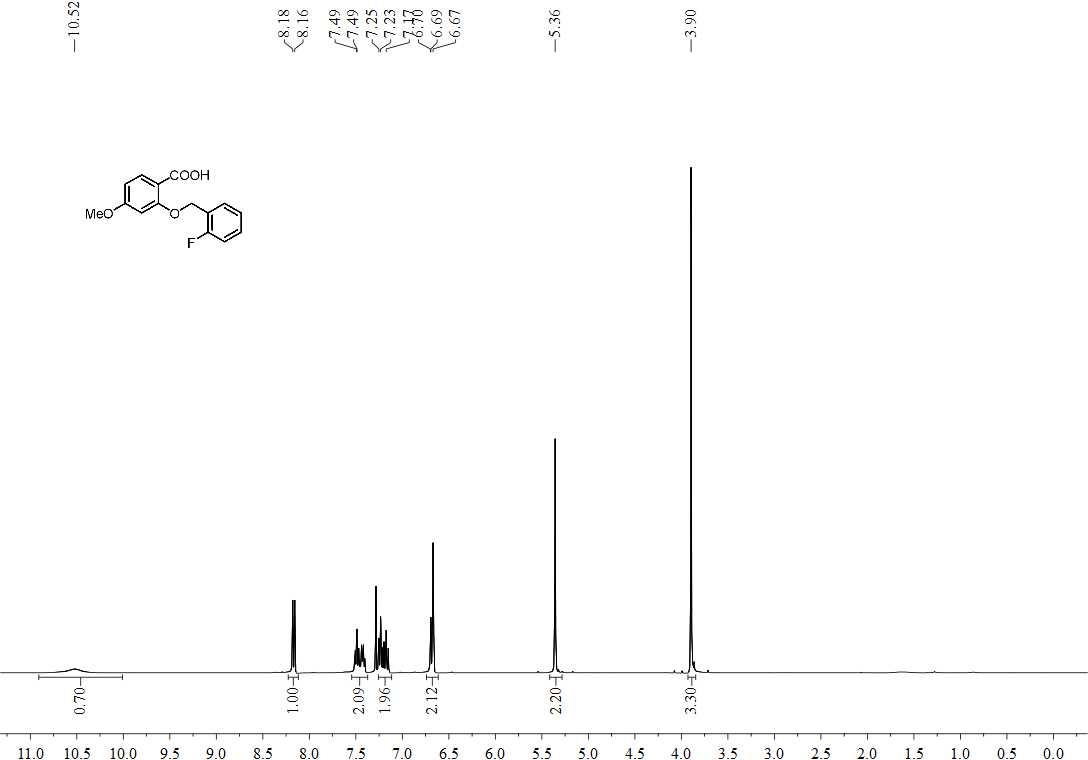


**Supplementary Figure 11.** ^1^H NMR spectra of 2-((2-fluorobenzyl)oxy)-4-methoxybenzoic acid (**1l**).

^13^C NMR (101 MHz, CDCl_3_)


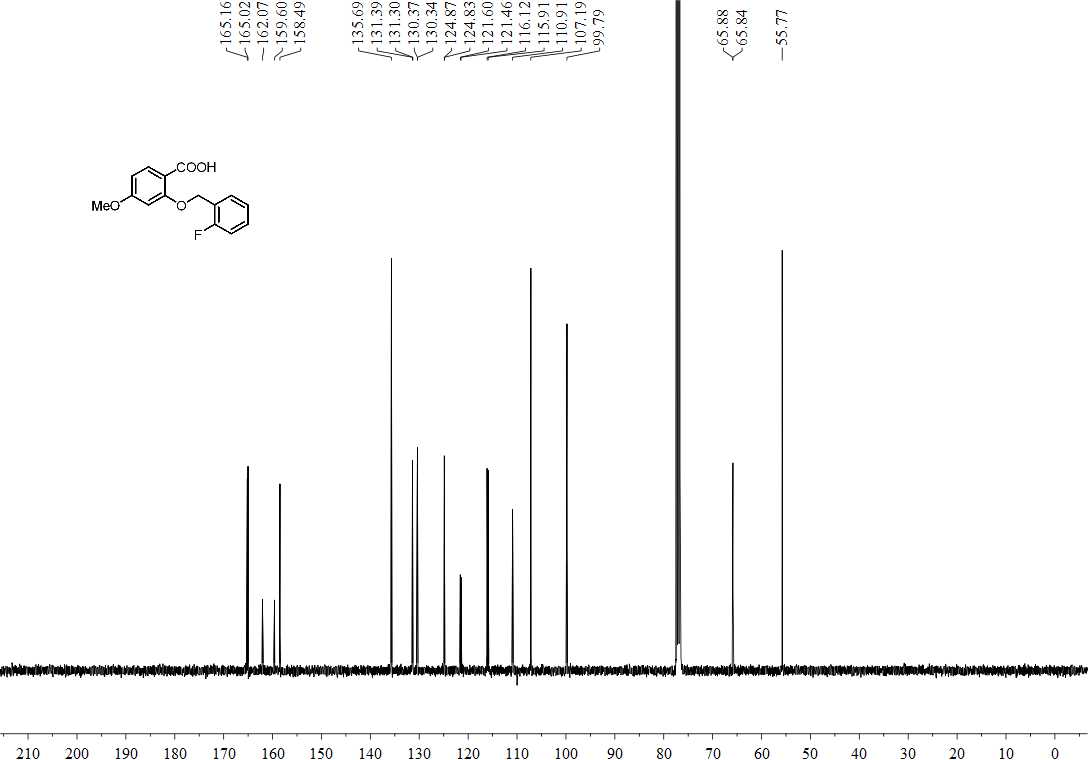


**Supplementary Figure 12.** ^13^C NMR spectra of 2-((2-fluorobenzyl)oxy)-4-methoxybenzoic acid (**1l**).

^19^F NMR (377 MHz, CDCl_3_)


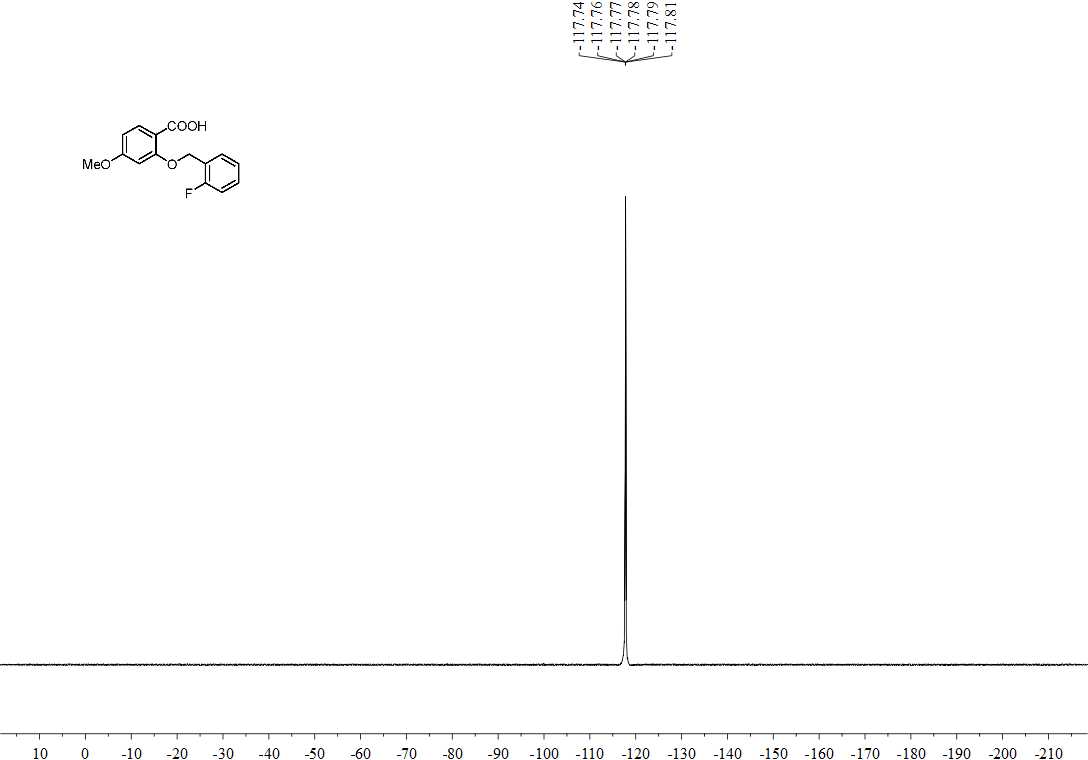


**Supplementary Figure 13.** ^19^F NMR spectra of 2-((2-fluorobenzyl)oxy)-4-methoxybenzoic acid (**1l**).

2-((3-Fluorobenzyl)oxy)-4-methoxybenzoic acid (1m)

^1^H NMR (400 MHz, CDCl_3_)


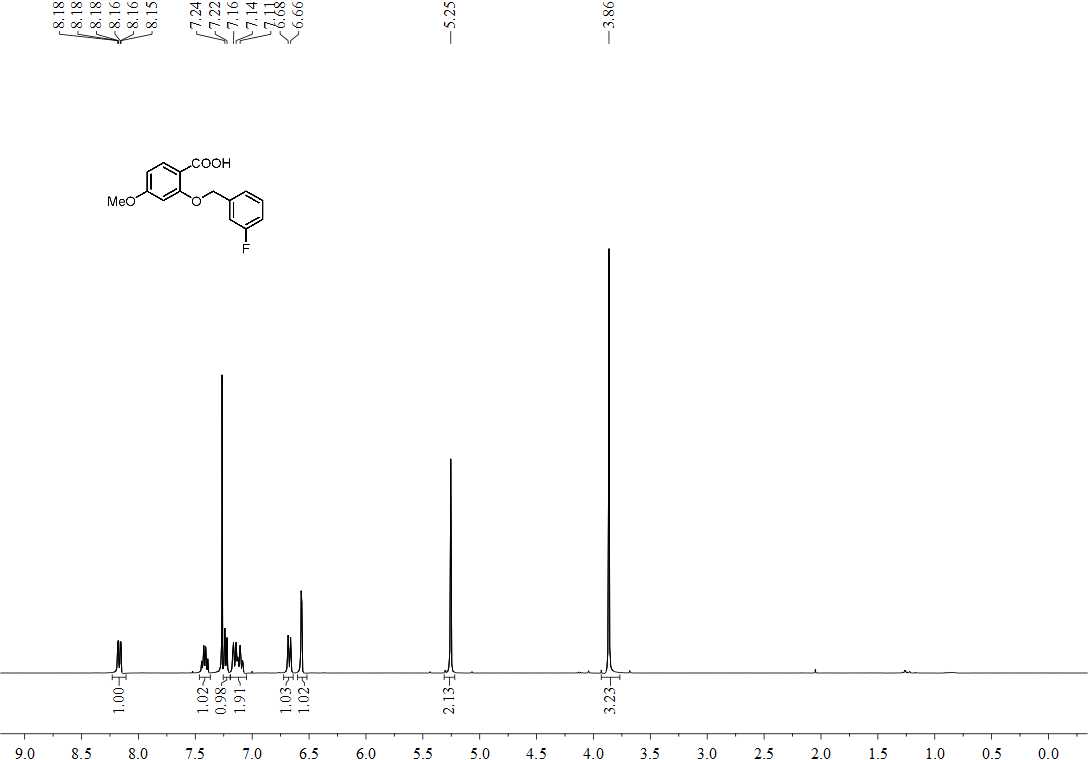


**Supplementary Figure 14.** ^1^H NMR spectra of 2-((3-fluorobenzyl)oxy)-4-methoxybenzoic acid (**1m**).

^13^C NMR (101 MHz, CDCl_3_)


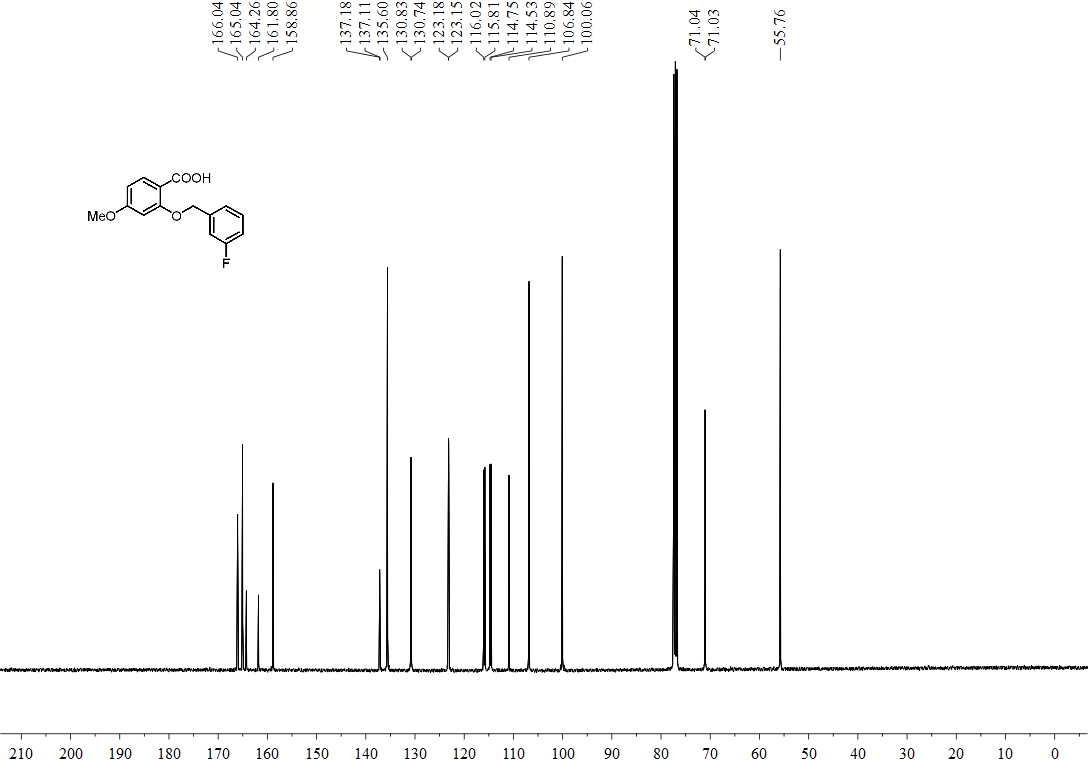


**Supplementary Figure 15.** ^13^C NMR spectra of 2-((3-Fluorobenzyl)oxy)-4-methoxybenzoic acid (**1m**).

^19^F NMR (377 MHz, CDCl_3_)


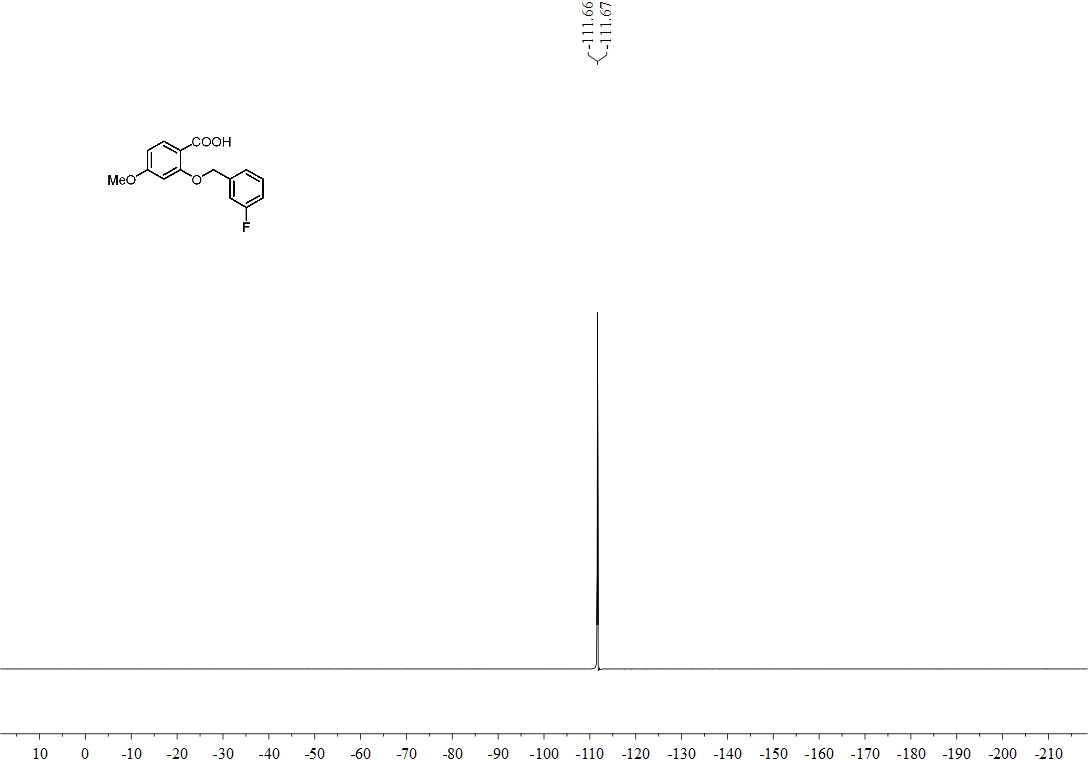


**Supplementary Figure 16.** ^19^F NMR spectra of 2-((3-Fluorobenzyl)oxy)-4-methoxybenzoic acid (**1m**).

2-((3-Chlorobenzyl)oxy)-4-methoxybenzoic acid (1n)

^1^H NMR (400 MHz, CDCl_3_)


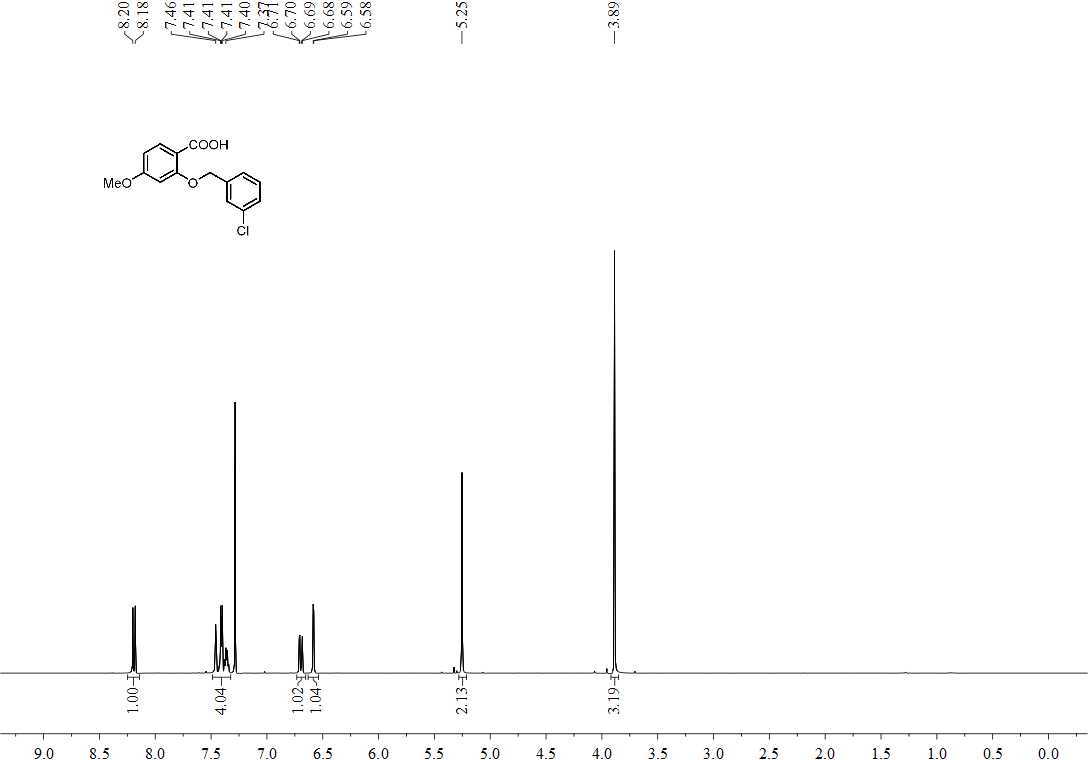


**Supplementary Figure 17.** ^1^H NMR spectra of 2-((3-chlorobenzyl)oxy)-4-methoxybenzoic acid (**1n**).

^13^C NMR (101 MHz, CDCl_3_)


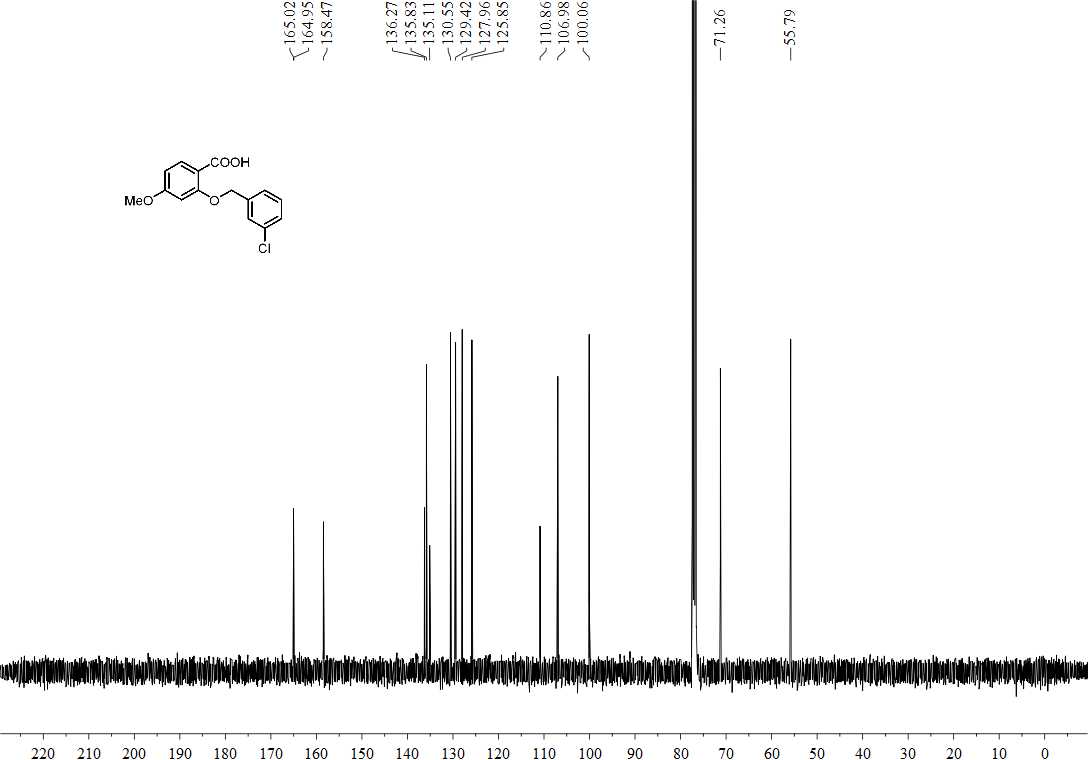


**Supplementary Figure 18.** ^13^C NMR spectra of 2-((3-chlorobenzyl)oxy)-4-methoxybenzoic acid (**1n**).

4-Methoxy-2-((3-(trifluoromethyl)benzyl)oxy)benzoic acid (1o)

^1^H NMR (400 MHz, CDCl_3_)


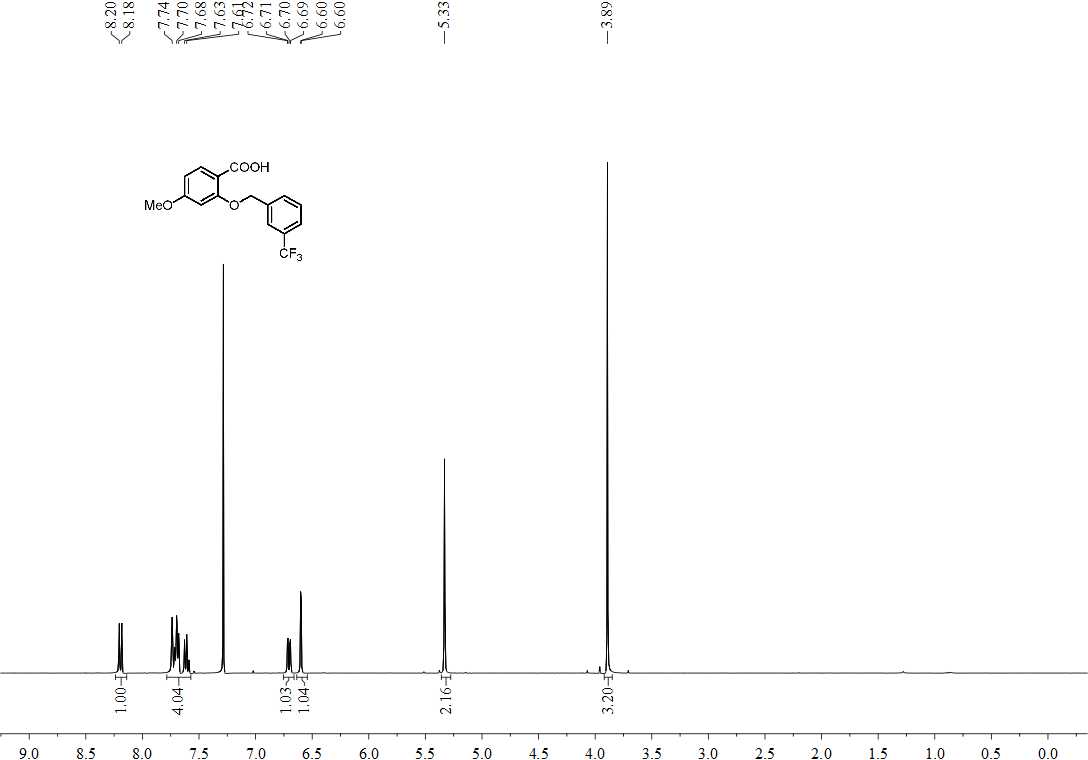


**Supplementary Figure 19.** ^1^H NMR spectra of 4-methoxy-2-((3-(trifluoromethyl)benzyl)oxy)benzoic acid (**1o**).

^13^C NMR (101 MHz, CDCl_3_)


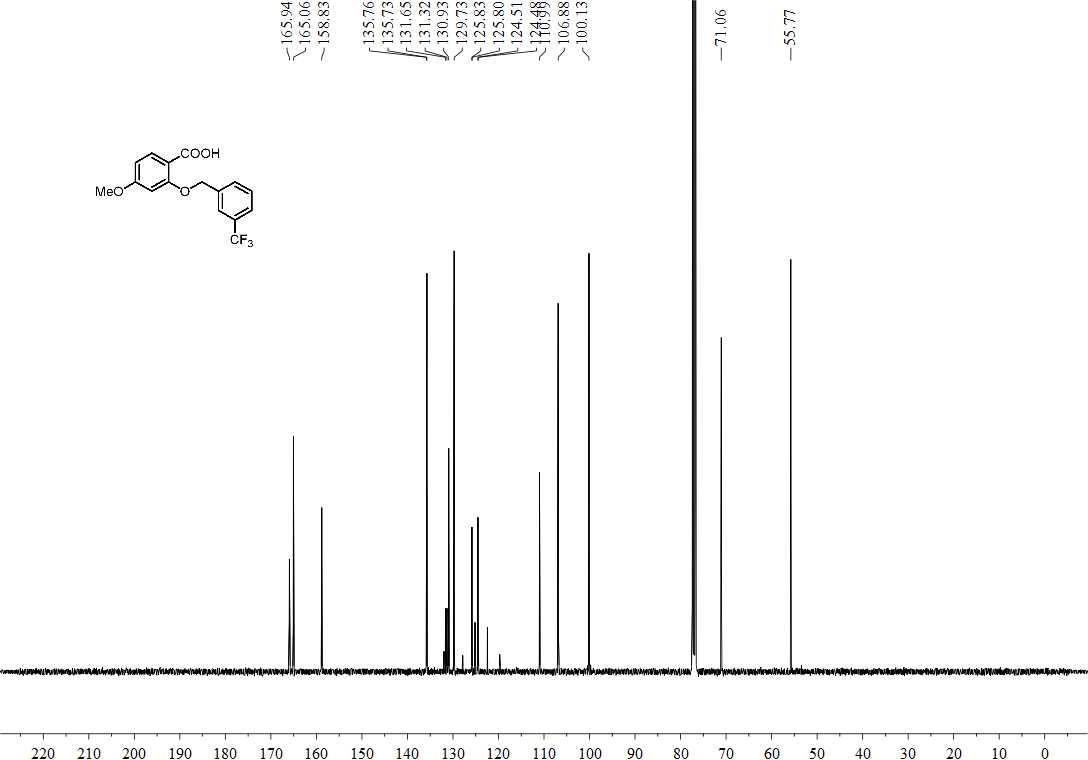


**Supplementary Figure 20.** ^13^C NMR spectra of 4-methoxy-2-((3-(trifluoromethyl)benzyl)oxy)benzoic acid (**1o**).

^19^F NMR (377 MHz, CDCl_3_)


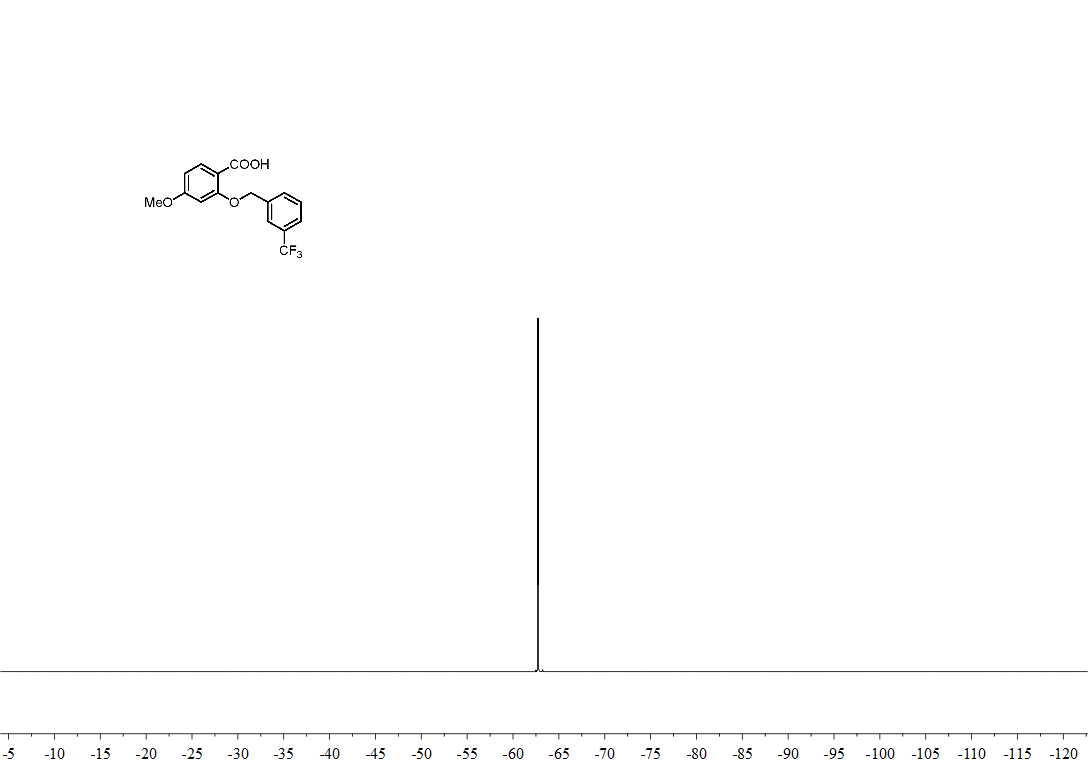


**Supplementary Figure 21.** ^19^F NMR spectra of 4-methoxy-2-((3-(trifluoromethyl)benzyl)oxy)benzoic acid (1o).

4-Methoxy-2-((3-methylbenzyl)oxy)benzoic acid (1p)

^1^H NMR (400 MHz, CDCl_3_)


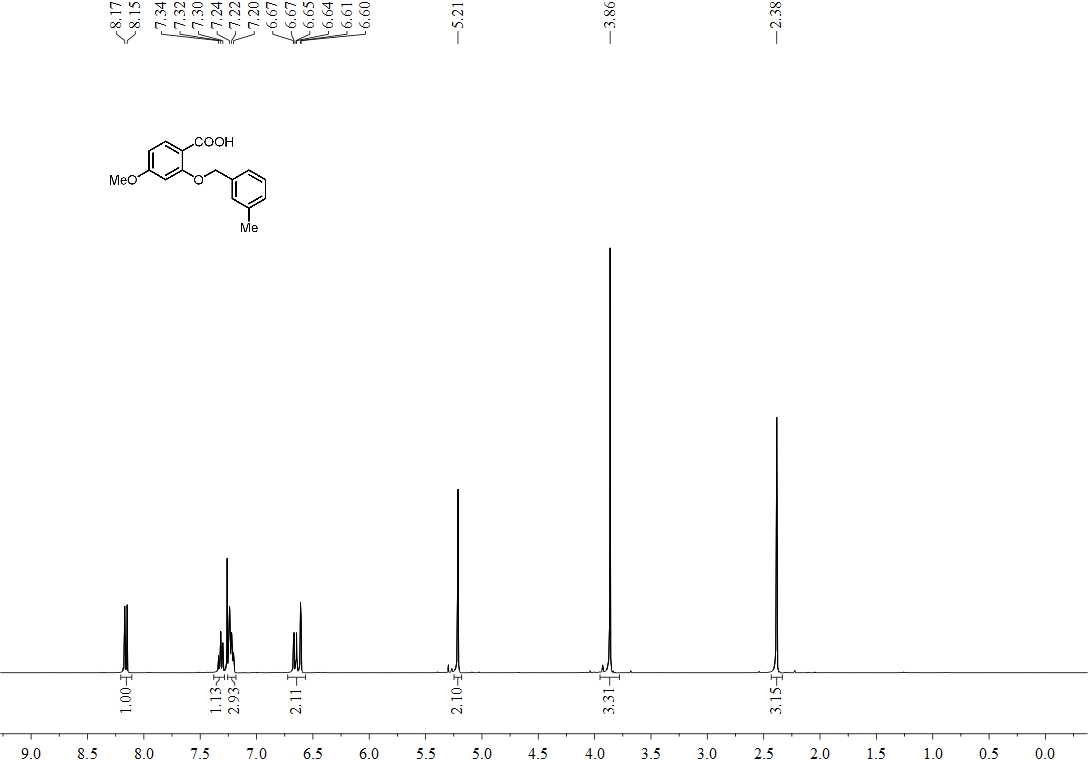


**Supplementary Figure 22.** ^1^H NMR spectra of 4-methoxy-2-((3-methylbenzyl)oxy)benzoic acid (**1p**).

^13^C NMR (101 MHz, CDCl_3_)


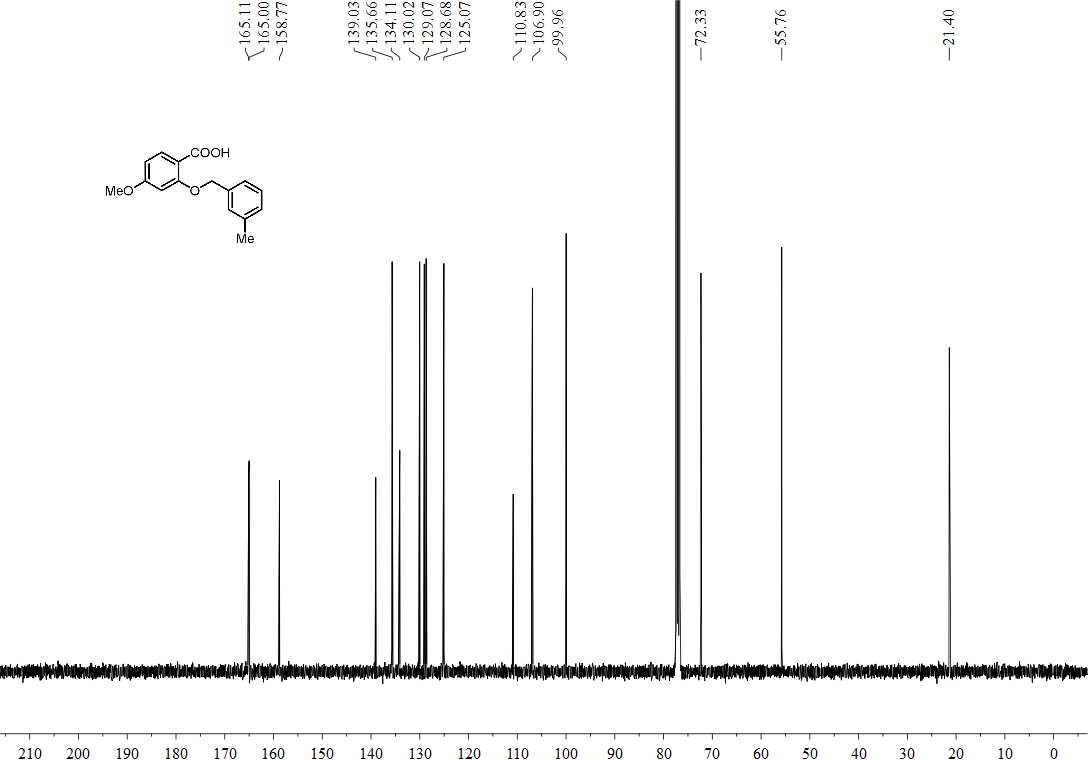


**Supplementary Figure 23.** ^13^C NMR spectra of 4-methoxy-2-((3-methylbenzyl)oxy)benzoic acid (**1p**).

4-Methoxy-2-((3-methoxybenzyl)oxy)benzoic acid (1q)

^1^H NMR (400 MHz, CDCl_3_)


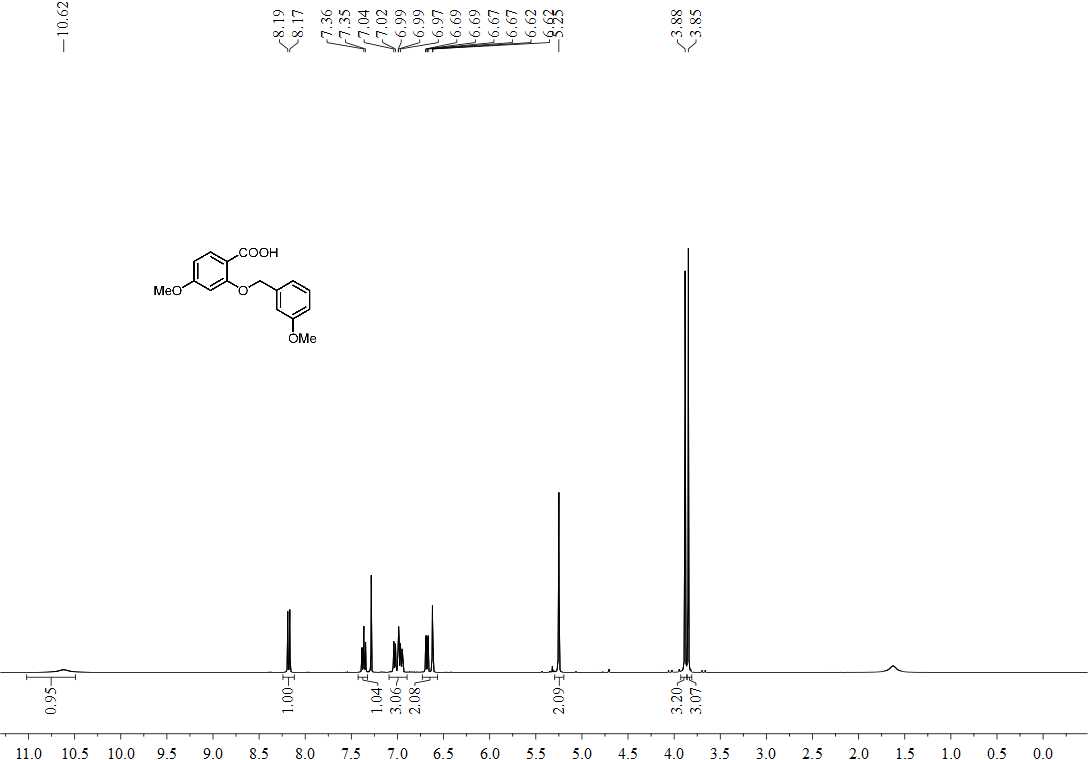


**Supplementary Figure 24.** ^1^H NMR spectra of 4-methoxy-2-((3-methoxybenzyl)oxy)benzoic acid (**1q**).

^13^C NMR (101 MHz, CDCl_3_)


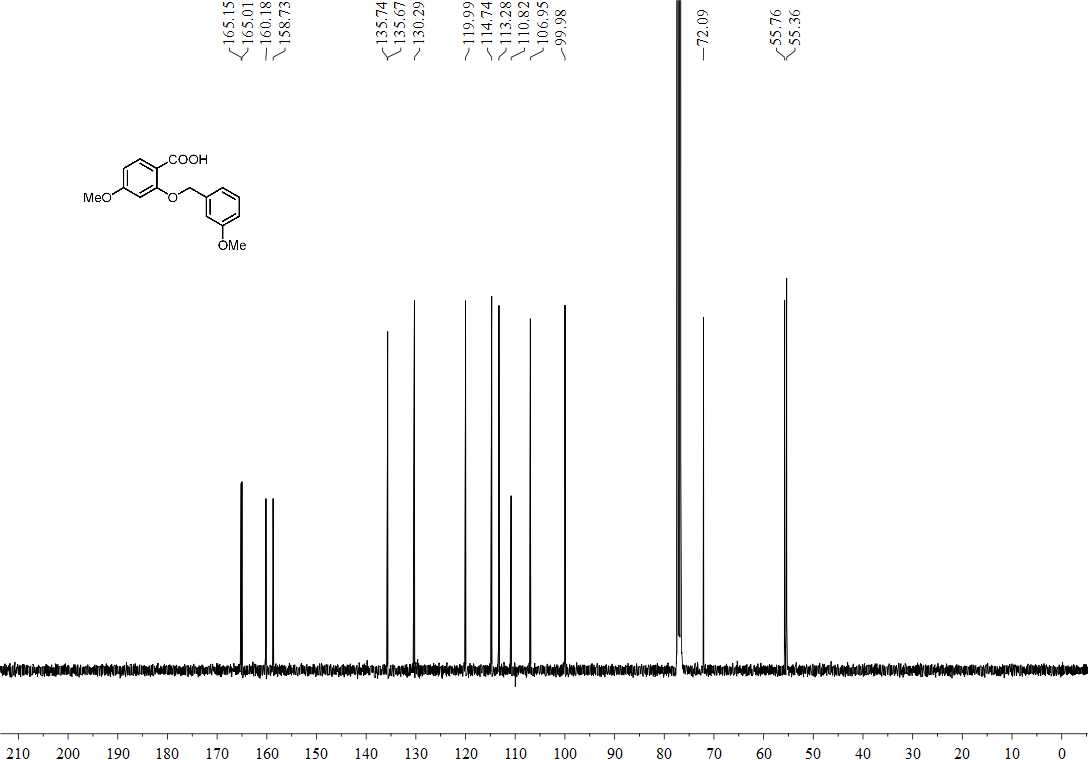


**Supplementary Figure 25.** ^13^C NMR spectra of 4-methoxy-2-((3-methoxybenzyl)oxy)benzoic acid (**1q**).

2-((3,5-Dimethylbenzyl)oxy)-4-methoxybenzoic acid (1r)

^1^H NMR (400 MHz, CDCl_3_)


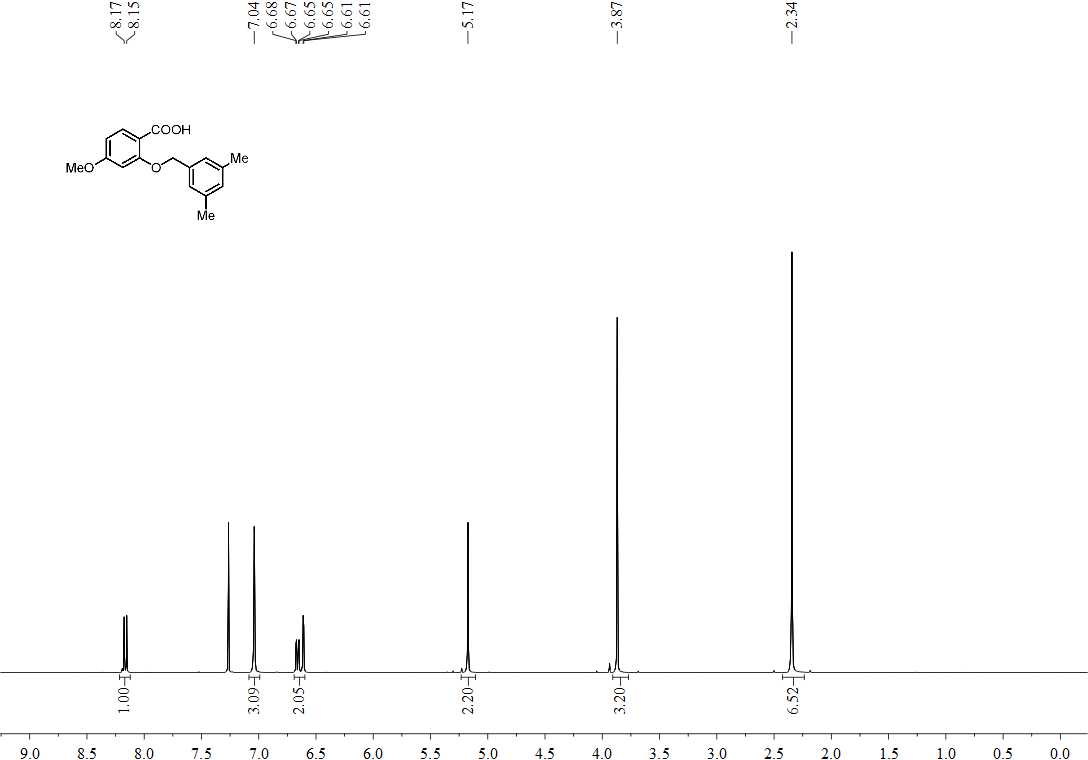


**Supplementary Figure 26.** ^1^H NMR spectra of 2-((3,5-dimethylbenzyl)oxy)-4-methoxybenzoic acid (**1r**).

^13^C NMR (101 MHz, CDCl_3_)


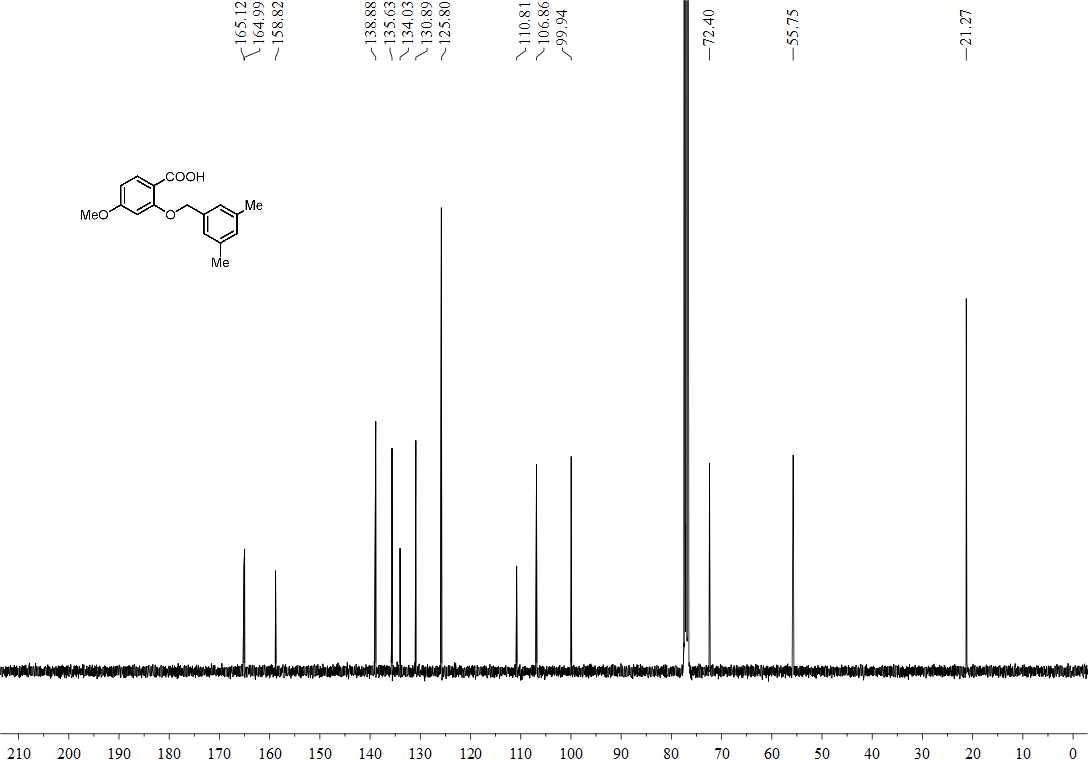


**Supplementary Figure 27.** ^13^C NMR spectra of 2-((3,5-dimethylbenzyl)oxy)-4-methoxybenzoic acid (**1r**).

2-((3,5-Dimethoxybenzyl)oxy)-4-methoxybenzoic acid (1s)

^1^H NMR (400 MHz, CDCl_3_)


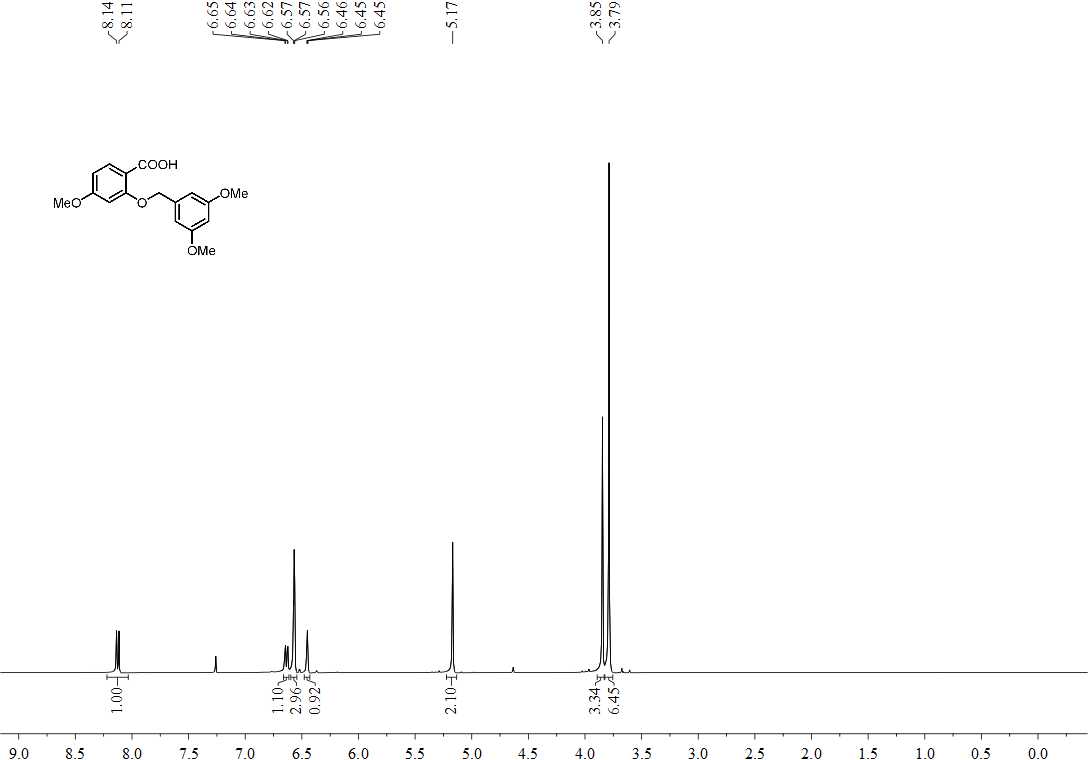


**Supplementary Figure 28.** ^1^H NMR spectra of 2-((3,5-dimethoxybenzyl)oxy)-4-methoxybenzoic acid (**1s**).

^13^C NMR (101 MHz, CDCl_3_)


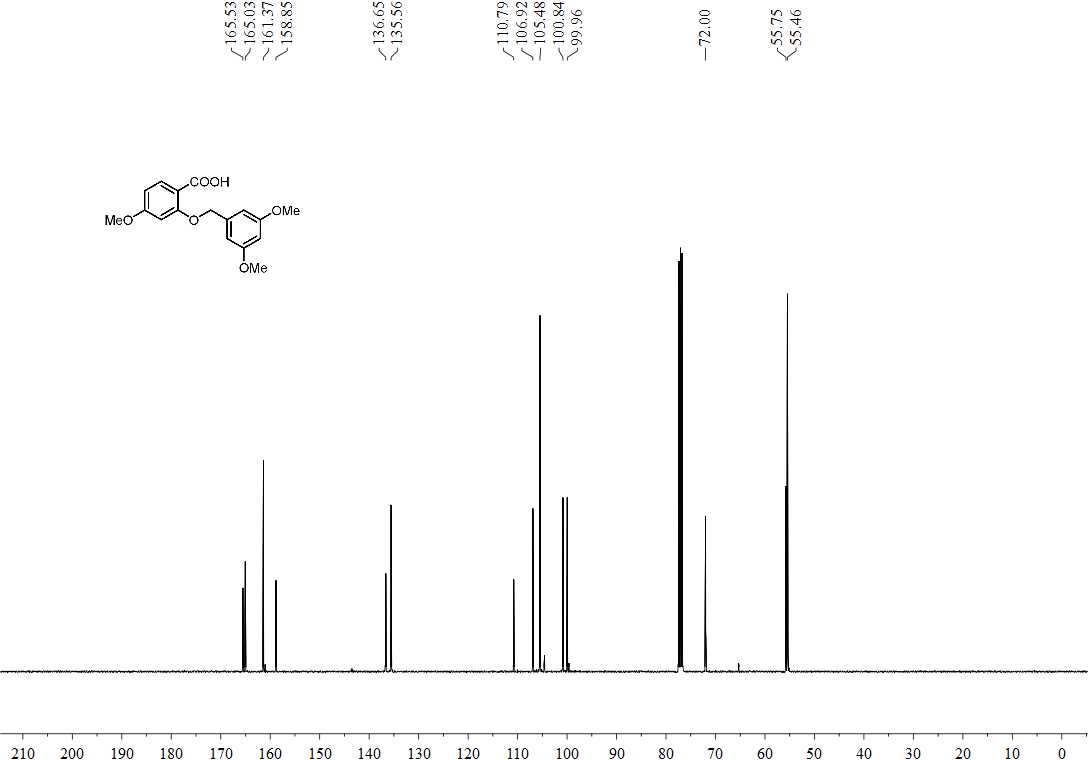


**Supplementary Figure 29.** ^13^C NMR spectra of 2-((3,5-dimethoxybenzyl)oxy)-4-methoxybenzoic acid (**1s**).

4-Methoxy-2-(naphthalen-1-ylmethoxy)benzoic acid (1t)

^1^H NMR (400 MHz, CDCl_3_)


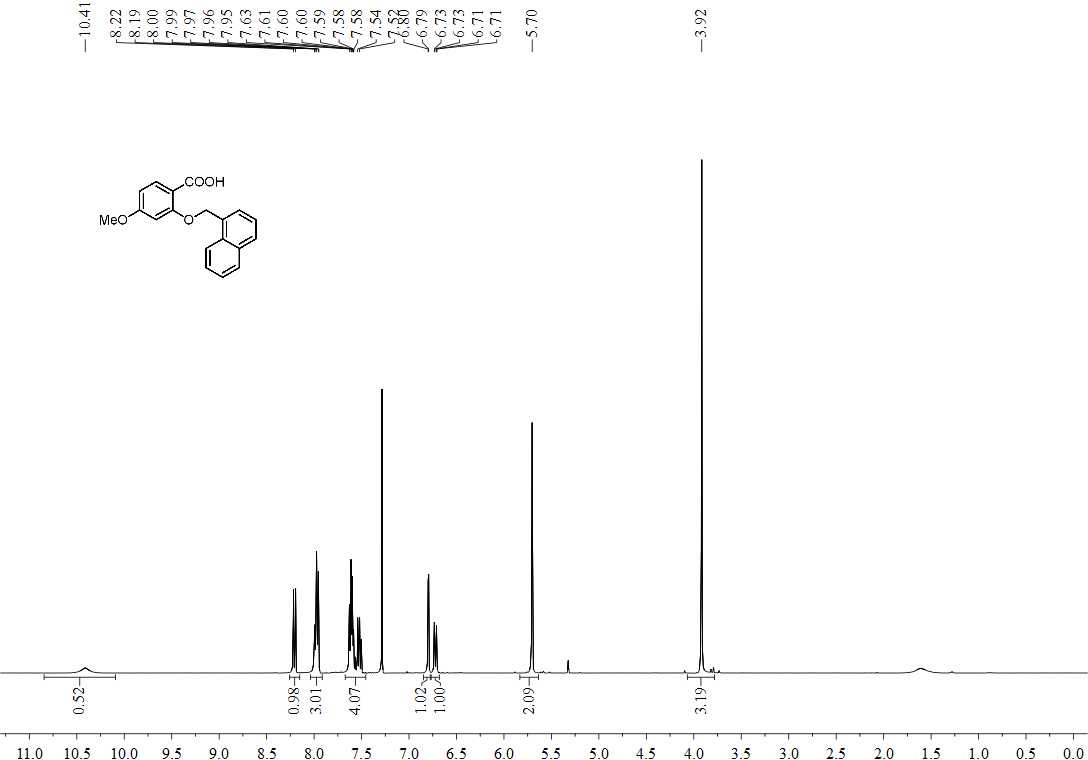


**Supplementary Figure 30.** ^1^H NMR spectra of 4-methoxy-2-(naphthalen-1-ylmethoxy)benzoic acid (**1t**).

^13^C NMR (101 MHz, CDCl_3_)


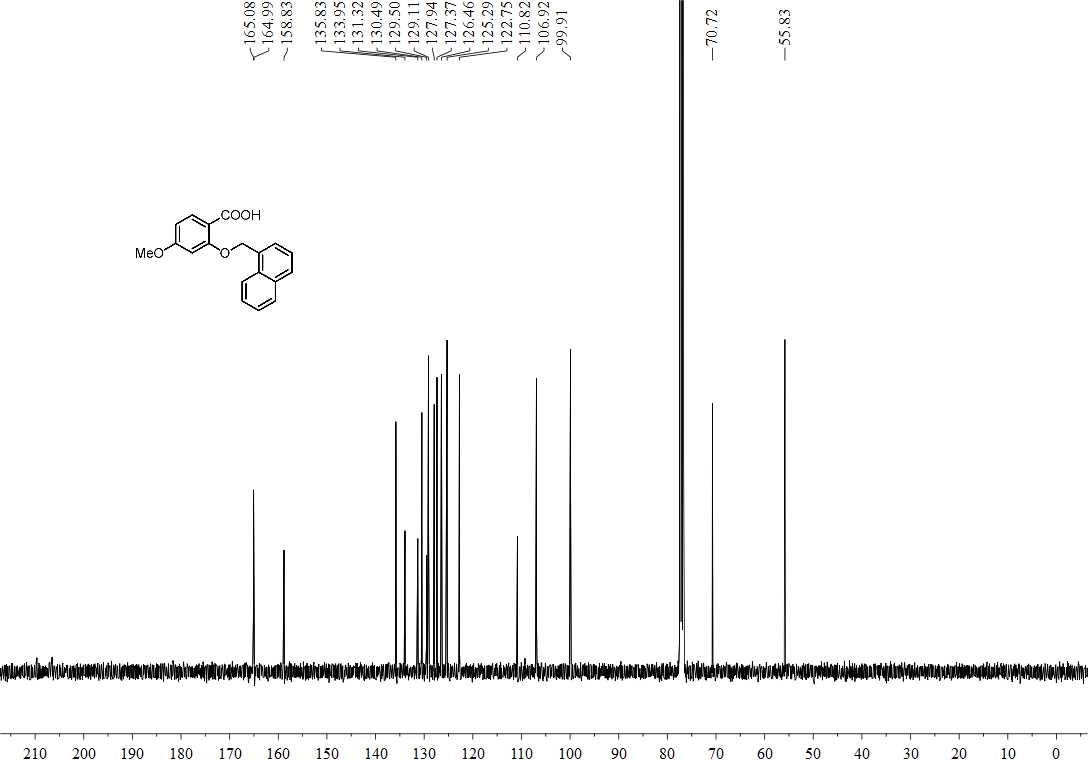


**Supplementary Figure 31.** ^13^C NMR spectra of 4-methoxy-2-(naphthalen-1-ylmethoxy)benzoic acid (**1t**).

2-(Benzyl(tert-butoxycarbonyl)amino)benzoic acid (1u)

^1^H NMR (400 MHz, CDCl_3_)


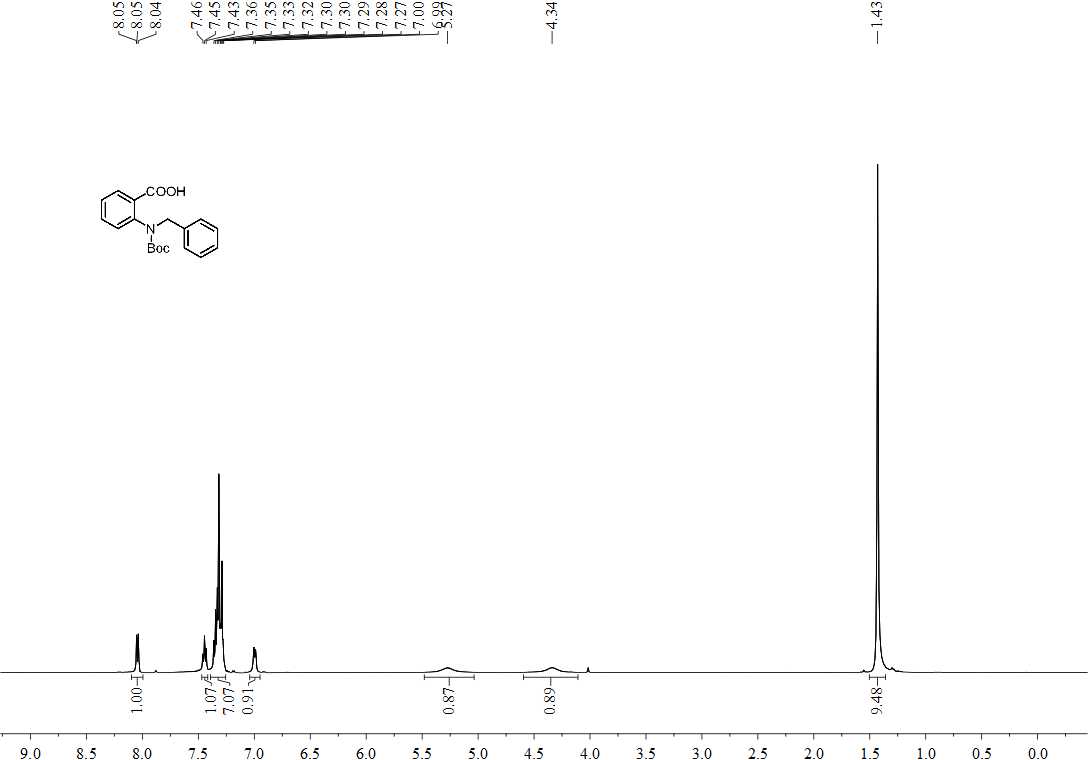


**Supplementary Figure 32.** ^1^H NMR spectra of 2-(benzyl(*tert*-butoxycarbonyl)amino)benzoic acid (1u).

^13^C NMR (101 MHz, CDCl_3_)


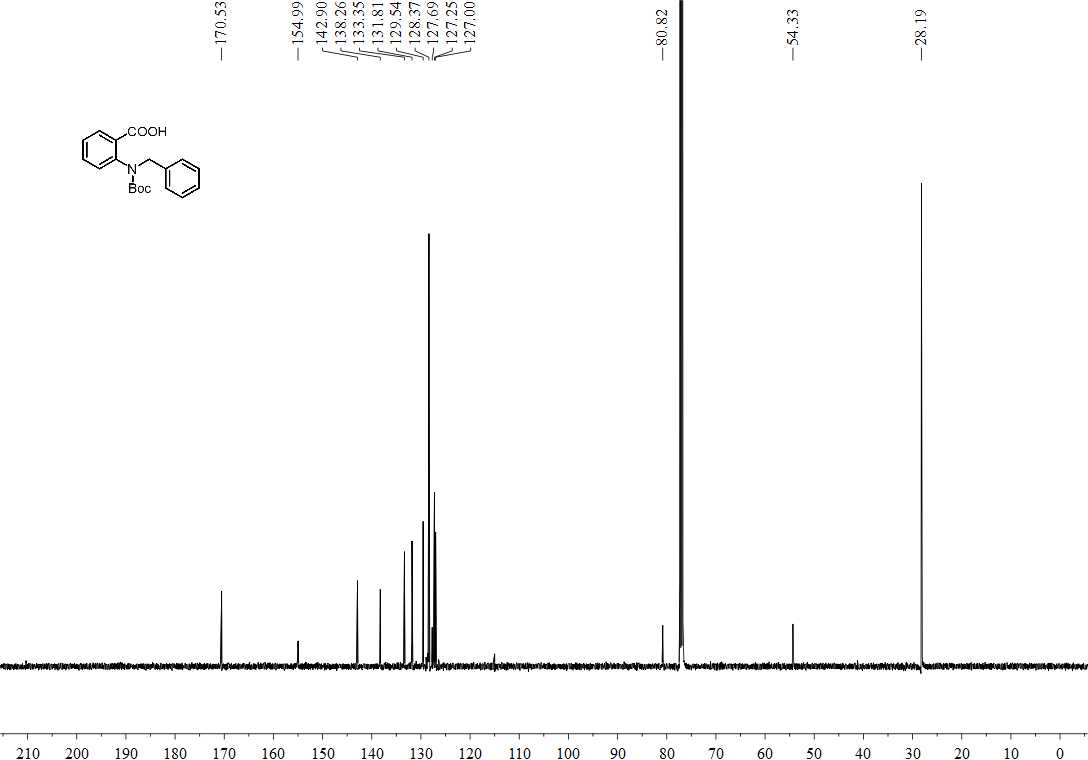


**Supplementary Figure 33.** ^13^C NMR spectra of 2-(benzyl(*tert*-butoxycarbonyl)amino)benzoic acid (**1u**).

4-(Benzyloxy)-2',4'-difluoro-[1,1'-biphenyl]-3-carboxylic acid (1v)

^1^H NMR (400 MHz, CDCl_3_)


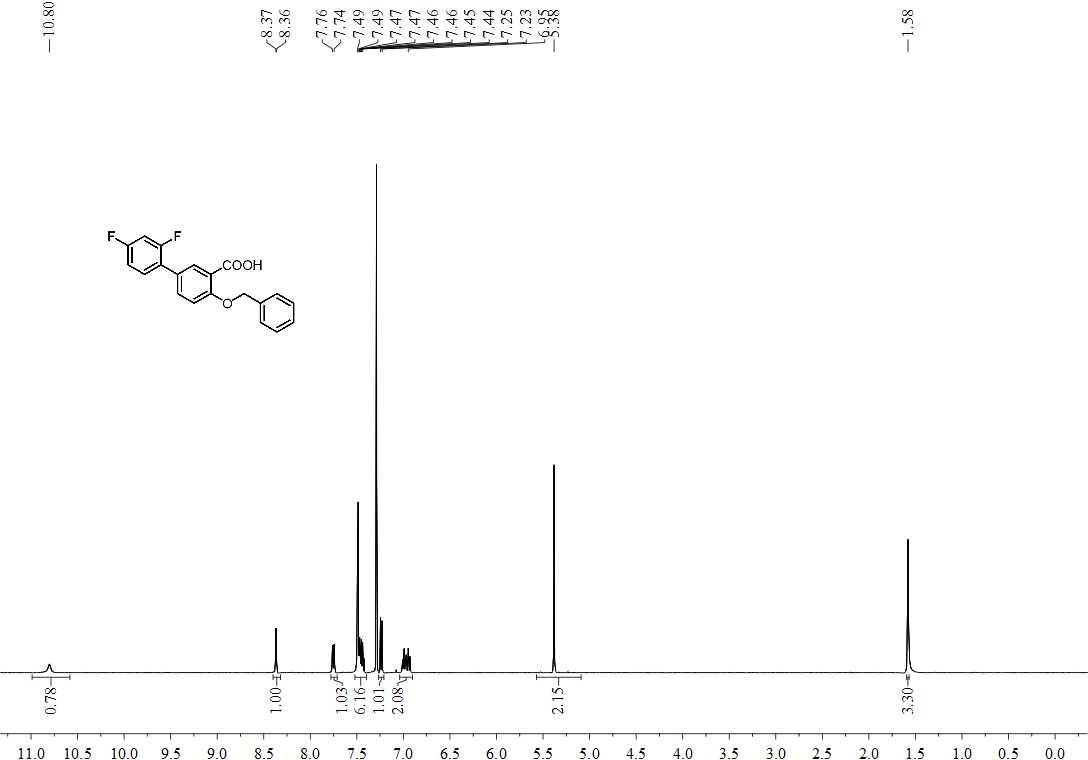


**Supplementary Figure 34.** ^1^H NMR spectra of 4-(benzyloxy)-2',4'-difluoro-[1,1'-biphenyl]-3-carboxylic acid (**1v**).

^13^C NMR (101 MHz, CDCl_3_)


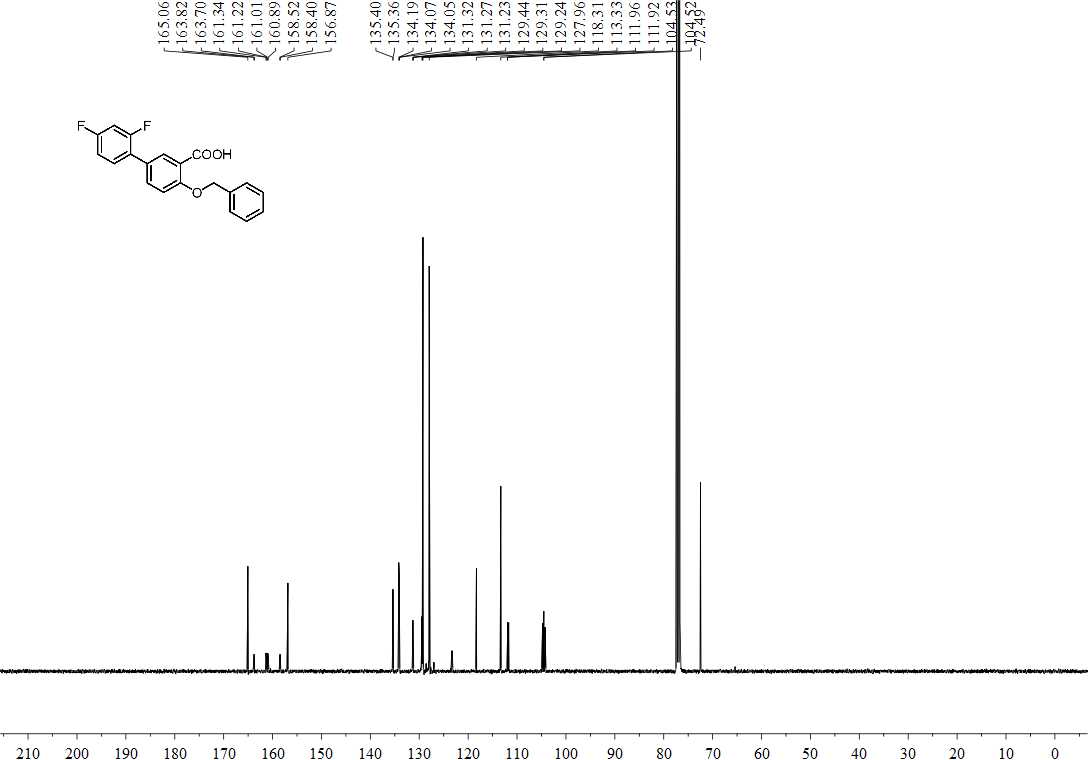


**Supplementary Figure 35.** ^13^C NMR spectra of 4-(benzyloxy)-2',4'-difluoro-[1,1'-biphenyl]-3-carboxylic acid (**1v**).

^19^F NMR (377 MHz, CDCl_3_)


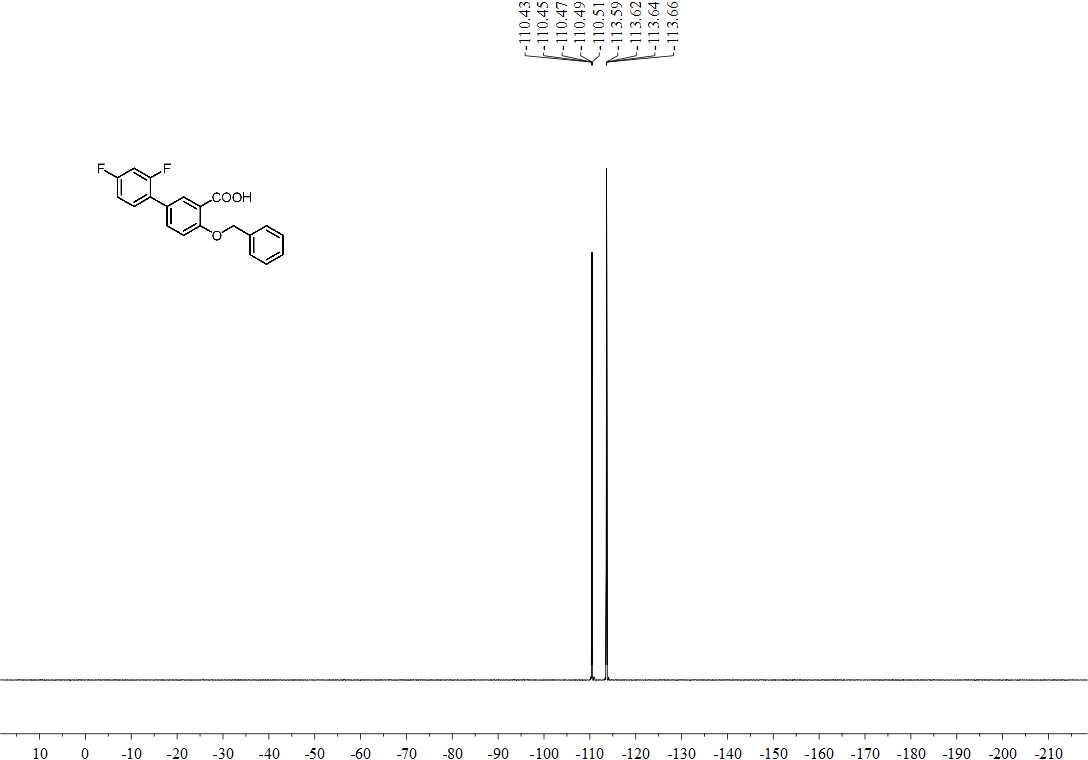


**Supplementary Figure 36.** ^19^F NMR spectra of 4-(benzyloxy)-2',4'-difluoro-[1,1'-biphenyl]-3-carboxylic acid (**1v**).

(R)-4-Methoxy-2-(1-phenylethoxy)benzoic acid (1w)

^1^H NMR (400 MHz, CDCl_3_)


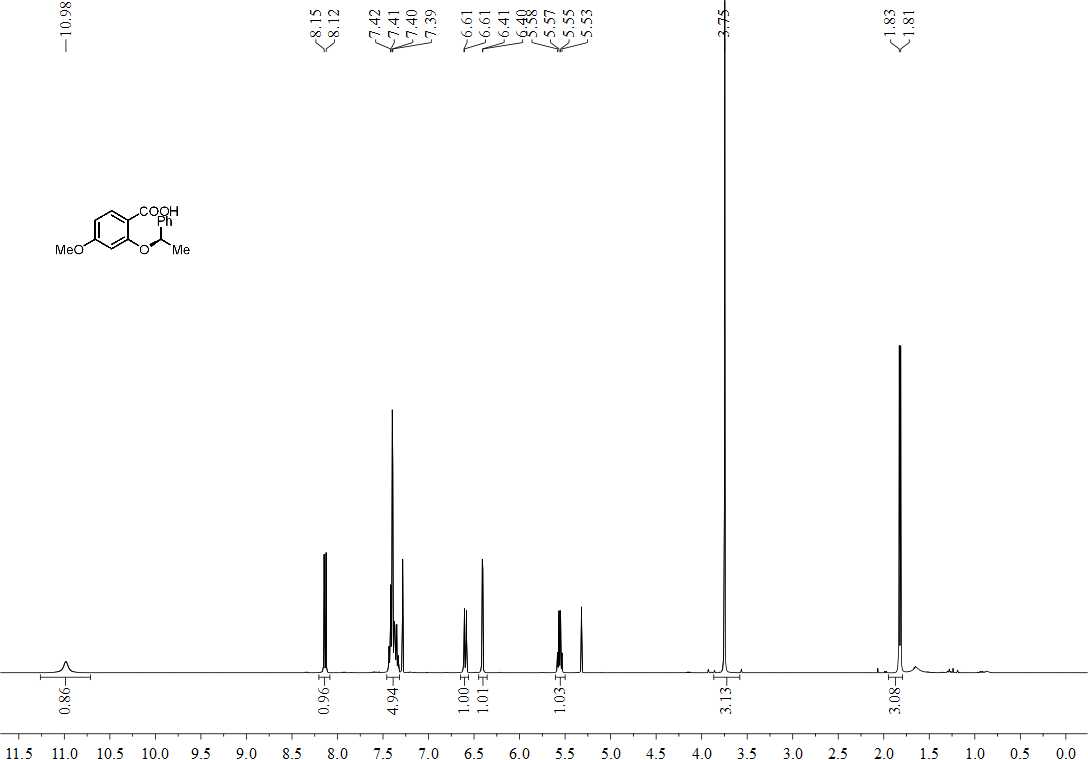


**Supplementary Figure 37.** ^1^H NMR spectra of (*R*)-4-methoxy-2-(1-phenylethoxy)benzoic acid (**1w**).

^13^C NMR (101 MHz, CDCl_3_)


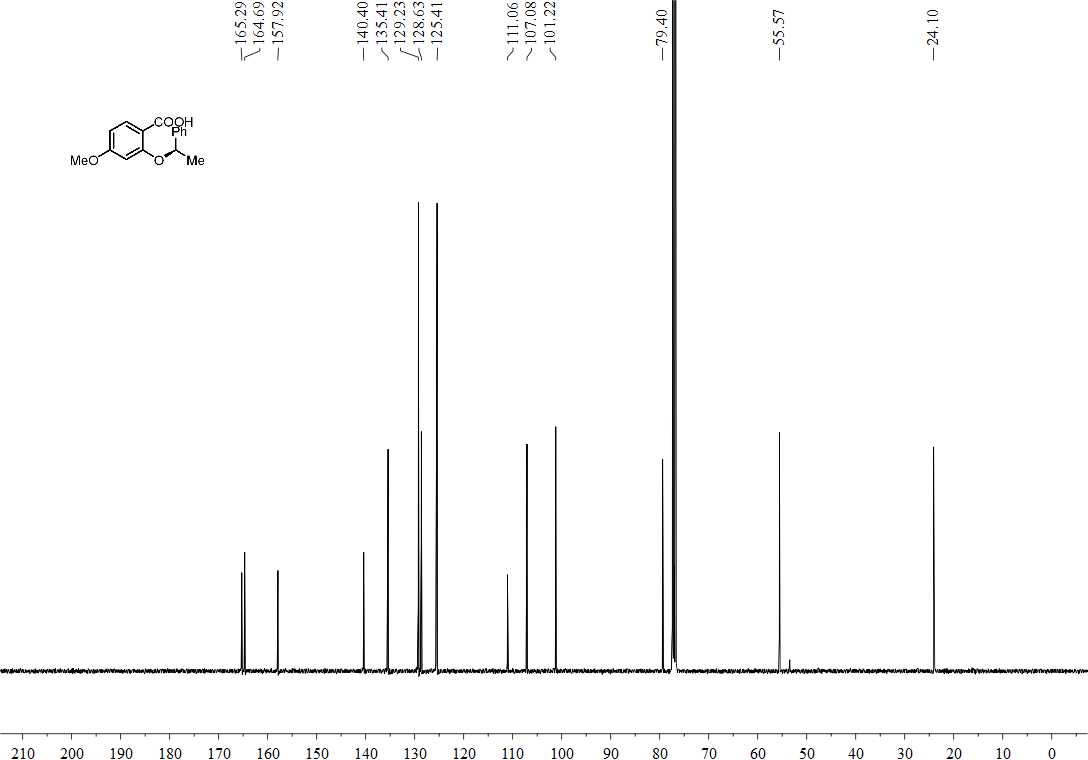


**Supplementary Figure 38.** ^13^C NMR spectra of (*R*)-4-methoxy-2-(1-phenylethoxy)benzoic acid (**1w**).

N-(tert-Butyl)-N-(2,6-dimethylbenzyl)acrylamide (6b)

^1^H NMR (400 MHz, CDCl_3_)


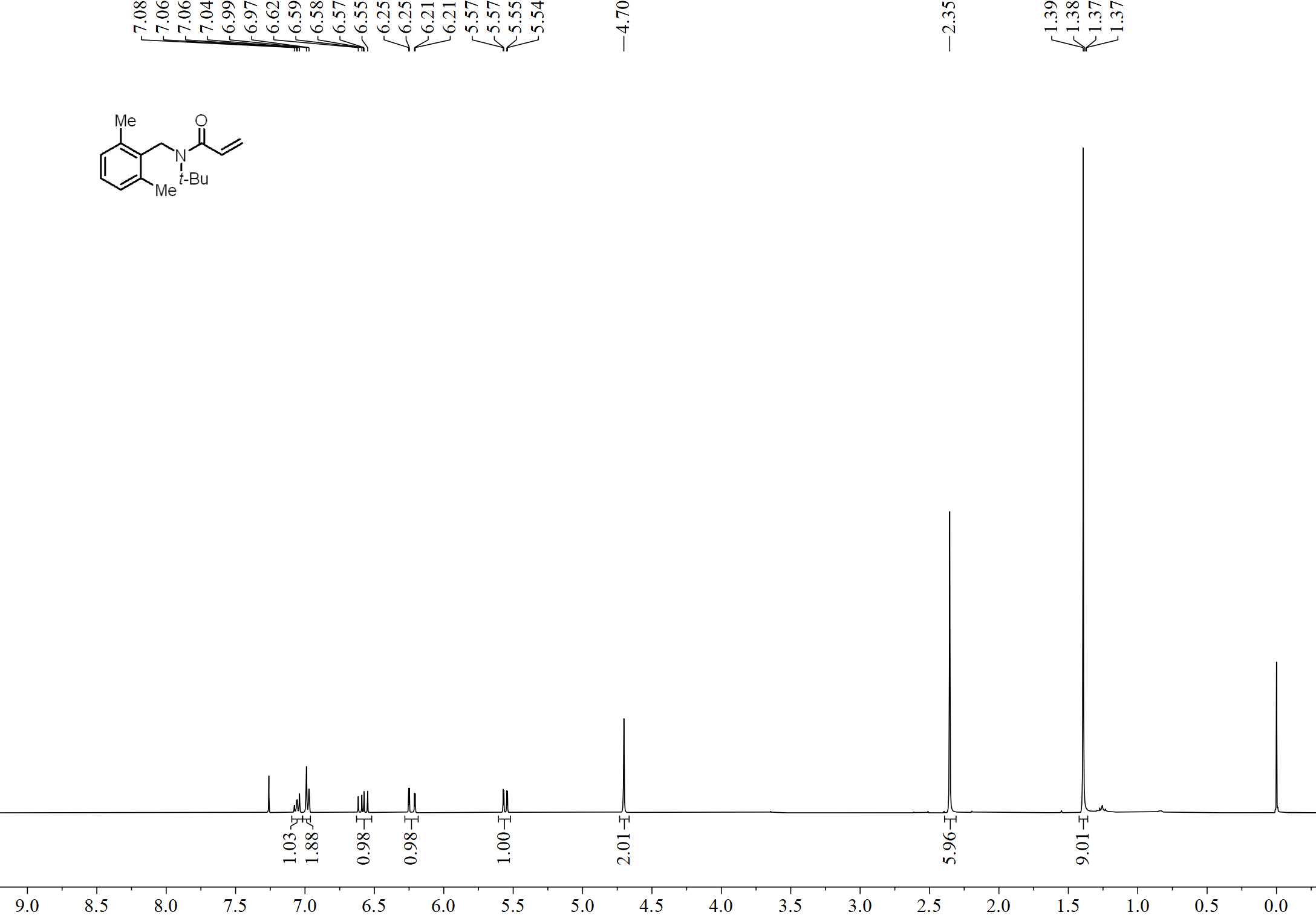


**Supplementary Figure 39.** ^1^H NMR spectra of *N*-(*tert*-butyl)-*N*-(2,6-dimethylbenzyl)acrylamide (**6b**).

^13^C NMR (101 MHz, CDCl_3_)


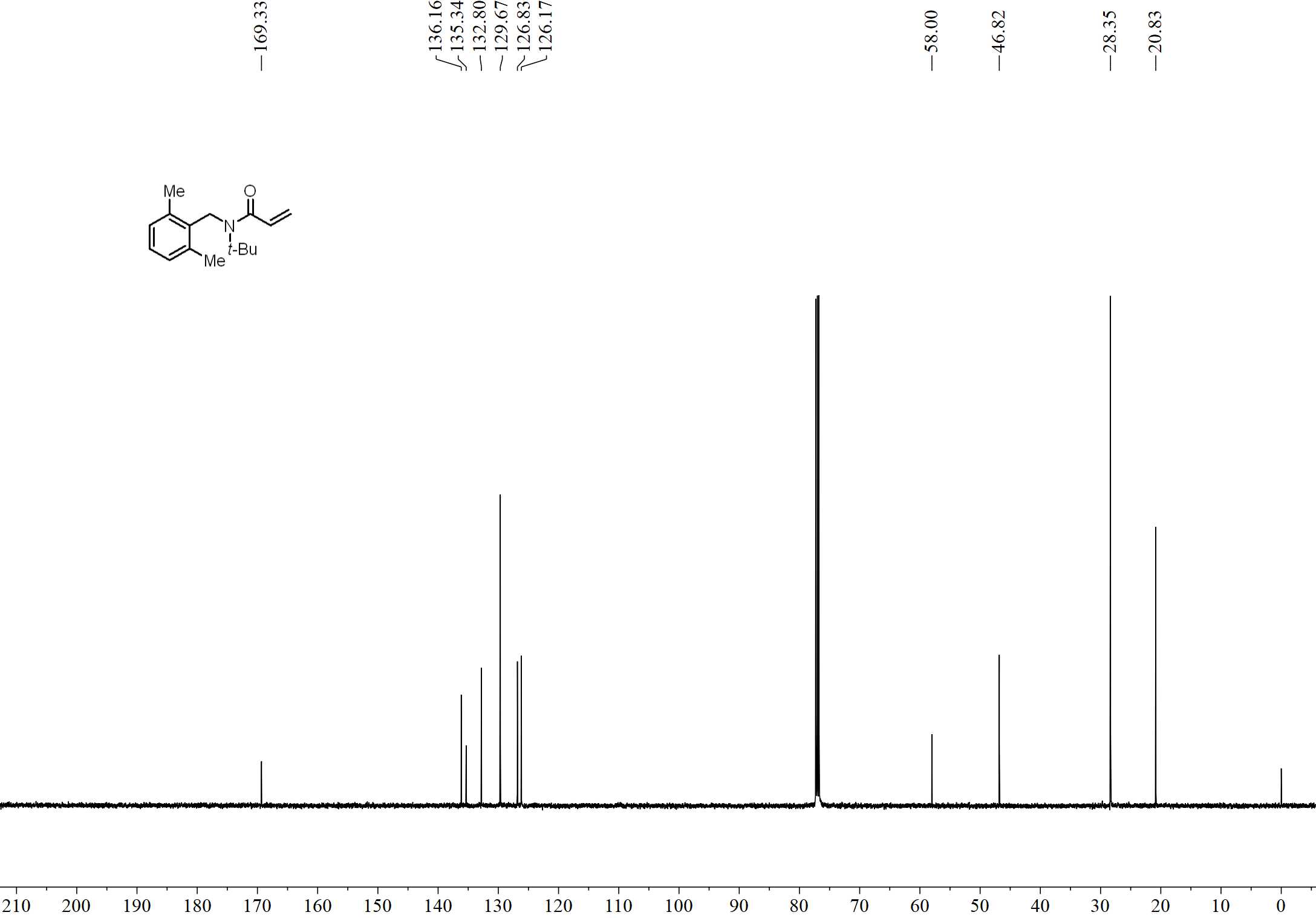


**Supplementary Figure 40.** ^1^H NMR spectra of *N*-(*tert*-butyl)-*N*-(2,6-dimethylbenzyl)acrylamide (**6b**).

N-(tert-Butyl)-N-(3,5-dimethoxybenzyl)acrylamide (6c)

^1^H NMR (400 MHz, CDCl_3_)


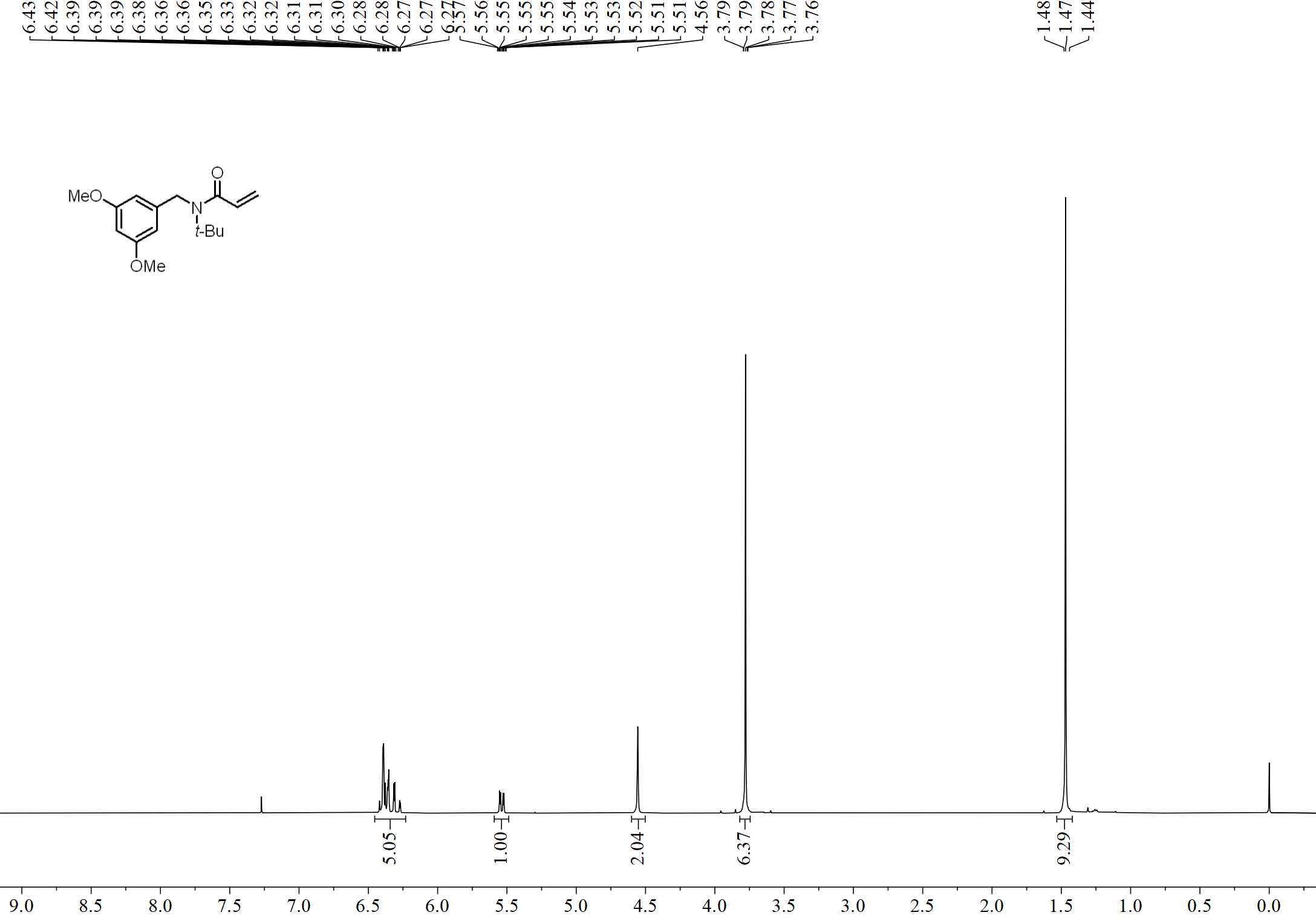


**Supplementary Figure 41.** ^1^H NMR spectra of *N*-(*tert*-butyl)-*N*-(3,5-dimethoxybenzyl)acrylamide (**6c**).

^13^C NMR (101 MHz, CDCl_3_)


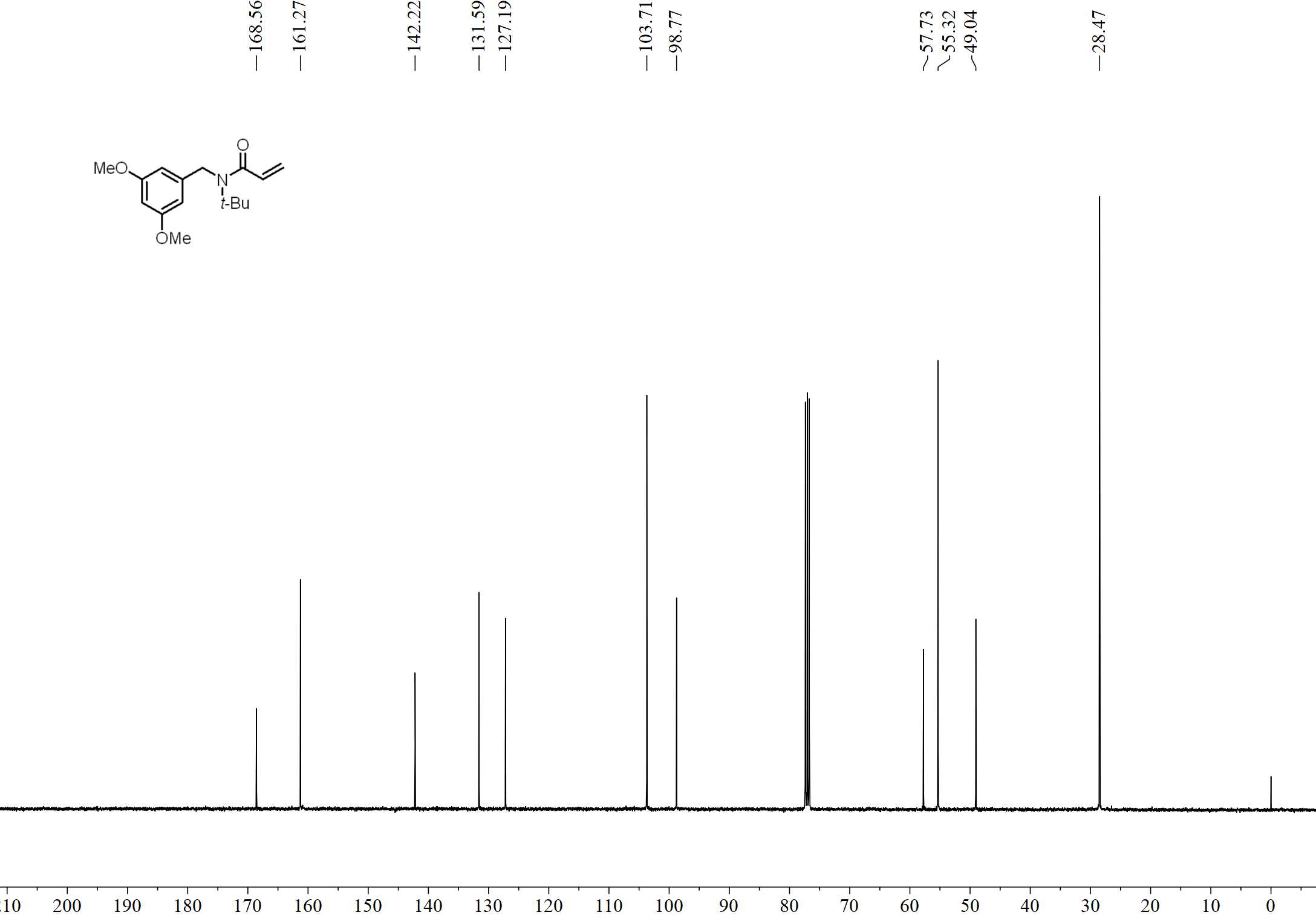


**Supplementary Figure 42.** ^13^C NMR spectra of *N*-(*tert*-butyl)-*N*-(3,5-dimethoxybenzyl)acrylamide (**6c**).

Spiro[chromane-3,1'-cyclohexane]-2',5'-dien-4-one (5a)

^1^H NMR (400 MHz, CDCl_3_)


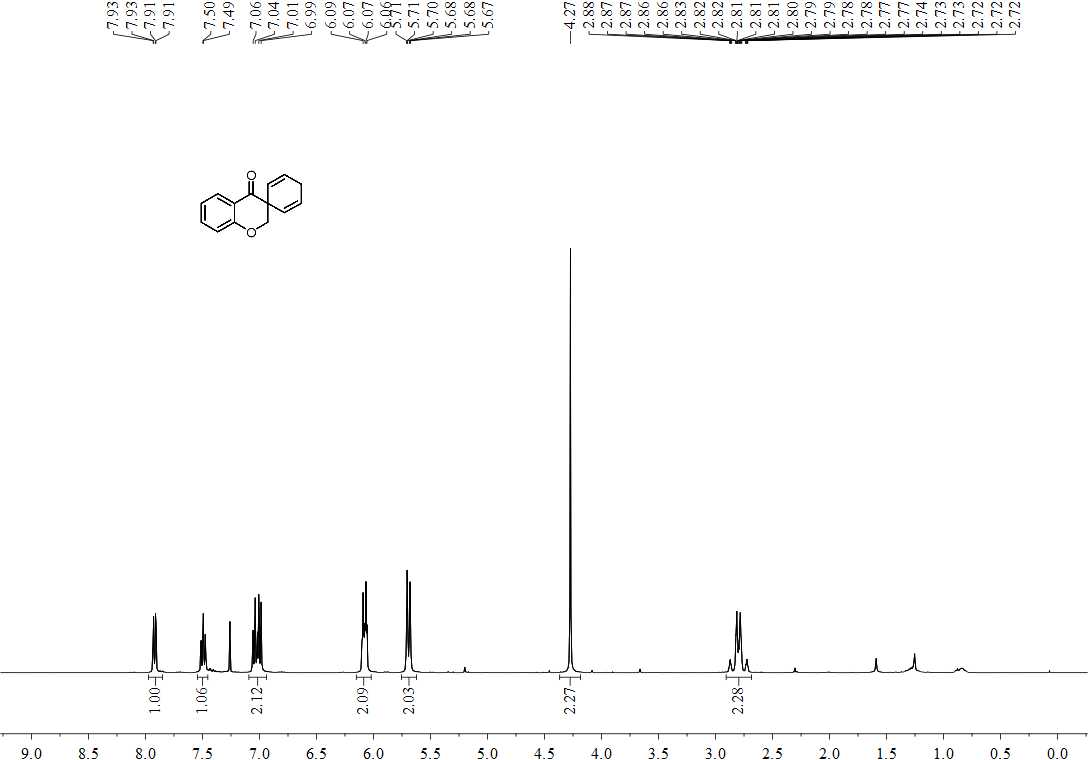


**Supplementary Figure 43.** ^1^H NMR spectra of spiro[chromane-3,1'-cyclohexane]-2',5'-dien-4-one (**5a**).

^13^C NMR (101 MHz, CDCl_3_)


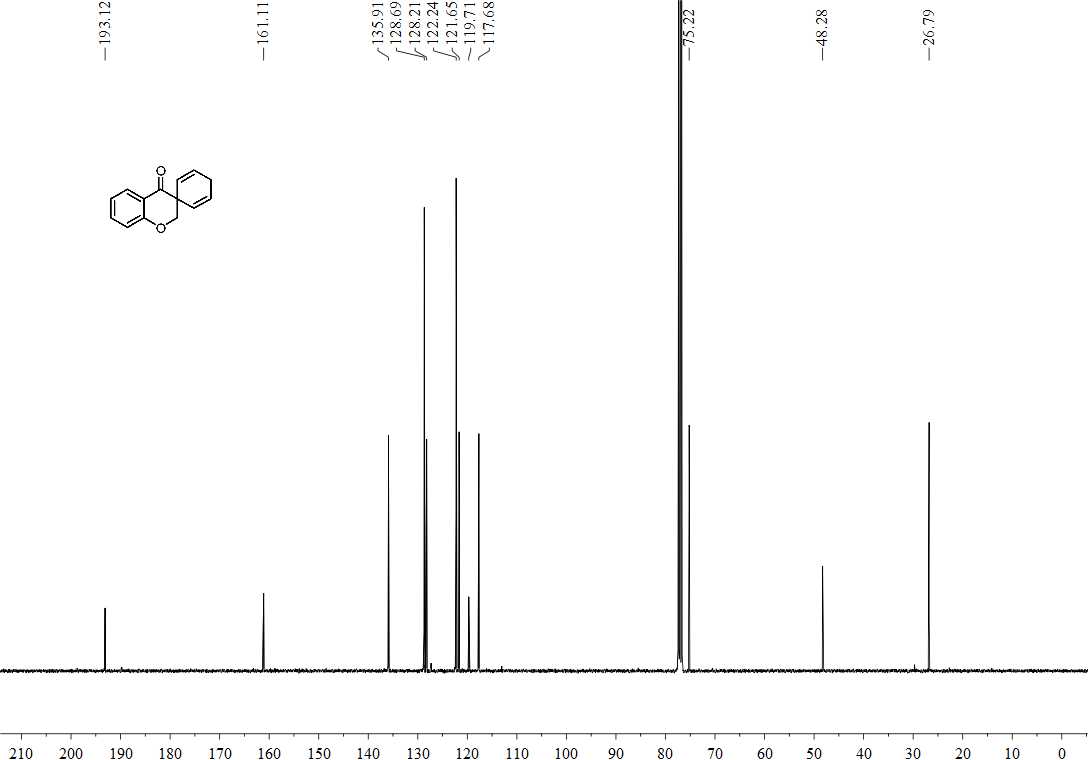


**Supplementary Figure 44.** ^13^C NMR spectra of spiro[chromane-3,1'-cyclohexane]-2',5'-dien-4-one (**5a**).

7-Methoxyspiro[chromane-3,1'-cyclohexane]-2',5'-dien-4-one (5b)

^1^H NMR (400 MHz, CDCl_3_)


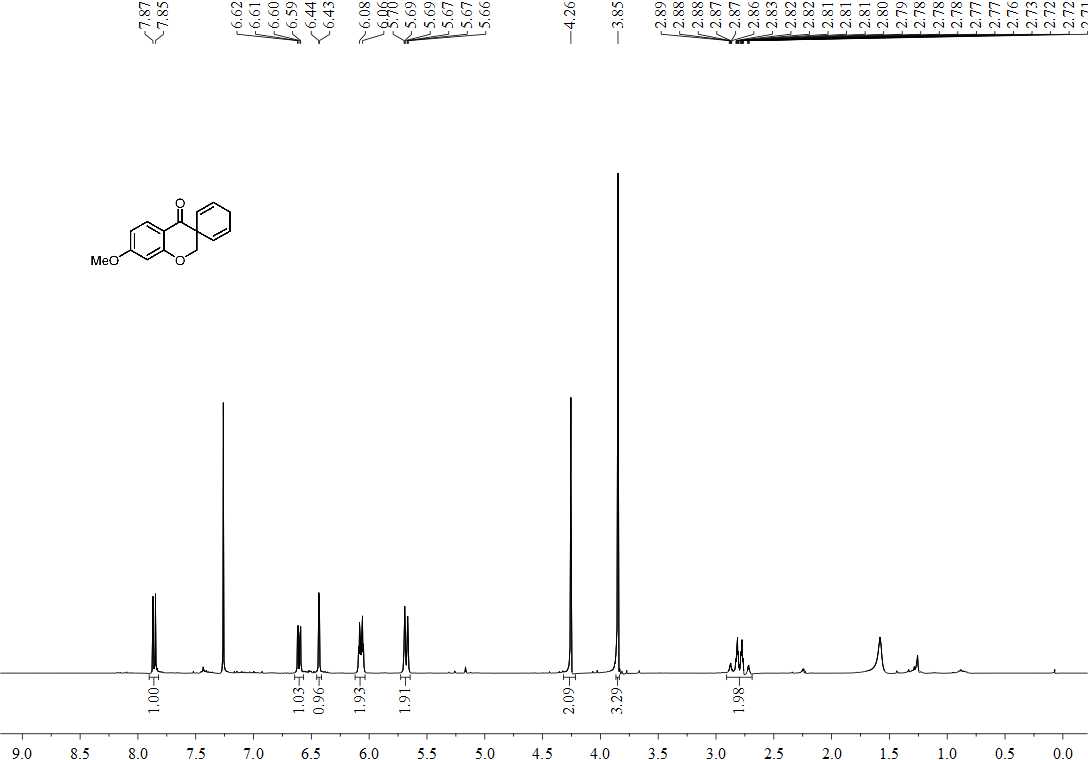


**Supplementary Figure 45.** ^1^H NMR spectra of 7-methoxyspiro[chromane-3,1'-cyclohexane]-2',5'-dien-4-one (**5b**).

^13^C NMR (101 MHz, CDCl_3_)


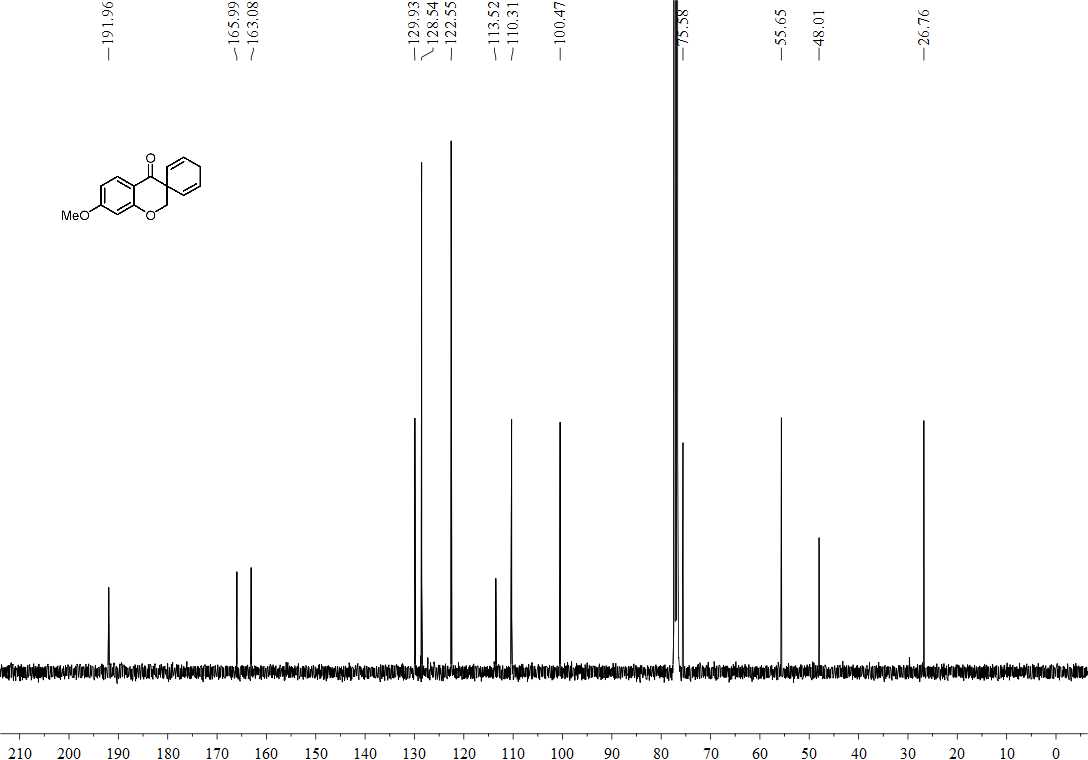


**Supplementary Figure 46.** ^13^C NMR spectra of 7-methoxyspiro[chromane-3,1'-cyclohexane]-2',5'-dien-4-one (**5b**).

7-Methylspiro[chromane-3,1'-cyclohexane]-2',5'-dien-4-one (5c)

^1^H NMR (400 MHz, CD_3_CN)


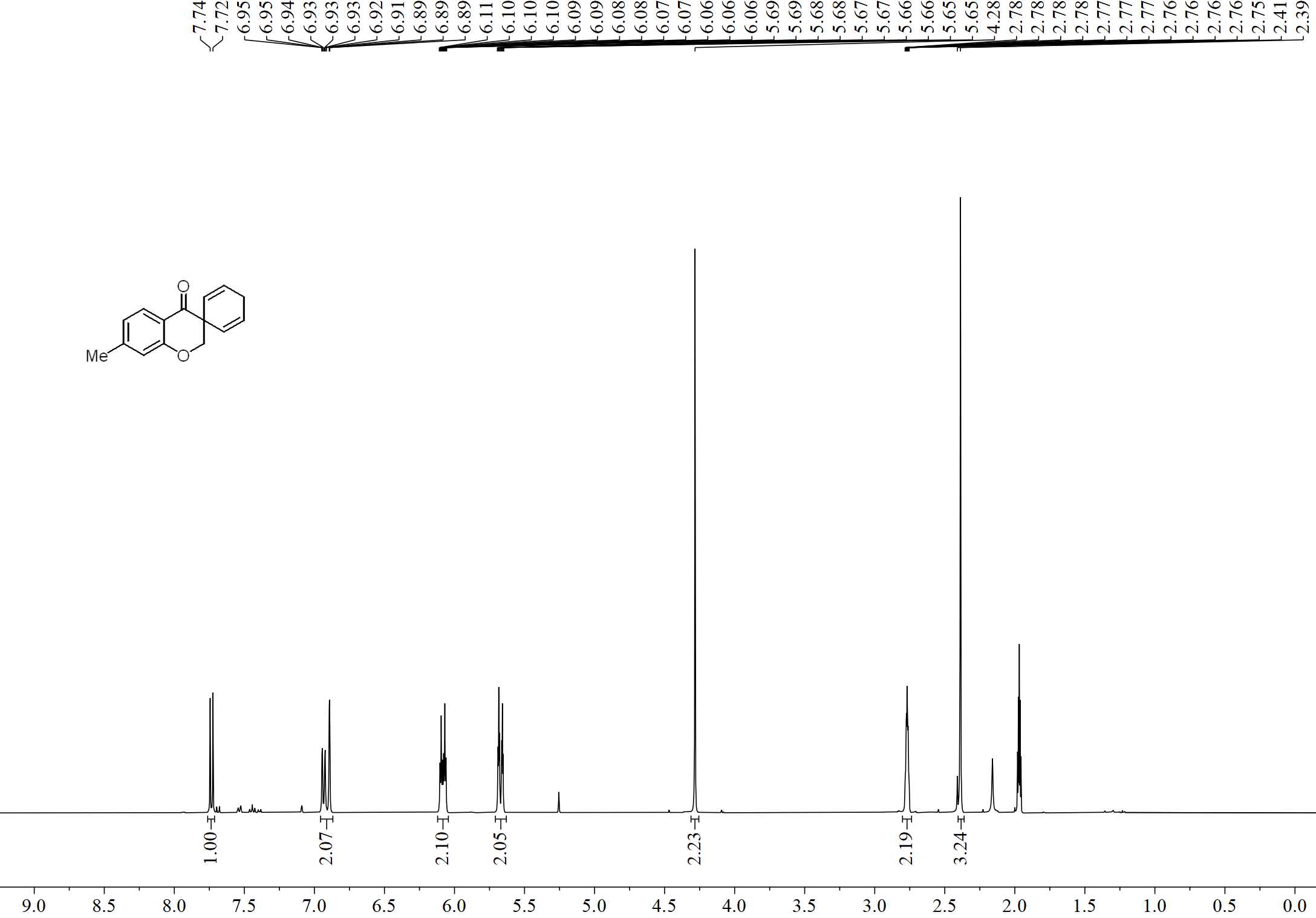


**Supplementary Figure 47.** ^1^H NMR spectra of 7-methylspiro[chromane-3,1'-cyclohexane]-2',5'-dien-4-one (**5c**).

^13^C NMR (126 MHz, CD_3_CN)


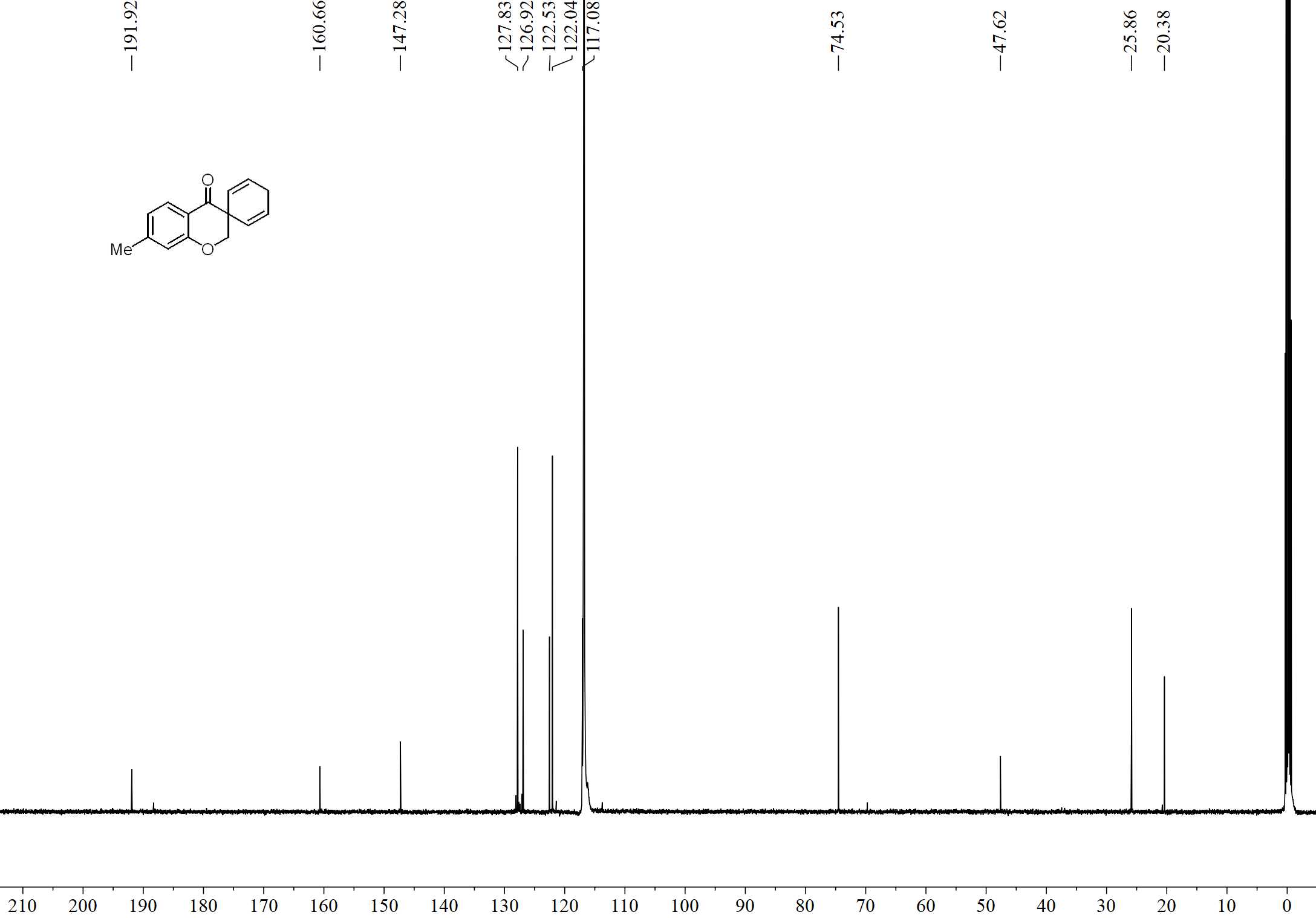


**Supplementary Figure 48.** ^13^C NMR spectra of 7-methylspiro[chromane-3,1'-cyclohexane]-2',5'-dien-4-one (**5c**).

8-Phenylspiro[chromane-3,1'-cyclohexane]-2',5'-dien-4-one (5d)

**^1^**^1^H NMR (400 MHz, CDCl_3_)


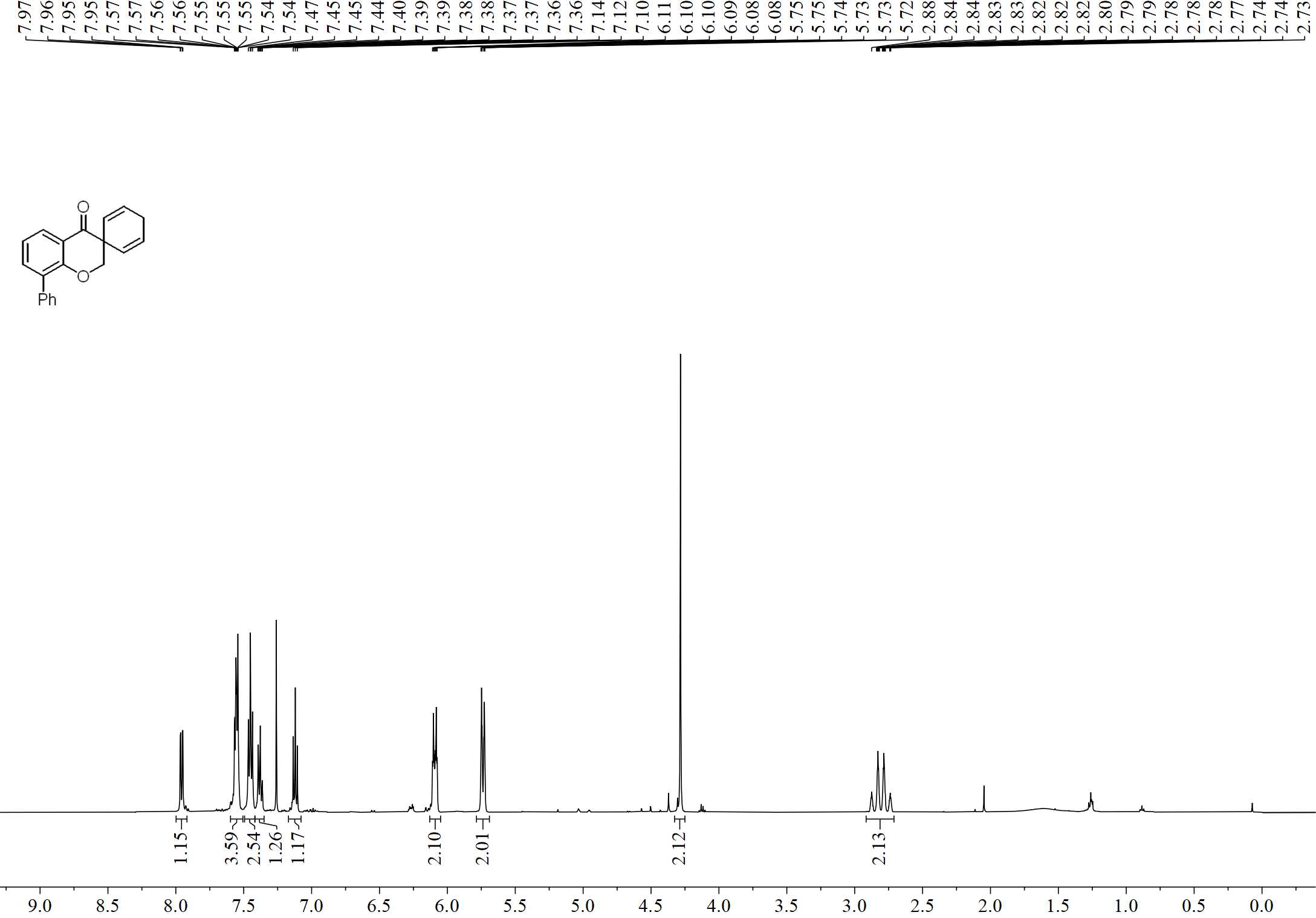


**Supplementary Figure 49.** ^1^H NMR spectra of 8-phenylspiro[chromane-3,1'-cyclohexane]-2',5'-dien-4-one (**5d**).

^13^C NMR (126 MHz, CDCl_3_)


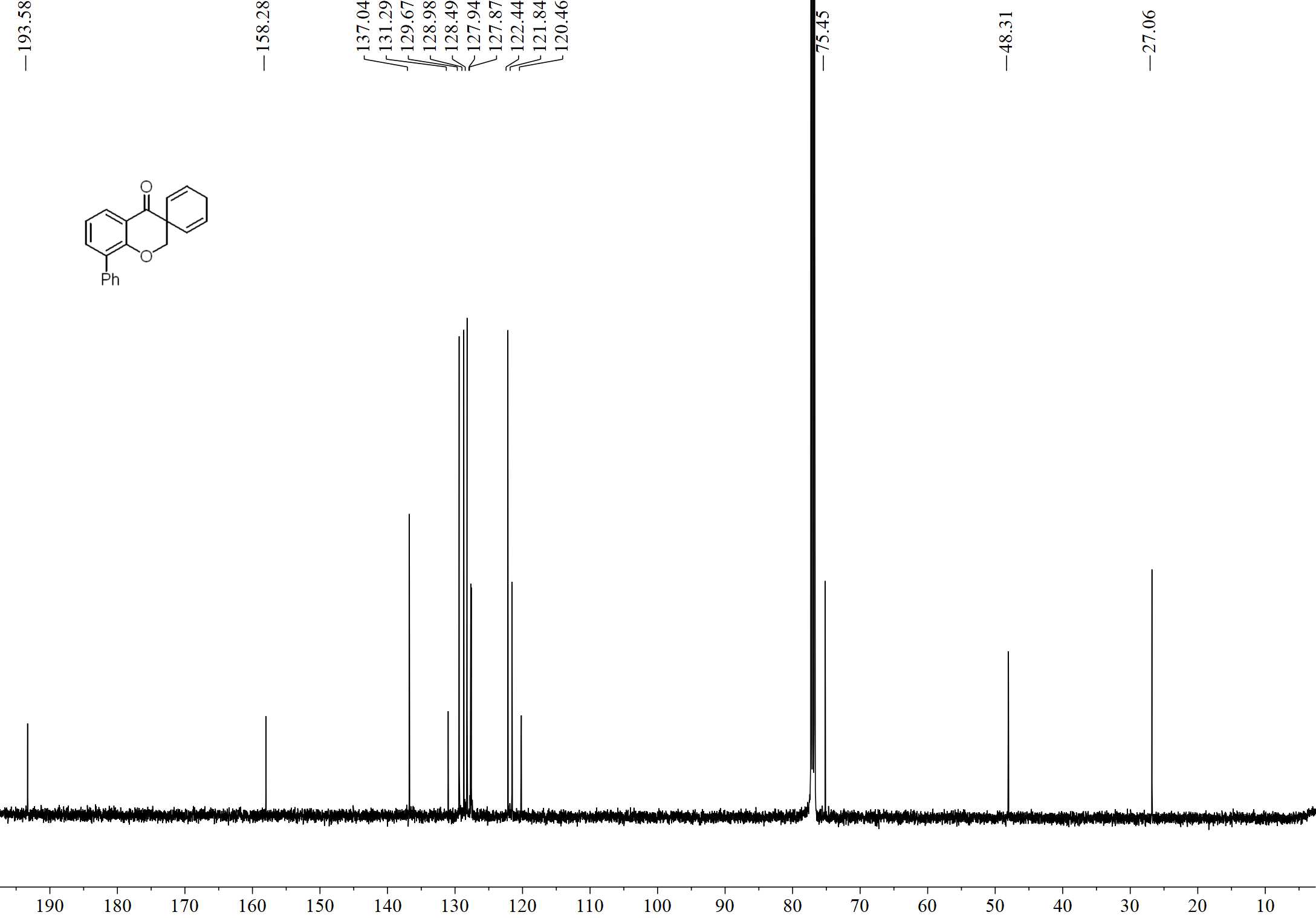


**Supplementary Figure 50.** ^13^C NMR spectra of 8-phenylspiro[chromane-3,1'-cyclohexane]-2',5'-dien-4-one (**5d**).

N-(4-Oxospiro[chromane-3,1'-cyclohexane]-2',5'-dien-7-yl)acetamide (5e)

^1^H NMR (400 MHz, CDCl_3_)


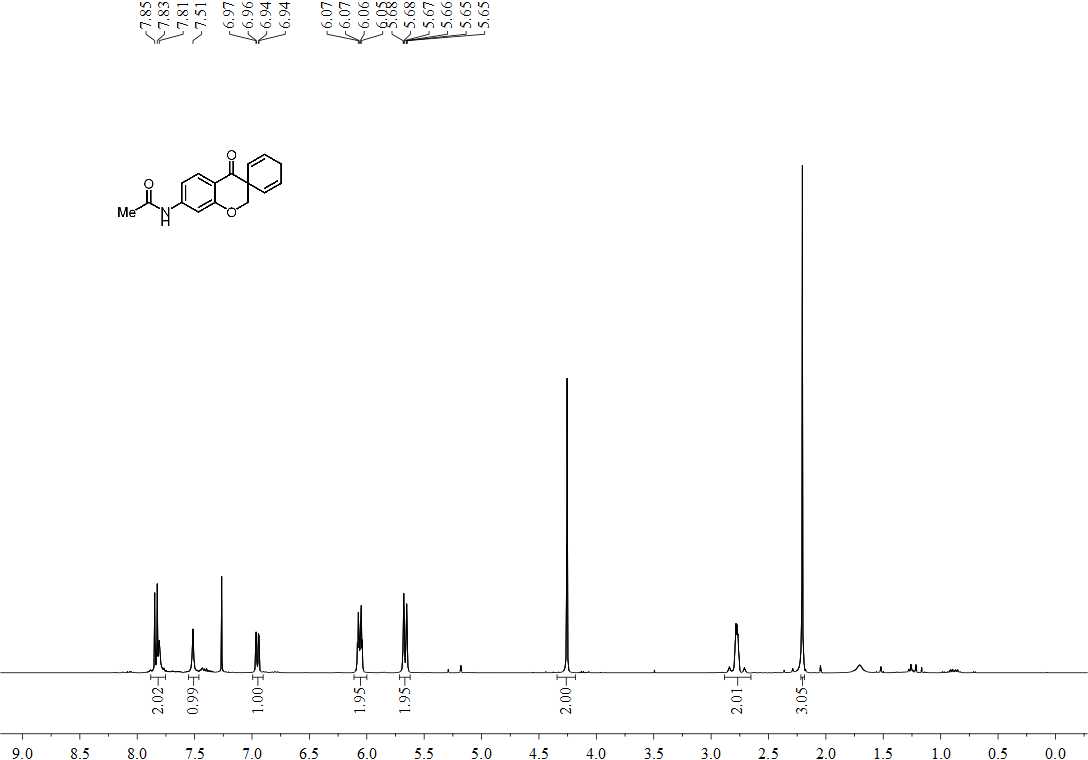


**Supplementary Figure 51.** ^1^H NMR spectra of *N*-(4-oxospiro[chromane-3,1'-cyclohexane]-2',5'-dien-7-yl)acetamide (**5e**).

^13^C NMR (101 MHz, CDCl_3_)


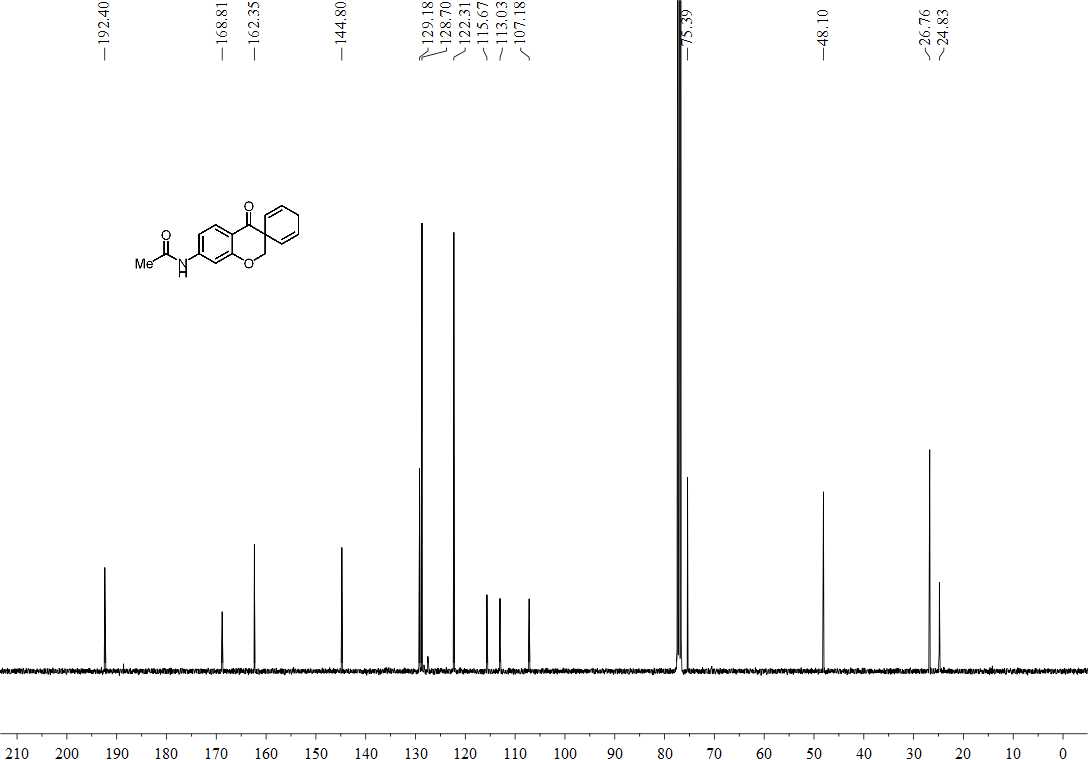


**Supplementary Figure 52.** ^13^C NMR spectra of *N*-(4-oxospiro[chromane-3,1'-cyclohexane]-2',5'-dien-7-yl)acetamide (**5e**).

8-Methoxyspiro[chromane-3,1'-cyclohexane]-2',5'-dien-4-one (5f)

^1^H NMR (400 MHz, CDCl_3_)


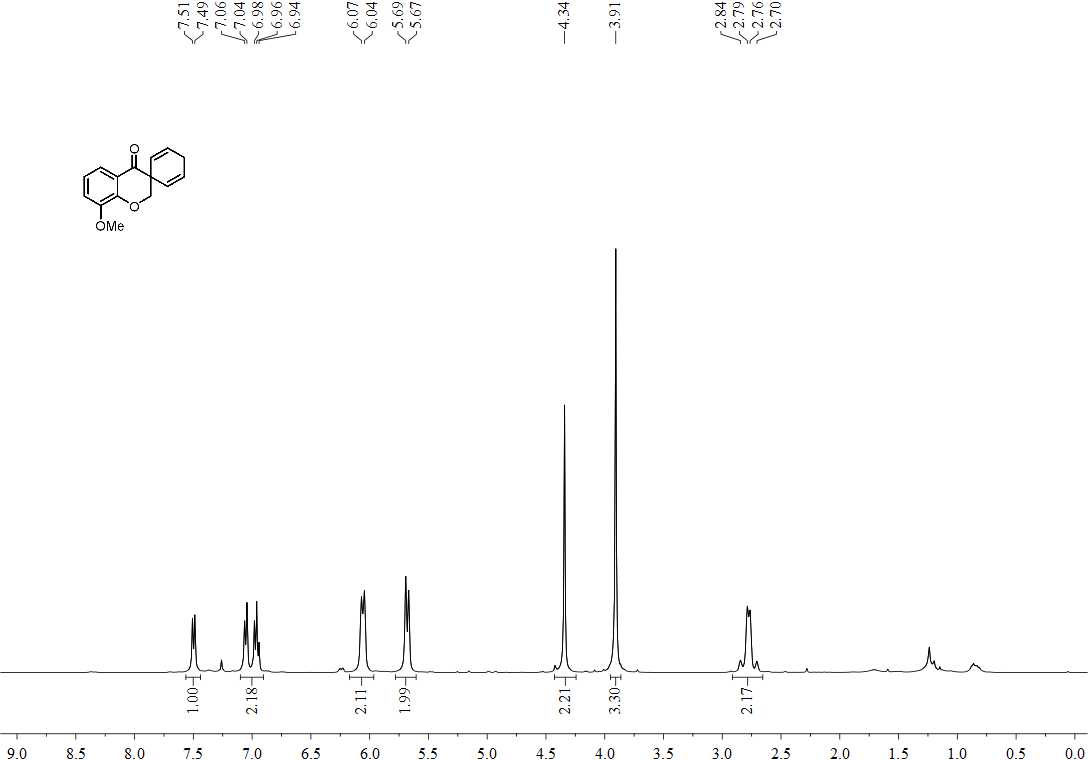


**Supplementary Figure 53.** ^1^H NMR spectra of 8-methoxyspiro[chromane-3,1'-cyclohexane]-2',5'-dien-4-one (**5f**).

^13^C NMR (101 MHz, CDCl_3_)


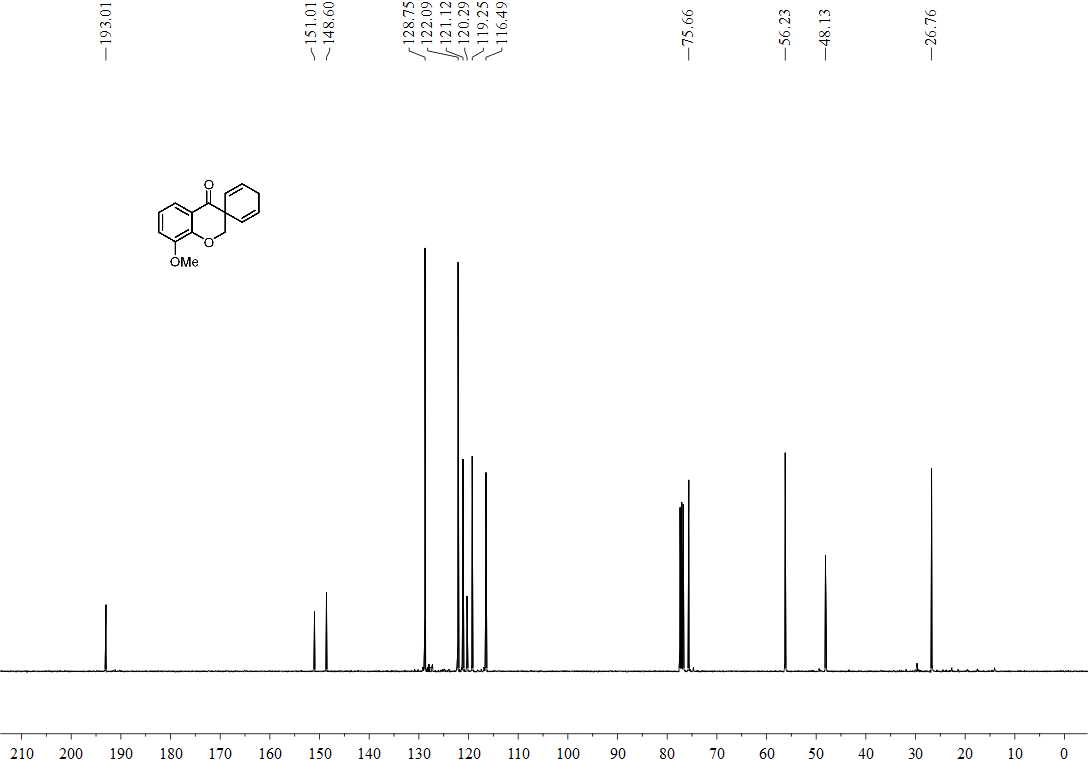


**Supplementary Figure 54.** ^1^H NMR spectra of 8-methoxyspiro[chromane-3,1'-cyclohexane]-2',5'-dien-4-one (**5f**).

6-Methoxyspiro[chromane-3,1'-cyclohexane]-2',5'-dien-4-one (5g)

^1^H NMR (400 MHz, CD_3_CN)


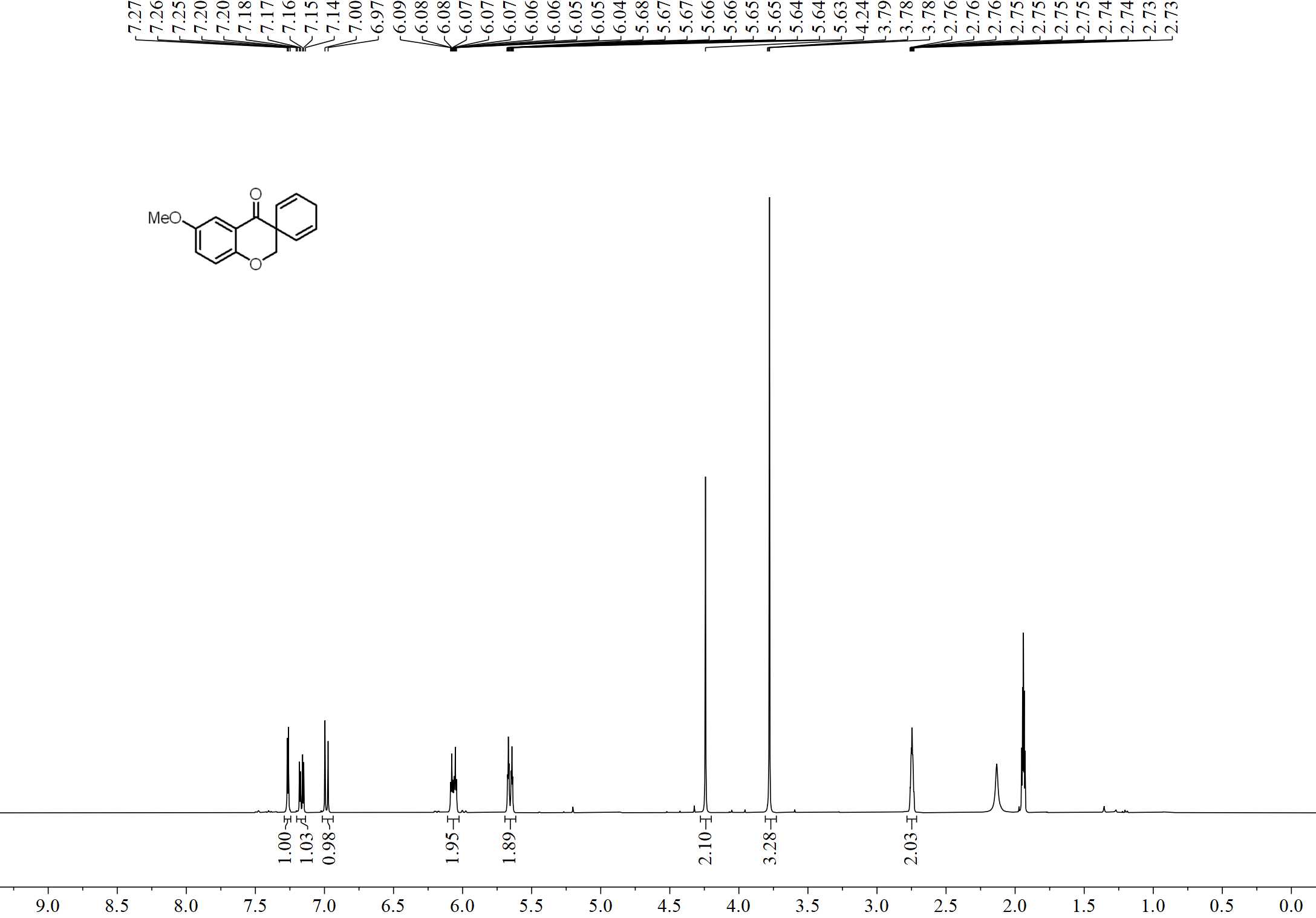


**Supplementary Figure 55.** ^1^H NMR spectra of 6-methoxyspiro[chromane-3,1'-cyclohexane]-2',5'-dien-4-one (**5g**).

^13^C NMR (126 MHz, CD_3_CN)


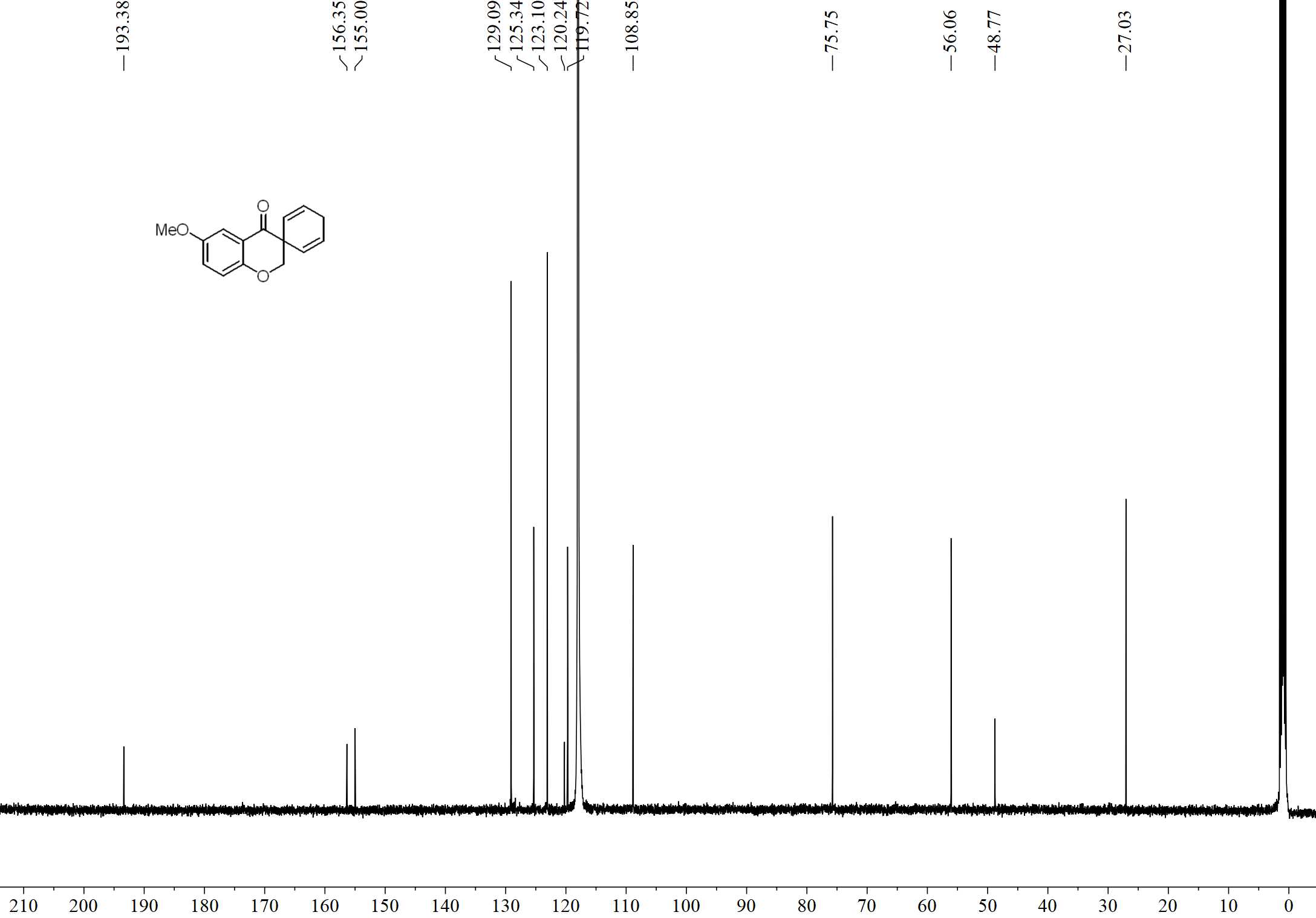


**Supplementary Figure 56.** ^13^C NMR spectra of 6-methoxyspiro[chromane-3,1'-cyclohexane]-2',5'-dien-4-one (**5g**).

5-Methoxyspiro[chromane-3,1'-cyclohexane]-2',5'-dien-4-one (5h)

^1^H NMR (400 MHz, CDCl_3_)


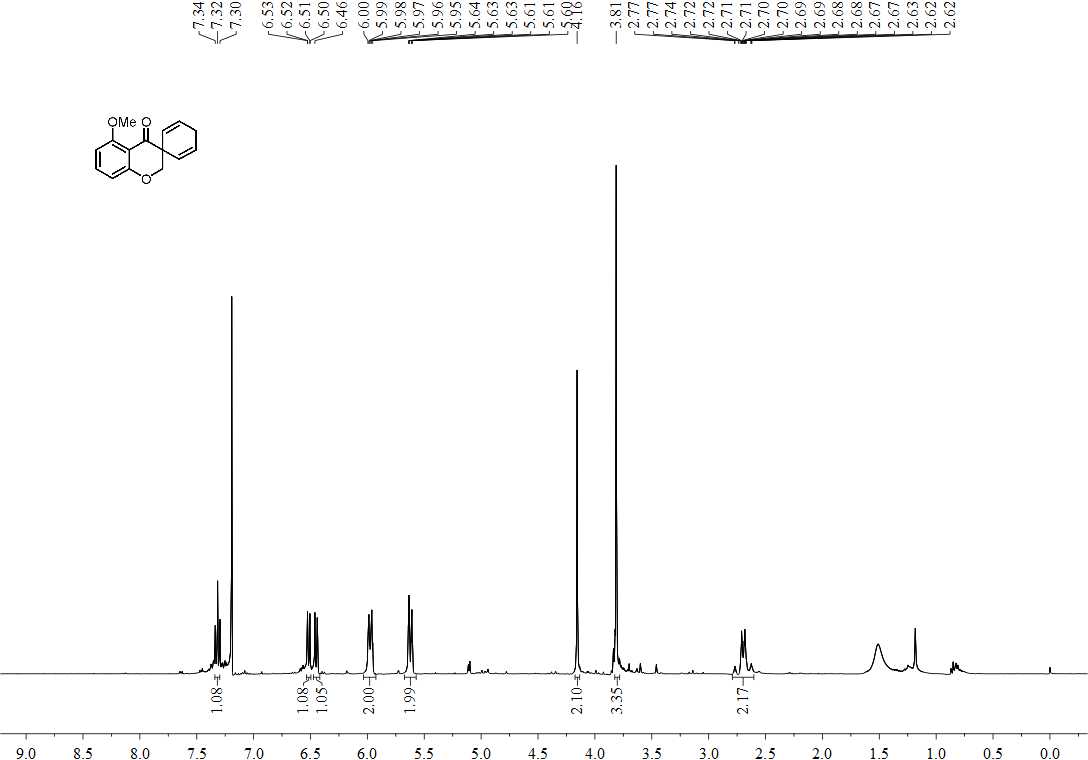


**Supplementary Figure 57.** ^1^H NMR spectra of 5-methoxyspiro[chromane-3,1'-cyclohexane]-2',5'-dien-4-one (**5h**).

^13^C NMR (101 MHz, CDCl_3_)


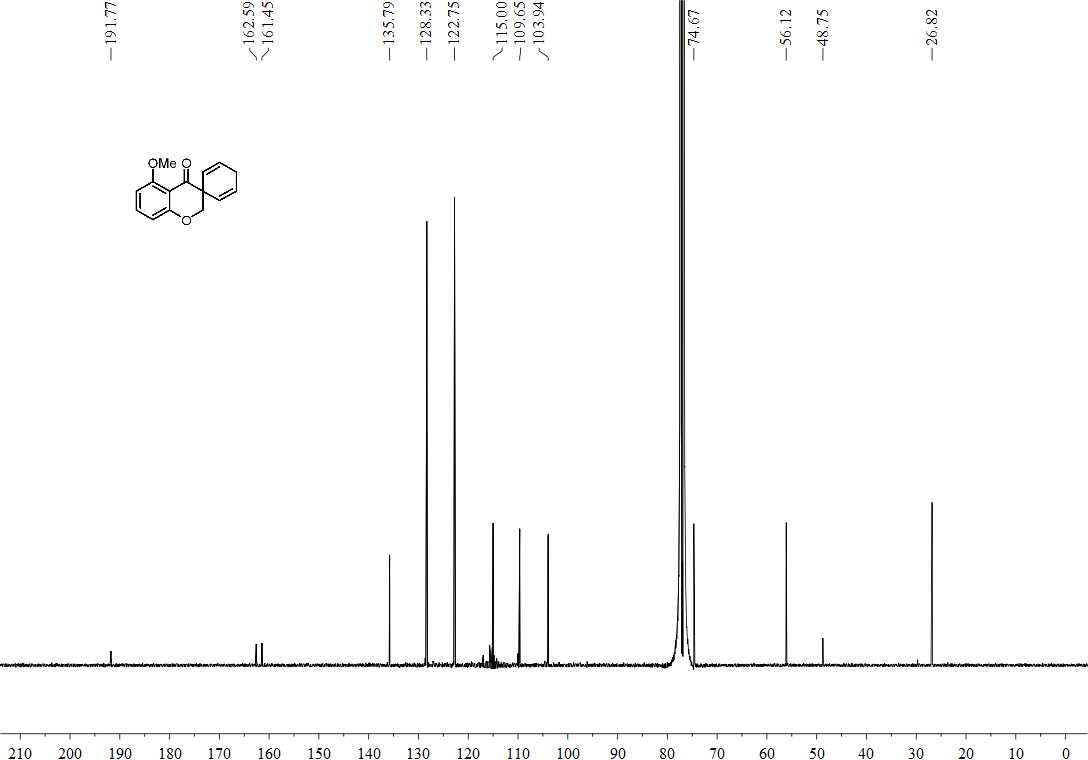


**Supplementary Figure 58.** ^13^C NMR spectra of 5-methoxyspiro[chromane-3,1'-cyclohexane]-2',5'-dien-4-one (**5h**).

7-Chlorospiro[chromane-3,1'-cyclohexane]-2',5'-dien-4-one (5i)

^1^H NMR (400 MHz, CD_3_CN)


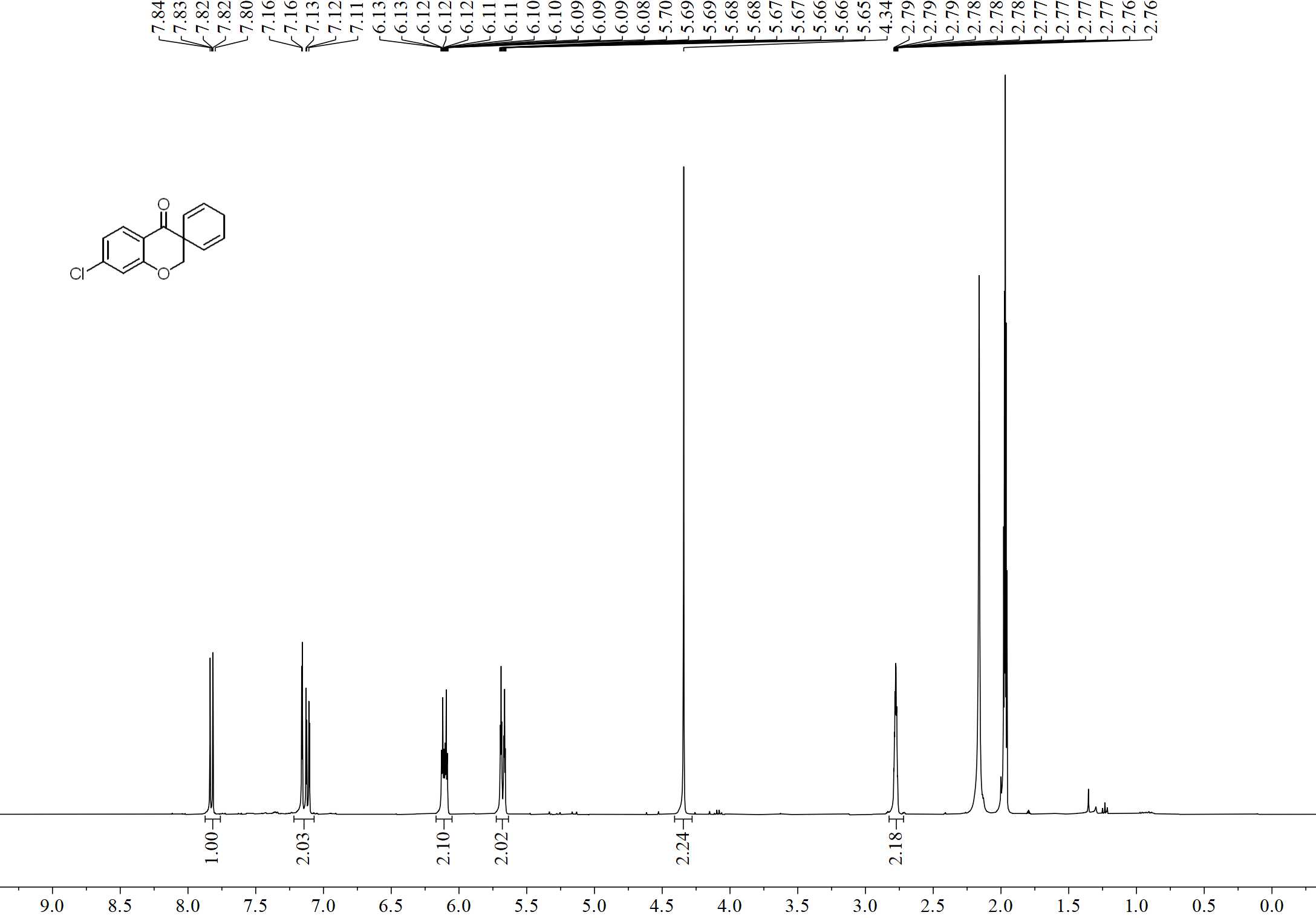


**Supplementary Figure 59.** ^1^H NMR spectra of 7-chlorospiro[chromane-3,1'-cyclohexane]-2',5'-dien-4-one (**5i**).

^13^C NMR (126 MHz, CD_3_CN)


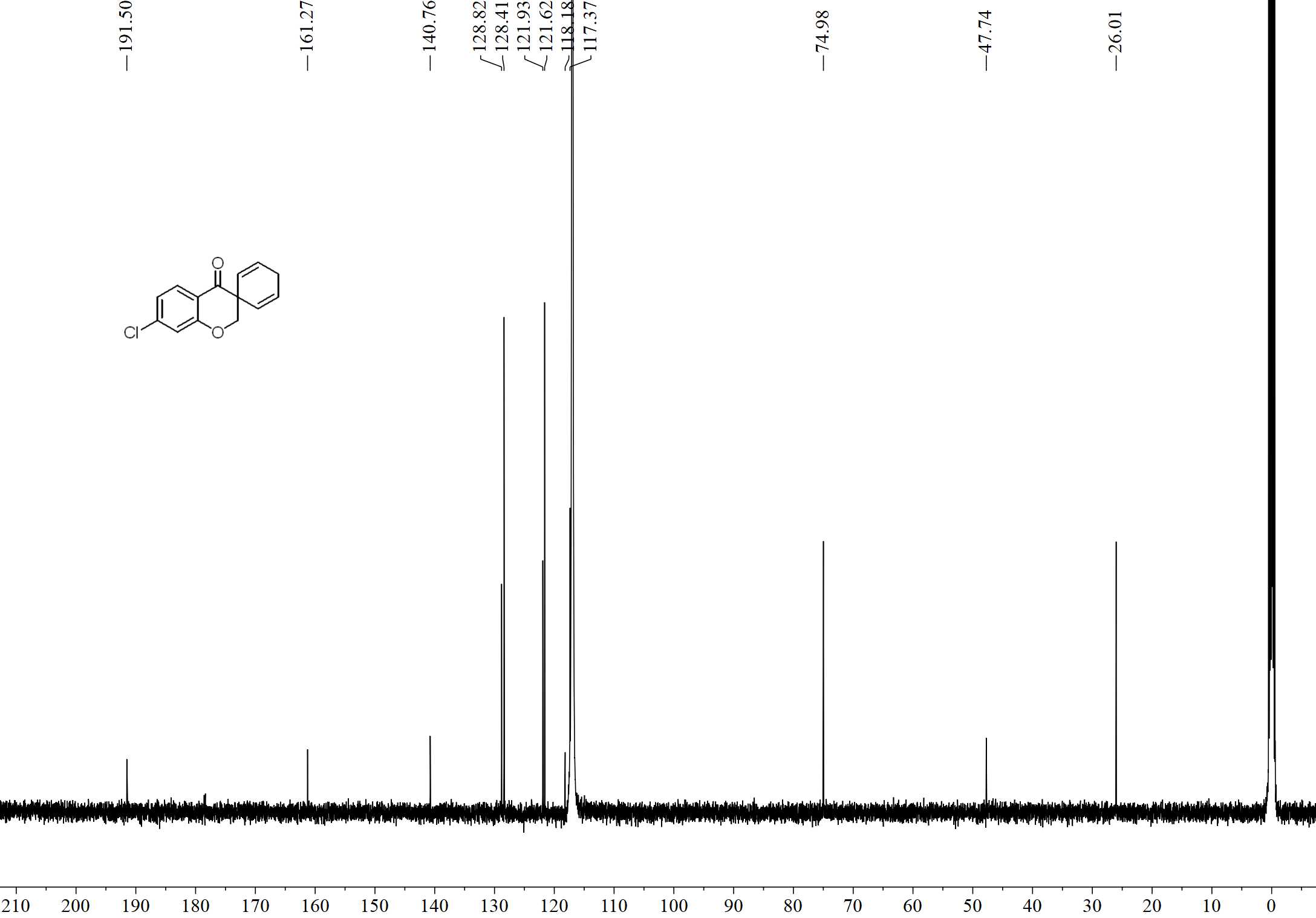


**Supplementary Figure 60.** ^1^H NMR spectra of 7-chlorospiro[chromane-3,1'-cyclohexane]-2',5'-dien-4-one (**5i**).

6-Acetylspiro[chromane-3,1'-cyclohexane]-2',5'-dien-4-one (5j)

^1^H NMR (400 MHz, CDCl_3_)


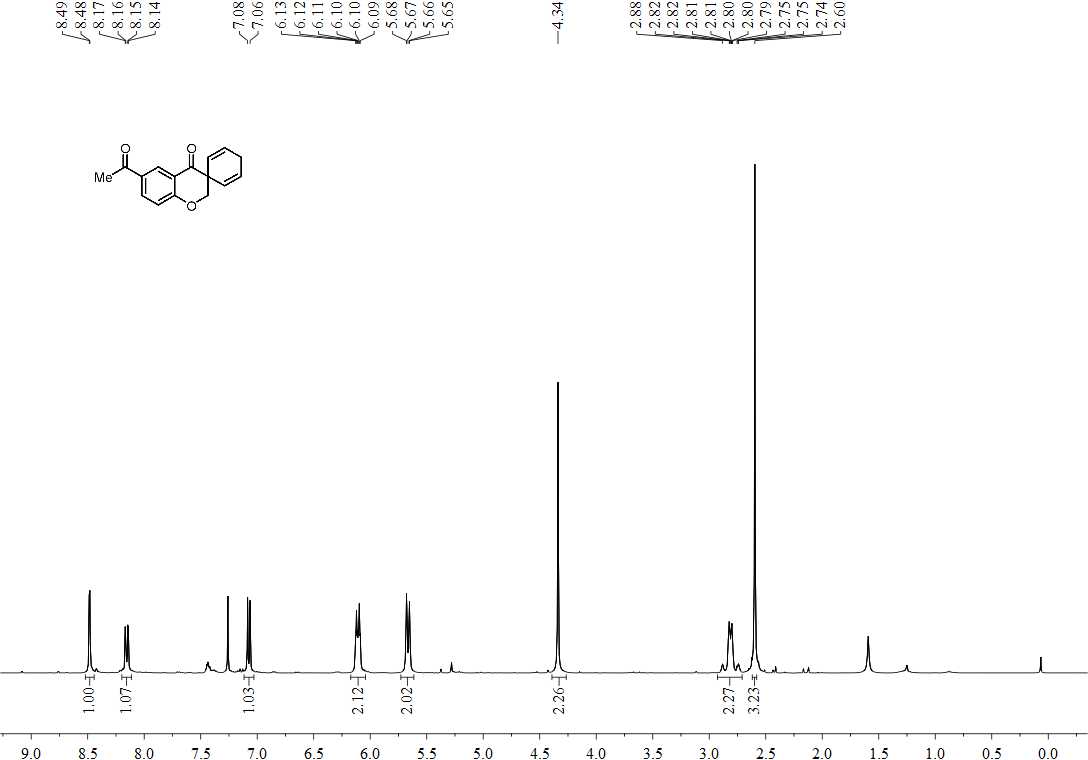


**Supplementary Figure 61.** ^1^H NMR spectra of 6-acetylspiro[chromane-3,1'-cyclohexane]-2',5'-dien-4-one (**5j**).

^13^C NMR (101 MHz, CDCl_3_)


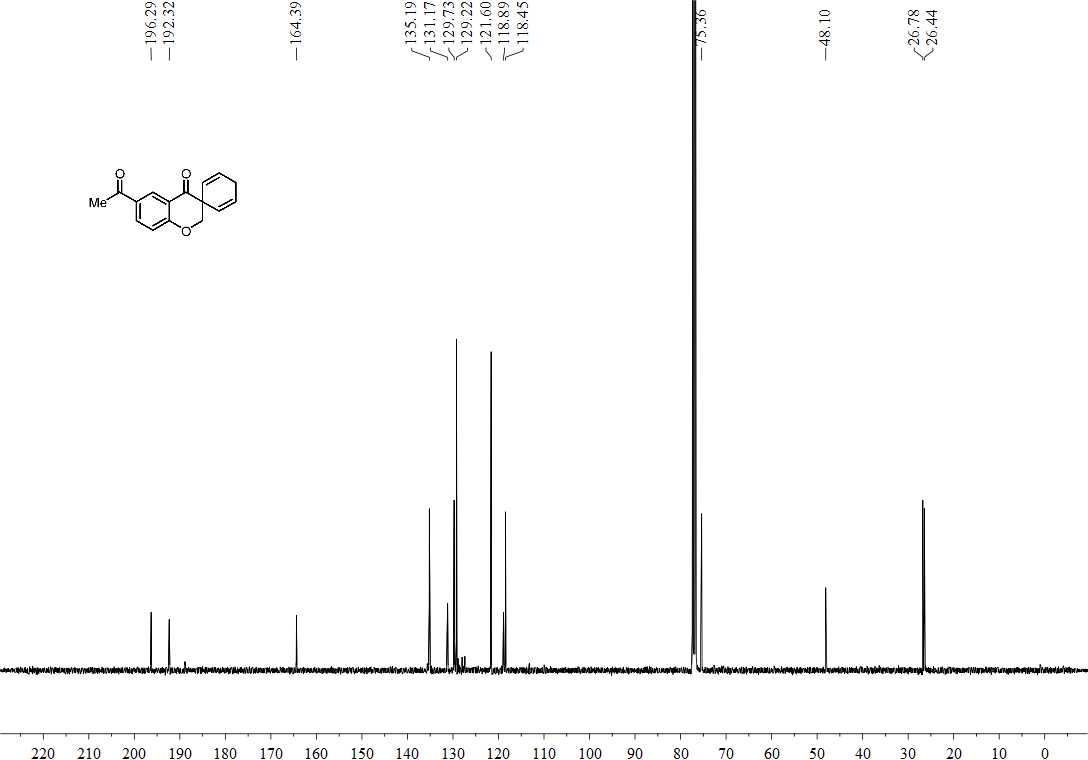


**Supplementary Figure 62.** ^13^C NMR spectra of 6-acetylspiro[chromane-3,1'-cyclohexane]-2',5'-dien-4-one (**5j**).

6-Bromo-7-methylspiro[chromane-3,1'-cyclohexane]-2',5'-dien-4-one (5k)

^1^H NMR (400 MHz, CD_3_CN)


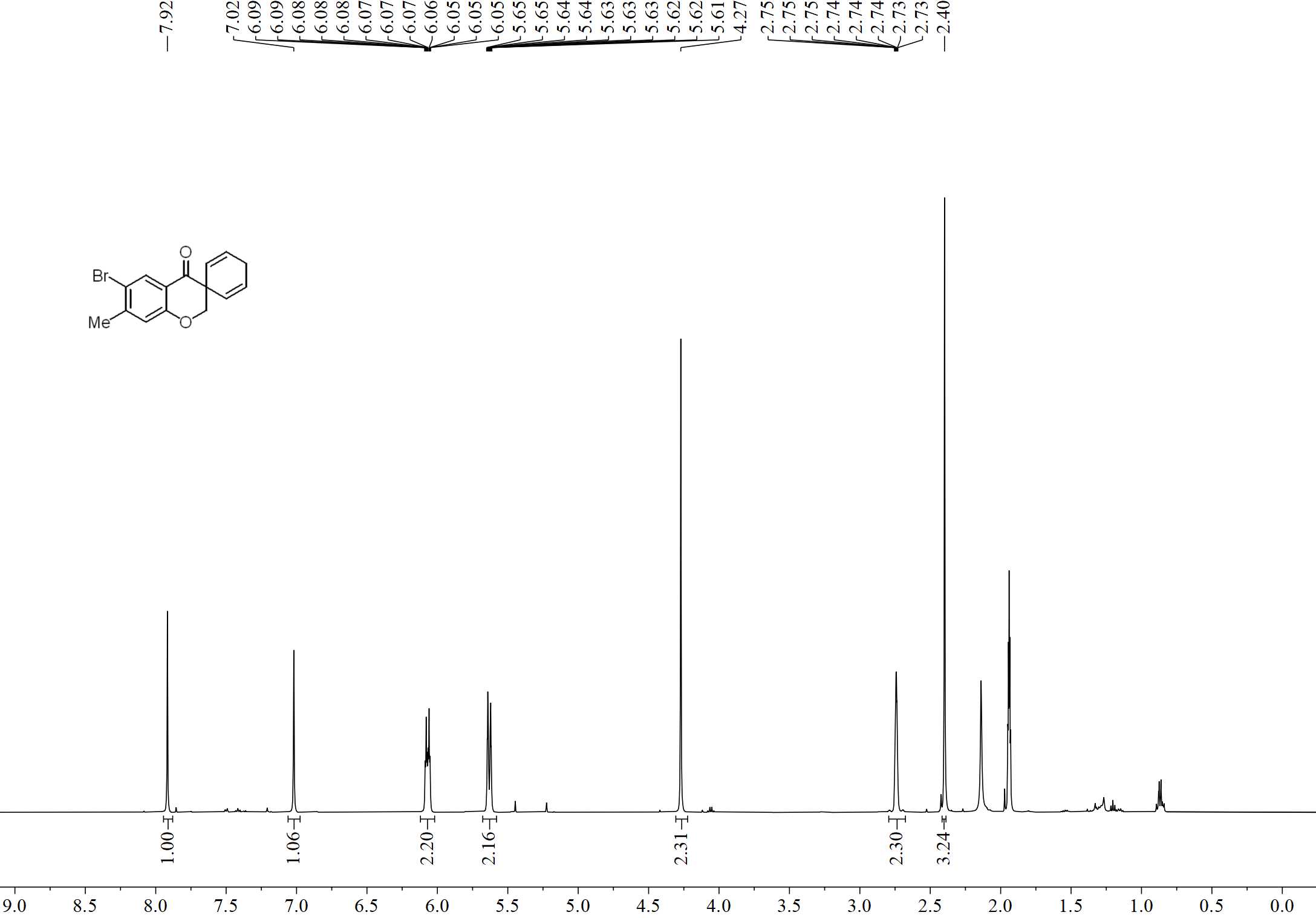


**Supplementary Figure 63.** ^1^H NMR spectra of 6-bromo-7-methylspiro[chromane-3,1'-cyclohexane]-2',5'-dien-4-one (**5k**).

.

^13^C NMR (126 MHz, CD_3_CN)


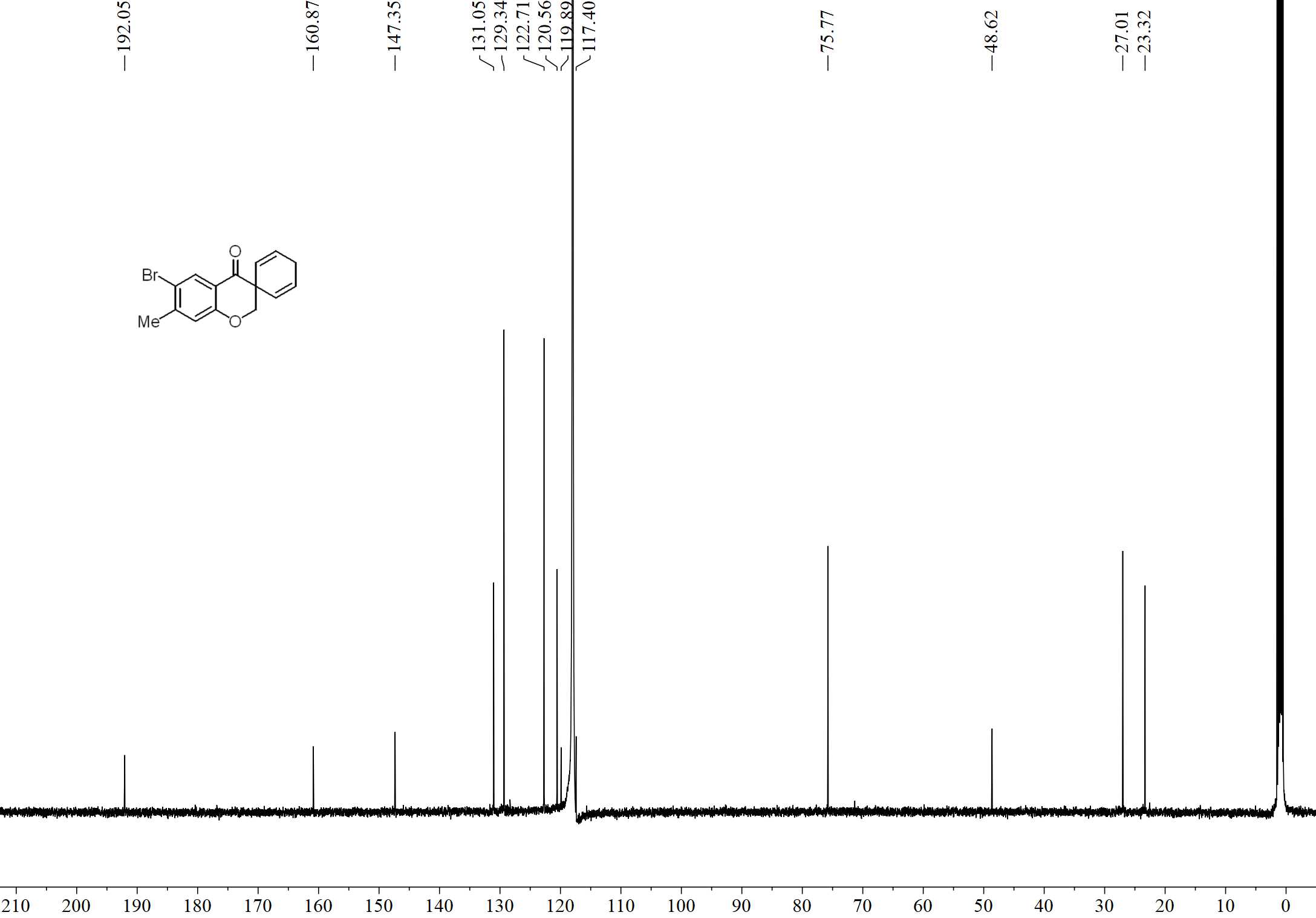


**Supplementary Figure 64.** ^13^C NMR spectra of 6-bromo-7-methylspiro[chromane-3,1'-cyclohexane]-2',5'-dien-4-one (**5k**).

2'-Fluoro-7-methoxyspiro[chromane-3,1'-cyclohexane]-2',5'-dien-4-one (5l)

^1^H NMR (400 MHz, CDCl_3_)


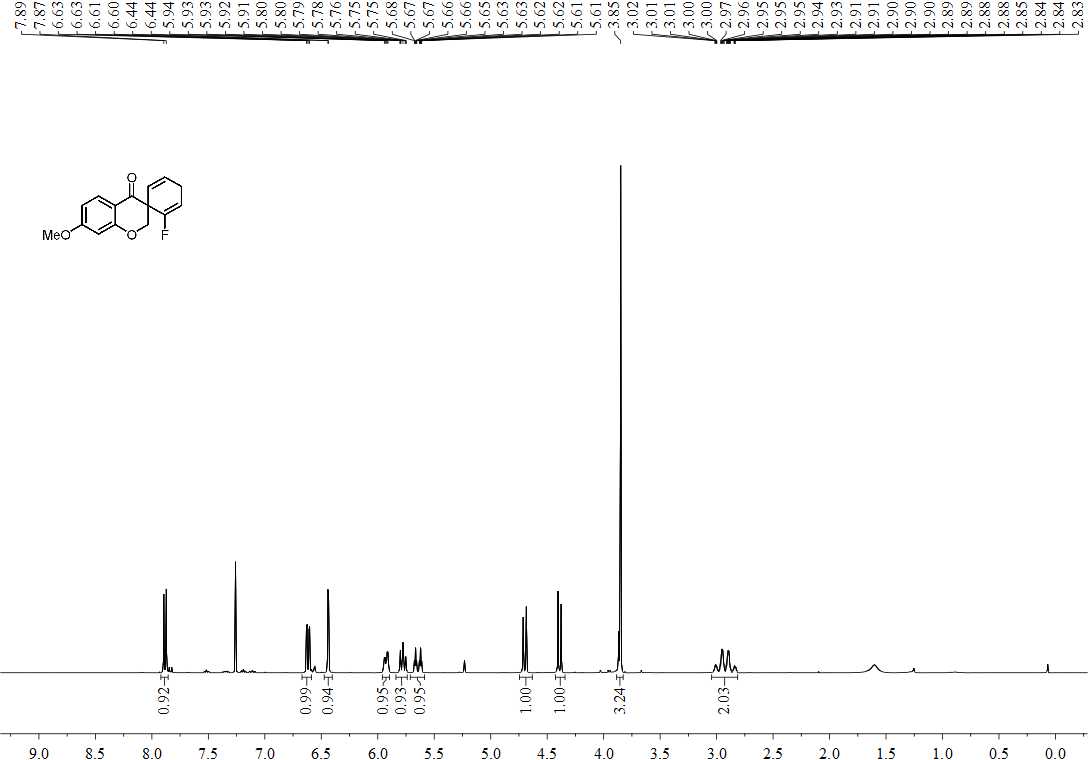


**Supplementary Figure 65.** ^1^H NMR spectra of 2'-fluoro-7-methoxyspiro[chromane-3,1'-cyclohexane]-2',5'-dien-4-one (**5l**).

^13^C NMR (101 MHz, CDCl_3_)


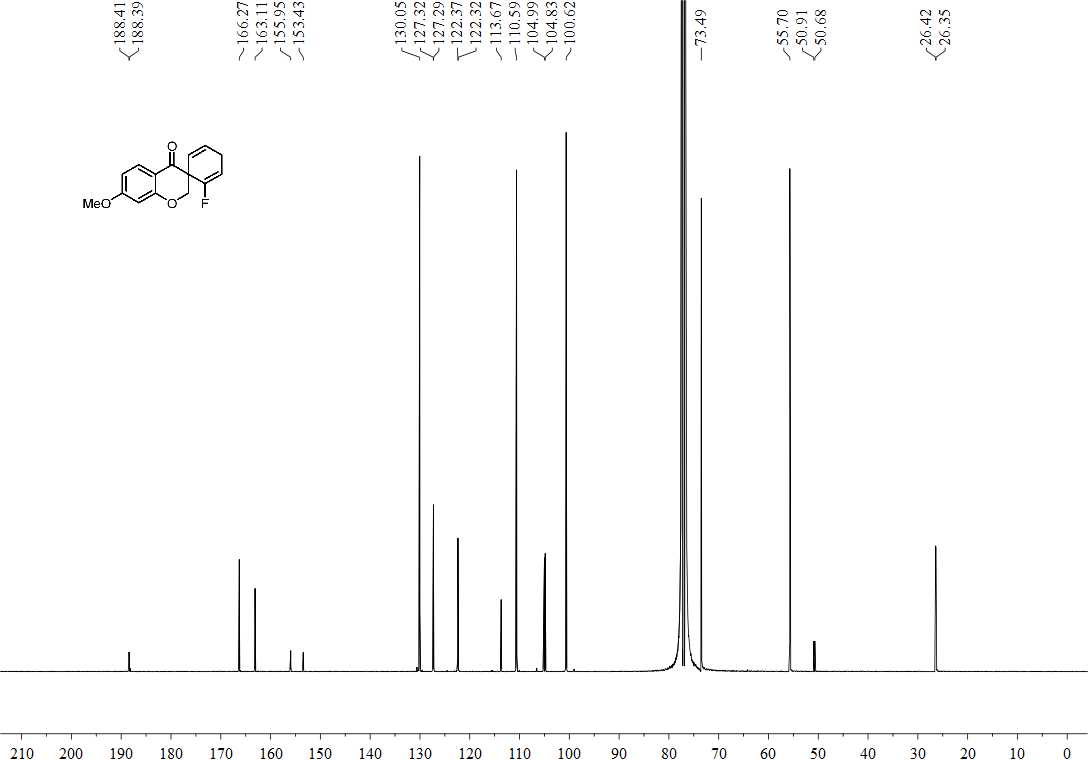


**Supplementary Figure 66.** ^13^C NMR spectra of 2'-fluoro-7-methoxyspiro[chromane-3,1'-cyclohexane]-2',5'-dien-4-one (**5l**).

^19^F NMR (377 MHz, CD_3_CN)


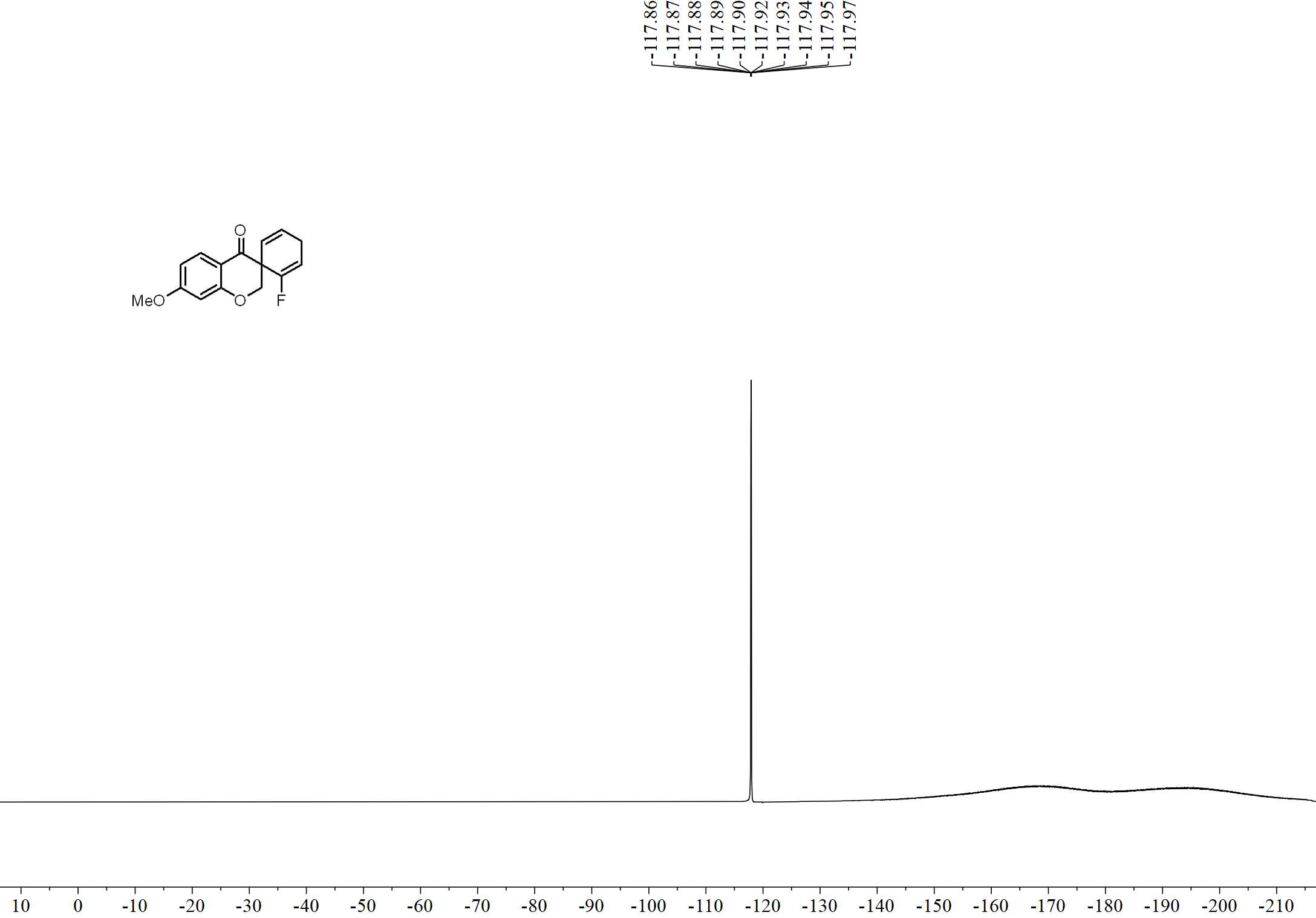


**Supplementary Figure 67.** ^19^F NMR spectra of 2'-fluoro-7-methoxyspiro[chromane-3,1'-cyclohexane]-2',5'-dien-4-one (**5l**).

3'-Fluoro-7-methoxyspiro[chromane-3,1'-cyclohexane]-2',5'-dien-4-one (5m)

^1^H NMR (400 MHz, CDCl_3_)


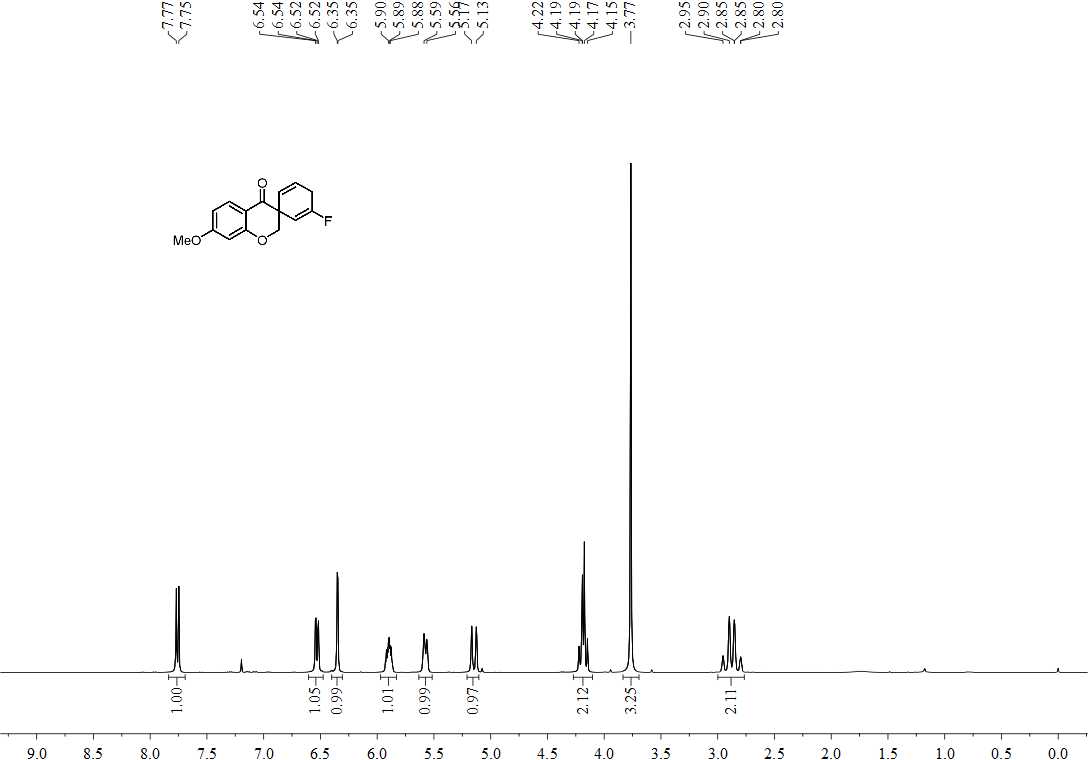


**Supplementary Figure 68.** ^1^H NMR spectra of 3'-fluoro-7-methoxyspiro[chromane-3,1'-cyclohexane]-2',5'-dien-4-one (**5m**).

^13^C NMR (101 MHz, CDCl_3_)


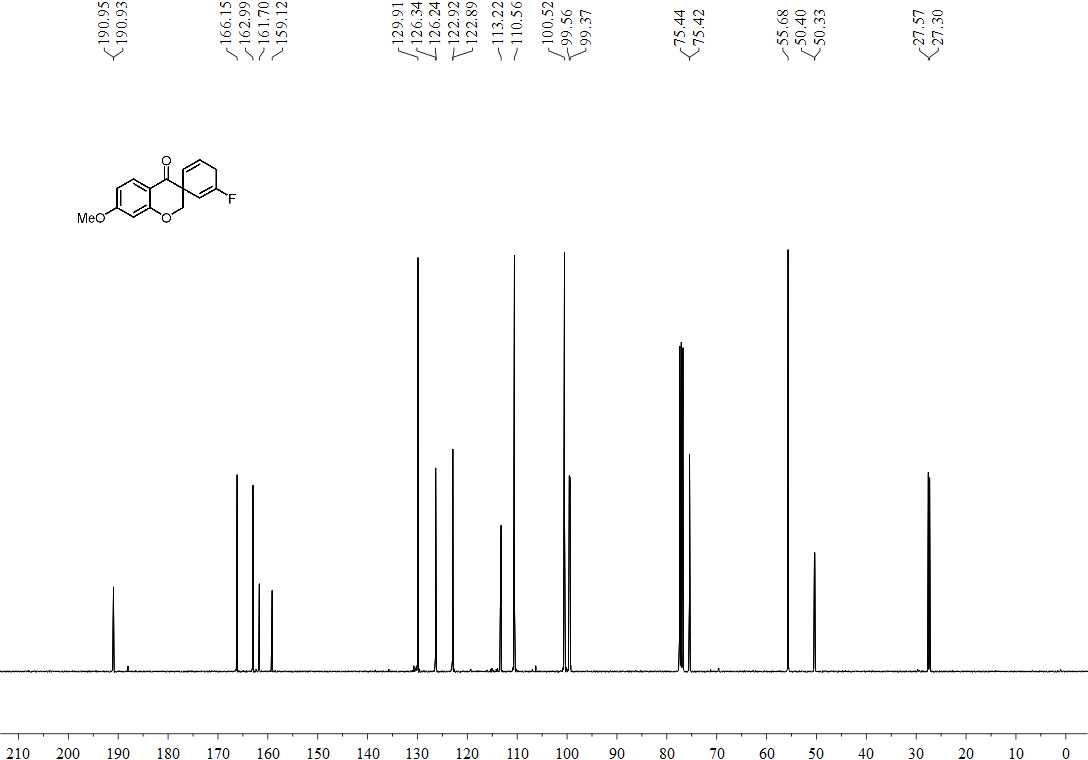


**Supplementary Figure 69.** ^13^C NMR spectra of 3'-fluoro-7-methoxyspiro[chromane-3,1'-cyclohexane]-2',5'-dien-4-one (**5m**).

^19^F NMR (377 MHz, CDCl_3_)


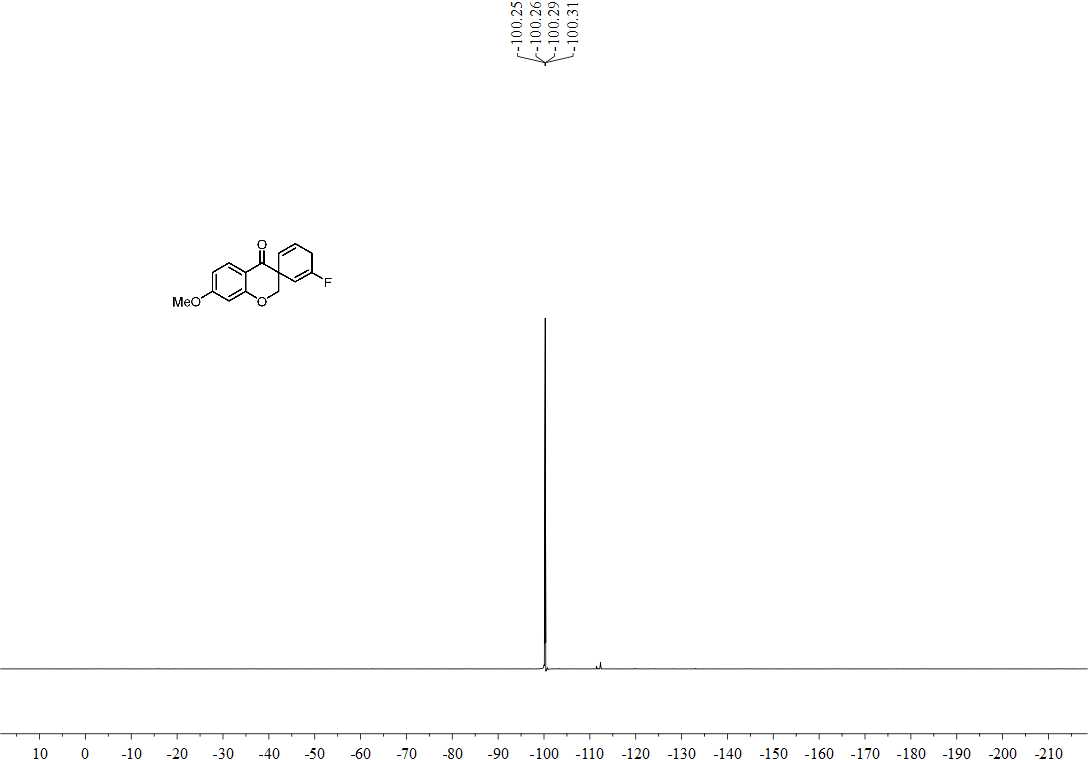


**Supplementary Figure 70.** ^19^F NMR spectra of 3'-fluoro-7-methoxyspiro[chromane-3,1'-cyclohexane]-2',5'-dien-4-one (**5m**).

3'-Chloro-7-methoxyspiro[chromane-3,1'-cyclohexane]-2',5'-dien-4-one (5n)

^1^H NMR (400 MHz, CDCl_3_)


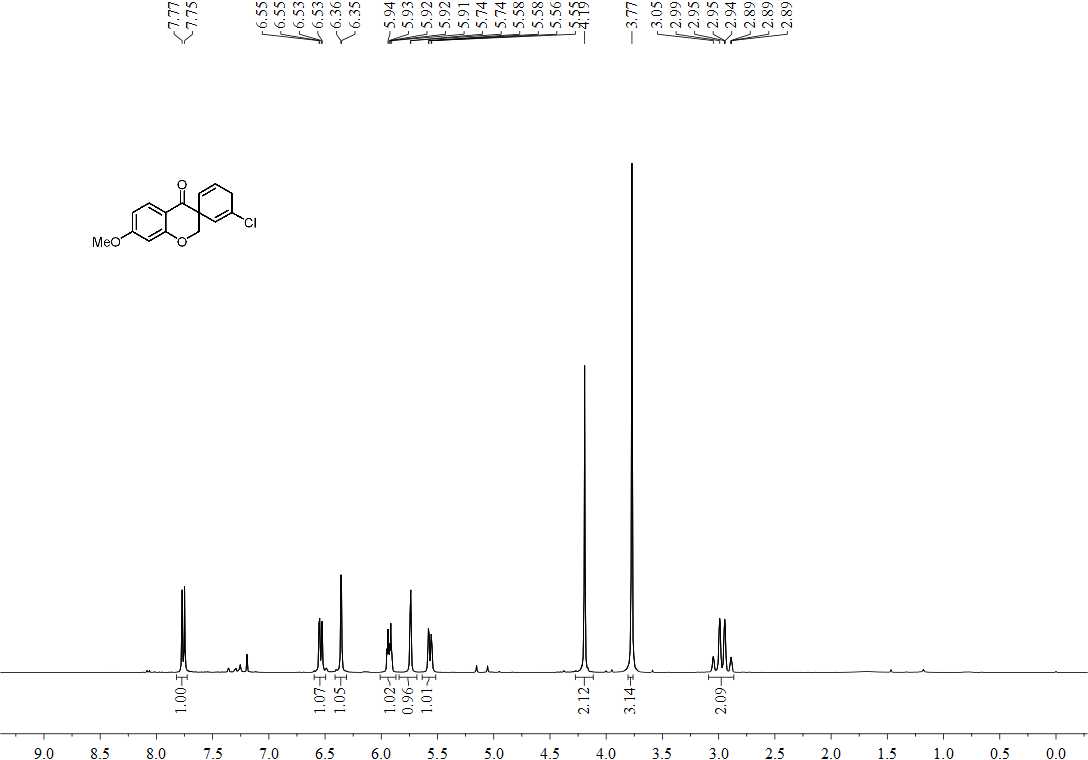


**Supplementary Figure 71.** ^1^H NMR spectra of 3'-chloro-7-methoxyspiro[chromane-3,1'-cyclohexane]-2',5'-dien-4-one (**5n**).

^13^C NMR (101 MHz, CDCl_3_)


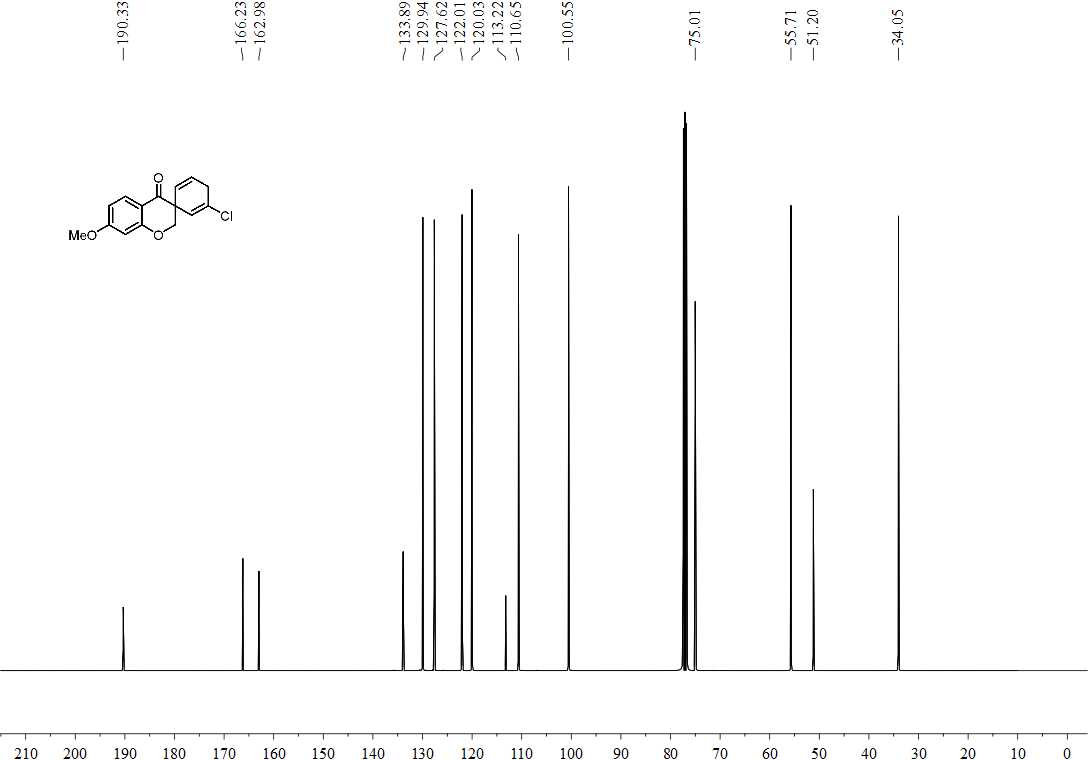


**Supplementary Figure 72.** ^13^C NMR spectra of 3'-chloro-7-methoxyspiro[chromane-3,1'-cyclohexane]-2',5'-dien-4-one (**5n**).

7-Methoxy-3'-(trifluoromethyl)spiro[chromane-3,1'-cyclohexane]-2',5'-dien-4-one (5o)

^1^H NMR (400 MHz, CDCl_3_)


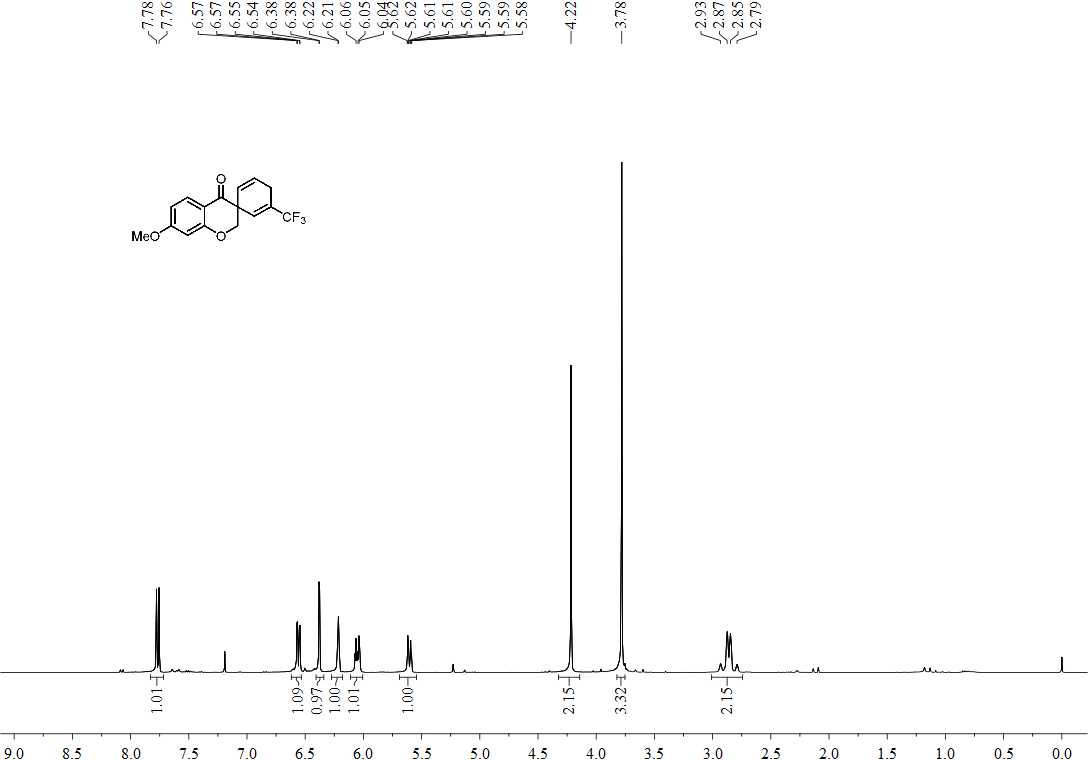


**Supplementary Figure 73.** ^1^H NMR spectra of 7-methoxy-3'-(trifluoromethyl)spiro[chromane-3,1'-cyclohexane]-2',5'-dien-4-one (**5o**).

^13^C NMR (101 MHz, CDCl_3_)


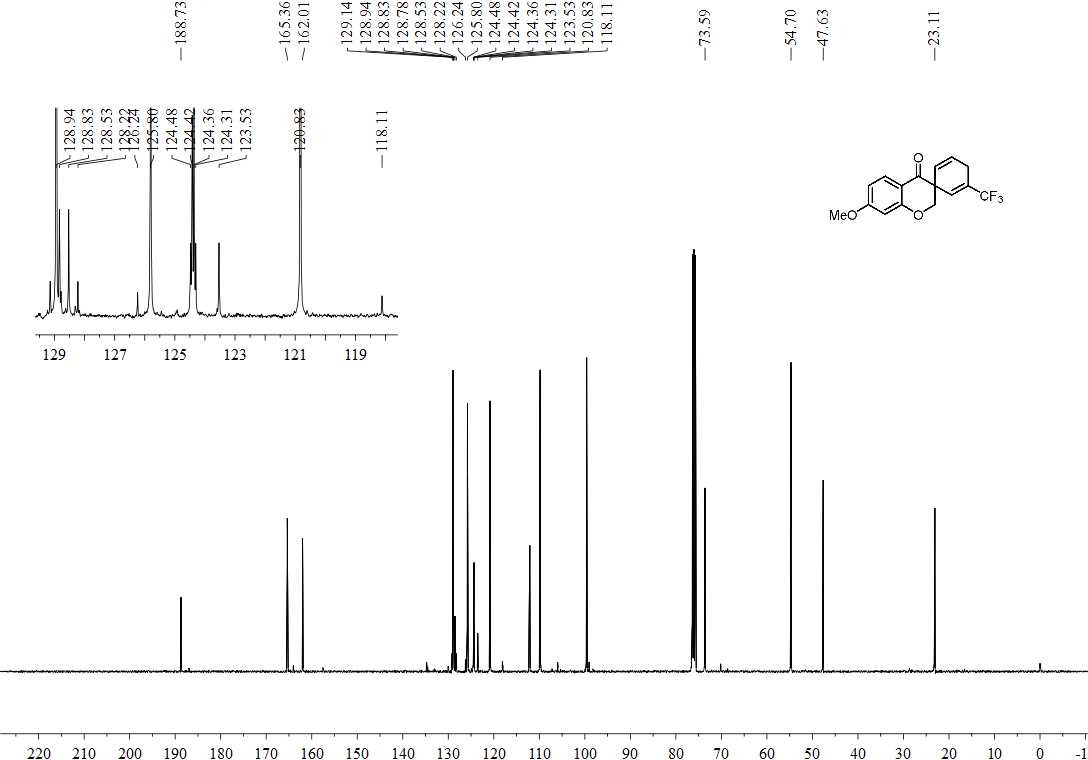


**Supplementary Figure 74.** ^13^C NMR spectra of 7-methoxy-3'-(trifluoromethyl)spiro[chromane-3,1'-cyclohexane]-2',5'-dien-4-one (**5o**)

^19^F NMR (377 MHz, CDCl_3_)


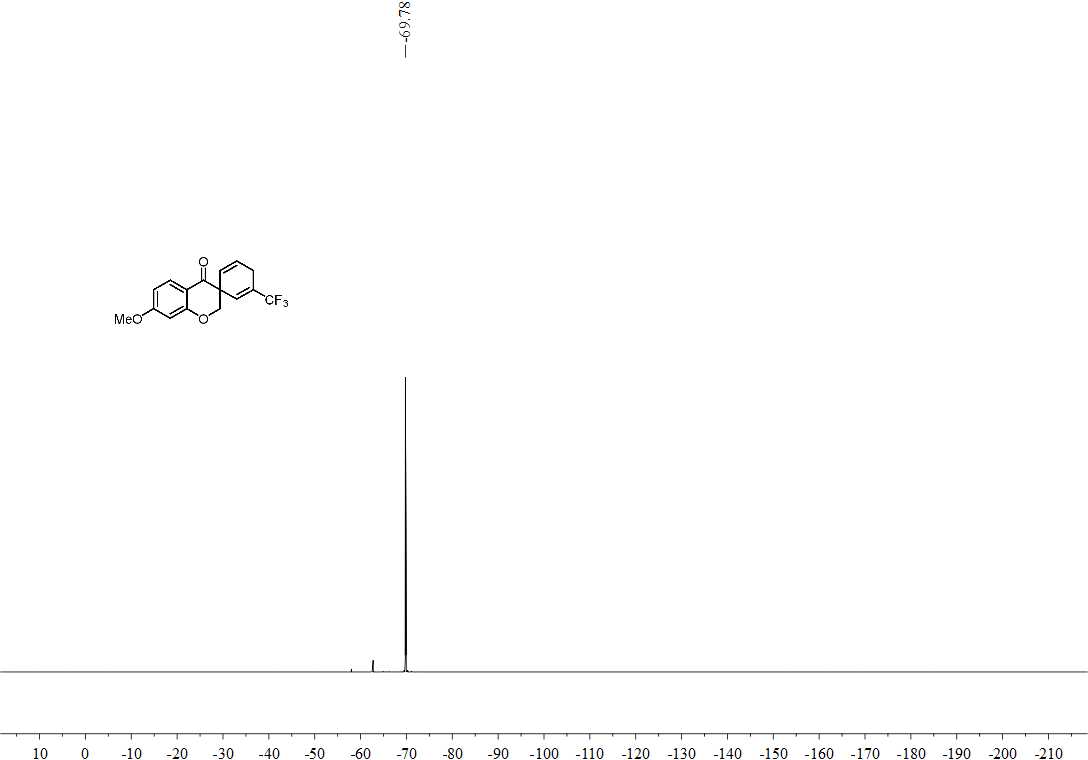


**Supplementary Figure 75.** ^19^F NMR spectra of 7-methoxy-3'-(trifluoromethyl)spiro[chromane-3,1'-cyclohexane]-2',5'-dien-4-one (**5o**)

7-Methoxy-3'-methylspiro[chromane-3,1'-cyclohexane]-2',5'-dien-4-one (5p)

^1^H NMR (400 MHz, CDCl_3_)


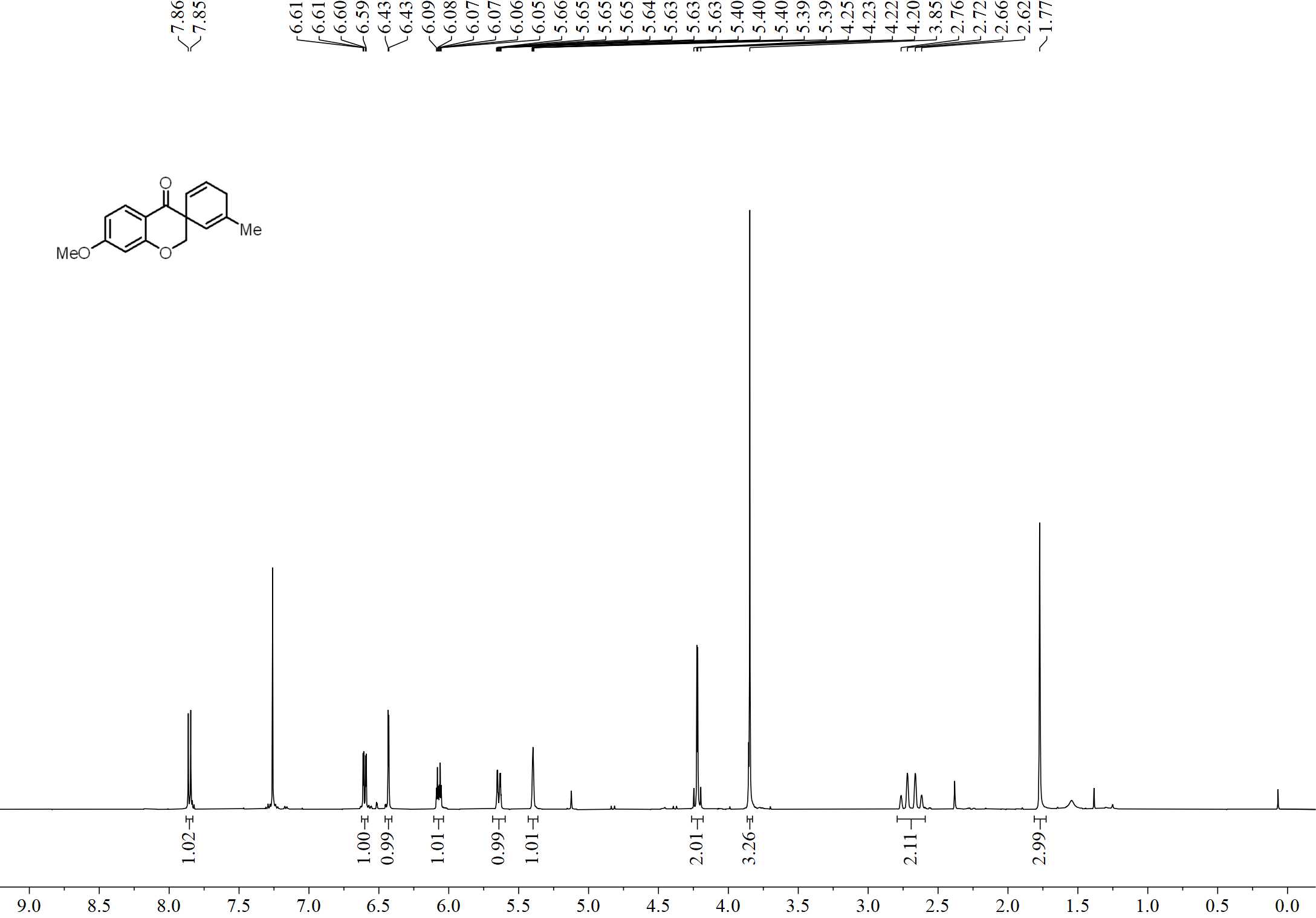


**Supplementary Figure 76.** ^1^H NMR spectra of 7-methoxy-3'-methylspiro[chromane-3,1'-cyclohexane]-2',5'-dien-4-one (**5p**).

^13^C NMR (101 MHz, CDCl_3_)


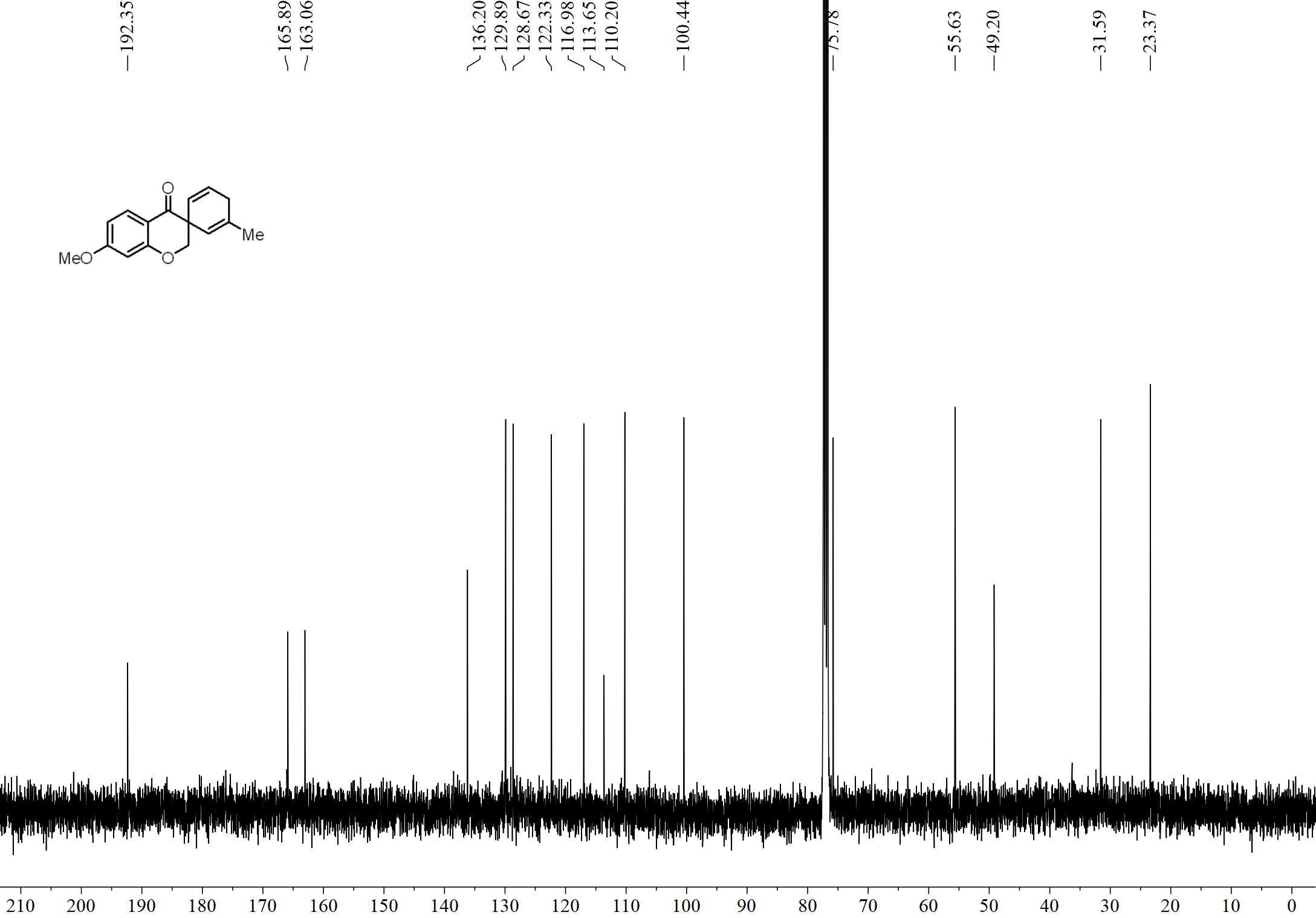


**Supplementary Figure 77.** ^13^C NMR spectra of 7-methoxy-3'-methylspiro[chromane-3,1'-cyclohexane]-2',5'-dien-4-one (**5p**).

3',7-Dimethoxyspiro[chromane-3,1'-cyclohexane]-2',5'-dien-4-one (5q)

^1^H NMR (400 MHz, CD_3_CN)


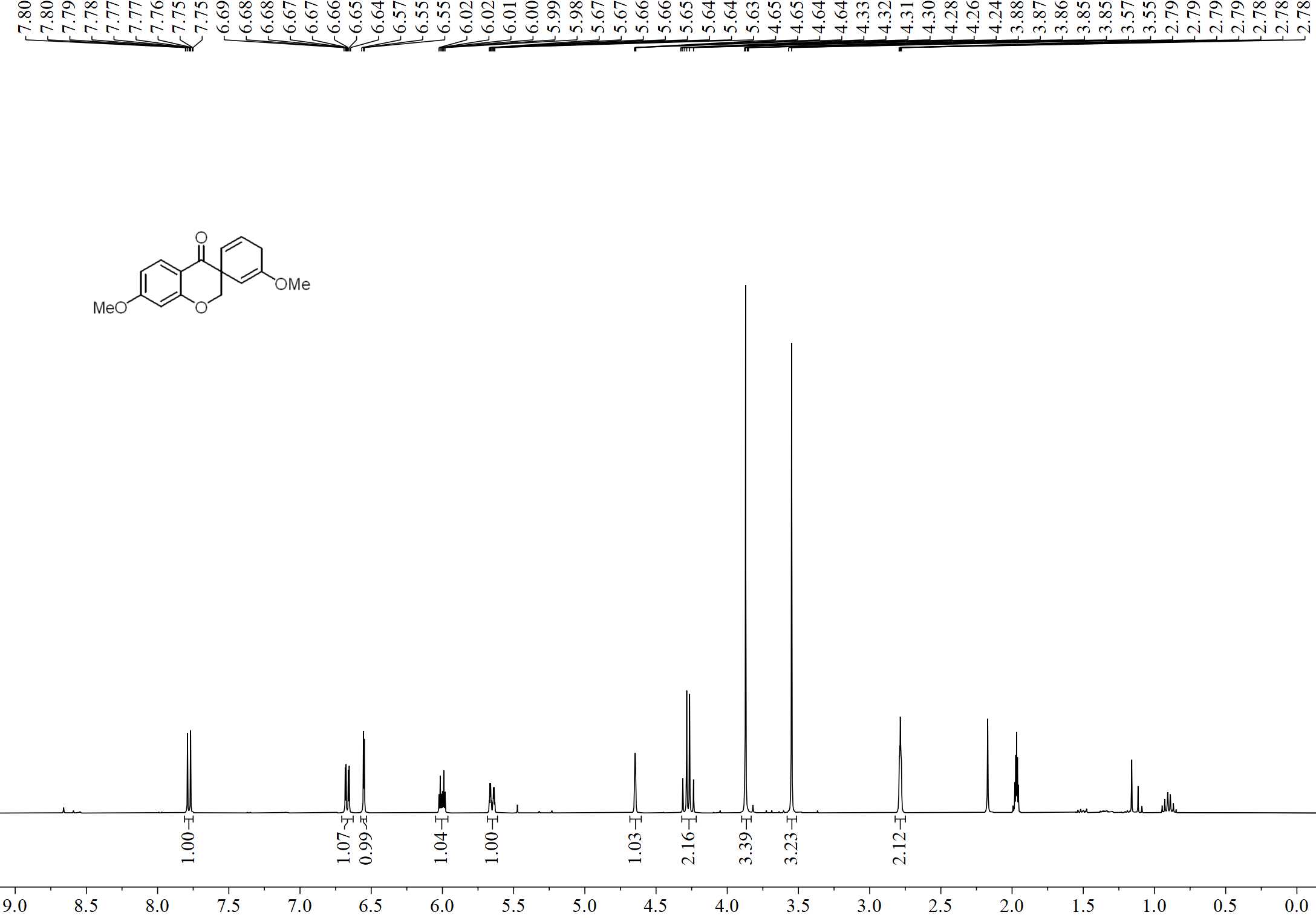


**Supplementary Figure 78.** ^1^H NMR spectra of 3',7-dimethoxyspiro[chromane-3,1'-cyclohexane]-2',5'-dien-4-one (**5q**).

^13^C NMR (101 MHz, CD_3_CN)


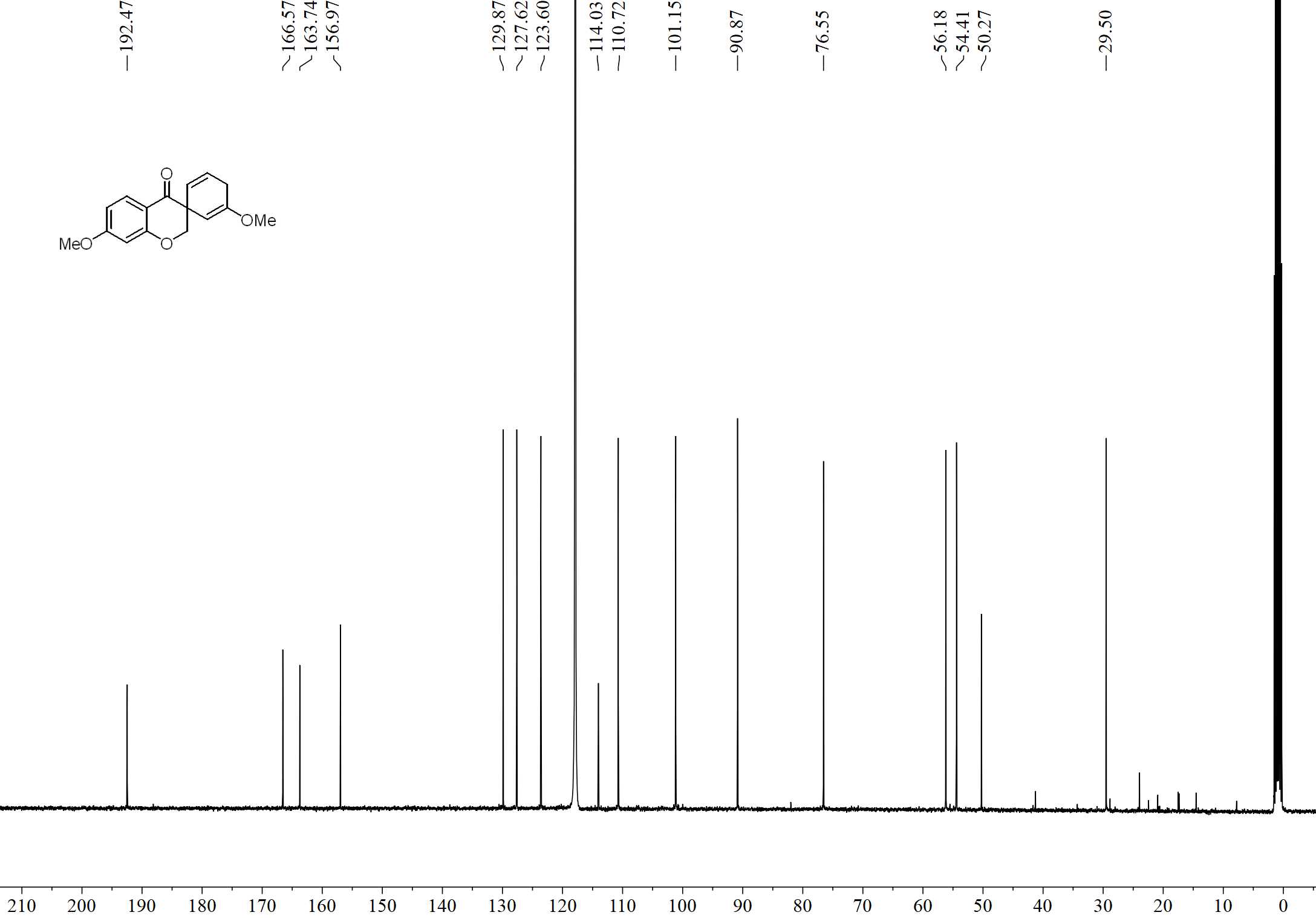


**Supplementary Figure 79.** ^13^C NMR spectra of 3',7-dimethoxyspiro[chromane-3,1'-cyclohexane]-2',5'-dien-4-one (**5q**).

7-Methoxy-3',5'-dimethylspiro[chromane-3,1'-cyclohexane]-2',5'-dien-4-one (5r)

^1^H NMR (400 MHz, CDCl_3_)


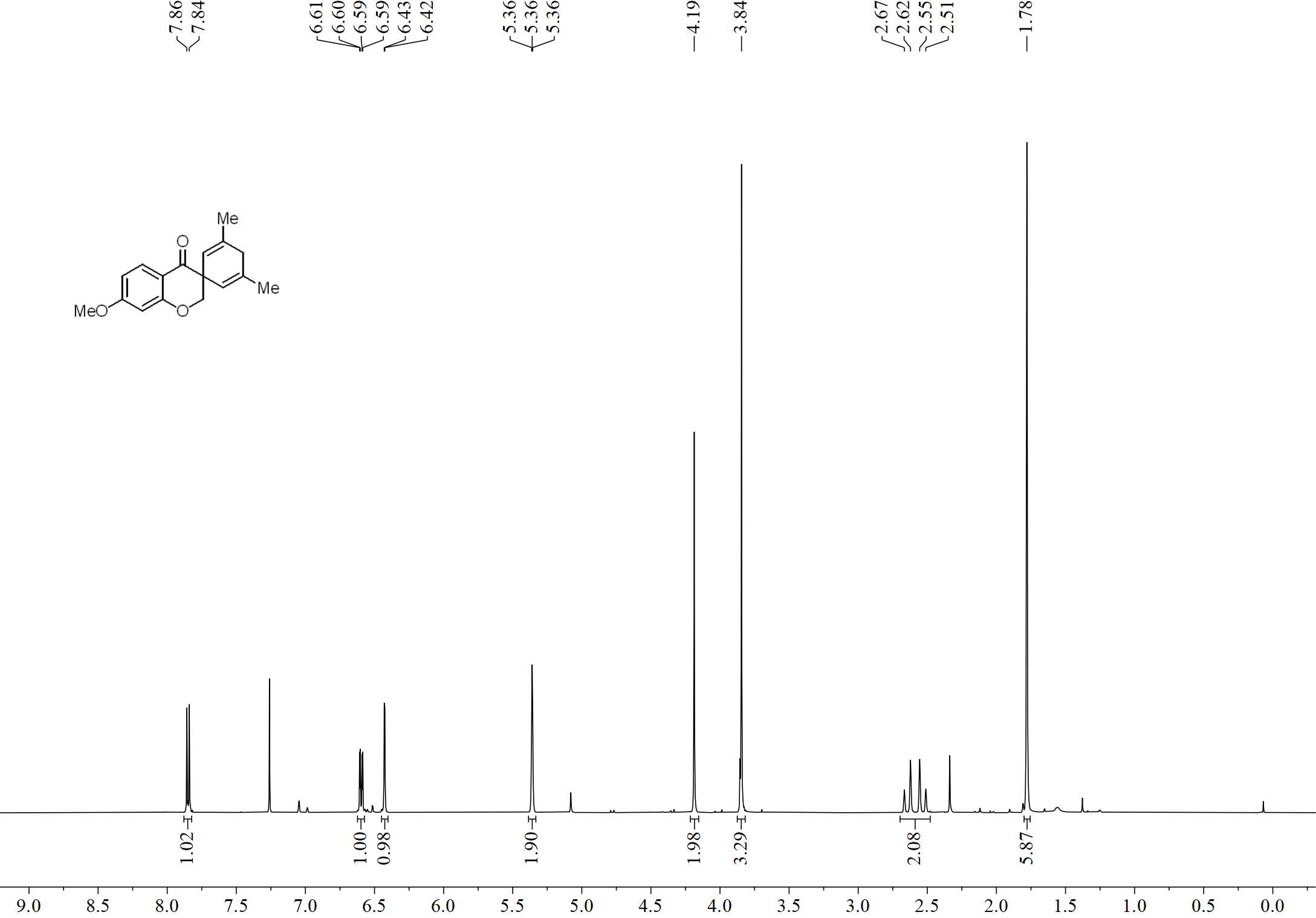


**Supplementary Figure 80.** ^1^H NMR spectra of 7-methoxy-3',5'-dimethylspiro[chromane-3,1'-cyclohexane]-2',5'-dien-4-one (**5r**).

^13^C NMR (101 MHz, CDCl_3_)


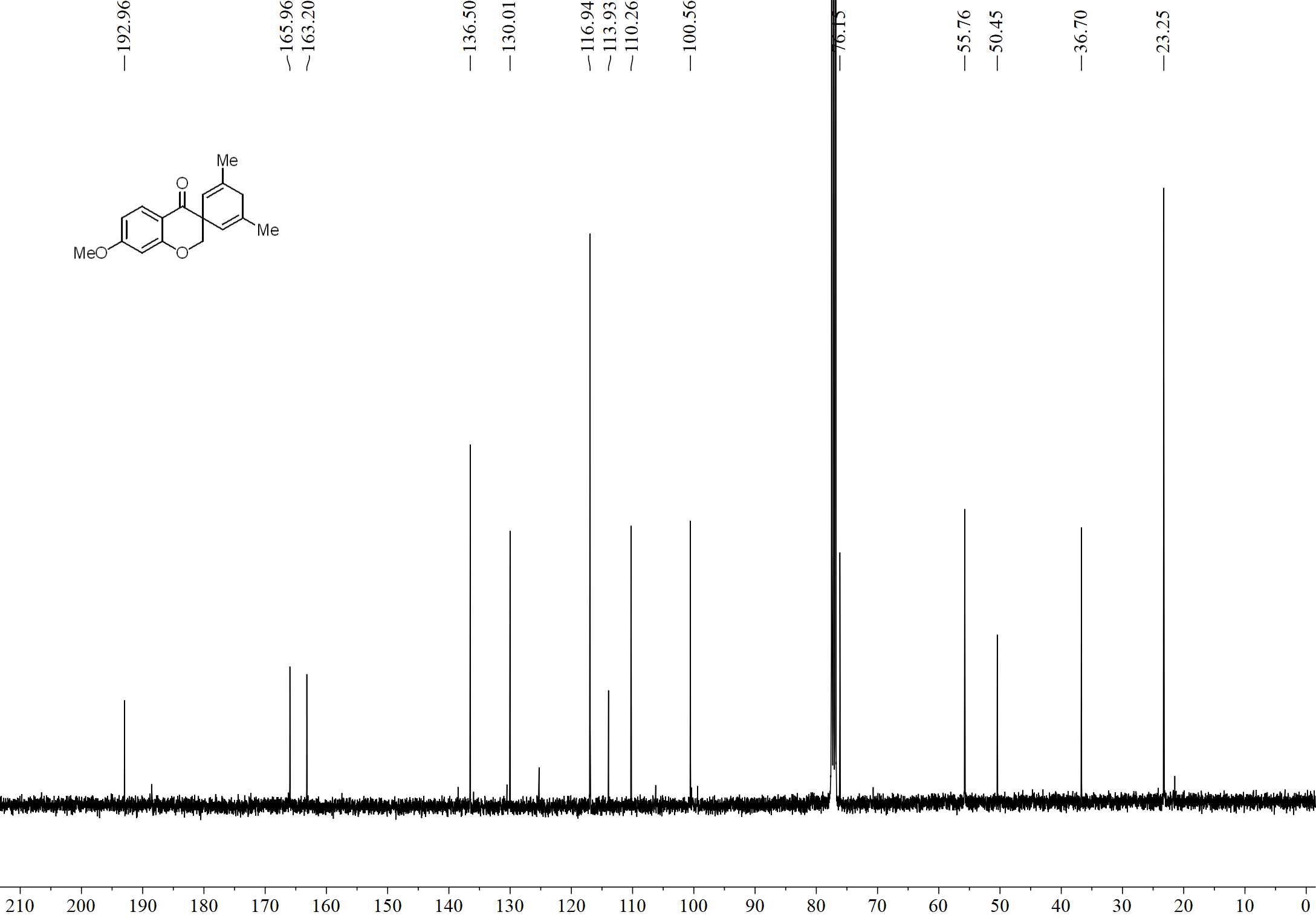


**Supplementary Figure 81.** ^13^C NMR spectra of 7-methoxy-3',5'-dimethylspiro[chromane-3,1'-cyclohexane]-2',5'-dien-4-one (**5r**).

3',5',7-Trimethoxyspiro[chromane-3,1'-cyclohexane]-2',5'-dien-4-one (5s)

^1^H NMR (400 MHz, CD_3_CN)


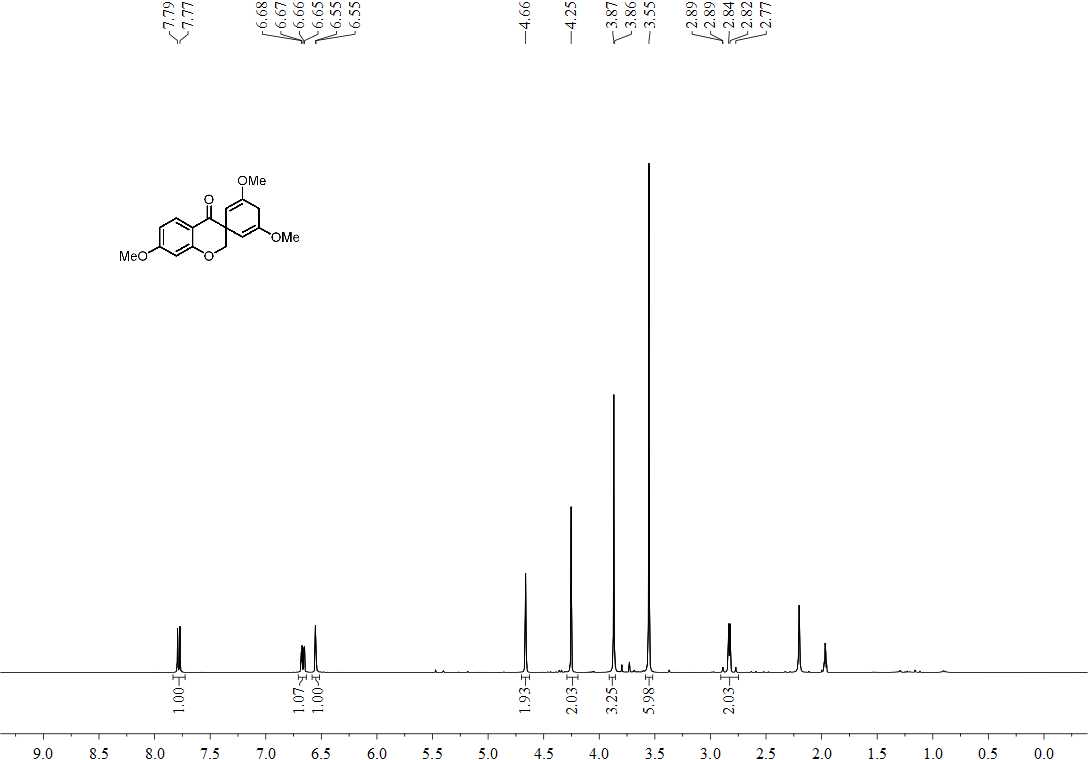


**Supplementary Figure 82.** ^1^H NMR spectra of 3',5',7-trimethoxyspiro[chromane-3,1'-cyclohexane]-2',5'-dien-4-one (**5s**).

^13^C NMR (101 MHz, CD_3_CN)


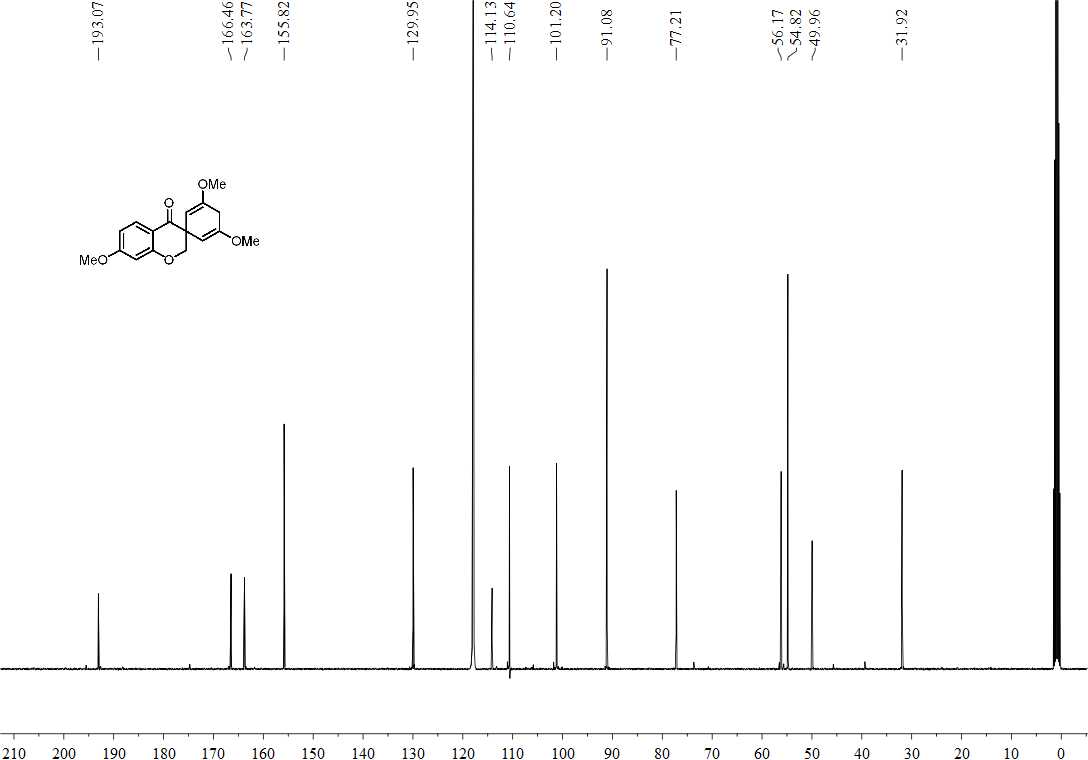


**Supplementary Figure 83.** ^13^C NMR spectra of 3',5',7-trimethoxyspiro[chromane-3,1'-cyclohexane]-2',5'-dien-4-one (**5s**).

7-Methoxy-4'H-spiro[chromane-3,1'-naphthalen]-4-one (5t)

^1^H NMR (400 MHz, CDCl_3_)


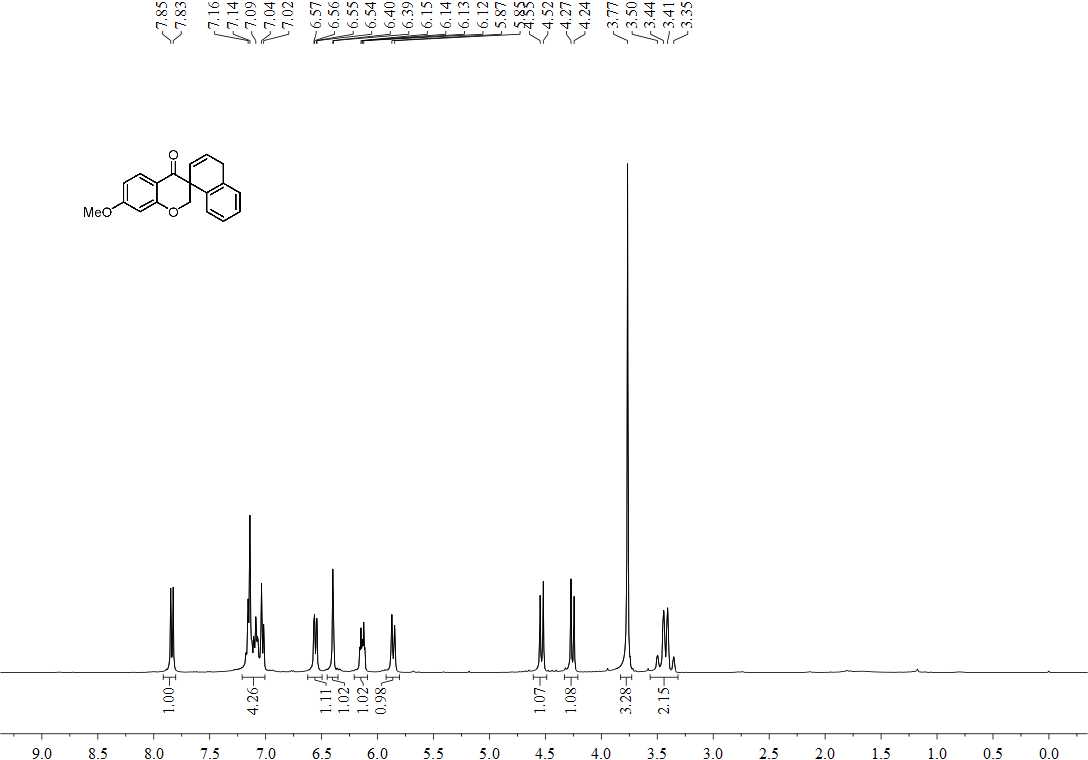


**Supplementary Figure 84.** ^1^H NMR spectra of 7-methoxy-4'H-spiro[chromane-3,1'-naphthalen]-4-one (**5t**).

^13^C NMR (101 MHz, CDCl_3_)


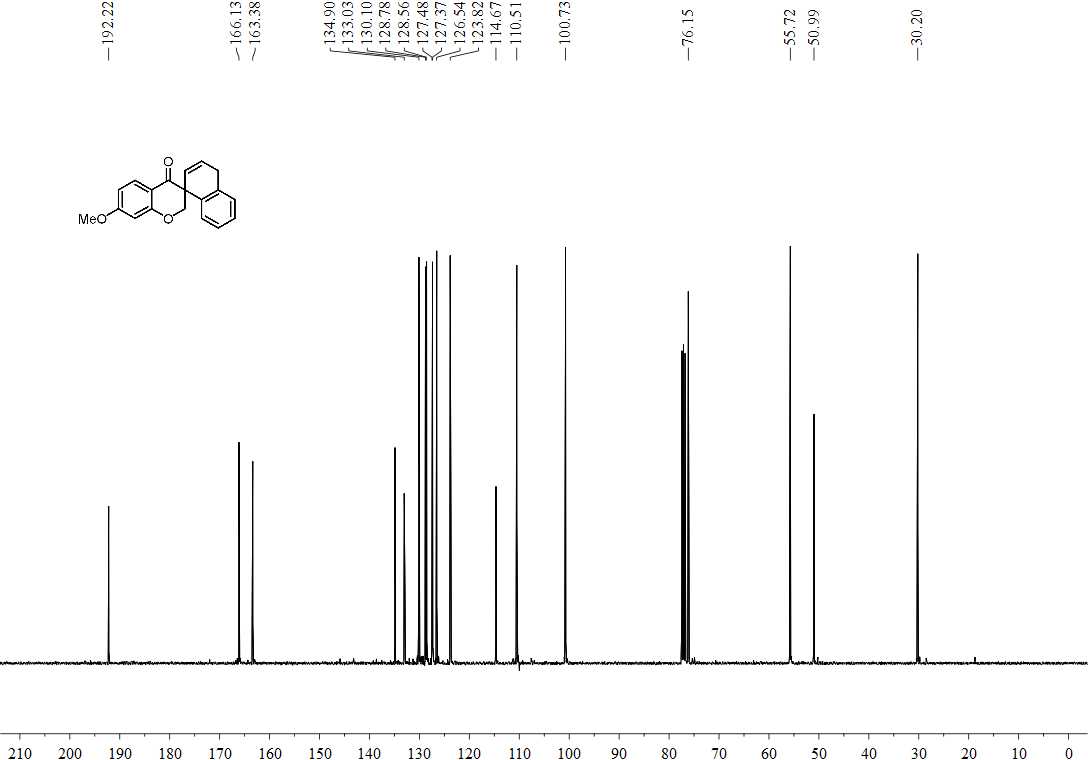


**Supplementary Figure 85.** ^13^C NMR spectra of 7-methoxy-4'H-spiro[chromane-3,1'-naphthalen]-4-one (**5t**).

tert-Butyl 4'-oxo-2'H-spiro[cyclohexane-1,3'-quinoline]-2,5-diene-1'(4'H)-carboxylate (5u)

^1^H NMR (400 MHz, CDCl_3_)


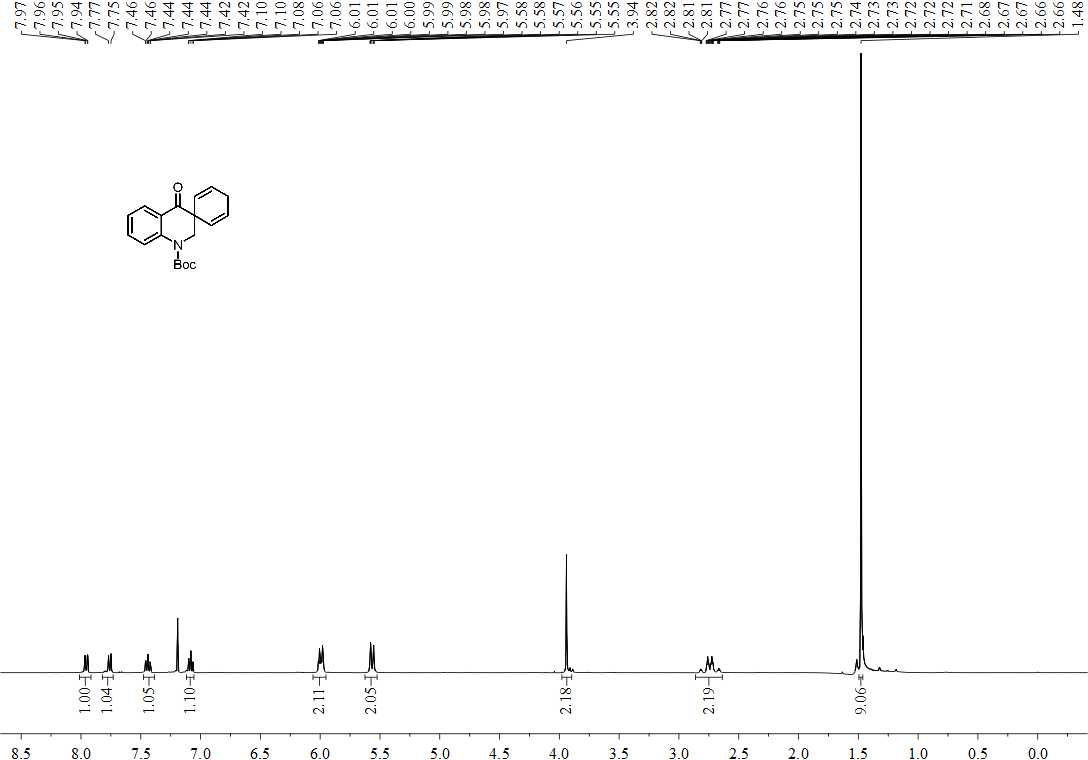


**Supplementary Figure 86.** ^1^H NMR spectra of *tert*-butyl 4'-oxo-2'H-spiro[cyclohexane-1,3'-quinoline]-2,5-diene-1'(4'H)-carboxylate (**5u**).

^13^C NMR (101 MHz, CDCl_3_)


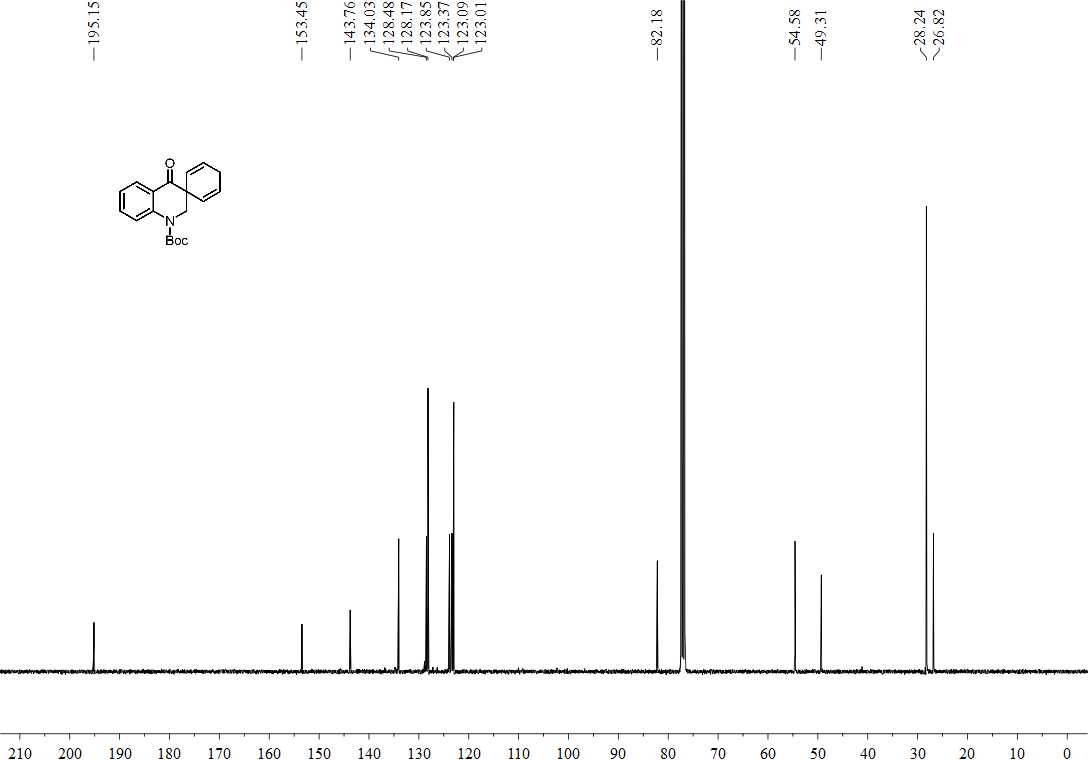


**Supplementary Figure 87.** ^13^C NMR spectra of *tert*-butyl 4'-oxo-2'H-spiro[cyclohexane-1,3'-quinoline]-2,5-diene-1'(4'H)-carboxylate (**5u**).

7-(2,4-Difluorophenyl)spiro[chromane-3,1'-cyclohexane]-2',5'-dien-4-one (5v)

^1^H NMR (400 MHz, CD_3_CN)


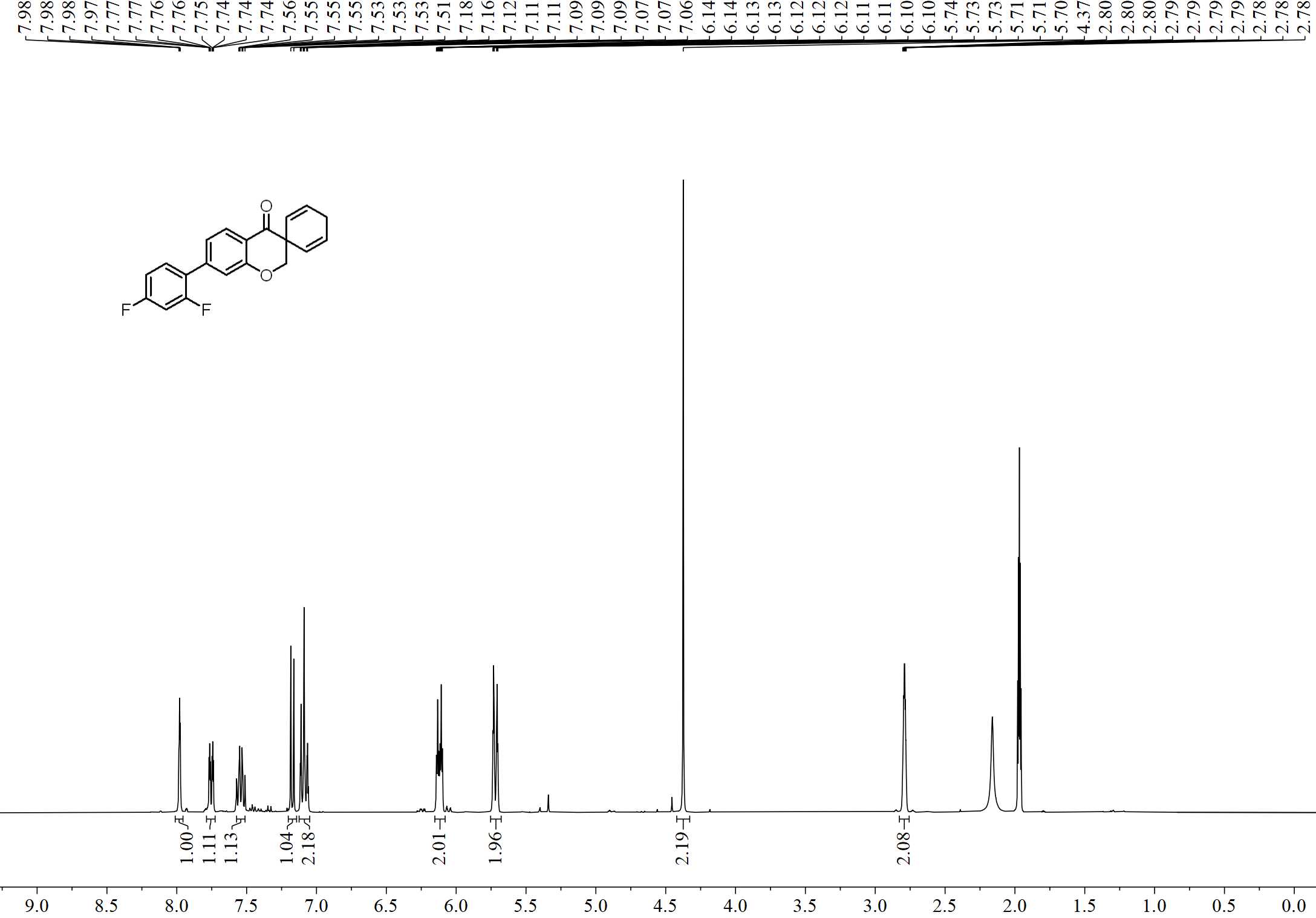


**Supplementary Figure 88.** ^1^H NMR spectra of 7-(2,4-difluorophenyl)spiro[chromane-3,1'-cyclohexane]-2',5'-dien-4-one (**5v**).

^13^C NMR (101 MHz, CD_3_CN)


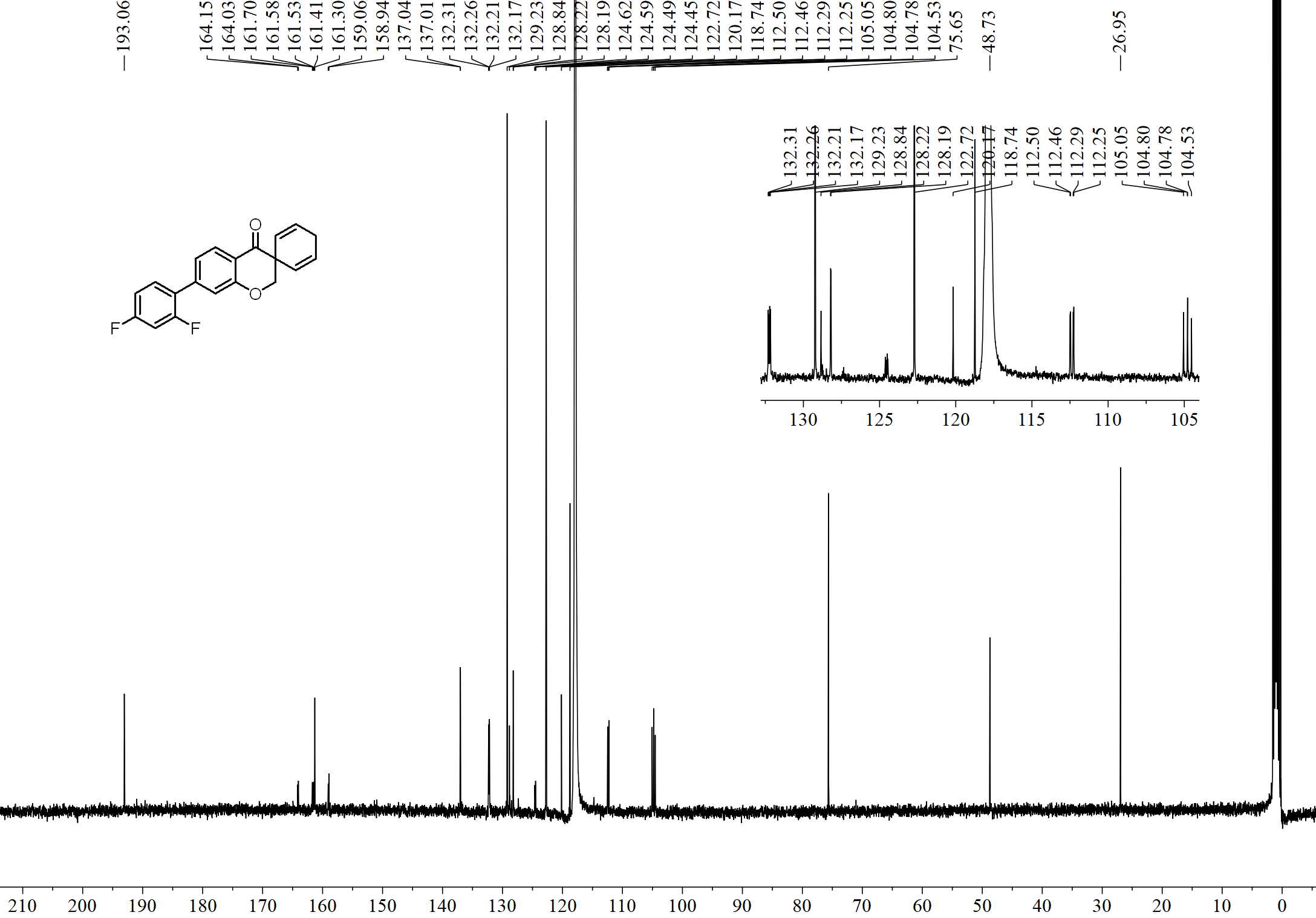


**Supplementary Figure 89.** ^13^C NMR spectra of 7-(2,4-difluorophenyl)spiro[chromane-3,1'-cyclohexane]-2',5'-dien-4-one (**5v**).

^19^F NMR (377 MHz, CD_3_CN)


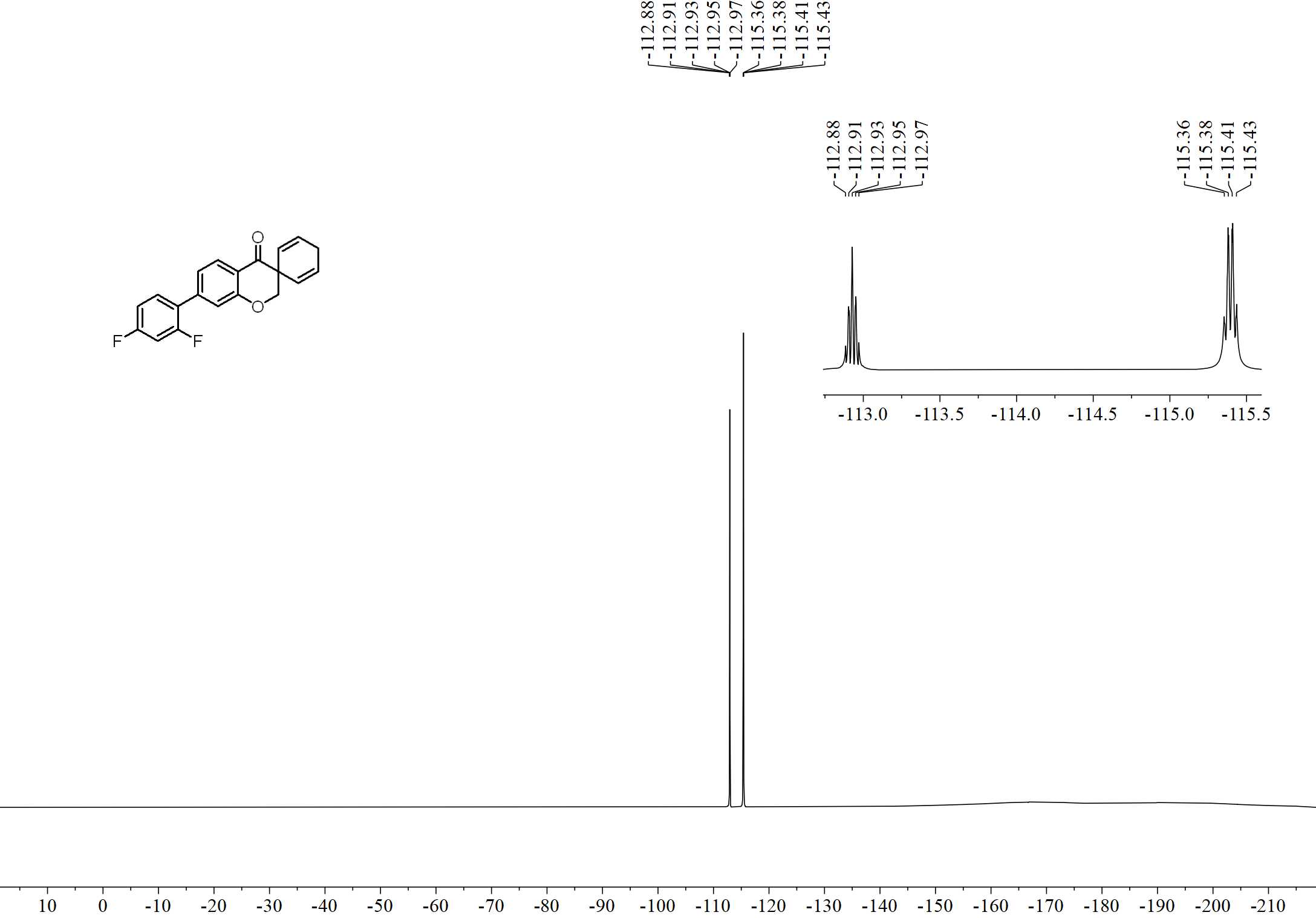


**Supplementary Figure 90.** ^19^F NMR spectra of 7-(2,4-difluorophenyl)spiro[chromane-3,1'-cyclohexane]-2',5'-dien-4-one (**5v**).

(R)-7-Methoxy-2-methylspiro[chromane-3,1'-cyclohexane]-2',5'-dien-4-one (5w)

^1^H NMR (400 MHz, CDCl_3_)


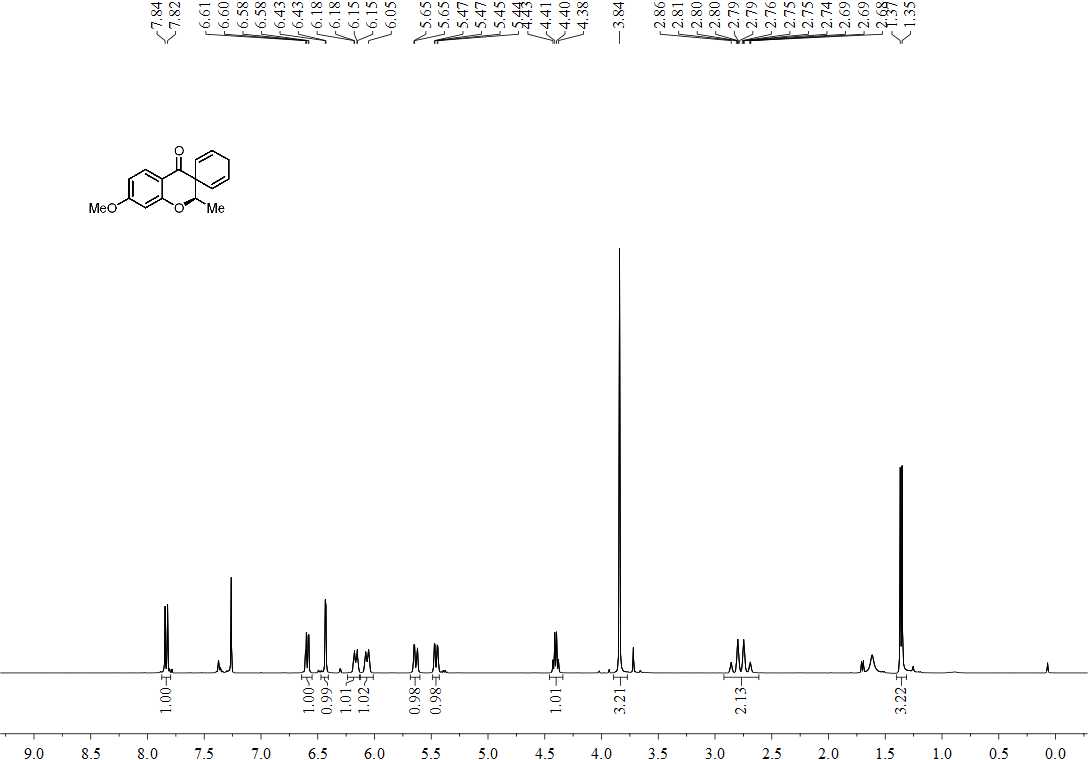


**Supplementary Figure 91.** ^1^H NMR spectra of (*R*)-7-methoxy-2-methylspiro[chromane-3,1'-cyclohexane]-2',5'-dien-4-one (**5w**).

^13^C NMR (101 MHz, CDCl_3_)


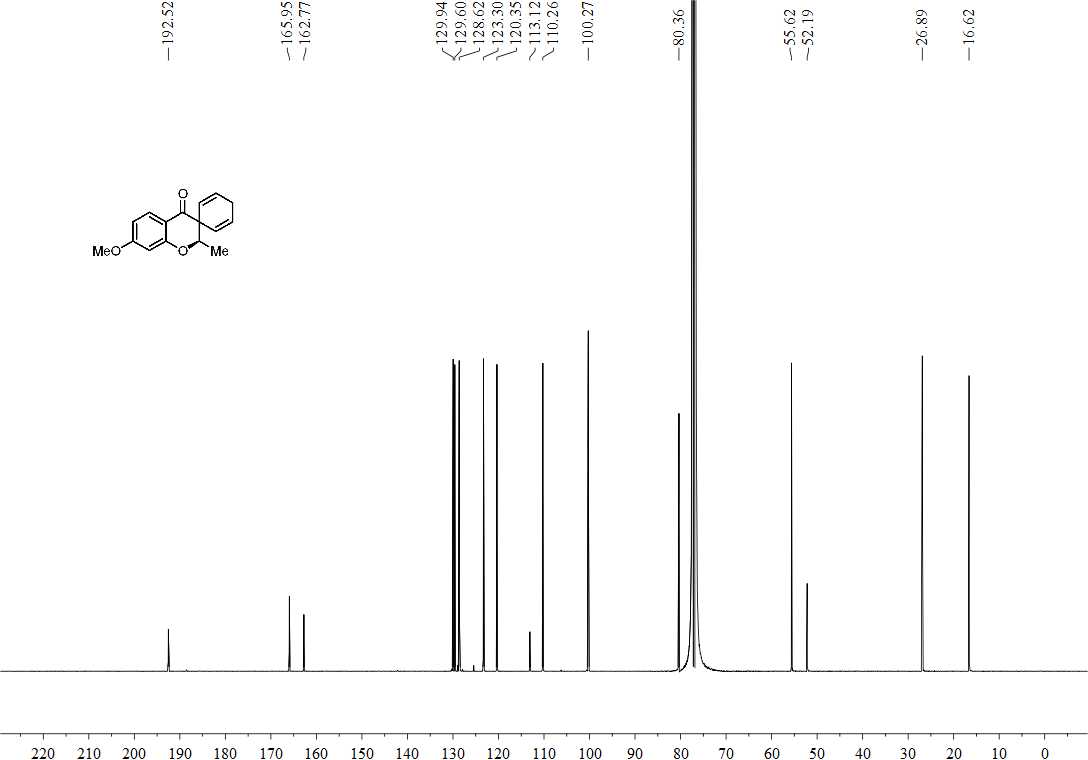


**Supplementary Figure 92.** ^13^C NMR spectra of (*R*)-7-methoxy-2-methylspiro[chromane-3,1'-cyclohexane]-2',5'-dien-4-one (**5w**).


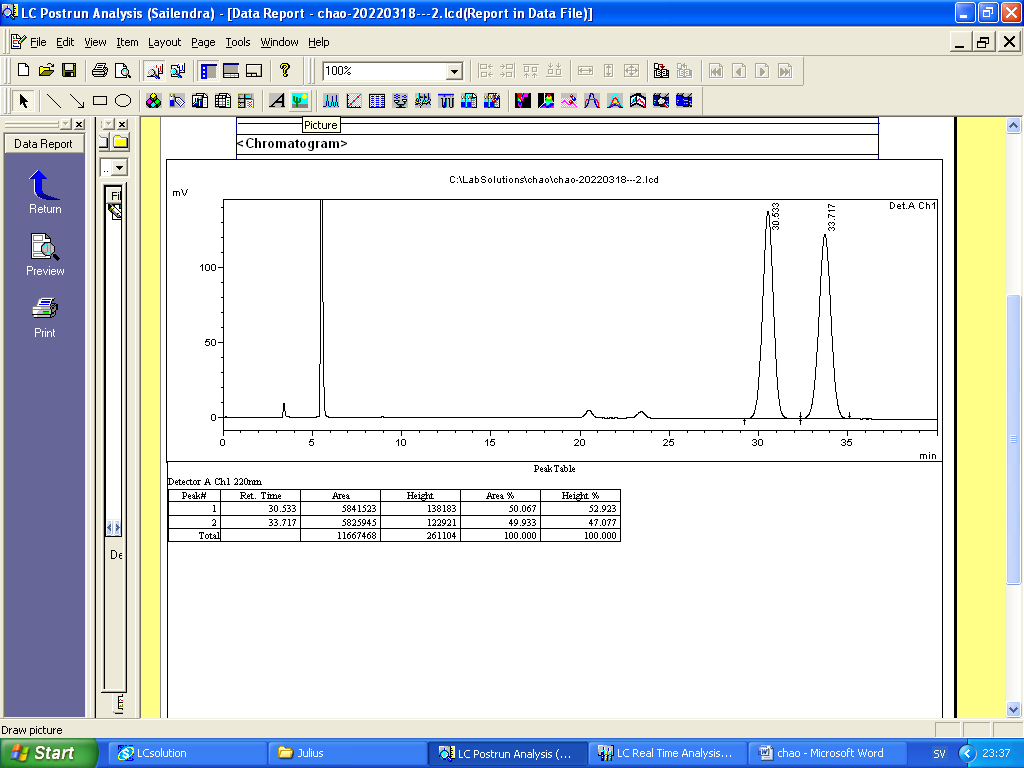


**Supplementary Figure 93.** HPLC chromatogram of *rac*-7-methoxy-2-methylspiro[chromane-3,1'-cyclohexane]-2',5'-dien-4-one (**5w**).


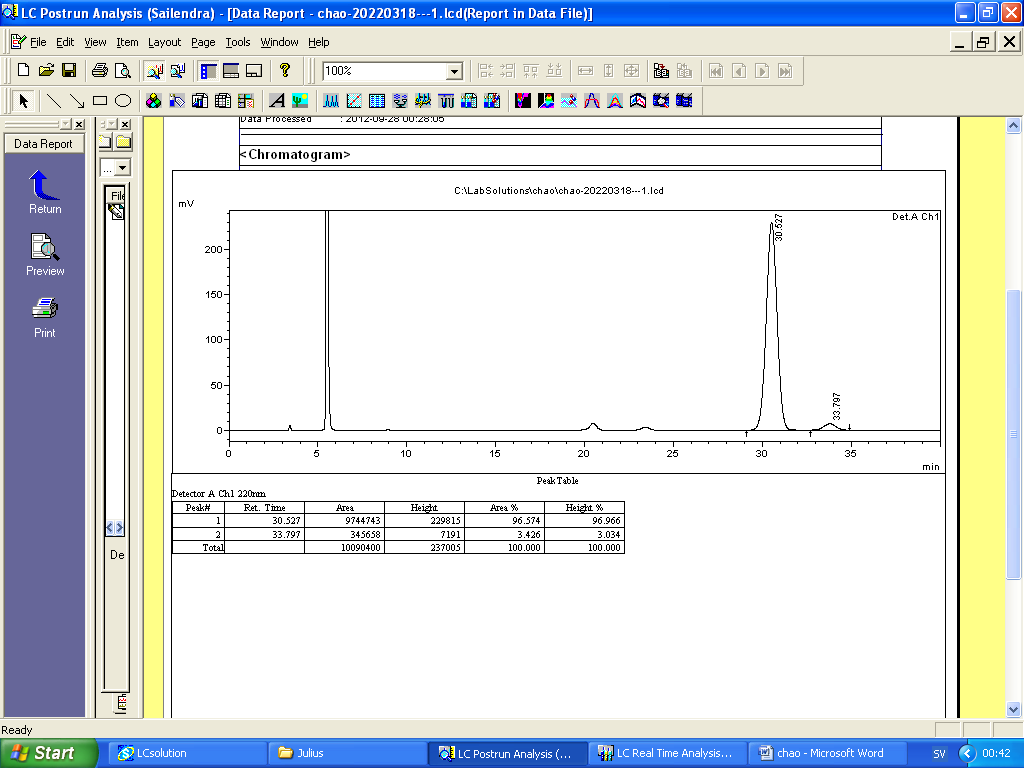


**Supplementary Figure 94.** HPLC chromatogram of (*R*)-7-methoxy-2-methylspiro[chromane-3,1'-cyclohexane]-2',5'-dien-4-one (**5w**).

Spiro[chromane-3,1'-cyclohexane]-2',5'-dien-4-ol (5aa)

^1^H NMR (500 MHz, CD_3_CN)


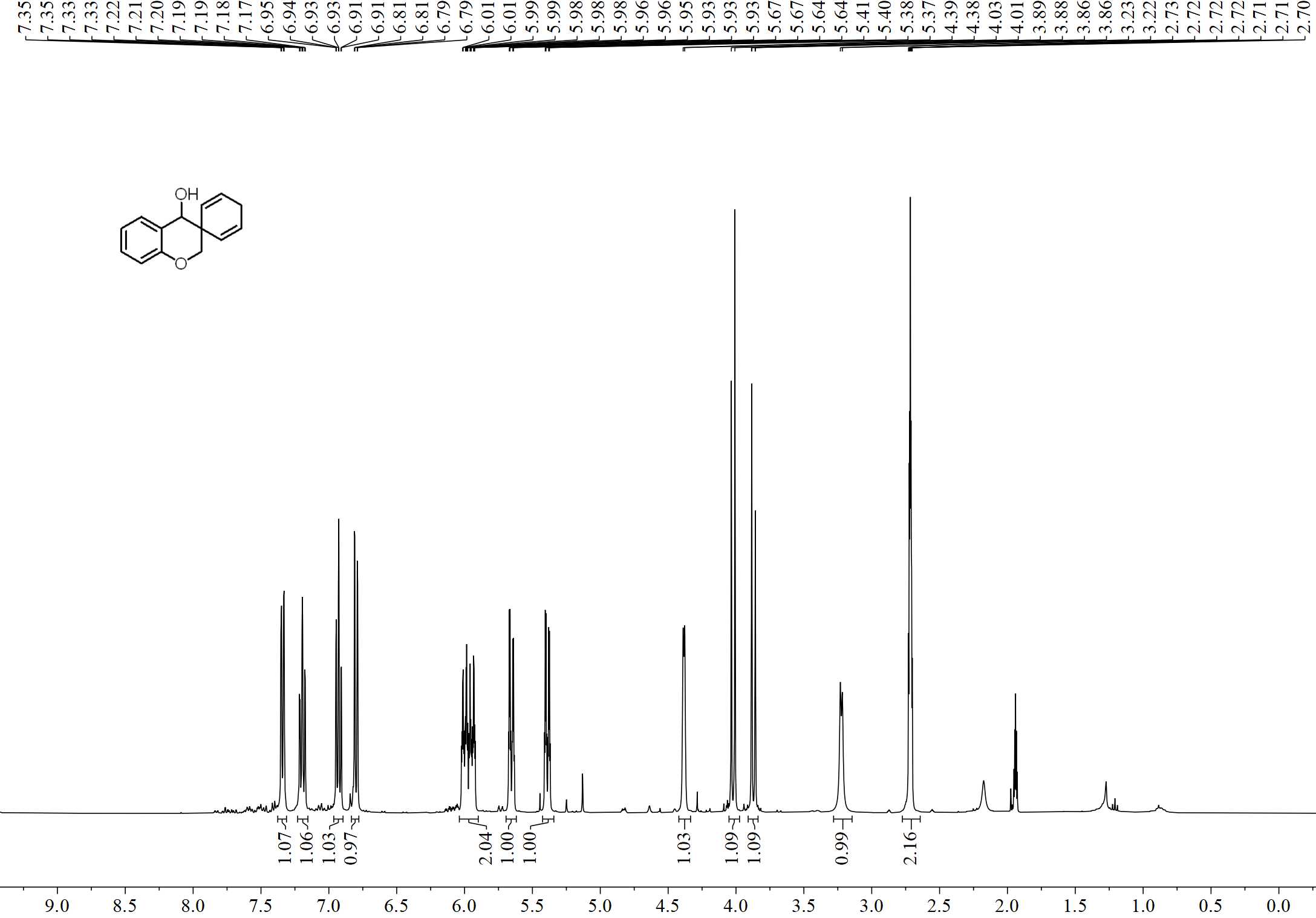


**Supplementary Figure 95.** ^1^H NMR spectra of spiro[chromane-3,1'-cyclohexane]-2',5'-dien-4-ol (**5aa**).

^13^C NMR (101 MHz, CD_3_CN)


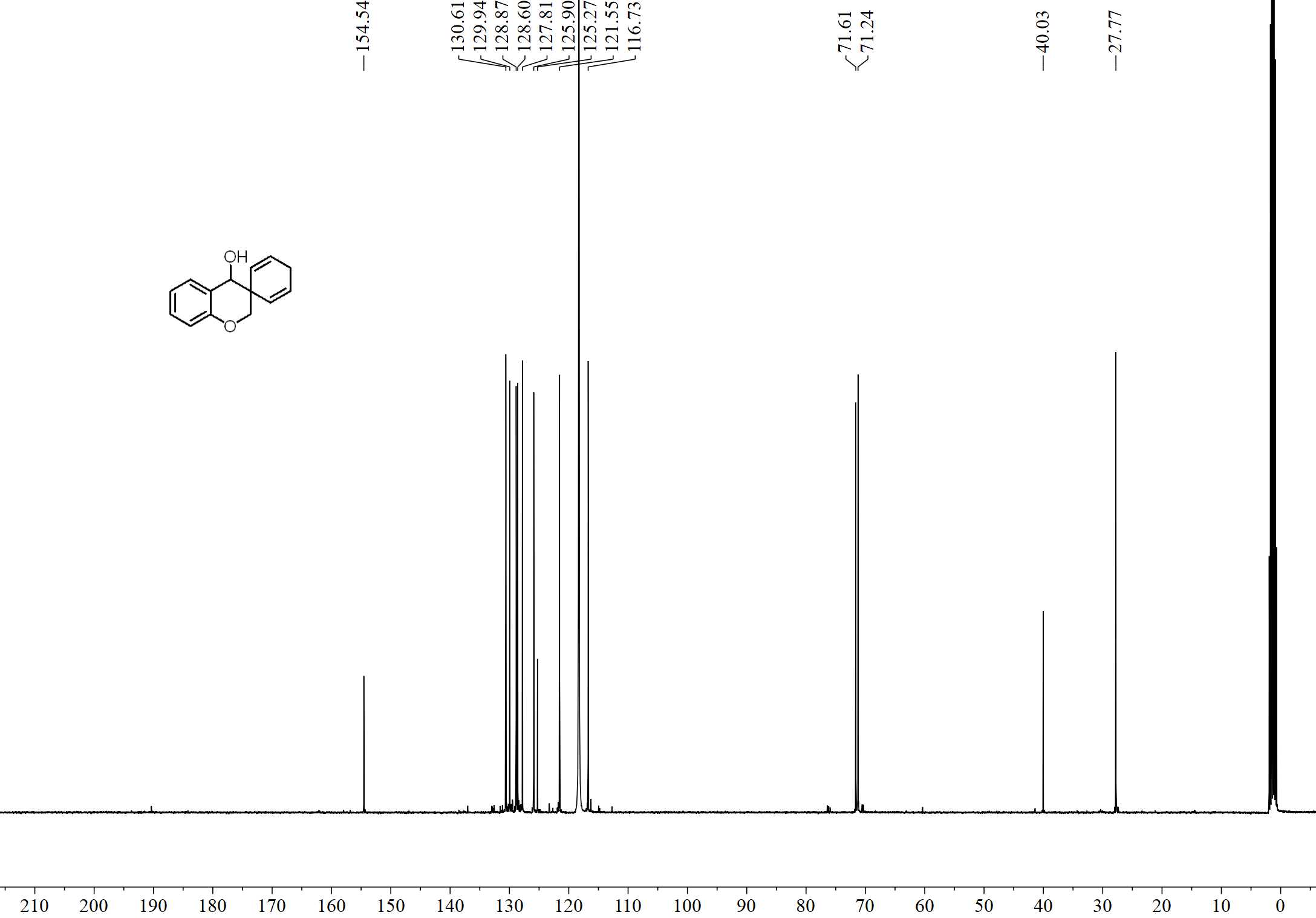


**Supplementary Figure 96.** ^13^C NMR spectra of spiro[chromane-3,1'-cyclohexane]-2',5'-dien-4-ol (**5aa**).

4-Methylspiro[chromane-3,1'-cyclohexane]-2',5'-dien-4-ol (5ab)

^1^H NMR (400 MHz, CD_3_CN)


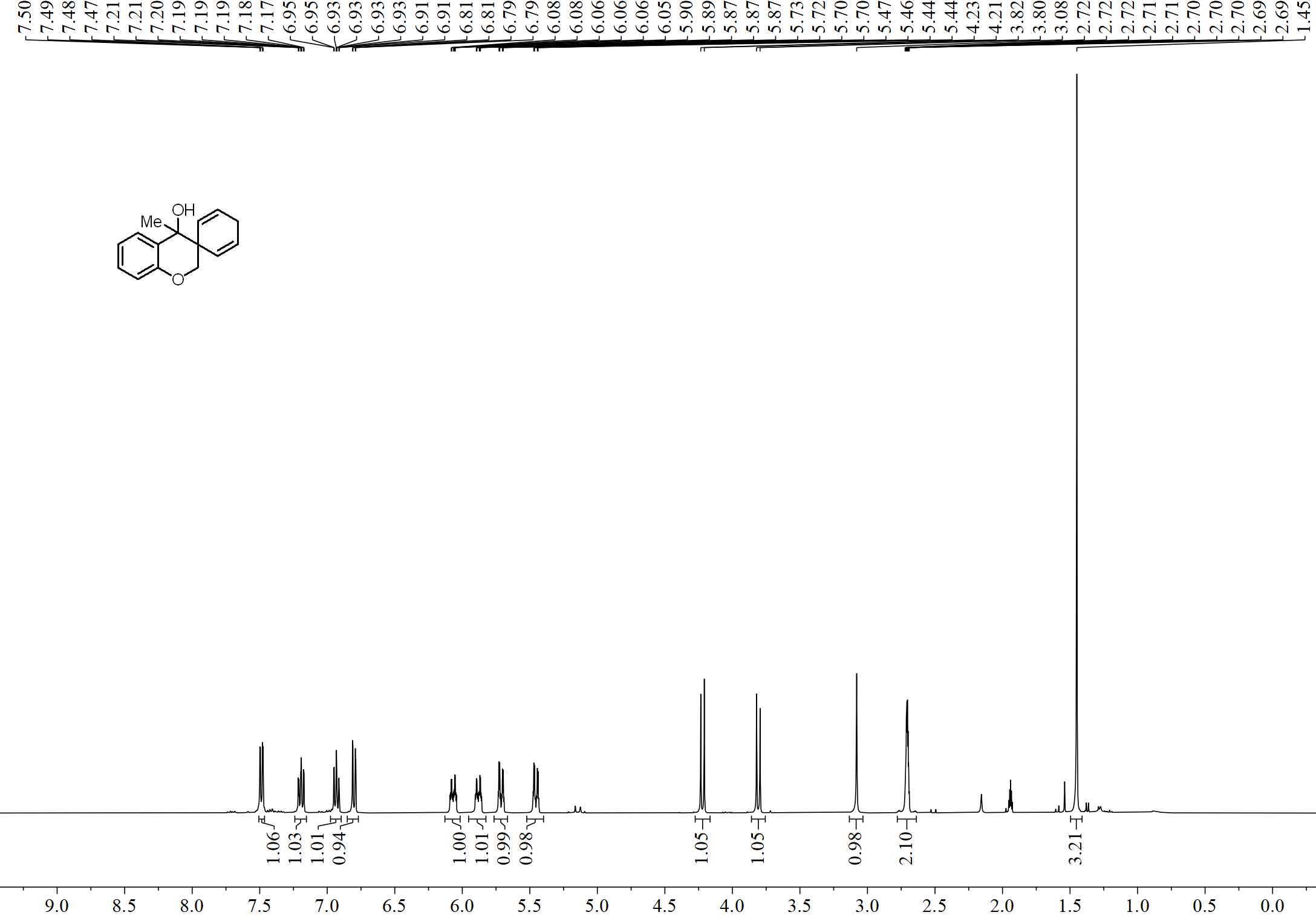


**Supplementary Figure 97.** ^1^H NMR spectra of 4-methylspiro[chromane-3,1'-cyclohexane]-2',5'-dien-4-ol (**5ab**).

^13^C NMR (101 MHz, CD_3_CN)


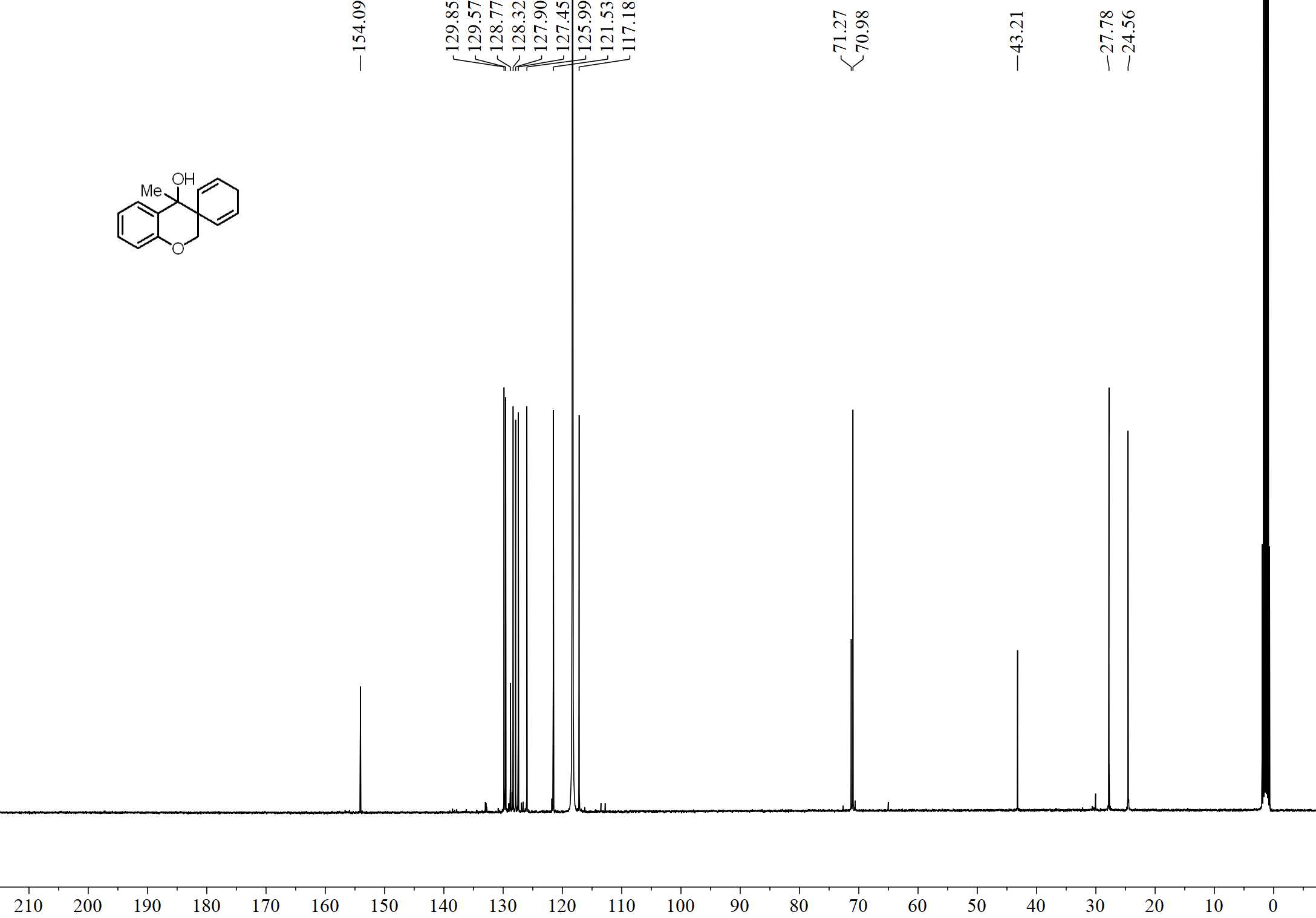


**Supplementary Figure 98.** ^13^C NMR spectra of 4-methylspiro[chromane-3,1'-cyclohexane]-2',5'-dien-4-ol (**5ab**).

Spiro[chromane-3,1'-cyclohexan]-4-ol (5ac)

^1^H NMR (400 MHz, CDCl_3_)


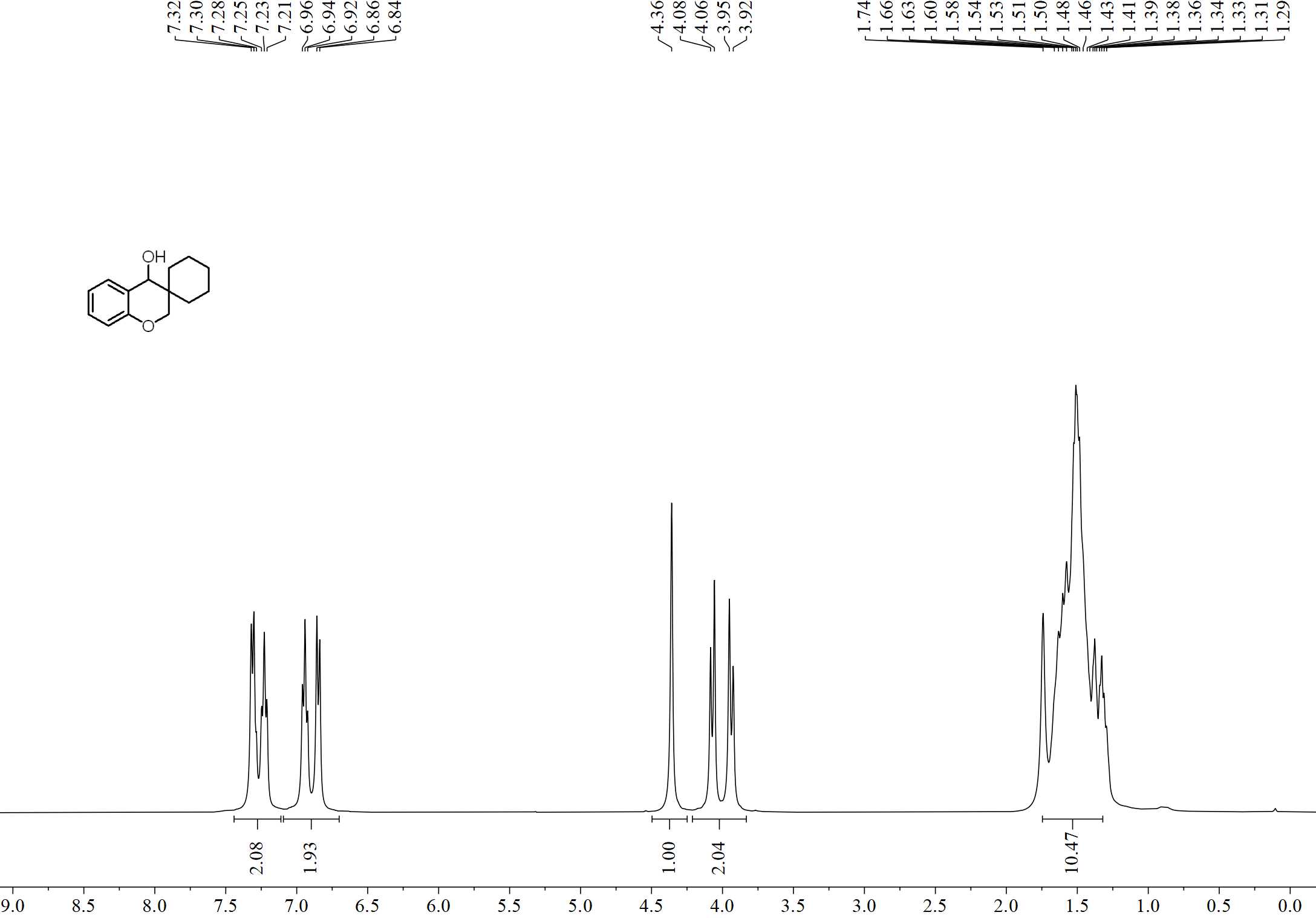


**Supplementary Figure 99.** ^1^H NMR spectra of spiro[chromane-3,1'-cyclohexan]-4-ol (**5ac**).

^13^C NMR (126 MHz, CDCl_3_)


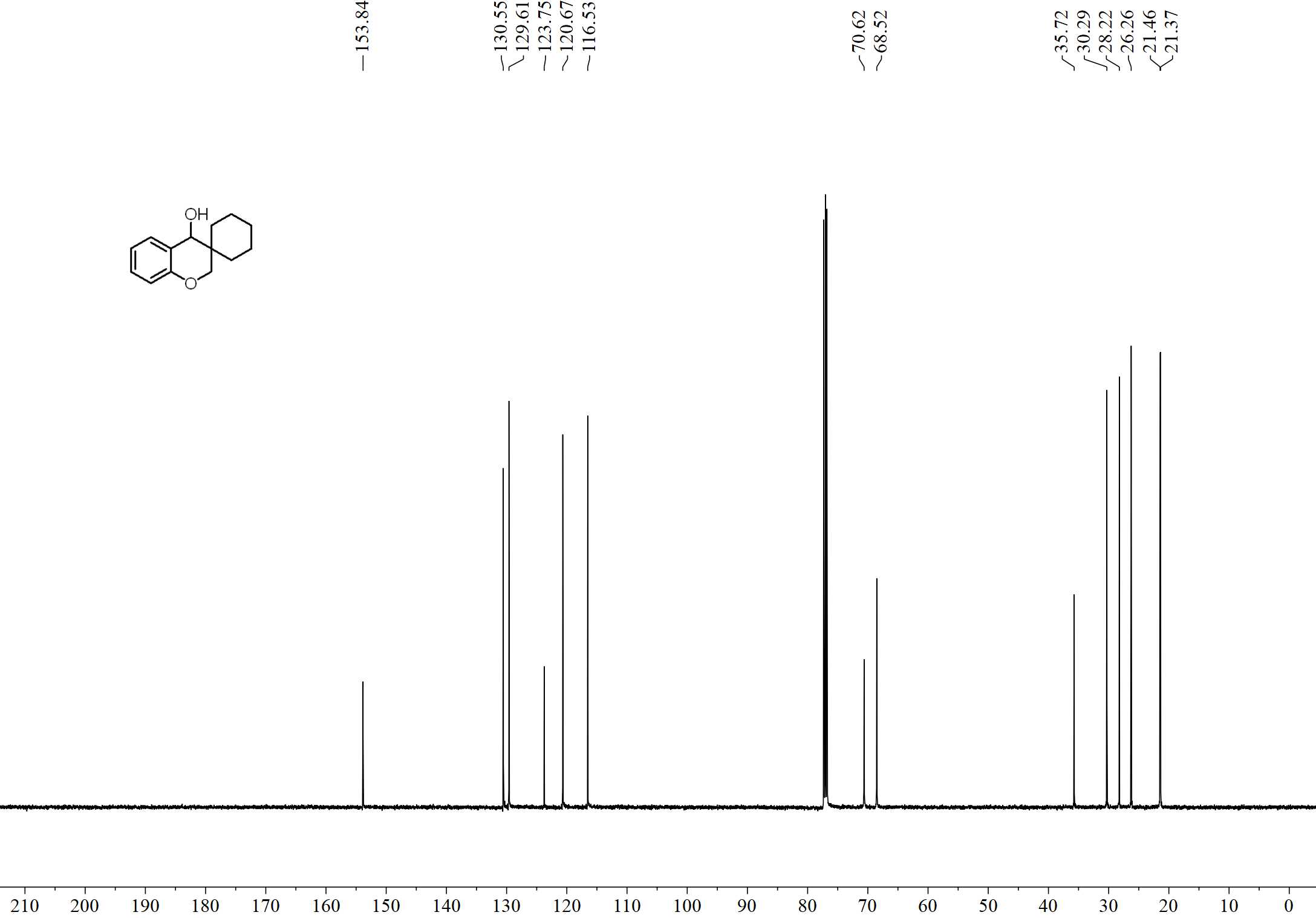


**Supplementary Figure 100.** ^13^C NMR spectra of spiro[chromane-3,1'-cyclohexan]-4-ol (**5ac**).

Spiro[chromane-3,1'-cyclohexane] (5ad)

^1^H NMR (400 MHz, CDCl_3_)


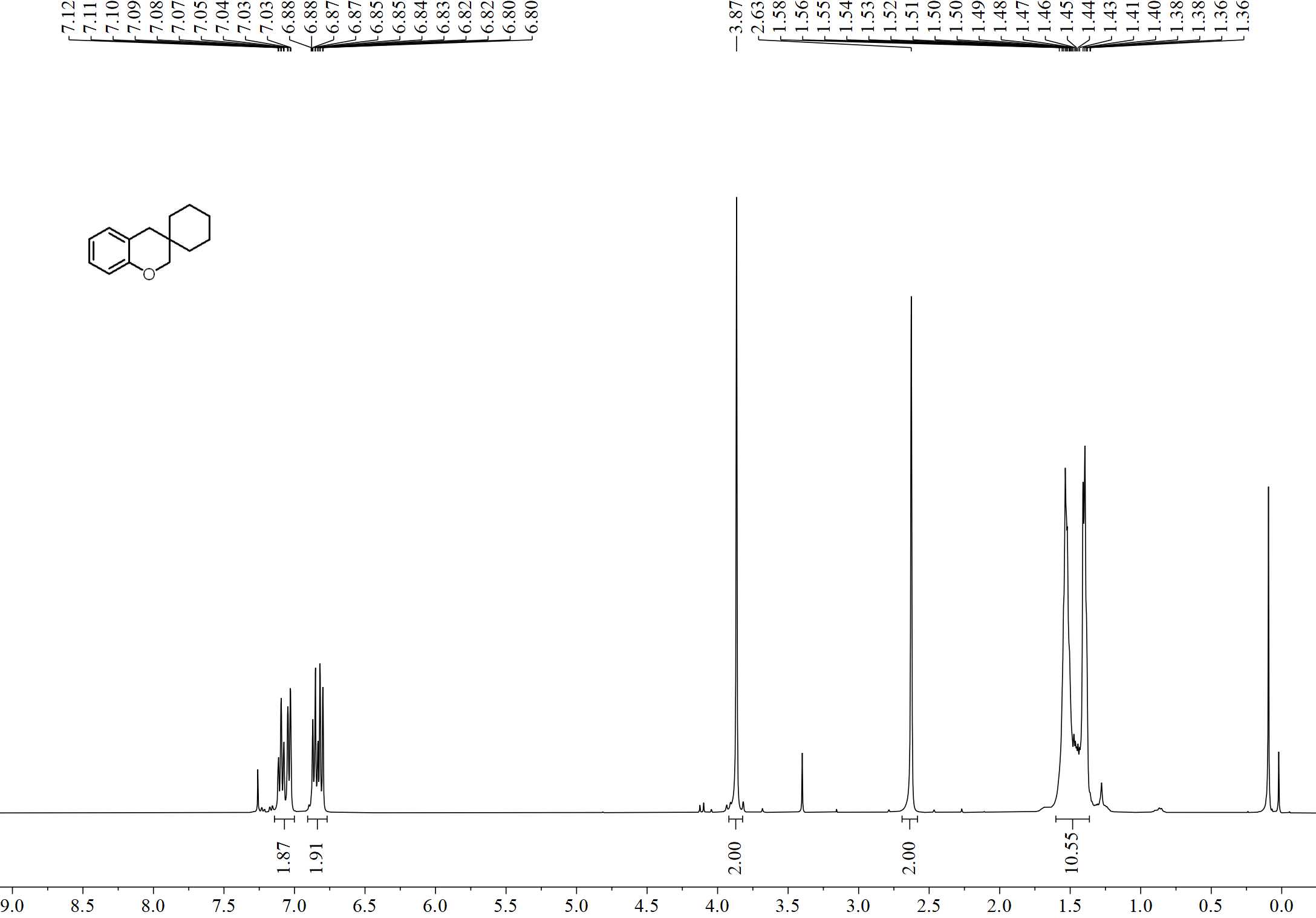


**Supplementary Figure 101.** ^1^H NMR spectra of spiro[chromane-3,1'-cyclohexane] (**5ad**).

^13^C NMR (101 MHz, CDCl_3_)


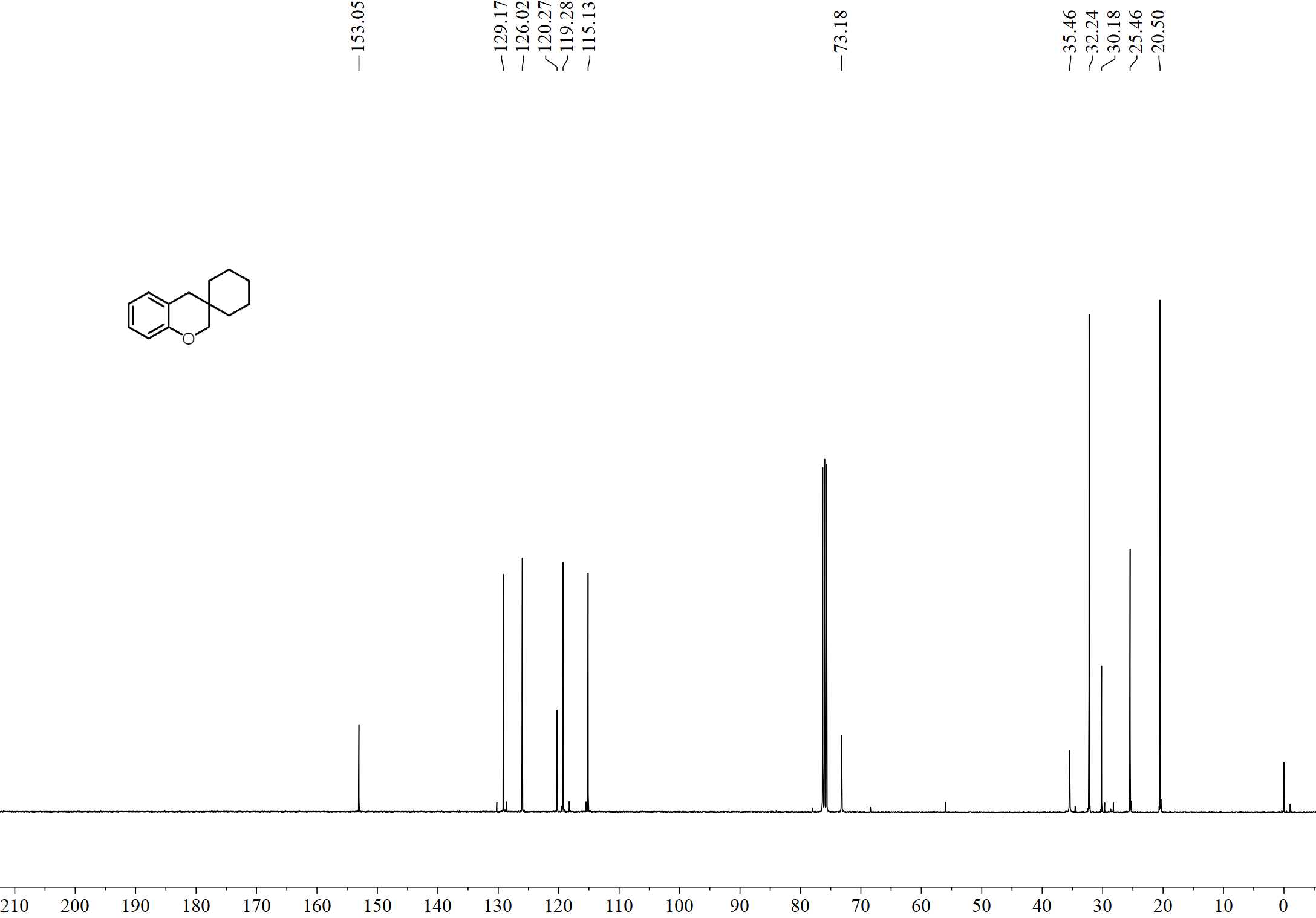


**Supplementary Figure 102.** ^13^C NMR spectra of spiro[chromane-3,1'-cyclohexane] (**5ad**).

3',7-Dimethoxyspiro[chromane-3,1'-cyclohexan]-3'-ene-4,5'-dione (5sa)

^1^H NMR (400 MHz, CDCl_3_)


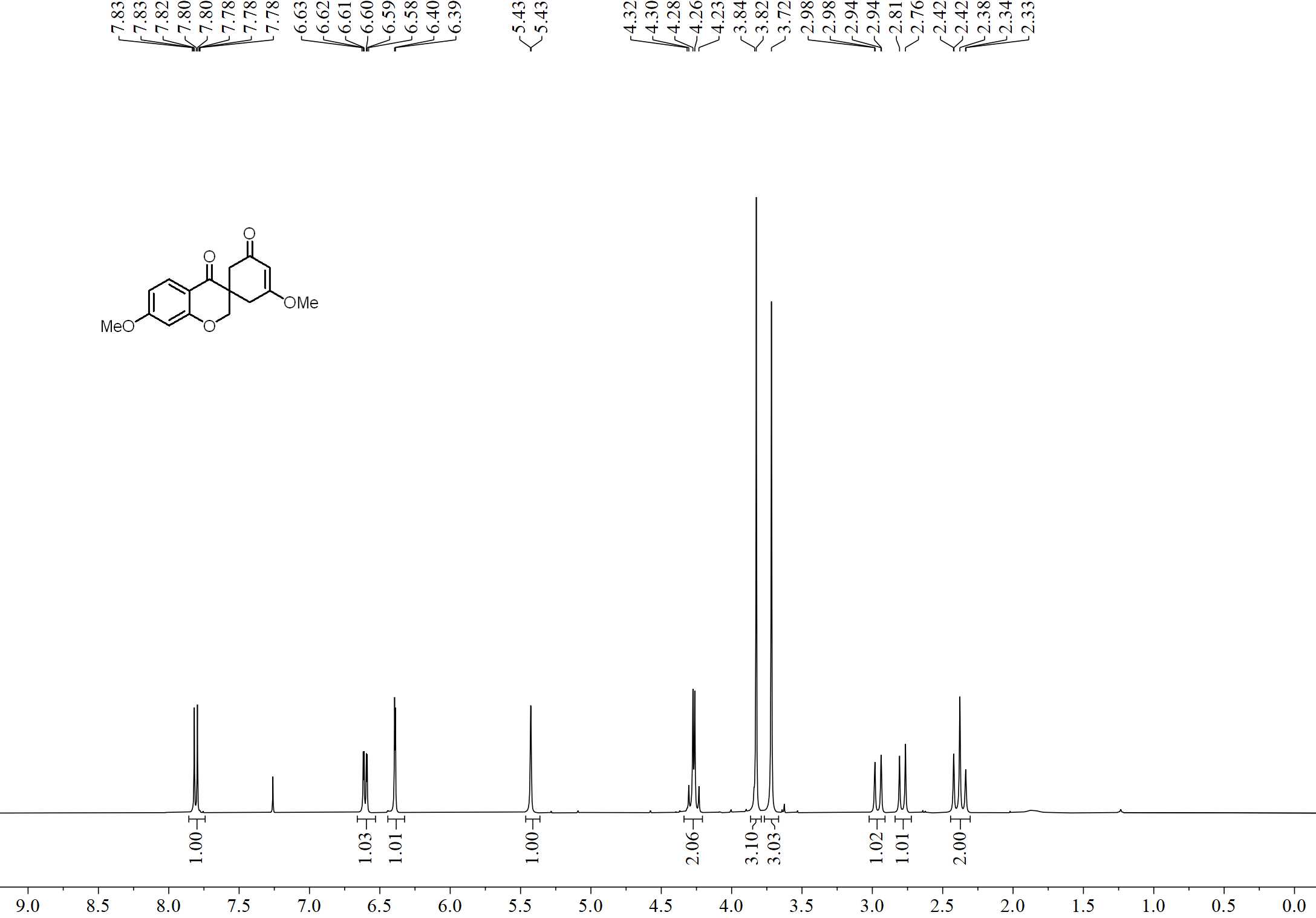


**Supplementary Figure 103.** ^1^H NMR spectra of 3',7-dimethoxyspiro[chromane-3,1'-cyclohexan]-3'-ene-4,5'-dione (**5sa**).

^13^C NMR (101 MHz, CDCl_3_)


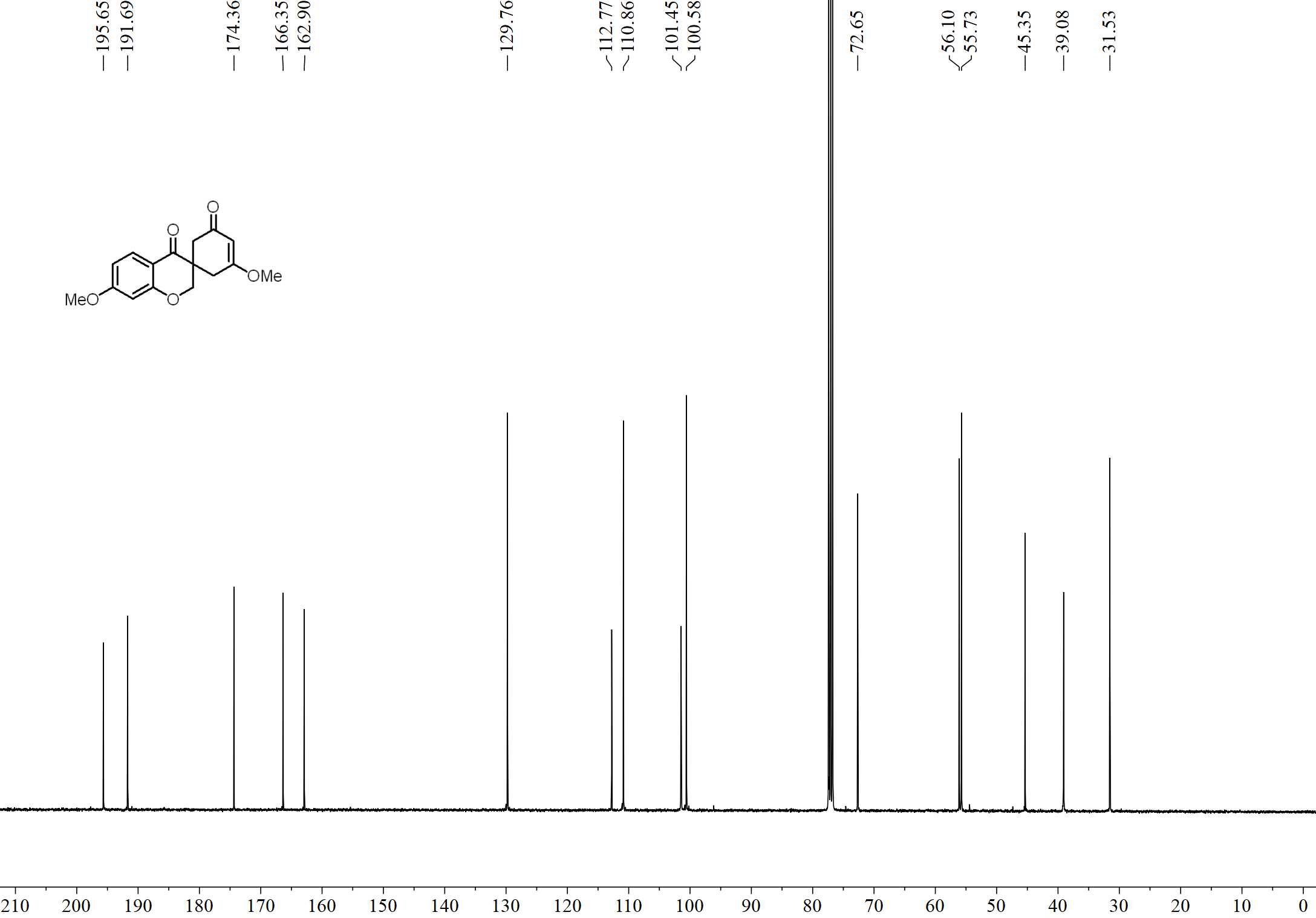


**Supplementary Figure 104.** ^13^C NMR spectra of 3',7-dimethoxyspiro[chromane-3,1'-cyclohexan]-3'-ene-4,5'-dione (**5sa**).

1-(2-(Benzyloxy)phenyl)-3,3-diphenylpropan-1-one (10)

^1^H NMR (400 MHz, CDCl_3_)


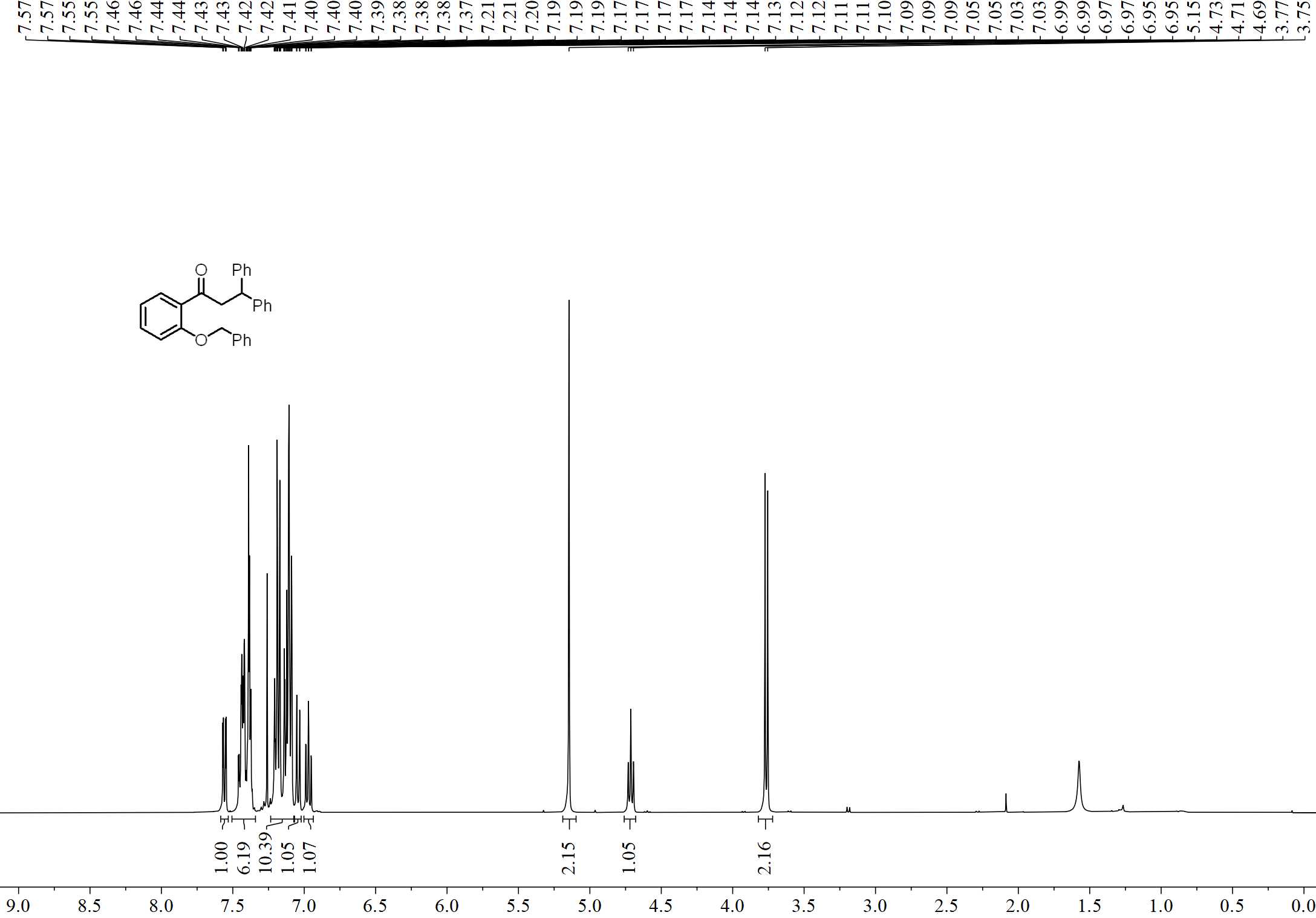


**Supplementary Figure 105.** ^1^H NMR spectra of 1-(2-(benzyloxy)phenyl)-3,3-diphenylpropan-1-one (**10**).

^13^C NMR (101 MHz, CDCl_3_)


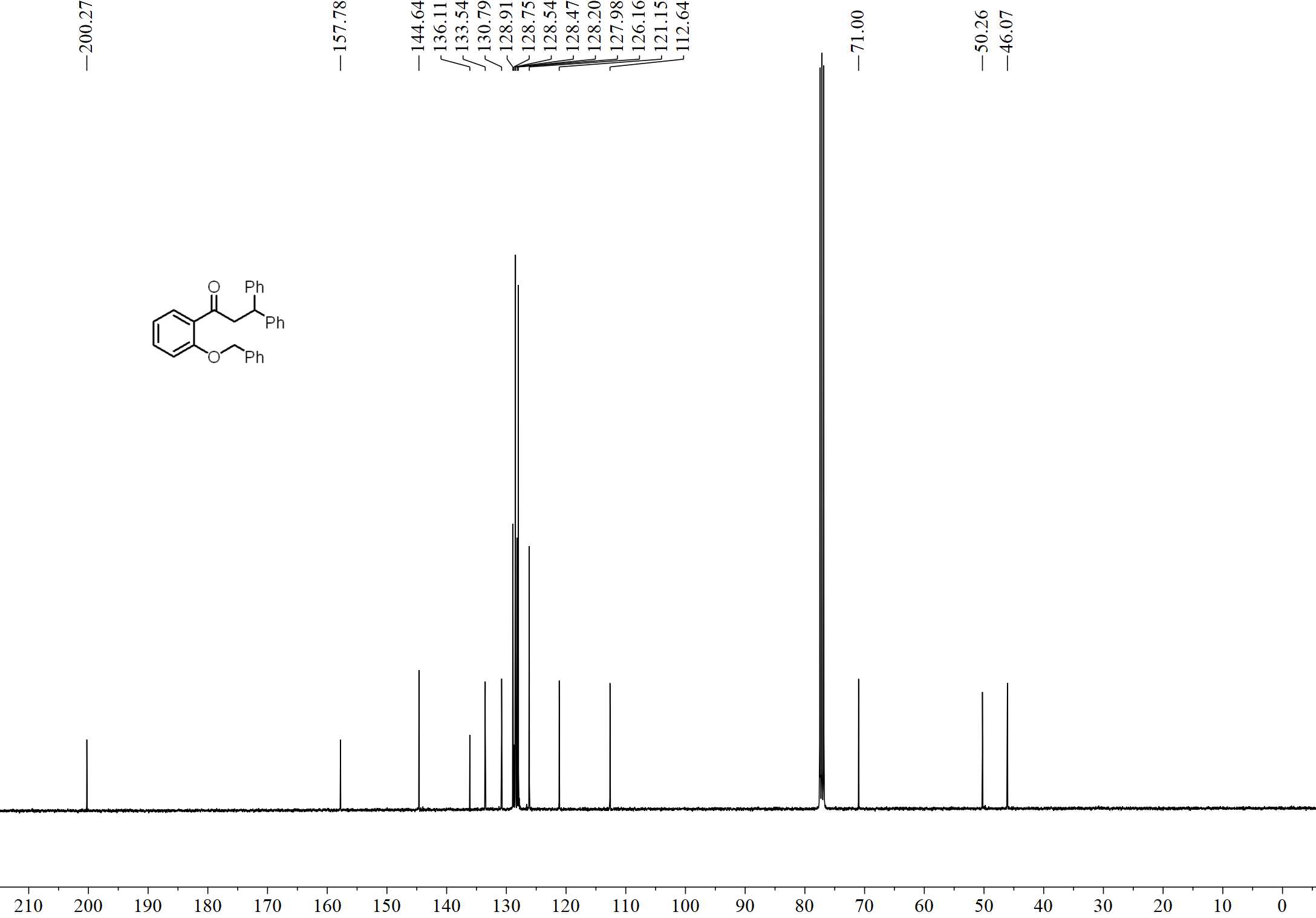


**Supplementary Figure 106.** ^13^C NMR spectra of 1-(2-(benzyloxy)phenyl)-3,3-diphenylpropan-1-one (**10**).

2-(tert-Butyl)-4-(2-oxo-2-phenylethyl)-2-azaspiro[4.5]deca-6,9-dien-3-one (9a)

^1^H NMR (400 MHz, CDCl_3_)

**Supplementary Figure 107.** ^1^H NMR spectra of 2-(*tert*-butyl)-4-(2-oxo-2-phenylethyl)-2-azaspiro[4.5]deca-6,9-dien-3-one (**9a**).

^13^C NMR (101 MHz, CDCl_3_)

**Supplementary Figure 108.** ^13^C NMR spectra of 2-(*tert*-butyl)-4-(2-oxo-2-phenylethyl)-2-azaspiro[4.5]deca-6,9-dien-3-one (**9a**).

2-(tert-Butyl)-4-(2-oxo-2-(p-tolyl)ethyl)-2-azaspiro[4.5]deca-6,9-dien-3-one (9b)

^1^H NMR (400 MHz, CDCl_3_)

**Supplementary Figure 109.** ^1^H NMR spectra of 2-(*tert*-butyl)-4-(2-oxo-2-(p-tolyl)ethyl)-2-azaspiro[4.5]deca-6,9-dien-3-one (**9b**).

^13^C NMR (101 MHz, CDCl_3_)

**Supplementary Figure 110.** ^13^C NMR spectra of 2-(*tert*-butyl)-4-(2-oxo-2-(p-tolyl)ethyl)-2-azaspiro[4.5]deca-6,9-dien-3-one (**9b**).

2-(tert-Butyl)-4-(2-(4-methoxyphenyl)-2-oxoethyl)-2-azaspiro[4.5]deca-6,9-dien-3-one (9c)

^1^H NMR (400 MHz, CDCl_3_)

**Supplementary Figure 111.** ^1^H NMR spectra of 2-(*tert*-butyl)-4-(2-(4-methoxyphenyl)-2-oxoethyl)-2-azaspiro[4.5]deca-6,9-dien-3-one (**9c**).

^13^C NMR (101 MHz, CDCl_3_)

**Supplementary Figure 112.** ^13^C NMR spectra of 2-(*tert*-butyl)-4-(2-(4-methoxyphenyl)-2-oxoethyl)-2-azaspiro[4.5]deca-6,9-dien-3-one (**9c**).

2-(tert-Butyl)-4-(2-(4-fluorophenyl)-2-oxoethyl)-2-azaspiro[4.5]deca-6,9-dien-3-one (9d)

^1^H NMR (400 MHz, CDCl_3_)

**Supplementary Figure 113.** ^1^H NMR spectra of 2-(*tert*-butyl)-4-(2-(4-fluorophenyl)-2-oxoethyl)-2-azaspiro[4.5]deca-6,9-dien-3-one (**9d**).

^13^C NMR (101 MHz, CDCl_3_)

**Supplementary Figure 114.** ^13^C NMR spectra of 2-(*tert*-butyl)-4-(2-(4-fluorophenyl)-2-oxoethyl)-2-azaspiro[4.5]deca-6,9-dien-3-one (**9d**).

^19^F NMR (377 MHz, CDCl_3_)

**Supplementary Figure 115.** ^19^F NMR spectra of 2-(*tert*-butyl)-4-(2-(4-fluorophenyl)-2-oxoethyl)-2-azaspiro[4.5]deca-6,9-dien-3-one (**9d**).

2-(tert-Butyl)-4-(2-(4-chlorophenyl)-2-oxoethyl)-2-azaspiro[4.5]deca-6,9-dien-3-one (9e)

^1^H NMR (400 MHz, CD_3_CN)

**Supplementary Figure 116.** ^1^H NMR spectra of 2-(*tert*-butyl)-4-(2-(4-chlorophenyl)-2-oxoethyl)-2-azaspiro[4.5]deca-6,9-dien-3-one (**9e**).

^13^C NMR (126 MHz, CD_3_CN)

**Supplementary Figure 117.** ^13^C NMR spectra of 2-(*tert*-butyl)-4-(2-(4-chlorophenyl)-2-oxoethyl)-2-azaspiro[4.5]deca-6,9-dien-3-one (**9e**).

2-(tert-Butyl)-4-(2-(2,4-dimethoxyphenyl)-2-oxoethyl)-2-azaspiro[4.5]deca-6,9-dien-3-one (9f)

^1^H NMR (400 MHz, CD_3_CN)

**Supplementary Figure 118.** ^1^H NMR spectra of 2-(*tert*-butyl)-4-(2-(2,4-dimethoxyphenyl)-2-oxoethyl)-2-azaspiro[4.5]deca-6,9-dien-3-one (**9f**).

^13^C NMR (101 MHz, CD_3_CN)

**Supplementary Figure 119.** ^13^C NMR spectra of 2-(*tert*-butyl)-4-(2-(2,4-dimethoxyphenyl)-2-oxoethyl)-2-azaspiro[4.5]deca-6,9-dien-3-one (**9f**)

2-(tert-Butyl)-4-(2-(3,4-dimethylphenyl)-2-oxoethyl)-2-azaspiro[4.5]deca-6,9-dien-3-one (9g)

^1^H NMR (400 MHz, CD_3_CN)

**Supplementary Figure 120.** ^1^H NMR spectra of 2-(*tert*-butyl)-4-(2-(3,4-dimethylphenyl)-2-oxoethyl)-2-azaspiro[4.5]deca-6,9-dien-3-one (**9g**).

^13^C NMR (101 MHz, CD_3_CN)

**Supplementary Figure 121.** ^1^H NMR spectra of spectra of 2-(*tert*-butyl)-4-(2-(3,4-dimethylphenyl)-2-oxoethyl)-2-azaspiro[4.5]deca-6,9-dien-3-one (**9g**).

2-(tert-Butyl)-4-(2-(furan-2-yl)-2-oxoethyl)-2-azaspiro[4.5]deca-6,9-dien-3-one (9h)

^1^H NMR (400 MHz, CD_3_CN)

**Supplementary Figure 122.** ^1^H NMR spectra of 2-(*tert*-butyl)-4-(2-(furan-2-yl)-2-oxoethyl)-2-azaspiro[4.5]deca-6,9-dien-3-one (**9h**).

^13^C NMR (101 MHz, CD_3_CN)

**Supplementary Figure 123.** ^1^H NMR spectra of spectra of 2-(*tert*-butyl)-4-(2-(furan-2-yl)-2-oxoethyl)-2-azaspiro[4.5]deca-6,9-dien-3-one (**9h**).

2-(tert-Butyl)-4-(2-(4-methoxyphenyl)-2-oxoethyl)-6,10-dimethyl-2-azaspiro[4.5]deca-6,9-dien-3-one (9i)

^1^H NMR (400 MHz, CD_3_CN)

**Supplementary Figure 124.** ^1^H NMR spectra of 2-(*tert*-butyl)-4-(2-(4-methoxyphenyl)-2-oxoethyl)-6,10-dimethyl-2-azaspiro[4.5]deca-6,9-dien-3-one (**9i**).

^13^C NMR (101 MHz, CD_3_CN)

**Supplementary Figure 125.** ^13^C NMR spectra of spectra of 2-(*tert*-butyl)-4-(2-(4-methoxyphenyl)-2-oxoethyl)-6,10-dimethyl-2-azaspiro[4.5]deca-6,9-dien-3-one (**9i**).

2-(tert-Butyl)-7,9-dimethoxy-4-(2-(4-methoxyphenyl)-2-oxoethyl)-2-azaspiro[4.5]deca-6,9-dien-3-one (9j)

^1^H NMR (500 MHz, CD_3_CN)

**Supplementary Figure 126.** ^1^H NMR spectra of 2-(*tert*-butyl)-7,9-dimethoxy-4-(2-(4-methoxyphenyl)-2-oxoethyl)-2-azaspiro[4.5]deca-6,9-dien-3-one (**9j**).

^13^C NMR (126 MHz, CD_3_CN)

**Supplementary Figure 127.** ^13^C NMR spectra of 2-(*tert*-butyl)-7,9-dimethoxy-4-(2-(4-methoxyphenyl)-2-oxoethyl)-2-azaspiro[4.5]deca-6,9-dien-3-one (**9j**).

4-(2-(6-(3-((3r,5r,7r)-Adamantan-1-yl)-4-methoxyphenyl)naphthalen-2-yl)-2-oxoethyl)-2-(tert-butyl)-2-azaspiro[4.5]deca-6,9-dien-3-one (9k)

^1^H NMR (400 MHz, CD_3_CN)

**Supplementary Figure 128.** ^1^H NMR spectra of 4-(2-(6-(3-((3r,5r,7r)-adamantan-1-yl)-4-methoxyphenyl)naphthalen-2-yl)-2-oxoethyl)-2-(*tert*-butyl)-2-azaspiro[4.5]deca-6,9-dien-3-one (**9k**).

^13^C NMR (101 MHz, CD_3_CN)

**Supplementary Figure 129.** ^13^C NMR spectra of 4-(2-(6-(3-((3r,5r,7r)-adamantan-1-yl)-4-methoxyphenyl)naphthalen-2-yl)-2-oxoethyl)-2-(*tert*-butyl)-2-azaspiro[4.5]deca-6,9-dien-3-one (**9k**).

# 5. Supplementary references

1. Zhou, C.; Lei, T.; Wei, X.-Z.; Ye, C.; Liu, Z.; Chen, B.; Tung, C.-H.; Wu, L.-Z. *J. Am. Chem. Soc.* **2020**, *142*, 16805–16813.
2. Garreau, M.; Le Vaillant, F.; Waser, J. *Angew. Chem., Int. Ed.* **2019,** *58*, 8182–8186.
3. Speckmeier, E.; Fischer, T. G.; Zeitler, K. *J. Am. Chem. Soc.* **2018,** *140*, 15353–15365.
4. Dalton, N.; Gordon, C. P.; Boyle, T. P.; Vandegraaf, N.; Deadman, J.; Rhodes, D. I.; Coates, J. A.; Pyne, S. G.; Keller, P. A.; Bremner, J. B. *Org. Biomol. Chem.,* **2016**, *14*, 6010–6023.
5. Mao, Y.; Liu, Y.; Hu, Y.; Wang, L.; Zhang, S.; Wang, W. *ACS Catal*. **2018**, *8*, 3016–3020.
6. Nuthakki, V. K.; Mudududdla, R.; Sharma, A.; Kumar, A.; Bharate, S. B. *Bioorg. Chem.* **2019**, *90*, 103062.
7. Stasiak, B.; Czapik, A.; Kwit, M. *J. Org. Chem.* **2021**, *86*, 643–656.
8. McDaniel, K. A.; Blood, A. R.; Smith, G. C.; Jui, N. T. *ACS Catal.* **2021**, *11*, 4968–4972.
9. Clayden, J.; Turnbull, R.; Pinto, I. *Org. Lett.* **2004**, 6, 609–611.
10. Ghosh, A.; Brueckner, A. C.; Cheong, P. H.-Y.; Carter, R. G. *J. Org. Chem.* **2019**, *84*, 9196–9214.
11. Laursen, J. B.; de Visser, P. C.; Nielsen, H. K.; Jensen, K. J.; Nielsen, J. *Bioorg. Med. Chem. Lett.* **2002**, *12*, 171–175.
12. Chen, J.; Lu, W.; Chen, H.; Bian, X.; Yang, G. *Biol. Pharm. Bull.* **2019**, *42*, 231–246.
13. Cohen, S. M.; Meyer, M.; Raymond, K. N. *J. Am. Chem. Soc.* **1998**, 120, 6277–6286.
14. Ding, X.; Dai, X.; Long, K.; Peng, C.; Andreotti, D.; Bamborough, P.; Eatherton, A. J.; Edge, C.; Jandu, K. S.; Nichols, P. L.; Philps, O. J.; Stasi, L. P.; Wan, Z.; Xiang, J.-N.; Dong, K.; Dossang, P.; Ho, M.-H.; Li, Y.; Mensah, L.; Guan, X.; Reith, A. D.; Ren, F. *Bioorg. Med. Chem. Lett.* **2017**, *27*, 4034–4038.
15. Madea, D.; Slanina, T.; Klán, P. *Chem. Commun.* **2016**, 52, 12901–12904.
16. Zhou, G.; Lim, D.; Coltart, D. M. *Org. Lett.* **2008**, *10*, 3809–3812.
